# *Ortho*-ester-substituted diaryliodonium salts enabled regioselective arylocyclization of naphthols toward 3,4-benzocoumarins

**DOI:** 10.3762/bjoc.20.76

**Published:** 2024-04-18

**Authors:** Ke Jiang, Cheng Pan, Limin Wang, Hao-Yang Wang, Jianwei Han

**Affiliations:** 1 Key Laboratory for Advanced Materials and Feringa Nobel Prize Scientist Joint Research Center, Department of Fine Chemistry and Institute of Fine Chemicals, School of Chemistry and Molecular Engineering, East China University of Science and Technology, 130 Meilong Road, Shanghai 200237, P. R. China; 2 National Center for Organic Mass Spectrometry in Shanghai, Shanghai Institute of Organic Chemistry, The Chinese Academy of Sciences, 345 Lingling Road, Shanghai 200032, China

**Keywords:** annulation, arylocyclization, 3,4-benzocoumarin, diaryliodonium salts, naphthol

## Abstract

Cyclic annulation involving diaryliodonium salts is an efficient tool for the construction of two or more chemical bonds in a one-pot process. *Ortho*-functionalized diaryliodonium salts have showcased distinct reactivity in the exploration of benzocyclization or arylocyclization. With this strategy of *ortho*-ester-substituted diaryliodonium salts, herein, we utilized a copper catalyst to activate the C–I bond of diaryliodonium salts in the generation of aryl radicals, thus resulting in an annulation reaction with naphthols and substituted phenols. This approach yielded a diverse array of 3,4-benzocoumarin derivatives bearing various substituents.

## Introduction

**Scheme 1:**
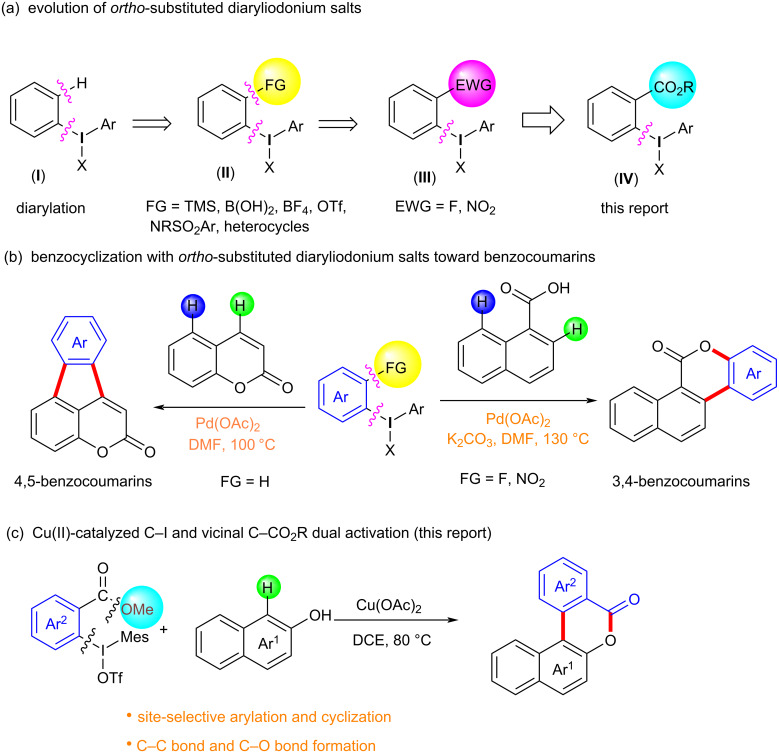
Arylation reactions of aromatic compounds and reaction patterns of *ortho*-functionalized diaryliodonium salts.

Diaryliodonium salts as electrophilic reagents have attracted significant attention in the field of organic synthesis owing to their efficiency and selectivity [1–7]. Particularly, they have been employed in benzocyclization and arylocyclization reactions, enabling intramolecular cyclization by forming aromatic or heterocyclic rings as a part of cyclic structures [8]. In these reactions, the dual activation of a C–I bond and vicinal C–H bonds/functional groups features a distinct advantage, facilitating the formation of two or more chemical bonds in a step-economic manner [9–13]. In a prior study, we reported a palladium-catalyzed efficient activation of both C–I bond and the adjacent C–H bond of diaryliodonium salts in the formation of 4,5-benzocoumarin derivatives, expanding the benzocoumarin family (Scheme 1b) [14]. Recently, *ortho*-functionalized diaryliodonium salts, due to their coordinating and electrophilic effects, have exhibited unique reactivity and chemoselectivity [15]. As such, a wide range of functional groups including the trimethylsilyl group, boronic acid, trifluoroborate moiety, trifluoromethanesulfonate, aryl sulfonamides, and heterocycles, have been incorporated into the *ortho*-position of diaryliodonium structures [16–21]. *Ortho*-trimethylsilyl or boronic acid-substituted diaryliodonium salts can serve as aryne precursors. *Ortho*-trifluoroborate-substituted diaryliodonium salts furnished iodonium zwitterions as bifunctional reagents [22–25]. Additionally, *ortho*-trifluoromethanesulfonate, *N*-sulfonyl, or tosylmethylene-substituted diaryliodonium salts can undergo intramolecular aryl migrations [26–28]. More recently, we explored the reactivity of *ortho*-functionalized diaryliodonium salts containing electron-withdrawing groups (EWGs) such as fluorine and nitro groups [29–30]. These *ortho*-substituted diaryliodonium salts undergo selective benzocyclizations and arylocyclizations with aromatic acids, leading to 3,4-benzocoumarin skeletons in the presence of palladium catalysts (Scheme 1b). Furthermore, Olofsson and colleagues described an unprecedented reaction pathway using *ortho*-fluoro-substituted diaryliodonium salts bearing strong electron-withdrawing groups, leading to novel diarylations of N-, O-, and S-nucleophiles [31–33]. Building on our great interest in *ortho*-functionalized diaryliodonium salts and their dual activation capabilities, we sought to incorporate carboxylic ester groups into the structures of *ortho*-substituted diaryliodonium salts to explore their properties and reactivity. Our previous investigations demonstrated the ability of diaryliodonium salts for selective mono-arylation of 2-naphthols [34]. In this context, we embark on a strategy to modify the neighbouring position of the diaryliodonium salt with an ester group, presenting a novel copper-catalysed regioselective arylocyclization of naphthols and substituted phenols. This method represents an efficient approach to access 3,4-benzocoumarin derivatives (Scheme 1c).

## Results and Discussion

To start the study, we used 2-naphthol (**1a**) and 1.1 equivalents of *ortho*-methyl formate-substituted diaryliodonium salt **2a** as template substrates. The reaction was performed in the presence of 10 mol % Cu(OTf)_2_ and 1.0 equivalent of K_2_CO_3_ in DCE at a temperature of 80 °C. To our delight, the reaction afforded 3,4-benzocoumarin **3aa** in a 27% yield (Table 1, entry 1). The structure of **3aa** was confirmed through NMR spectroscopy and mass spectra analysis. Subsequently, we started to screen various bases such as Na_2_CO_3_, Cs_2_CO_3_, KOH, NaO*t-*Bu, LiHMDS, and DMAP (Table 1, entries 2–7). Fortunately, it was found that the reaction yield was increased to 50% in the absence of any base (Table 1, entry 8). Further investigations for assessing the influence of various solvents including dimethyl sulfoxide (DMSO), *N*,*N*-dimethylformamide (DMF), toluene, acetic acid (AcOH) and water (Table 1, entries 9–13) were carried out. However, polar solvents such as AcOH and H_2_O were proved to be unsuitable for this reaction. For catalysts, we found that Cu(OAc)_2_ gave the best results (Table 1, entries 15–18). Finally, the reaction temperature and time were optimized, **3aa** was produced in 61% yield at a temperature of 80 °C after 3 hours (Table 1, entry 15).

**Table 1:** Optimization of reaction conditions.^a^

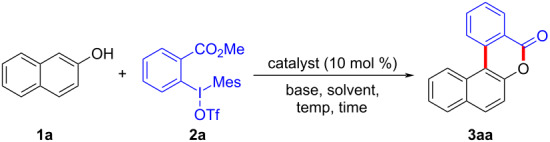				

Entry	Solvent	Base	Catalyst	**3aa** (%)^b^

1	DCE	K_2_CO_3_	Cu(OTf)_2_	27
2	DCE	Na_2_CO_3_	Cu(OTf)_2_	25
3	DCE	Cs_2_CO_3_	Cu(OTf)_2_	16
4	DCE	KOH	Cu(OTf)_2_	24
5	DCE	DMAP	Cu(OTf)_2_	26
6	DCE	NaO*t*-Bu	Cu(OTf)_2_	35
7	DCE	LiHMDS	Cu(OTf)_2_	30
8	DCE	–	Cu(OTf)_2_	50
9	DMSO	–	Cu(OTf)_2_	45^c^ (40)^d^
10	DMF	–	Cu(OTf)_2_	23
11	toluene	–	Cu(OTf)_2_	10
12	AcOH	–	Cu(OTf)_2_	0
13	H_2_O	–	Cu(OTf)_2_	0
14^e^	DCE	–	Cu(OTf)_2_	48
15	DCE	–	Cu(OAc)_2_	61
16	DCE	–	Pd(OAc)_2_	22
17	DCE	–	PdCl_2_	40
18	DCE	–	AgOAc	20

^a^Reaction conditions: **1a** (0.3 mmol, 1 equiv), **2a** (0.33 mmol, 1.1 equiv), base (0.3 mmol; 1 equiv), catalyst (10 mol %), solvent (2 mL), 80 °C, 3 hours. ^b^Isolated yields were obtained after purification by column chromatography. ^c^The reaction temperature was 110 °C. ^d^The reaction temperature was 130 °C. ^e^The reaction was quenched after 12 hours.

With the optimized reaction conditions in hand, we started to explore the substrate scope of the cyclization to construct a variety of 3,4-benzocoumarin derivatives. Our investigations commenced with 2-naphthol (**1**), and the results are presented in Table 2. Various substituted naphthols with a broad range of substituents on the naphthalene unit were well tolerated in the reaction, affording the corresponding products **3aa**–**aq** in generally moderate to good yields of 22–83% (Table 2, entries 1–17). These substituents included halogen (Br), methyl, phenyl, aldehyde, ester, and methoxy groups, all of which were compatible with the reaction conditions. Notably, compounds **3ab**, **3ah**, **3aj**, **3am** and **3ap** bearing bromine are very useful modules for the synthesis of functional materials via cross-coupling reactions. Next, we extended our investigation to 1-naphthol in this reaction, and found that the arylation of 1-naphthol was achieved selectively at the C-2 position. The cascade cyclization resulted in the corresponding products **3an** and **3ao** in yields of 49% and 40%, respectively (Table 2, entries 14 and 15). When 5,6,7,8-tetrahydro-2-naphthol was subjected to the reaction, we obtained products **3ar** and **3as** as a mixture (40% and 10% yield, respectively, Table 2, entry 18). However, when naphthol bearing a strong electron-withdrawing group (such as a nitro group) in the *para* position was reacted, the corresponding product could not be obtained, but instead the O-arylated product **3at** was obtained (Table 2, entry 19). Apart from naphthol, we also tested substituted phenols under the standard conditions. The corresponding products of **3au** and **3av** were produced in 34% and 39% yields, respectively, in which methoxy and *tert-*butyl groups were located in the *para* position to the hydroxy group (Table 2, entries 20 and 21). In the case of **3al**, the mono-arylation of naphthol generated **3al’** in 20% isolated yield, which is the reason for the low yield of **3al**.

**Table 2:** Scope of naphthols and phenols for the synthesis of 3,4-benzocoumarins.^a,b^.

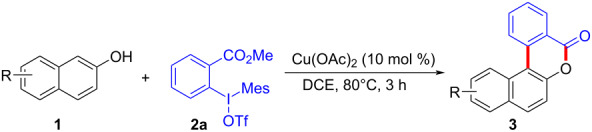				

Entry	**1**	Product		Yield (%)^b^

1	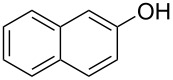	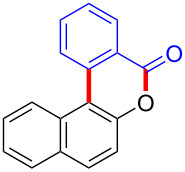 **3aa**		61
2	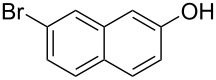	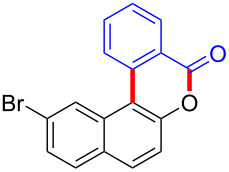 **3ab**		63
3	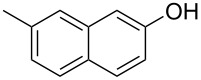	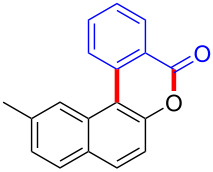 **3ac**		80
4	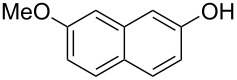	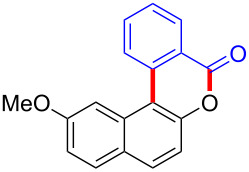 **3ad**		77
5	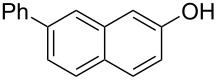	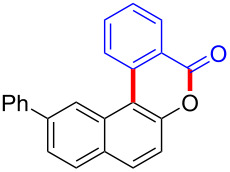 **3ae**		26
6	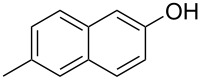	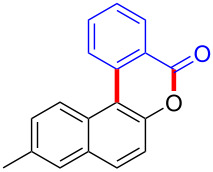 **3af**		31
7	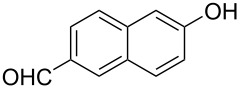	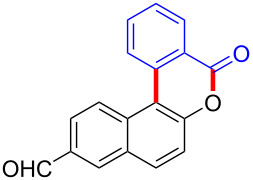 **3ag**		28
8	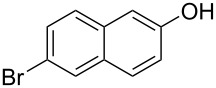	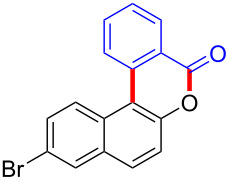 **3ah**		54
9	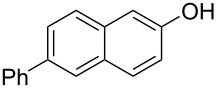	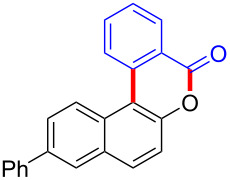 **3ai**		25
10	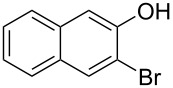	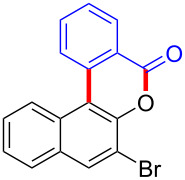 **3aj**		22
11	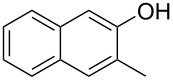	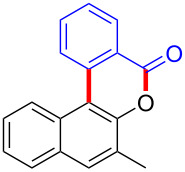 **3ak**		49
12	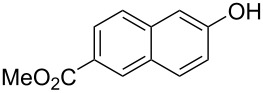	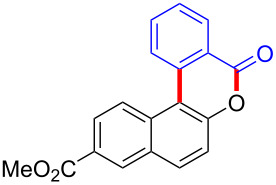 **3al**	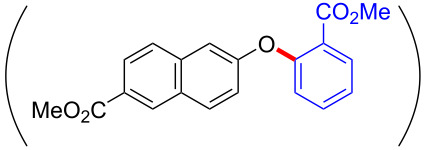 **3al’**	48 (20)
13	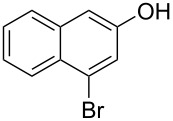	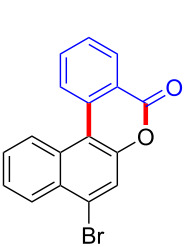 **3am**		3
14	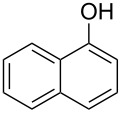	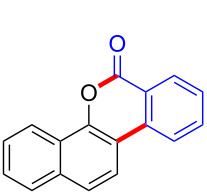 **3an**		49
15	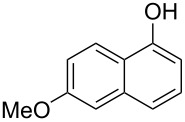	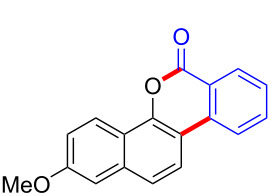 **3ao**		40
16	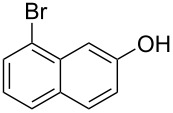	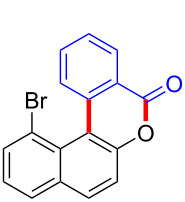 **3ap**		25
17	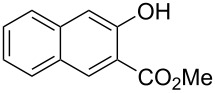	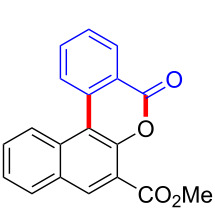 **3aq**		43
18	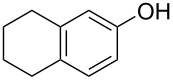	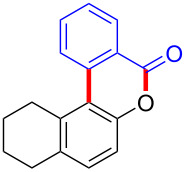 **3ar**	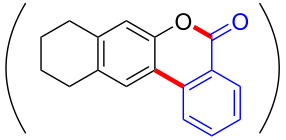 **3as**	45 (10)
19	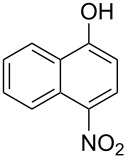	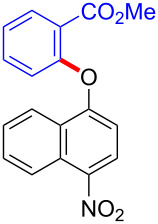 **3at**		51
20	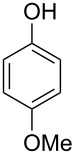	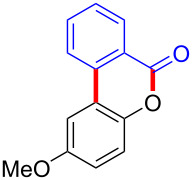 **3au**		34
21	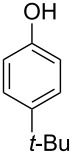	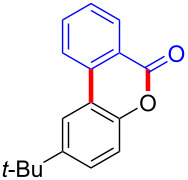 **3av**		39

^a^Reaction conditions: **1** (0.3 mmol, 1 equiv), **2a** (0.33 mmol, 1.1 equiv), Cu(OAc)_2_ (10 mol %), DCE (2 mL), 80 °C, 3 hours. ^b^Isolated yields were obtained after purification with column chromatography. Mes = 2,4,6-trimethylphenyl, OTf = trifluoromethansulfonate.

We subsequently turned our attention to explore the effect of structural diversity of the *ortho*-ester-substituted diaryliodonium salts. Firstly, a family of substituted diaryliodonium salts were synthesized in a one-pot procedure. These *ortho*-substituted diaryliodonium salts were isolated as stable solids, whose structures were fully characterized by NMR spectroscopy. As shown in Table 3, we utilized 2-naphthol and 1-naphthol as template substrates to react with various unsymmetrical 2-ester-substituted diaryliodonium salts. Remarkably, iodonium salts **2** proved to be versatile in this reaction, regardless of the electronic nature and position of the substituents. The desired 3,4-benzocoumarin products **3ba**–**ma** were obtained in yields of 21–59%. Notably, substituents such as halogens (F, Cl, and Br), methyl, methoxy, and trifluoromethyl groups at the *ortho*-, *meta*-, or *para*-positions to the ester group were all well-tolerated (Table 3).

**Table 3:** Scope of *ortho*-ester-substituted diaryliodonium salts.^a^

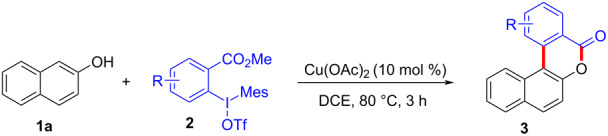			

Entry	**2**	Product	Yield (%)^b^

1	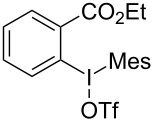 **2b**	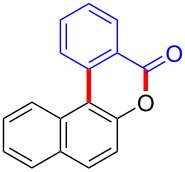 **3aa**	55
2	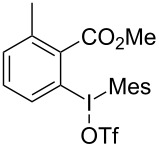 **2c**	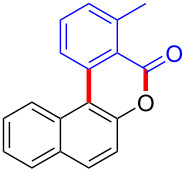 **3ca**	32
3	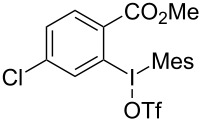 **2d**	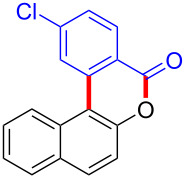 **3da**	50
4	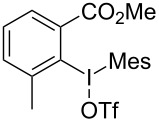 **2e**	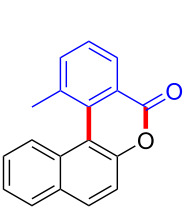 **3ea**	46
5	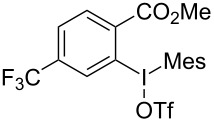 **2f**	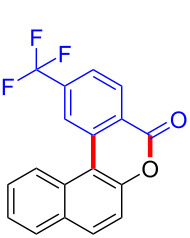 **3fa**	49
6	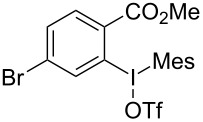 **2g**	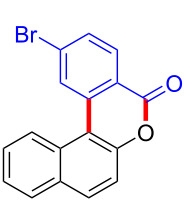 **3ga**	55
7	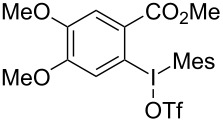 **2h**	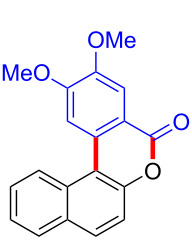 **3ha**	43
8	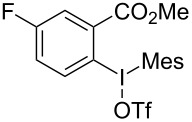 **2i**	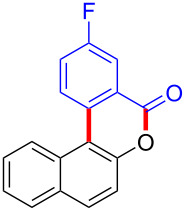 **3ia**	21
9	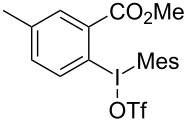 **2j**	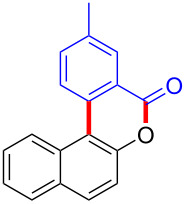 **3ja**	59
10^c^	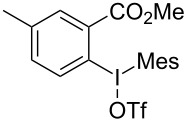 **2j**	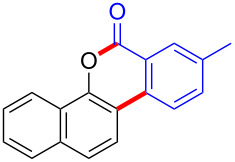 **3jb**	28
11	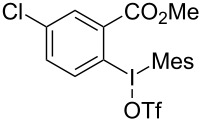 **2k**	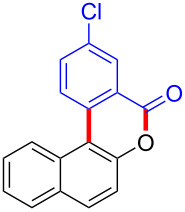 **3ka**	37
12^c^	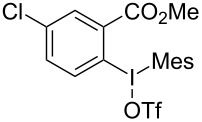 **2k**	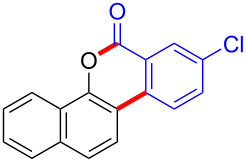 **3kb**	45
13	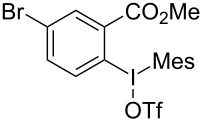 **2l**	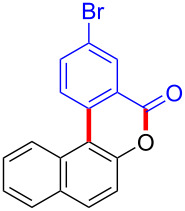 **3la**	35
14	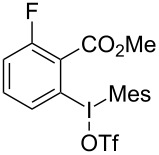 **2m**	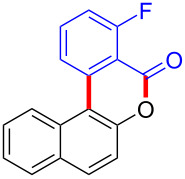 **3ma**	50

^a^Reaction conditions: **1** (0.3 mmol, 1 equiv), **2** (0.33 mmol, 1.1 equiv), Cu(OAc)_2_ (10 mol %), DCE (2 mL), 80 °C, 3 hours. ^b^Isolated yields were obtained after purification with column chromatography. ^c^1-Naphthol was used instead of 2-naphthol. Mes = 2,4,6-trimethylphenyl, OTf = trifluoromethansulfonate.

To gain further insights into the reaction mechanism, we conducted control experiments. Given the utility of diaryliodonium salts in radical chemistry, we introduced 2 equivalents of 2,2,6,6-tetramethylpiperidine-1-oxyl (TEMPO) or 2 equivalents of butylated hydroxytoluene (BHT) into the template reaction. Remarkably, we observed that the desired product was not formed, suggesting a radical pathway. Subsequently, we investigated the bond-formation sequence in the benzocyclization reaction. A possible intermediate of **3al’** was prepared and tested in the reaction under the standard conditions, however, product **3aa** was not obtained.

**Scheme 2:**
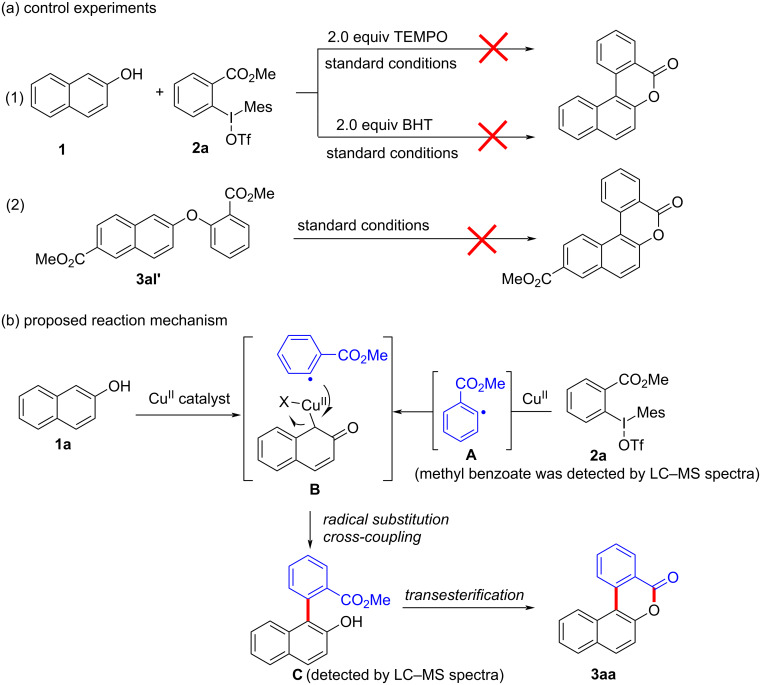
Mechanism study. Standard conditions: **1** (0.3 mmol, 1 equiv), **2** (0.33 mmol, 1.1 equiv), Cu(OAc)_2_ (10 mol %), DCE (2 mL), 80 °C, 3 hours. TEMPO = 2,2,6,6-tetramethylpiperidine-1-oxyl; BHT = butylated hydroxytoluene.

Based on the literature known results and the experimental evidences [35–36], we proposed a plausible reaction mechanism (Scheme 2b). The reaction started with the formation of radical intermediate **A** from diaryliodonium salt **2a**. Naphthol **1a** forms intermediate **B** with **A** after participation with the Cu(II) catalyst. Intermediate **B** generates **C** by radical substitution. A final intramolecular transesterification yields the benzocoumarin product **3aa**.

## Conclusion

In summary, we have employed *ortho*-ester-substituted diaryliodonium salts in a cascade cyclization, the cyclization features a copper-catalyzed activation strategy involving the cleavage of the C–I bond and esterification. The resulting cascade of selective arylation/intramolecular cyclization facilitated the synthesis of 3,4-benzocoumarin derivatives. The protocol enables the efficient formation of two chemical bonds in one pot, representing a valuable tool for the synthesis of polycyclic benzocoumarins. Our ongoing research endeavours are dedicated to explore the detailed reaction mechanism with the ultimate aim of broadening the scope and applicability of this approach.

## Supporting Information

File 1 Experimental procedures, LC–MS spectra and characterization data of all products, copies of ^1^H, ^13^C, ^19^F NMR spectra of all compounds.

## Data Availability

The data that supports the findings of this study is available from the corresponding author upon reasonable request.
