# Supplementary material for: Ortho-ester-substituted diaryliodonium salts enabled regioselective arylocyclization of naphthols toward 3,4-benzocoumarins
Source: Beilstein J Org Chem. 2024 Apr 18;20:841–51. doi: 10.3762/bjoc.20.76 (PMC11035988; doi:10.3762/bjoc.20.76)
Supplement: File 1 — Experimental procedures, LC–MS spectra and characterization data of all products, copies of 1H, 13C, 19F NMR spectra of all compounds. [file Beilstein_J_Org_Chem-20-841-s001.pdf]

BEILSTEIN JOURNAL OF ORGANIC CHEMISTRY

Supporting Information

for

Ortho-ester-substituted diaryliodonium salts enabled regioselective arylocyclization of naphthols toward 3,4-benzocoumarins

Ke Jiang, Cheng Pan, Limin Wang, Hao-Yang Wang and Jianwei Han

Beilstein J. Org. Chem. **2024,** *20,* 841–851. doi:10.3762/bjoc.20.76

Experimental procedures, LC–MS spectra and characterization data of all products, copies of ^{1}H, ^{13}C, ^{19}F NMR spectra of all compounds

License and Terms: This is a supporting information file under the terms of the Creative Commons Attribution License (https://creativecommons.org/licenses/by/4.0). Please note that the reuse, redistribution and reproduction in particular requires that the author(s) and source are credited and that individual graphics may be subject to special legal provisions.
The license is subject to the *Beilstein Journal of Organic Chemistry* terms and conditions: (https://www.beilstein-journals.org/bjoc/terms)

Table of Contents

Part 1. General information S2
Part 2. Synthesis and characterization of *ortho*-ester-substituted diaryliodonium salts S3
Part 3. Table of recation condition optimization S8
Part 4. Synthesis and characterization of 3,4-benzocoumarins S9
Part 5. Mechanism study S25
Part 6. References S26
Part 7. ^{1}H, ^{13}C, and ^{19}F NMR spectra of products S27

Part 1. General information

a. Methods:

NMR spectrum: ^1H, ^{13}C, and ^{19}F NMR spectra were recorded in $CDCl_3$ or DMSO-d_6 (with tetramethylsilane as an internal standard) on a Bruker AVANCE 400 spectrometer at ambient temperature, operating at 400 MHz, 101 MHz, and 376 MHz, respectively. Data were reported as follows: Chemical shifts (δ) are reported in ppm, and coupling constants (J) are in Hertz (Hz). The following abbreviations were used to explain the multiplicities: s = singlet, d = doublet, t = triplet, q = quartet, m = multiplet, br = broad.

Mass spectroscopy: Mass spectra were in general recorded on a Waters LCT Premier XE spectrometer or EI Mass spectra were measured on HP HP5989A, Aglient HP5873 or Waters Micromass GCT mass spectrometer. ESI-MS analyses were performed in positive ionization mode on an Agilent 1100-MSD or Bruker Daltonics FTMS-7 mass spectrometer.

Chromatography: Column chromatography was performed with silica gel (200-300 mesh ASTM).

b. Materials:

All solvents were purchased from Adamas-beta and dried and/or distilled by standard methods. All reagents were purchased from commercial sources (Adamas-beta; Sinopharma reagents; TCI; Acros) and used without further purification. Reactions were monitored by TLC (detection with UV light). The preparation of all other materials is described in detail below.

Part 2. Synthesis and characterization of *ortho*-ester-substituted diaryliodonium salts

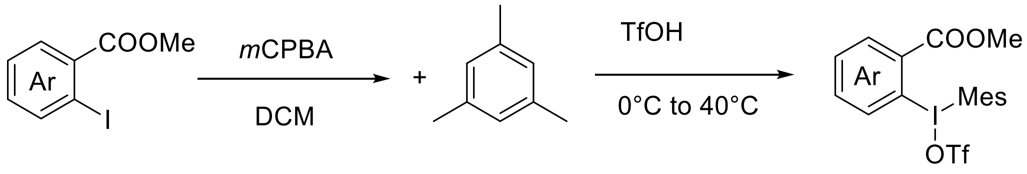

2

General procedure 1: To a solution of methyl 2-iodobenzoate (10 mmol) in CH_2Cl_2 (40 mL) was slowly added *m*CPBA (2.5 g, 11mmol) at room temperature. After stirring for 5 minutes, mesitylene (2.0ml,12mmol) was added. Then trifluoromethanesulfonic acid (1.2 mL,15 mmol) was added dropwise over 2 minutes at 0 °C and the solution was stirred at 4 0°C for 2–3 hours until TLC indicated that the methyl 2-iodobenzoate was completely consumed. The solvent was removed in vacuo and Et_2O (50 mL) was added in the mixture. The mixture was stirred vigorously until the solid precipitated. The solids were washed with Et_2O (2 × 10 mL) and filtrated under reduced pressure to give the *ortho*-ester-substituted diaryliodonium Salts (**2a–j**) in a pure form [1].

Methyl 2-(mesityl(((trifluoromethyl)sulfonyl)oxy)-λ³-iodaneyl)benzoate (2a)

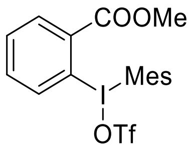

Prepared according to the ***General procedure 1*** on 10 mmol scale. The desired product of *ortho*-ester-substituted diaryliodonium salts (**2a**) was obtained as white solid (4.24 g, 81%).

^{1}H NMR (400 MHz, DMSO-*d₆*) δ 8.34 (d, *J* = 7.3 Hz, 1H), 7.83 – 7.73 (m, 2H), 7.42 (s, 2H), 6.90 (d, *J* = 7.7 Hz, 1H), 4.07 (s, 3H), 2.53 (s, 6H), 2.43 (s, 3H).

Analytical data are consistent with the reported ones [2].

Ethyl 2-(mesityl(((trifluoromethyl)sulfonyl)oxy)- λ³-iodaneyl)benzoate (2b)

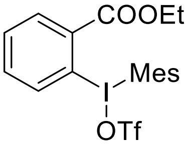

Prepared according to the ***General procedure 1*** on 1 mmol scale. The desired product of *ortho*-ester-substituted diaryliodonium salts (**2b**) was obtained as white solid (462 mg, 85%).

M.P.: 186-189 °C.

^{1}H NMR (400 MHz, DMSO-d_6) δ 8.33 (dd, *J* = 7.5, 1.8 Hz, 1H), 7.80 (d, *J* = 1.0 Hz, 2H), 7.41 (s, 2H), 6.87 (dd, *J* = 8.0, 0.9 Hz, 1H), 4.52 (d, *J* = 7.1 Hz, 2H), 3.36(water, the same as below), 2.51 (s, 6H), 2.42 (s, 3H), 1.42 (t, *J* = 7.1 Hz, 3H).

^{13}C NMR (101 MHz, DMSO-d_6) δ 167.44 (s), 144.59 (s), 143.30 (s), 137.37 (s), 132.97 (s), 131.43 (s), 130.17 (s), 128.57 (s), 127.65 (s), 120.72(q, $J_{C\text{-}F}$ = 323.2Hz), 117.87 (s), 113.65 (s), 63.82 (s), 26.13 (s), 20.83 (s), 13.95 (s).

^{19}F NMR (376 MHz, DMSO-*d$_6$*) δ -77.77.
The peak at 3.36 ppm is the water peak from DMSO-d6, which cannot be removed, and is similar to the following
HRMS m/z (ESI-TOF): calculated for $C_{18}H_{20}O_2I^+$ [M-OTf]$^+$ 395.0508, found 395.0486.

Methyl 2-(mesityl(((trifluoromethyl)sulfonyl)oxy)- λ^3-iodaneyl)-6-methylbenzoate (2c)

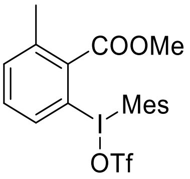

Prepared according to the ***General procedure 1*** on 1 mmol scale. The desired product of *ortho*-ester-substituted diaryliodonium salts (**2c**) was obtained as white solid (450 mg, 80%).
M.P.: 175-178 °C.
^{1}H NMR (400 MHz, DMSO-*d$_6$*) δ 7.65 (d, *J* = 7.5 Hz, 1H), 7.56 (t, *J* = 7.9 Hz, 1H), 7.37 (s, 2H), 6.92 (d, *J* = 8.0 Hz, 1H), 4.04 (s, 3H), 2.64 (s, 3H), 2.51 (s, 6H), 2.40 (s, 3H).
^{13}C NMR (101 MHz, DMSO-*d$_6$*) δ 169.41, 144.37, 144.35, 143.05, 135.65, 135.03, 130.07, 127.70, 127.63, 120.69(q, $J_{C\text{-}F}$ = 323.2Hz), 119.38, 115.59, 54.32, 39.52, 26.04, 22.90, 20.75.
^{19}F NMR (376 MHz, DMSO-*d$_6$*) δ -77.77.
HRMS m/z (ESI-TOF): calculated for $C_{18}H_{20}O_2I^+$ [M-OTf]$^+$ 395.0508, found 395.0489.

Methyl 4-chloro-2-(mesityl(((trifluoromethyl)sulfonyl)oxy)- λ^3-iodaneyl)benzoate (2d)

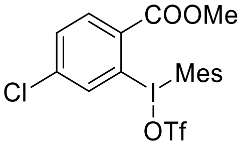

Prepared according to the ***General procedure 1*** on 1 mmol scale. The desired product of *ortho*-ester-substituted diaryliodonium salts (**2d**) was obtained as white solid (423 mg, 75%).
M.P.: 157-160 °C.
^{1}H NMR (400 MHz, DMSO-*d$_6$*) δ 8.29 (d, *J* = 8.4 Hz, 1H), 7.90 (dd, *J* = 8.3, 1.4 Hz, 1H), 7.43 (s, 2H), 6.64 (d, *J* = 1.5 Hz, 1H), 4.06 (s, 3H), 2.54 (s, 6H), 2.44 (s, 3H).
^{13}C NMR (101 MHz, DMSO-*d$_6$*) δ 167.34, 144.96, 143.32, 141.45, 133.88, 131.58, 130.28, 127.91, 126.88, 120.69(q, $J_{C\text{-}F}$ = 323.2Hz), 118.34, 115.40, 54.63, 39.52, 26.09, 20.82.
^{19}F NMR (376 MHz, DMSO-*d$_6$*) δ -77.76.
HRMS m/z (ESI-TOF): calculated for $C_{17}H_{17}ClO_2I^+$ [M-OTf]$^+$ 414.9962, found 414.9986.

Methyl 2-(mesityl(((trifluoromethyl)sulfonyl)oxy)- λ^3-iodaneyl)-3-methylbenzoate (2e)

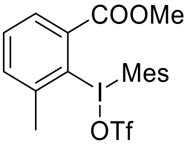

Prepared according to the ***General procedure 1*** on 1 mmol scale. The desired product of *ortho*-ester-substituted diaryliodonium salts (**2e**) was obtained as white solid (425 mg, 78%).
M.P.: 159-162 °C.
^{1}H NMR (400 MHz, DMSO-*d6*) δ 7.82 (dd, *J* = 7.5, 1.4 Hz, 1H), 7.77 (d, *J* = 6.3 Hz, 1H), 7.69 (t, *J* = 7.6 Hz, 1H), 7.18 (s, 2H), 3.77 (s, 3H), 2.53 (s, 3H), 2.39 (s, 6H), 2.29 (s, 3H).
^{13}C NMR (101 MHz, DMSO-*d6*) δ 166.19, 143.54, 143.00, 142.11, 135.95, 133.21, 132.44, 130.20, 129.85, 120.69(q, $J_{C\text{-}F}$ = 324.2Hz), 120.62, 119.09, 117.44, 53.38, 39.52, 25.37, 25.23, 20.38.
^{19}F NMR (376 MHz, DMSO-*d6*) δ -77.77.
HRMS m/z (ESI-TOF): calculated for $C_{18}H_{20}O_2I^+$ $[M\text{-}OTf]^+$ 395.0508, found 395.0488.

Methyl 2-(mesityl(((trifluoromethyl)sulfonyl)oxy)-λ^3-iodaneyl)-4-(trifluoromethyl)benzoate (2f)

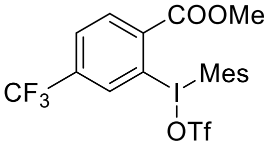

Prepared according to the ***General procedure 1*** on 1 mmol scale. The desired product of *ortho*-ester-substituted diaryliodonium salts (**2f**) was obtained as white solid (425 mg, 71%).
M.P.: 175-178 °C.
^{1}H NMR (400 MHz, DMSO-*d6*) δ 8.48 (d, *J* = 8.0 Hz, 1H), 8.20 (d, *J* = 8.1 Hz, 1H), 7.45 (s, 2H), 6.89 (s, 1H), 4.10 (s, 3H), 2.53 (s, 6H), 2.44 (s, 3H).
^{13}C NMR (101 MHz, DMSO-*d6*) δ 167.03, 145.05, 143.30, 135.41, 135.08, 133.59, 131.87, 130.20, 126.71(dd, $J_{C\text{-}F}$ = 394.9, 4.0Hz), 122.29(q, $J_{C\text{-}F}$ = 274.7Hz), 120.68(q, $J_{C\text{-}F}$ = 323.2Hz), 118.44, 115.14, 54.86, 39.52, 26.00, 20.76.
^{19}F NMR (376 MHz, DMSO-*d6*) δ -62.28, -77.80.
HRMS m/z (ESI-TOF): calculated for $C_{18}H_{17}F_3O_2I^+$ $[M\text{-}OTf]^+$ 449.0225, found 449.0206.

Methyl 4-bromo-2-(mesityl(((trifluoromethyl)sulfonyl)oxy)- λ^3-iodaneyl)benzoate (2g)

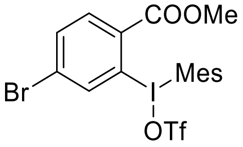

Prepared according to the ***General procedure 1*** on 1 mmol scale. The desired product of *ortho*-ester-substituted diaryliodonium salts (**2g**) was obtained as white solid (487 mg, 80%).
M.P.: 187-190 °C.
^{1}H NMR (400 MHz, DMSO-*d6*) δ 8.20 (d, *J* = 8.3 Hz, 1H), 8.03 (d, *J* = 1.7 Hz, 1H), 7.44 (s, 2H), 6.77 (d, *J* = 1.7 Hz, 1H), 4.05 (s, 3H), 2.53 (s, 6H), 2.44 (s, 3H).
^{13}C NMR (101 MHz, DMSO-*d6*) δ 167.49, 144.94, 143.30, 134.50, 134.00, 130.63, 130.49, 130.26, 127.16, 120.69(q, $J_{C\text{-}F}$ = 324.2Hz), 118.32, 115.47, 54.62, 39.52, 26.08, 20.82.
^{19}F NMR (376 MHz, DMSO-*d6*) δ -77.76.
HRMS m/z (ESI-TOF): calculated for $C_{17}H_{17}BrO_2I^+$ $[M\text{-}OTf]^+$ 458.9457, found 458.9435.

Methyl 2-(mesityl(((trifluoromethyl)sulfonyl)oxy)- λ^3-iodaneyl)-4,5-dimethoxybenzoate (2h)

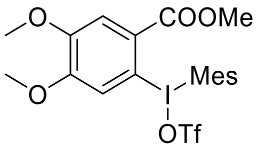

Prepared according to the ***General procedure 1*** on 1 mmol scale. The desired product of *ortho*-ester-substituted diaryliodonium salts (**2h**) was obtained as white solid (442 mg, 75%).

M.P.: 190-193 °C.

^{1}H NMR (400 MHz, DMSO-*d$_6$*) δ 7.73 (s, 1H), 7.41 (s, 2H), 6.11 (s, 1H), 4.04 (s, 3H), 3.88 (s, 3H), 2.52 (d, *J* = 7.5 Hz, 6H), 2.41 (s, 3H).

^{13}C NMR (101 MHz, DMSO-*d$_6$*) δ 167.77, 155.12, 150.35, 144.65, 143.22, 129.97, 120.69(q, J_{C-F} = 324.2Hz), 119.74, 118.50, 114.04, 110.35, 104.53, 56.30, 55.56, 54.30, 39.52, 26.12, 20.75.

^{19}F NMR (376 MHz, DMSO-*d$_6$*) δ -77.77.

HRMS m/z (ESI-TOF): calculated for $C_{19}H_{22}O_4I^+$ $[M\text{-}OTf]^+$ 441.0563, found 441.0541.

Methyl 5-fluoro-2-(mesityl(((trifluoromethyl)sulfonyl)oxy)- λ^3-iodaneyl)benzoate (2i)

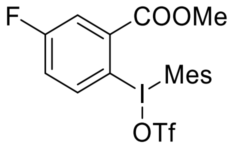

Prepared according to the ***General procedure 1*** on 1 mmol scale. The desired product of *ortho*-ester-substituted diaryliodonium salts (**2i**) was obtained as white solid (438 mg, 80%).

M.P.: 186-189 °C.

^{1}H NMR (400 MHz, CDCl$_3$) δ 8.03 (dd, *J* = 8.1, 2.9 Hz, 1H), 7.30 (ddd, *J* = 10.0, 7.3, 2.9 Hz, 1H), 7.20 (s, 2H), 6.79 (dd, *J* = 9.0, 4.6 Hz, 1H), 4.13 (s, 3H), 2.55 (s, 6H), 2.42 (s, 3H).

^{13}C NMR (101 MHz, CDCl$_3$) δ 166.81, 162.99, 145.57, 143.91, 130.61(d, J_{C-F} = 8.1Hz), 129.56(d, J_{C-F} = 8.1Hz), 124.40(d, J_{C-F} = 23.2Hz), 120.79(d, J_{C-F} = 23.2Hz), 120.42(q, J_{C-F} = 321.2Hz), 117.53(d, J_{C-F} = 263.6Hz), 117.40, 116.22, 105.91(d, J_{C-F} = 3.0Hz), 77.16, 55.21, 26.94, 26.84, 21.43.

^{19}F NMR (376 MHz, CDCl$_3$) δ -77.23, -109.22.

HRMS m/z (ESI-TOF): calculated for $C_{17}H_{17}FO_2I^+$ $[M\text{-}OTF]^+$ 399.0257, found 399.0240.

Methyl 2-(mesityl(((trifluoromethyl)sulfonyl)oxy)-λ^3-iodaneyl)-5-methylbenzoate (2j)

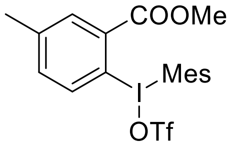

Prepared according to the ***General procedure 1*** on 1 mmol scale. The desired product of *ortho*-ester-substituted diaryliodonium salts (**2j**) was obtained as white solid (478 mg, 88%).

M.P.: 176-179 °C.

^{1}H NMR (400 MHz, DMSO-*d$_6$*) δ 8.76 (d, *J* = 1.6 Hz, 1H), 8.04 (dd, *J* = 8.5, 1.6 Hz, 1H), 7.90 (s, 2H), 7.34 (d, *J* = 8.5 Hz, 1H), 4.65 (s, 3H), 3.06 (s, 6H), 3.00 (s, 6H).

^{13}C NMR (101 MHz, DMSO-*d$_6$*) δ 167.93, 144.51, 143.18, 141.86, 137.91, 133.36, 130.12, 128.43, 127.33, 120.68(q, $J_{C\text{-}F}$ = 324.2Hz), 117.83, 109.87, 54.38, 39.52, 26.09, 20.78, 20.09.

^{19}F NMR (377 MHz, DMSO-*d$_6$*) δ -77.76.

HRMS m/z (ESI-TOF): calculated for $C_{18}H_{20}O_2I^+$ [M-OTF]$^+$ 395.0508, found 395.0496.

Methyl 5-chloro-2-(mesityl(((trifluoromethyl)sulfonyl)oxy)- λ^3-iodaneyl)benzoate (2k)

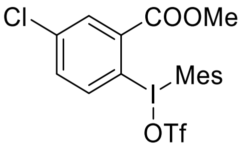

Prepared according to the ***General procedure 1*** on 1 mmol scale. The desired product of *ortho*-ester-substituted diaryliodonium salts (**2k**) was obtained as white solid (456 mg, 81%).

M.P.: 179-182℃.

^{1}H NMR (400 MHz, CDCl$_3$) δ 8.29 (d, *J* = 2.4 Hz, 1H), 7.51 (dd, *J* = 8.7, 2.4 Hz, 1H), 7.20 (s, 2H), 6.74 (d, *J* = 8.7 Hz, 1H), 4.14 (s, 3H), 2.55 (s, 6H), 2.43 (s, 3H).

^{13}C NMR (101 MHz, DMSO-*d$_6$*) δ 166.87, 144.73, 143.27, 136.66, 136.42, 132.15, 130.40, 130.23, 129.58, 120.68(q, $J_{C\text{-}F}$ = 324.2Hz), 118.06, 111.93, 54.66, 39.52, 26.11, 20.78.

^{19}F NMR (377 MHz, DMSO-*d$_6$*) δ -77.76.

HRMS m/z (ESI-TOF): calculated for $C_{17}H_{17}ClO_2I^+$ [M-OTF]$^+$ 414.9962, found 414.9946.

Methyl 5-bromo-2-(mesityl(((trifluoromethyl)sulfonyl)oxy)- λ^3-iodaneyl)benzoate (2l)

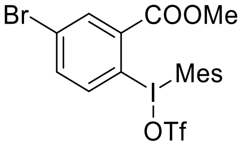

Prepared according to the ***General procedure 1*** on 1 mmol scale. The desired product of *ortho*-ester-substituted diaryliodonium salts (**2l**) was obtained as white solid (510 mg, 84%).

M.P.: 170-173℃.

^{1}H NMR (400 MHz, CDCl$_3$) δ 8.43 (d, *J* = 2.3 Hz, 1H), 7.65 (dd, *J* = 8.7, 2.3 Hz, 1H), 7.20 (s, 2H), 6.66 (d, *J* = 8.7 Hz, 1H), 4.14 (s, 3H), 2.55 (s, 6H), 2.43 (s, 3H).

^{13}C NMR (101 MHz, DMSO-*d$_6$*) δ 166.84, 144.76, 143.31, 139.62, 135.00, 130.53, 130.26, 129.68, 124.79, 120.70(q, $J_{C\text{-}F}$ = 324.2Hz), 118.01, 112.75, 54.69, 39.52, 26.14, 20.82.

^{19}F NMR (377 MHz, DMSO-*d$_6$*) δ -77.76.

HRMS m/z (ESI-TOF): calculated for $C_{17}H_{17}BrO_2I^+$ [M-OTF]$^+$ 458.9457, found 458.9445.

Methyl 2-fluoro-6-(mesityl(((trifluoromethyl)sulfonyl)oxy)- λ^3-iodaneyl)benzoate (2m)

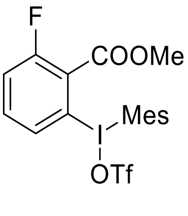

Prepared according to the ***General procedure 1*** on 1 mmol scale. The desired product of *ortho*-ester-substituted diaryliodonium salts (**2m**) was obtained as white solid (427 mg, 78%).
M.P.: 140-143℃.
^{1}H NMR (400 MHz, DMSO-*d$_6$*) δ 7.78 – 7.67 (m, 2H), 7.39 (s, 2H), 6.79 (d, J = 7.3 Hz, 1H), 4.06 (s, 3H), 2.52 (s, 6H), 2.41 (s, 3H).
^{13}C NMR (101 MHz, DMSO-*d$_6$*) δ 166.78, 164.33, 161.69, 144.68, 143.23, 138.30, 138.20, 130.19, 125.39(d, $J_{C\text{-}F}$ = 3.0Hz), 120.71(q, $J_{C\text{-}F}$ = 322.2Hz), 120.36(d, $J_{C\text{-}F}$ = 24.2Hz), 116.88(d, $J_{C\text{-}F}$ = 7.1Hz), 116.04, 54.79, 39.52, 26.08, 20.80.
^{19}F NMR (376 MHz, DMSO-*d$_6$*) δ -77.77, -100.41.
HRMS m/z (ESI-TOF): calculated for $C_{17}H_{17}FO_2I^+$ [M-OTF]$^+$ 399.0257, found 399.0240.

Part 3. Table of recation condition optimization

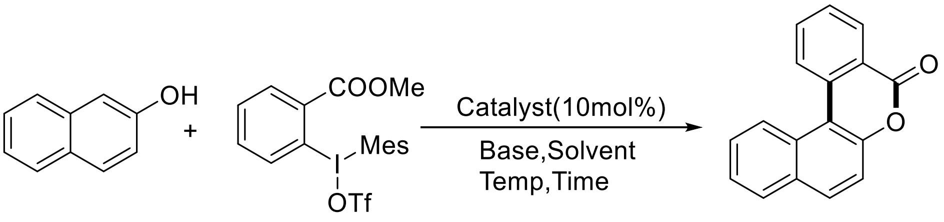

1a **2a** **3aa**

Table S1: Base optimization

Entry	Base	Yield
1	K_2CO_3	27%
2	Na_2CO_3	25%
3	Cs_2CO_3	16%
4	KOH	24%
5	KH_2PO_4	10%
6	/	50%
7	DMAP	26%
8	NaO*t*-Bu	35%
9	LiHMDS	30%
10	NaHMDS	20%
11	HNa	0%

Reaction conditions unless specifically noted: **1a** (43.2 mg, 0.3 mmol, 1.0 equiv), **2a** (174.9 mg, 0.33 mmol, 1.1 equiv), base (0.3 mmol, 1.0 equiv), and catalyst $Cu(OTf)_2$ (10.8 mg, 0.03 mmol, 10 mol %) in solvent DCE (2 mL) at 80 °C for 3 h. Isolated yields were obtained after purification with column chromatography.

Table S2: Solvent optimization

Entry	Solvent	Yield
1	DCE	50%
2	DMSO	45%
3	DMF	23%
4	NMP	0%
5	Toluene	10%

6	AcOH	0%
7	MeCN	36%
8	DMA	35%
9	EA	17%
10	H_2O	0%
11	Ethanol	28%
12	DCE/H_2O (1/1)	0%
13	Cyclohexane	30%
14	Cyclohexane/ Ethyl ether (1/1)	0%
15	DMSO (110 °C)	40%
16	DMSO (130 °C)	37%
17	DCE (12 h)	48%

Reaction conditions unless specifically noted: **1a** (43.2 mg, 0.3 mmol, 1.0 equiv), **2a** (174.9 mg, 0.33 mmol, 1.1 equiv), and catalyst $Cu(OTf)_2$ (10.8 mg, 0.03 mmol, 10 mol %) in solvent (2 mL) at 80 °C for 3 h. Isolated yields were obtained after purification with column chromatography.

Table S3: Catalyst optimization

Entry	Catalyst	Yield
1	$Cu(OAc)_2$	61%
2[a]	$Cu(OTf)_2$	50%(48%)
3	$Pd(OAc)_2$	22%
4	$Cu(CF_3SO_3)_2$	32%
5	$Pd(PPh_3)_4$	20%
6	/	0%
7	$PdCl_2$	40%
8	$Pd(acac)_2$	24%
9	$FeCl_3$	21%
10	AgOAc	20%

Reaction conditions unless specifically noted: **1a** (43.2 mg, 0.3 mmol, 1.0 equiv), **2a** (174.9 mg, 0.33 mmol, 1.1 equiv), and catalyst (0.03 mmol, 10 mol %) in solvent DCE (2 mL) at 80 °C for 3 h. Isolated yields were obtained after purification with column chromatography. [a]The reaction time was 12 hours.

Part 4. Synthesis and characterization of 3,4-benzocoumarins

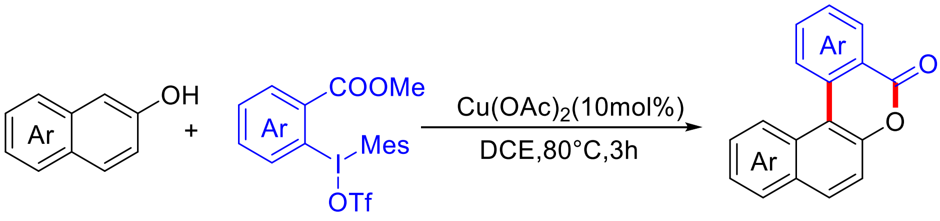

1 **2** **3**

General procedure 2: To an oven-dried Schlenk tube was added 2-Naphthol **1** (0.3 mmol, 1.0 equiv) *ortho*-ester-substituted diaryliodonium salt **2** (0.33 mmol, 1.1 equiv) $Cu(OAc)_2$ (0.03 mmol, 10% equiv) and DCE (2 mL). The mixture was stirred at 80 °C in an oil bath until TLC indicated that the 2-naphthol was completely consumed. The solvent was evaporated under vacuum. The crude products were purified using flash column chromatography (eluent: petroleum ether: EtOAc) on silica gel to afford the desired product **3**.

The compounds are difficult to purify by chromatography due to their high lipophilicity and the yields may deviate slightly. The impurity in the NMR spectra mainly arise from solvent impurities.

5*H*-Dibenzo[*c,f*]chromen-5-one (3aa)

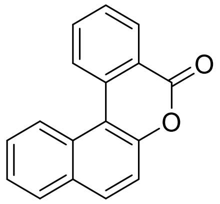

The preparation was according to the ***general procedure 2*** on 0.3 mmol scale, and obtained an isolated yield of 61% (45 mg) as yellow solid after column chromatography (petroleum ether: EtOAc = 8:1) on silica gel.

^{1}H NMR (400 MHz, CDCl$_3$) δ 8.78 (d, *J* = 8.6 Hz, 1H), 8.66 (d, *J* = 8.3 Hz, 1H), 8.51 (dd, *J* = 7.9, 1.3 Hz, 1H), 7.99 – 7.86 (m, 3H), 7.72 – 7.61 (m, 2H), 7.56 (ddd, *J* = 8.0, 6.9, 1.0 Hz, 1H), 7.49 (d, *J* = 8.9 Hz, 1H).

Analytical data are consistent with the reported ones [3].

The peak at 1.58 ppm is the water peak from $CDCl_3$, which cannot be removed, and is similar to the following

11-Bromo-5*H*-dibenzo[*c,f*]chromen-5-one (3ab)

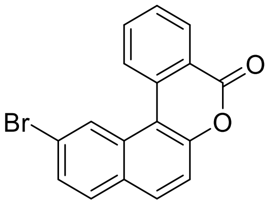

The preparation was according to the ***general procedure 2*** on 0.3 mmol scale, and obtained an isolated yield of 63% (61 mg) as yellow solid after column chromatography (petroleum ether: EtOAc = 8:1) on silica gel.

M.P.: 131-134℃.

^{1}H NMR (400 MHz, CDCl$_3$)δ 8.50 (dd, *J* = 7.9, 1.1 Hz, 1H), 8.42 (dd, *J* = 8.0, 1.5 Hz, 1H), 7.97 – 7.91 (m, 1H), 7.88 (d, *J* = 8.9 Hz, 1H), 7.80 (d, *J* = 8.7 Hz, 1H), 7.59 – 7.56 (m, 2H), 7.51 – 7.46 (m, 2H).

^{13}C NMR (101 MHz, CDCl$_3$) δ 161.08, 150.82, 134.87, 134.83, 131.40, 130.96, 130.87, 130.71, 130.13, 128.89, 128.66, 127.46, 126.10, 122.73, 122.37, 118.18, 111.98, 77.16.

HRMS m/z (EI-TOF): calculated for $C_{17}H_9BrO_2$ $[M]^+$ 323.9786, found 323.9787.

11-Methyl-5*H*-dibenzo[*c,f*]chromen-5-one (3ac)

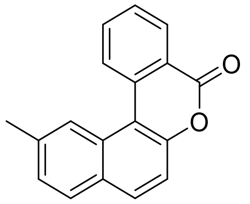

The preparation was according to the ***general procedure 2*** on 0.3 mmol scale, and obtained an isolated yield of 80% (62 mg) as yellow solid after column chromatography (petroleum ether: EtOAc = 8:1) on silica gel.

M.P.: 133-136℃.

^{1}H NMR (400 MHz, CDCl$_3$) δ 8.66 (d, *J* = 8.3 Hz, 1H), 8.54 (s, 1H), 8.50 (dd, *J* = 7.9, 1.2 Hz, 1H), 7.88 (ddd, *J* = 20.2, 9.4, 4.9 Hz, 3H), 7.66 – 7.59 (m, 1H), 7.40 (dd, *J* = 13.0, 5.0 Hz, 2H), 2.62 (s, 3H).

^{13}C NMR (101 MHz, CDCl$_3$) δ 161.57, 150.56, 137.97, 135.70, 134.43, 131.42, 130.80, 129.94, 129.92, 129.29, 128.15, 127.56, 126.47, 124.47, 122.40, 116.73, 112.15, 77.16, 22.51.

HRMS m/z (EI-TOF): calculated for $C_{18}H_{12}O_2$ $[M]^+$ 260.0837, found 260.0840.

11-Methoxy-5*H*-dibenzo[*c,f*]chromen-5-one (3ad)

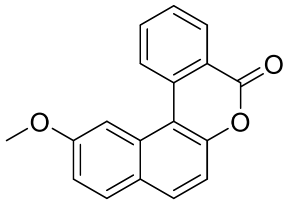

The preparation was according to the ***general procedure 2*** on 0.3 mmol scale, and obtained an isolated yield of 77% (64 mg) as yellow solid after column chromatography (petroleum ether: EtOAc = 8:1) on silica gel.

M.P.: 129-132℃.

^{1}H NMR (400 MHz, CDCl$_3$) δ 8.69 (d, *J* = 8.3 Hz, 1H), 8.51 (dd, *J* = 7.9, 1.2 Hz, 1H), 8.15 (d, *J* = 2.2 Hz, 1H), 7.92 – 7.83 (m, 3H), 7.65 – 7.59 (m, 1H), 7.35 (d, *J* = 8.8 Hz, 1H), 7.23 (dd, *J* = 8.9, 2.4 Hz, 1H), 4.00 (s, 3H).

^{13}C NMR (101 MHz, CDCl$_3$) δ 161.56, 159.63, 151.14, 135.89, 134.47, 131.46, 131.14, 131.01, 130.96, 128.10, 127.00, 125.71, 122.39, 116.59, 115.29, 111.86, 105.95, 77.16, 55.69.

HRMS m/z (EI-TOF): calculated for $C_{18}H_{12}O_3$ $[M]^+$ 276.0786, found 276.0791.

11-Phenyl-5*H*-dibenzo[*c,f*]chromen-5-one (3ae)

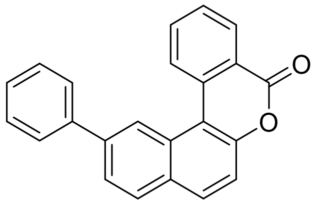

The preparation was according to the ***general procedure 2*** on 0.3 mmol scale, and obtained an isolated yield of 26% (26 mg) as yellow solid after column chromatography (petroleum ether: EtOAc = 8:1) on silica gel.

M.P.: 139-142℃.

^{1}H NMR (400 MHz, CDCl$_3$) δ 8.96 – 8.94 (m, 1H), 8.86 (d, *J* = 0.6 Hz, 1H), 8.62 (d, *J* = 8.3 Hz, 1H), 8.49 (dd, *J* = 7.9, 1.3 Hz, 1H), 7.97 (d, *J* = 8.4 Hz, 1H), 7.91 – 7.83 (m, 2H), 7.76 (dd, *J* = 8.4, 1.6 Hz, 1H), 7.71 (dd, *J* = 5.2, 3.4 Hz, 2H), 7.64 – 7.58 (m, 1H), 7.53 (dd, *J* = 10.3, 4.8 Hz, 2H), 7.44 (dt, *J* = 7.3, 2.9 Hz, 2H).

^{13}C NMR (101 MHz, CDCl$_3$) δ 161.35, 150.67, 141.06, 140.69, 135.45, 134.54, 131.29, 130.85, 130.77, 129.94, 129.87, 129.19, 128.29, 127.93, 127.67, 126.37, 125.16, 123.31, 122.43, 117.63, 112.83, 77.16.

HRMS m/z (EI-TOF): calculated for $C_{23}H_{14}O_2$ $[M]^+$ 322.0994, found 322.0990.

10-Methyl-5*H*-dibenzo[*c,f*]chromen-5-one (3af)

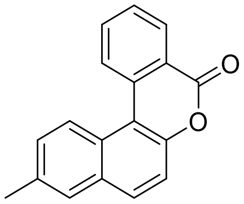

The preparation was according to the ***general procedure 2*** on 0.3 mmol scale, and obtained an isolated yield of 31% (25 mg) as yellow solid after column chromatography (petroleum ether: EtOAc = 8:1) on silica gel.

M.P.:141-144°C.

^{1}H NMR (400 MHz, CDCl$_3$) δ 8.67 – 8.59 (m, 2H), 8.49 (dd, *J* = 7.9, 1.2 Hz, 1H), 7.90 – 7.85 (m, 1H), 7.82 (d, *J* = 8.9 Hz, 1H), 7.70 (s, 1H), 7.64 – 7.59 (m, 1H), 7.48 (dd, *J* = 8.8, 1.7 Hz, 1H), 7.44 (d, *J* = 8.9 Hz, 1H), 2.55 (s, 3H).

^{13}C NMR (101 MHz, CDCl$_3$) δ 161.56, 149.91, 135.65, 135.23, 134.43, 132.04, 131.11, 130.81, 130.01, 128.62, 128.21, 127.69, 126.47, 124.97, 122.44, 117.67, 112.60, 77.16, 21.35.

HRMS m/z (EI-TOF): calculated for $C_{18}H_{12}O_2$ [M]$^+$ 260.0837, found 260.0841.

5-Oxo-5*H*-dibenzo[*c,f*]chromene-10-carbaldehyde (3ag)

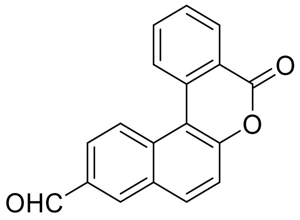

The preparation was according to the ***general procedure 2*** on 0.3 mmol scale, and obtained an isolated yield of 28% (23 mg) as yellow solid after column chromatography (petroleum ether: EtOAc = 8:1) on silica gel.

M.P.:145-148°C.

^{1}H NMR (400 MHz, CDCl$_3$) δ 10.22 (s, 1H), 8.89 (d, *J* = 8.9 Hz, 1H), 8.62 (d, *J* = 8.2 Hz, 1H), 8.53 (d, *J* = 7.8 Hz, 1H), 8.46 (s, 1H), 8.15 (dd, *J* = 8.9, 1.5 Hz, 1H), 8.10 (d, *J* = 8.8 Hz, 1H), 7.95 (t, *J* = 7.7 Hz, 1H), 7.70 (t, *J* = 7.6 Hz, 1H), 7.62 (d, *J* = 8.8 Hz, 1H).

^{13}C NMR (101 MHz, CDCl$_3$) δ 191.66, 160.91, 152.23, 134.86, 134.83, 134.79, 133.50, 132.81, 131.16, 131.06, 129.01, 126.61, 126.26, 125.14, 122.60, 119.16, 113.43, 111.88, 77.16.

HRMS m/z (EI-TOF): calculated for $C_{18}H_{10}O_3$ [M]$^+$ 274.0630, found 274.0633.

10-Bromo-5*H*-dibenzo[*c,f*]chromen-5-one (3ah)

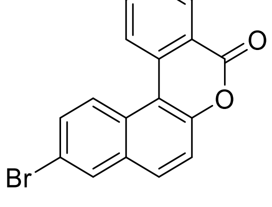

The preparation was according to the ***general procedure 2*** on 0.3 mmol scale, and obtained an isolated yield of 54% (52 mg) as yellow solid after column chromatography (petroleum ether: EtOAc = 8:1) on silica gel.
M.P.:201-204°C.
^{1}H NMR (400 MHz, CDCl$_3$) δ 8.61 (d, *J* = 9.2 Hz, 1H), 8.56 – 8.47 (m, 2H), 8.08 (d, *J* = 2.1 Hz, 1H), 7.93 – 7.87 (m, 1H), 7.81 (d, *J* = 8.9 Hz, 1H), 7.71 (dd, *J* = 9.2, 2.1 Hz, 1H), 7.68 – 7.63 (m, 1H), 7.50 (d, *J* = 8.9 Hz, 1H).
^{13}C NMR (101 MHz, CDCl$_3$) δ 161.13, 150.39, 134.99, 134.65, 133.02, 131.37, 131.02, 130.98, 130.55, 128.72, 128.20, 126.84, 126.35, 122.56, 119.36, 118.95, 113.01, 77.16.
HRMS m/z (EI-TOF): calculated for $C_{17}H_9BrO_2$ $[M]^+$ 323.9786, found 323.9790.

10-Phenyl-5*H*-dibenzo[*c,f*]chromen-5-one (3ai)

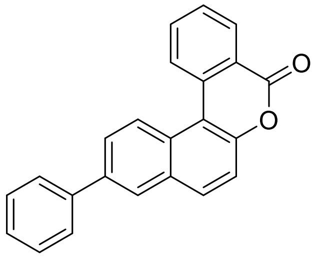

The preparation was according to the ***general procedure 2*** on 0.3 mmol scale, and obtained an isolated yield of 25% (24 mg) as yellow solid after column chromatography (petroleum ether: EtOAc = 8:1) on silica gel.
M.P.:149-152°C.
^{1}H NMR (400 MHz, CDCl$_3$) δ 8.82 (d, *J* = 8.9 Hz, 1H), 8.67 (d, *J* = 8.2 Hz, 1H), 8.51 (dd, *J* = 7.9, 1.2 Hz, 1H), 8.13 (d, *J* = 1.8 Hz, 1H), 7.97 (d, *J* = 8.9 Hz, 1H), 7.94 – 7.88 (m, 2H), 7.76 (d, *J* = 7.3 Hz, 2H), 7.65 (t, *J* = 7.5 Hz, 1H), 7.55 – 7.49 (m, 3H), 7.43 (t, *J* = 7.3 Hz, 1H).
^{13}C NMR (101 MHz, CDCl$_3$) δ 161.42, 150.42, 140.16, 138.15, 135.49, 134.55, 132.15, 131.91, 130.89, 129.17, 128.77, 128.41, 127.88, 127.38, 127.32, 127.10, 126.49, 125.72, 122.51, 118.17, 112.71, 77.16.
HRMS m/z (EI-TOF): calculated for $C_{23}H_{14}O_2$ $[M]^+$ 322.0994, found 322.0998.

7-Bromo-5*H*-dibenzo[*c,f*]chromen-5-one (3aj)

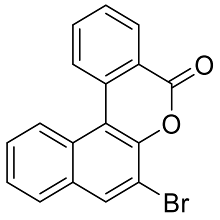

The preparation was according to the ***general procedure 2*** on 0.3 mmol scale, and obtained an isolated yield of 22% (20 mg) as yellow solid after column chromatography (petroleum ether: EtOAc = 8:1) on silica gel.
M.P.:154-157°C.

^{1}H NMR (400 MHz, CDCl$_3$) δ 8.71 (d, *J* = 8.6 Hz, 1H), 8.62 (d, *J* = 8.3 Hz, 1H), 8.52 (dd, *J* = 7.9, 1.2 Hz, 1H), 8.22 (s, 1H), 7.94 – 7.89 (m, 1H), 7.87 (d, *J* = 8.1 Hz, 1H), 7.67 (dd, *J* = 11.3, 4.1 Hz, 2H), 7.61 – 7.55 (m, 1H).

^{13}C NMR (101 MHz, CDCl$_3$) δ 160.46, 146.54, 135.02, 134.63, 134.19, 131.97, 130.92, 129.01, 128.72, 128.52, 128.08, 126.97, 126.35, 125.35, 122.54, 114.48, 111.04, 77.16.

HRMS m/z (EI-TOF): calculated for $C_{17}H_9BrO_2$ $[M]^+$ 323.9786, found 323.9790.

7-Methyl-5*H*-dibenzo[*c,f*]chromen-5-one (3ak)

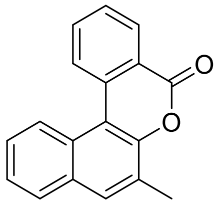

The preparation was according to the ***general procedure 2*** on 0.3 mmol scale, and obtained an isolated yield of 49% (38 mg) as yellow solid after column chromatography (petroleum ether: EtOAc = 8:1) on silica gel.

M.P.:127-130°C.

^{1}H NMR (400 MHz, CDCl$_3$) δ 8.67 (d, *J* = 8.5 Hz, 1H), 8.60 (d, *J* = 8.3 Hz, 1H), 8.49 (dd, *J* = 7.9, 1.2 Hz, 1H), 7.86 (ddd, *J* = 8.9, 5.3, 1.6 Hz, 2H), 7.73 (s, 1H), 7.59 (ddd, *J* = 10.0, 8.2, 1.2 Hz, 2H), 7.53 – 7.47 (m, 1H), 2.60 (d, *J* = 0.8 Hz, 3H).

^{13}C NMR (101 MHz, CDCl$_3$) δ 161.44, 149.61, 135.71, 134.32, 131.43, 131.36, 130.63, 128.58, 128.42, 128.16, 126.85, 126.72, 126.60, 125.44, 124.99, 122.25, 112.47, 77.16, 16.92.

HRMS m/z (EI-TOF): calculated for $C_{18}H_{12}O_2$ $[M]^+$ 260.0837, found 260.0845.

Methyl 5-oxo-5*H*-dibenzo[*c,f*]chromene-10-carboxylate (3al)

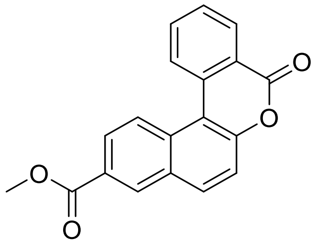

The preparation was according to the ***general procedure 2*** on 0.3 mmol scale, and obtained an isolated yield of 48% (44 mg) as yellow solid after column chromatography (petroleum ether: EtOAc = 8:1) on silica gel.

M.P.:150-153°C.

^{1}H NMR (400 MHz, CDCl$_3$) δ 8.81 (d, *J* = 9.0 Hz, 1H), 8.68 (d, *J* = 1.6 Hz, 1H), 8.62 (d, *J* = 8.3 Hz, 1H), 8.52 (dd, *J* = 7.9, 1.1 Hz, 1H), 8.24 (dd, *J* = 9.0, 1.8 Hz, 1H), 8.03 (d, *J* = 8.9 Hz, 1H), 7.96 – 7.90 (m, 1H), 7.67 (t, *J* = 7.6 Hz, 1H), 7.56 (d, *J* = 8.9 Hz, 1H), 4.02 (s, 3H).

^{13}C NMR (101 MHz, CDCl$_3$) δ 166.80, 161.07, 151.76, 135.06, 134.73, 132.74, 132.19, 132.07, 131.01, 130.98, 128.77, 127.36, 127.09, 126.51, 125.40, 122.51, 118.73, 112.98, 77.16, 52.56.

HRMS m/z (EI-TOF): calculated for $C_{19}H_{12}O_4$ $[M]^+$ 304.0736, found 304.0741.

Methyl 6-(2-(methoxycarbonyl)phenoxy)-2-naphthoate (3al')

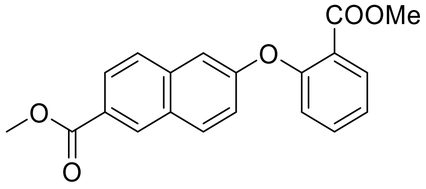

The preparation was according to the ***general procedure 2*** on 0.3 mmol scale, and obtained an isolated yield of 20% (20 mg) as yellow solid after column chromatography (petroleum ether: EtOAc = 8:1) on silica gel.

M.P.:125-128℃.

^{1}H NMR (400 MHz, CDCl$_3$) δ 8.56 (s, 1H), 8.00 (dd, *J* = 8.3, 1.5 Hz, 2H), 7.93 (d, *J* = 8.9 Hz, 1H), 7.66 (d, *J* = 8.7 Hz, 1H), 7.58 – 7.51 (m, 1H), 7.34 – 7.26 (m, 2H), 7.12 (dd, *J* = 6.0, 1.8 Hz, 2H), 3.96 (s, 3H), 3.75 (s, 3H).

^{13}C NMR (101 MHz, CDCl$_3$) δ 129.66, 128.21, 120.53, 117.54, 99.15, 96.37, 94.62, 93.87, 93.32, 91.25, 89.65, 88.52, 88.44, 87.05, 86.19, 84.69, 82.34, 74.16, 39.52, 14.67, 14.62.

HRMS m/z (EI-TOF): calculated for $C_{20}H_{16}O_5$ $[M]^+$ 336.0998, found 336.1002.

8-Bromo-5*H*-dibenzo[*c*,*f*]chromen-5-one (3am)

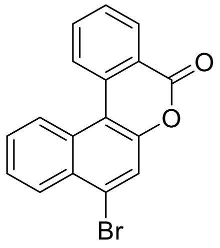

The preparation was according to the ***general procedure 2*** on 0.3 mmol scale, and obtained an isolated yield of 83% (81 mg) as yellow solid after column chromatography (petroleum ether: EtOAc = 8:1) on silica gel.

M.P.:151-154℃.

^{1}H NMR (400 MHz, CDCl$_3$) δ 8.75 (d, *J* = 8.5 Hz, 1H), 8.58 (d, *J* = 8.3 Hz, 1H), 8.50 (dd, *J* = 7.9, 1.1 Hz, 1H), 8.40 (dd, *J* = 8.3, 1.0 Hz, 1H), 7.93 – 7.86 (m, 1H), 7.84 (s, 1H), 7.72 (ddd, *J* = 8.5, 6.9, 1.4 Hz, 1H), 7.66 (dd, *J* = 8.0, 7.1 Hz, 2H).

^{13}C NMR (101 MHz, CDCl$_3$) δ 160.90, 149.37, 134.95, 134.67, 131.02, 130.30, 130.19, 128.69, 128.60, 126.76, 126.69, 125.68, 125.55, 122.53, 121.75, 112.76, 77.16.

HRMS m/z (EI-TOF): calculated for $C_{17}H_9BrO_2$ $[M]^+$ 323.9786, found 323.9795.

6*H*-Dibenzo[*c*,*h*]chromen-6-one (3an)

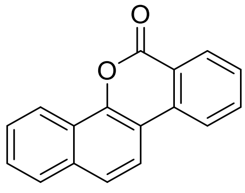

The preparation was according to the ***general procedure 2*** on 0.3 mmol scale, and obtained an isolated yield of 49% (36 mg) as yellow solid after column chromatography (petroleum ether: EtOAc = 8:1) on silica gel.
M.P.:171-174℃.
^{1}H NMR (400 MHz, $CDCl_3$) δ 8.57 (d, *J* = 7.9 Hz, 1H), 8.46 (d, *J* = 7.9 Hz, 1H), 8.18 (d, *J* = 8.1 Hz, 1H), 8.04 (d, *J* = 8.7 Hz, 1H), 7.86 (t, *J* = 7.2 Hz, 2H), 7.75 (d, *J* = 8.7 Hz, 1H), 7.65 – 7.58 (m, 3H).
^{13}C NMR (101 MHz, $CDCl_3$) δ 161.38, 147.35, 135.50, 135.10, 134.36, 130.76, 128.74, 128.01, 127.77, 127.24, 124.62, 123.98, 122.44, 122.15, 121.27, 119.27, 113.14, 77.16.
HRMS m/z (EI-TOF): calculated for $C_{17}H_{10}O_2$ $[M]^+$ 246.0681, found 246.0685.

2-Methoxy-6*H*-dibenzo[*c,h*]chromen-6-one (3ao)

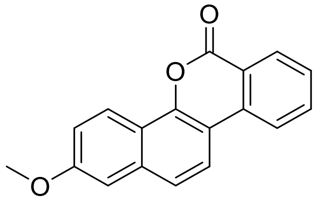

The preparation was according to the ***general procedure 2*** on 0.3 mmol scale, and obtained an isolated yield of 40% (33 mg) as yellow solid after column chromatography (petroleum ether: EtOAc = 8:1) on silica gel.
M.P.:155-158℃.
^{1}H NMR (400 MHz, $CDCl_3$) δ 8.49 – 8.40 (m, 2H), 8.13 (d, *J* = 8.0 Hz, 1H), 8.00 (d, *J* = 8.8 Hz, 1H), 7.87 – 7.80 (m, 1H), 7.63 (d, *J* = 8.7 Hz, 1H), 7.58 – 7.52 (m, 1H), 7.27 – 7.23 (m, 2H), 7.15 (d, *J* = 2.5 Hz, 1H), 3.95 (s, 3H).
^{13}C NMR (101 MHz, $CDCl_3$) δ 161.52, 159.41, 147.62, 136.08, 135.77, 135.04, 130.70, 128.23, 124.15, 123.51, 121.81, 120.78, 120.02, 119.43, 118.93, 111.36, 106.36, 77.16, 55.49.
HRMS m/z (EI-TOF): calculated for $C_{18}H_{12}O_3$ $[M]^+$ 276.0786, found 276.0790.

12-Bromo-5*H*-dibenzo[*c,f*]chromen-5-one (3ap)

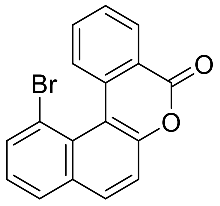

The preparation was according to the ***general procedure 2*** on 0.3 mmol scale, and obtained an isolated yield of 25% (24 mg) as yellow solid after column chromatography (petroleum ether: EtOAc = 8:1) on silica gel.
M.P.:152-155℃.
^{1}H NMR (400 MHz, DMSO-d_6) δ 8.35 (d, *J* = 9.1 Hz, 1H), 8.15 (td, *J* = 8.3, 1.1 Hz, 2H), 8.06 (dd, *J* = 7.6, 1.1 Hz, 1H), 7.87 (ddd, *J* = 8.7, 7.2, 1.7 Hz, 1H), 7.74 (t, *J* = 9.1 Hz, 2H), 7.56 (ddd, *J* = 11.5, 9.5, 4.4 Hz, 2H).
^{13}C NMR (101 MHz, DMSO-d_6) δ 174.24, 158.03, 153.68, 136.38, 135.30, 134.33, 132.66, 128.84, 128.23, 127.15, 125.76, 125.12, 124.30, 120.31, 118.92, 117.55, 115.89, 39.52.
HRMS m/z (EI-TOF): calculated for $C_{17}H_9BrO_2$ $[M]^+$ 323.9786, found 323.9788.

Methyl 5-oxo-5*H*-dibenzo[*c*,*f*]chromene-7-carboxylate (3aq)

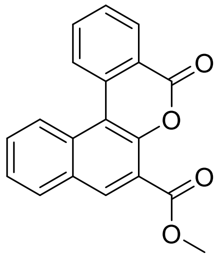

The preparation was according to the ***general procedure 2*** on 0.3 mmol scale, and obtained an isolated yield of 43% (39 mg) as yellow solid after column chromatography (petroleum ether: EtOAc = 8:1) on silica gel.

M.P.:145-148℃.

^{1}H NMR (400 MHz, CDCl$_3$) δ 8.53 (s, 1H), 8.01 (dd, *J* = 7.8, 1.7 Hz, 1H), 7.93 (d, *J* = 8.1 Hz, 1H), 7.69 (d, *J* = 8.2 Hz, 1H), 7.58 – 7.53 (m, 1H), 7.52 – 7.44 (m, 2H), 7.26 – 7.21 (m, 1H), 6.99 (d, *J* = 8.2 Hz, 1H), 3.90 (s, 3H).

^{13}C NMR (101 MHz, CDCl$_3$) δ 166.21, 156.77, 156.51, 153.13, 135.88, 133.73, 133.62, 132.14, 129.30, 128.91, 128.68, 126.97, 125.74, 123.47, 122.86, 122.70, 120.11, 115.99, 77.16, 52.43.

HRMS m/z (EI-TOF): calculated for $C_{19}H_{12}O_4$ [M]$^+$ 304.0736, found 304.0741.

9,10,11,12-Tetrahydro-5*H*-dibenzo[*c*,*f*]chromen-5-one (3ar)

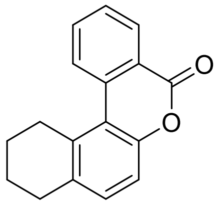

The preparation was according to the ***general procedure 2*** on 0.3 mmol scale, and obtained an isolated yield of 45% (34 mg) as yellow solid after column chromatography (petroleum ether: EtOAc = 8:1) on silica gel.

M.P.:144-147℃.

^{1}H NMR (400 MHz, CDCl$_3$) δ 8.25 (d, *J* = 8.0 Hz, 1H), 7.64 (dd, *J* = 8.3, 7.1 Hz, 1H), 7.42 – 7.28 (m, 3H), 7.20 (d, *J* = 8.5 Hz, 1H), 3.52 (t, *J* = 5.5 Hz, 2H), 2.81 (s, 2H), 1.87 – 1.76 (m, 4H).

^{13}C NMR (101 MHz, CDCl$_3$) δ 179.28, 156.28, 155.24, 140.73, 135.94, 134.16, 133.23, 126.92, 123.61, 123.26, 119.87, 117.41, 115.41, 77.16, 30.35, 29.40, 23.30, 22.26.

HRMS m/z (EI-TOF): calculated for $C_{17}H_{14}O_2$ [M]$^+$ 250.0994, found 250.0998.

8,9,10,11-Tetrahydro-5*H*-dibenzo[*c*,*g*]chromen-5-one (3as)

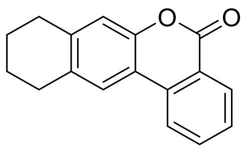

The preparation was according to the ***general procedure 2*** on 0.3 mmol scale, and obtained an isolated yield of 10% (7.5 mg) as yellow solid after column chromatography (petroleum ether: EtOAc = 8:1) on silica gel.
M.P.:142-145℃.
^{1}H NMR (400 MHz, $CDCl_3$) δ 8.33 (dd, *J* = 8.0, 1.6 Hz, 1H), 8.02 (s, 1H), 7.69 (ddd, *J* = 8.7, 7.1, 1.7 Hz, 1H), 7.48 – 7.43 (m, 1H), 7.37 – 7.31 (m, 1H), 7.19 (s, 1H), 2.91 (dd, *J* = 6.0, 3.3 Hz, 4H), 1.88 – 1.82 (m, 4H).
^{13}C NMR (101 MHz, $CDCl_3$) δ 177.36, 156.38, 154.32, 146.13, 134.58, 133.80, 126.85, 126.29, 123.63, 121.96, 119.85, 118.02, 117.30, 77.16, 30.27, 28.95, 23.13, 22.78.
HRMS m/z (EI-TOF): calculated for $C_{17}H_{14}O_2$ $[M]^+$ 250.0994, found 250.0998.

Methyl 2-((4-nitronaphthalen-1-yl)oxy)benzoate (3at)

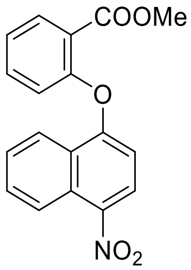

The preparation was according to the ***general procedure 2*** on 0.3 mmol scale, and obtained an isolated yield of 51% (49 mg) as yellow solid after column chromatography (petroleum ether: EtOAc = 8:1) on silica gel.
M.P.:133-136℃.
^{1}H NMR (400 MHz, $CDCl_3$) δ 8.79 (d, *J* = 8.8 Hz, 1H), 8.58 (d, *J* = 8.1 Hz, 1H), 8.24 (d, *J* = 8.7 Hz, 1H), 8.09 (dd, *J* = 7.8, 1.7 Hz, 1H), 7.81 (ddd, *J* = 8.6, 6.9, 1.3 Hz, 1H), 7.67 (ddd, *J* = 17.3, 8.1, 1.3 Hz, 2H), 7.41 (td, *J* = 7.8, 1.0 Hz, 1H), 7.22 (d, *J* = 8.1 Hz, 1H), 6.51 (d, *J* = 8.7 Hz, 1H), 3.61 (s, 3H).
^{13}C NMR (101 MHz, $CDCl_3$) δ 165.26, 160.01, 153.99, 134.52, 132.83, 130.56, 130.51, 127.29, 127.24, 126.48, 126.09, 125.88, 124.13, 123.67, 123.39, 123.05, 106.49, 77.16, 52.40.
HRMS m/z (EI-TOF): calculated for $C_{18}H_{13}NO_5$ $[M]^+$ 323.0794, found 323.0799.

2-Methoxy-6*H*-benzo[*c*]chromen-6-one (3au)

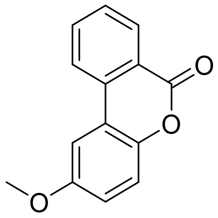

The preparation was according to the ***general procedure 2*** on 0.3 mmol scale, and obtained an isolated yield of 34% (23 mg) as yellow solid after column chromatography (petroleum ether: EtOAc = 8:1) on silica gel.
M.P.:140-143℃.
^{1}H NMR (400 MHz, $CDCl_3$) δ 8.36 (dd, *J* = 8.0, 1.6 Hz, 1H), 7.75 – 7.70 (m, 2H), 7.50 (d, *J* = 8.5 Hz, 1H), 7.45 (d, *J* = 9.1 Hz, 1H), 7.40 – 7.36 (m, 1H), 7.34 (dd, *J* = 9.1, 3.1 Hz, 1H), 3.93 (s, 3H).

^{13}C NMR (101 MHz, CDCl$_3$) δ 177.27, 156.14, 151.16, 134.75, 126.84, 125.09, 123.87, 120.44, 119.58, 119.20, 118.12, 114.98, 105.96, 77.16, 56.09.
HRMS m/z (EI-TOF): calculated for $C_{14}H_{10}O_3$ $[M]^+$ 226.0630, found 226.0638.

2-(*tert*-Butyl)-6*H*-benzo[*c*]chromen-6-one (3av)

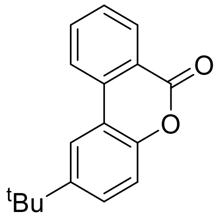

The preparation was according to the ***general procedure 2*** on 0.3 mmol scale, and obtained an isolated yield of 39% (29 mg) as yellow solid after column chromatography (petroleum ether: EtOAc = 8:1) on silica gel.
M.P.:135-138℃.
^{1}H NMR (400 MHz, CDCl$_3$) δ 8.34 (dd, *J* = 8.0, 1.5 Hz, 1H), 8.32 (d, *J* = 2.5 Hz, 1H), 7.79 – 7.76 (m, 1H), 7.69 (ddd, *J* = 8.7, 7.1, 1.7 Hz, 1H), 7.46 (dd, *J* = 8.4, 0.5 Hz, 1H), 7.42 (d, *J* = 8.8 Hz, 1H), 7.37 – 7.32 (m, 1H), 1.40 (s, 9H).
^{13}C NMR (101 MHz, CDCl$_3$) δ 177.54, 156.24, 154.41, 147.15, 141.40, 134.69, 132.86, 131.02, 126.83, 123.79, 122.50, 118.00, 117.69, 77.16, 34.84, 31.45.
HRMS m/z (EI-TOF): calculated for $C_{17}H_{16}O_2$ $[M]^+$ 252.1150, found 252.1145.

4-Methyl-5*H*-dibenzo[*c,f*]chromen-5-one (3ca)

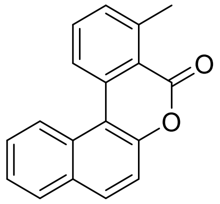

The preparation was according to the ***general procedure 2*** on 0.3 mmol scale, and obtained an isolated yield of 32% (25 mg) as yellow solid after column chromatography (petroleum ether: EtOAc = 8:1) on silica gel.
M.P.:122-125℃.
^{1}H NMR (400 MHz, CDCl$_3$) δ 8.70 (d, *J* = 8.6 Hz, 1H), 8.44 (d, *J* = 8.2 Hz, 1H), 7.91 (dd, *J* = 15.5, 8.4 Hz, 2H), 7.72 (t, *J* = 7.9 Hz, 1H), 7.64 (ddd, *J* = 8.5, 6.9, 1.4 Hz, 1H), 7.56 – 7.51 (m, 1H), 7.44 (t, *J* = 7.5 Hz, 2H), 2.92 (s, 3H).
^{13}C NMR (101 MHz, CDCl$_3$) δ 160.68, 149.98, 143.92, 136.80, 133.44, 131.68, 131.45, 131.44, 129.66, 129.36, 127.65, 125.30, 125.21, 124.89, 121.13, 117.26, 113.17, 77.16, 23.81.
HRMS m/z (EI-TOF): calculated for $C_{18}H_{12}O_2$ $[M]^+$ 260.0837, found 260.0838.

2-Chloro-5*H*-dibenzo[*c*,*f*]chromen-5-one (3da)

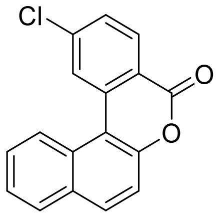

The preparation was according to the ***general procedure 2*** on 0.3 mmol scale, and obtained an isolated yield of 50% (42 mg) as yellow solid after column chromatography (petroleum ether: EtOAc = 8:1) on silica gel.

M.P.:132-135℃.

^{1}H NMR (400 MHz, $CDCl_3$) δ 8.68 (d, *J* = 8.7 Hz, 1H), 8.61 (d, *J* = 1.7 Hz, 1H), 8.42 (d, *J* = 8.5 Hz, 1H), 7.97 – 7.93 (m, 2H), 7.71 (ddd, *J* = 8.4, 6.9, 1.3 Hz, 1H), 7.61 – 7.55 (m, 2H), 7.47 (d, *J* = 8.9 Hz, 1H).

^{13}C NMR (101 MHz, $CDCl_3$) δ 160.71, 150.97, 141.46, 136.90, 132.52, 132.40, 131.70, 129.63, 129.41, 128.68, 128.43, 126.28, 125.78, 124.62, 120.71, 117.65, 111.60, 77.16.

HRMS m/z (EI-TOF): calculated for $C_{17}H_9ClO_2$ $[M]^+$ 280.0291, found 280.0298.

1-Methyl-5*H*-dibenzo[*c*,*f*]chromen-5-one (3ea)

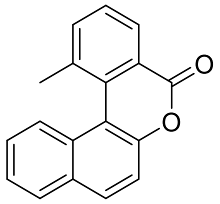

The preparation was according to the ***general procedure 2*** on 0.3 mmol scale, and obtained an isolated yield of 46% (36 mg) as yellow solid after column chromatography (petroleum ether: EtOAc = 8:1) on silica gel.

M.P.:135-138℃.

^{1}H NMR (400 MHz, $CDCl_3$) δ 8.31 (d, *J* = 7.7 Hz, 1H), 7.94 (dd, *J* = 13.5, 8.3 Hz, 2H), 7.76 (dd, *J* = 15.4, 8.1 Hz, 2H), 7.60 – 7.47 (m, 4H), 2.35 (s, 3H).

^{13}C NMR (101 MHz, $CDCl_3$) δ 162.26, 149.87, 137.84, 135.67, 134.31, 131.16, 131.03, 129.39, 128.44, 128.11, 127.99, 127.04, 126.38, 125.29, 124.57, 117.16, 113.57, 77.16, 23.84.

HRMS m/z (EI-TOF): calculated for $C_{18}H_{12}O_2$ $[M]^+$ 260.0837, found 260.0838.

2-(Trifluoromethyl)-5*H*-dibenzo[*c*,*f*]chromen-5-one (3fa)

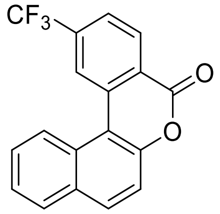

The preparation was according to the ***general procedure 2*** on 0.3 mmol scale, and obtained an isolated yield of 49% (46 mg) as yellow solid after column chromatography (petroleum ether: EtOAc = 8:1) on silica gel.
M.P.:140-143℃.
^{1}H NMR (400 MHz, CDCl$_3$) δ 8.90 (s, 1H), 8.66 (d, *J* = 8.6 Hz, 1H), 8.61 (d, *J* = 8.2 Hz, 1H), 7.98 (d, *J* = 9.1 Hz, 2H), 7.85 (d, *J* = 7.8 Hz, 1H), 7.74 (ddd, *J* = 8.4, 7.0, 1.3 Hz, 1H), 7.61 (dd, *J* = 11.2, 3.9 Hz, 1H), 7.50 (d, *J* = 8.9 Hz, 1H).
^{13}C NMR (101 MHz, CDCl$_3$) δ 160.31, 150.90, 136.01, 135.94 (q, J_{C-F} = 33.3Hz), 132.80, 131.80, 131.72, 129.73, 129.35, 128.70, 125.95, 124.57(q, J_{C-F} = 4Hz), 124.41, 123.62 (q, J_{C-F} = 274.7Hz), 123.56 (q, J_{C-F} = 4 Hz), 117.56, 114.34, 111.76, 77.16.
^{19}F NMR (376 MHz, CDCl$_3$) δ -63.16.
HRMS m/z (EI-TOF): calculated for $C_{18}H_9F_3O_2$ $[M]^+$ 314.0555, found 314.0558.

2-Bromo-5*H*-dibenzo[*c*,*f*]chromen-5-one (3ga)

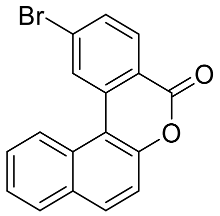

The preparation was according to the ***general procedure 2*** on 0.3 mmol scale, and obtained an isolated yield of 55% (53 mg) as yellow solid after column chromatography (petroleum ether: EtOAc = 8:1) on silica gel.
M.P.:136-139℃.
^{1}H NMR (400 MHz, CDCl$_3$) δ 8.78 (d, *J* = 9.8 Hz, 1H), 8.65 (d, *J* = 8.6 Hz, 1H), 8.32 (d, *J* = 8.4 Hz, 1H), 7.94 (dd, *J* = 8.4, 3.1 Hz, 2H), 7.72 (ddd, *J* = 12.8, 8.4, 1.4 Hz, 2H), 7.57 (t, *J* = 7.5 Hz, 1H), 7.46 (d, *J* = 8.9 Hz, 1H).
^{13}C NMR (101 MHz, CDCl$_3$) δ 160.82, 150.94, 136.95, 132.51, 132.33, 131.69, 131.55, 130.21, 129.62, 129.37, 129.27, 128.46, 125.78, 124.58, 121.07, 117.63, 111.47, 77.16.
HRMS m/z (EI-TOF): calculated for $C_{17}H_9BrO_2$ $[M]^+$ 323.9786, found 323.9791.

2,3-Dimethoxy-5*H*-dibenzo[*c*,*f*]chromen-5-one (3ha)

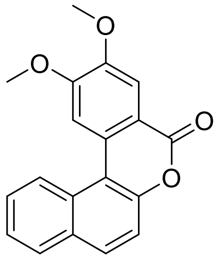

The preparation was according to the ***general procedure 2*** on 0.3 mmol scale, and obtained an isolated yield of 43% (39 mg) as yellow solid after column chromatography (petroleum ether: EtOAc = 8:1) on silica gel.
M.P.:129-132℃.

^{1}H NMR (400 MHz, CDCl$_3$) δ 8.76 (d, *J* = 8.6 Hz, 1H), 8.08 (s, 1H), 7.96 (d, *J* = 7.7 Hz, 1H), 7.89 (d, *J* = 8.3 Hz, 2H), 7.67 (ddd, *J* = 8.5, 6.9, 1.3 Hz, 1H), 7.58 – 7.53 (m, 1H), 7.49 (d, *J* = 8.9 Hz, 1H), 4.11 (s, 3H), 4.05 (s, 3H).
^{13}C NMR (101 MHz, CDCl$_3$) δ 161.31, 154.55, 150.07, 149.54, 131.80, 130.80, 130.76, 129.66, 129.47, 127.69, 125.37, 124.58, 117.85, 115.88, 112.72, 110.93, 108.12, 77.16, 56.52, 56.47.
HRMS m/z (EI-TOF): calculated for $C_{19}H_{14}O_4$ $[M]^+$ 306.0892, found 306.0895.

3-Fluoro-5*H*-dibenzo[*c,f*]chromen-5-one (3ia)

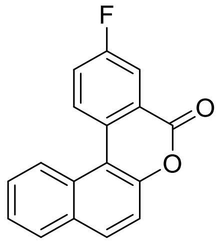

The preparation was according to the ***general procedure 2*** on 0.3 mmol scale, and obtained an isolated yield of 21% (17 mg) as yellow solid after column chromatography (petroleum ether: EtOAc = 8:1) on silica gel.
M.P.:130-133°C.
^{1}H NMR (400 MHz, CDCl$_3$) δ 8.71 – 8.62 (m, 2H), 8.15 (dd, *J* = 8.4, 2.9 Hz, 1H), 7.93 (dd, *J* = 13.6, 8.2 Hz, 2H), 7.68 (ddd, *J* = 8.5, 6.9, 1.4 Hz, 1H), 7.63 – 7.53 (m, 2H), 7.48 (d, *J* = 8.9 Hz, 1H).
^{13}C NMR (101 MHz, CDCl$_3$) δ 160.56, 149.93, 132.08(d, J_{C-F} = 3.0Hz), 131.95, 131.81, 131.63, 129.57, 129.37, 128.93(d, J_{C-F} = 8.1Hz),127.89, 126.88(d, J_{C-F} = 243.4Hz), 124.85, 124.46(d, J_{C-F} = 8.1Hz), 122.53(d, J_{C-F} = 22.2Hz), 117.63, 116.50(d, J_{C-F} = 23.2Hz), 112.13, 77.16.
^{19}F NMR (376 MHz, CDCl$_3$) δ -110.76.
HRMS m/z (EI-TOF): calculated for $C_{17}H_9FO_2$ $[M]^+$ 264.0587, found 264.0580.

3-Methyl-5*H*-dibenzo[*c,f*]chromen-5-one (3ja)

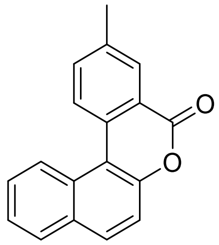

The preparation was according to the ***general procedure 2*** on 0.3 mmol scale, and obtained an isolated yield of 59% (46 mg) as yellow solid after column chromatography (petroleum ether: EtOAc = 8:1) on silica gel.
M.P.:154-157°C.
^{1}H NMR (400 MHz, CDCl$_3$) δ 8.74 (d, *J* = 8.6 Hz, 1H), 8.53 (d, *J* = 8.4 Hz, 1H), 8.29 (d, *J* = 0.8 Hz, 1H), 7.93 (d, *J* = 7.9 Hz, 1H), 7.88 (d, *J* = 8.9 Hz, 1H), 7.71 – 7.62 (m, 2H), 7.57 – 7.51 (m, 1H), 7.46 (d, *J* = 8.9 Hz, 1H), 2.54 (s, 3H).
^{13}C NMR (101 MHz, CDCl$_3$) δ 161.62, 149.98, 138.66, 135.64, 132.96, 131.72, 131.10, 130.65, 129.61, 129.41, 127.73, 126.45, 125.40, 125.16, 122.30, 117.69, 112.78, 77.16, 21.29.
HRMS m/z (EI-TOF): calculated for $C_{18}H_{12}O_2$ $[M]^+$ 260.0837, found 260.0831.

8-Methyl-6*H*-dibenzo[*c*,*h*]chromen-6-one (3jb)

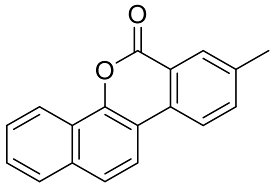

The preparation was according to the ***general procedure 2*** on 0.3 mmol scale, and obtained an isolated yield of 28% (22 mg) as yellow solid after column chromatography (petroleum ether: EtOAc = 8:1) on silica gel.

M.P.:150-153℃.

^{1}H NMR (400 MHz, CDCl$_3$) δ 8.57 (d, *J* = 8.0 Hz, 1H), 8.26 (s, 1H), 8.05 (dd, *J* = 16.5, 8.5 Hz, 2H), 7.86 (d, *J* = 7.4 Hz, 1H), 7.75 (d, *J* = 8.8 Hz, 1H), 7.66 (dd, *J* = 8.2, 1.4 Hz, 1H), 7.61 (ddd, *J* = 9.1, 7.7, 1.1 Hz, 2H), 2.51 (s, 3H).

^{13}C NMR (101 MHz, CDCl$_3$) δ 161.57, 146.86, 139.09, 136.31, 134.10, 132.94, 130.51, 130.48, 127.75, 127.15, 124.52, 123.97, 122.32, 122.12, 121.08, 119.26, 113.29, 77.16, 21.40.

HRMS m/z (EI-TOF): calculated for $C_{18}H_{12}O_2$ $[M]^+$ 260.0837, found 260.0840.

3-Chloro-5*H*-dibenzo[*c*,*f*]chromen-5-one (3ka)

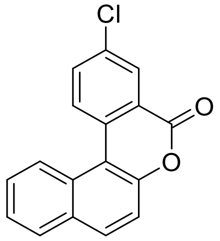

The preparation was according to the ***general procedure 2*** on 0.3 mmol scale, and obtained an isolated yield of 37% (31 mg) as yellow solid after column chromatography (petroleum ether: EtOAc = 8:1) on silica gel.

M.P.:140-143℃.

^{1}H NMR (400 MHz, CDCl$_3$) δ 8.68 (d, *J* = 8.6 Hz, 1H), 8.60 (d, *J* = 8.8 Hz, 1H), 8.46 (d, *J* = 2.4 Hz, 1H), 7.95 (t, *J* = 7.8 Hz, 2H), 7.83 (dd, *J* = 8.8, 2.4 Hz, 1H), 7.69 (ddd, *J* = 8.5, 7.0, 1.3 Hz, 1H), 7.57 (t, *J* = 7.5 Hz, 1H), 7.48 (d, *J* = 8.9 Hz, 1H).

^{13}C NMR (101 MHz, CDCl$_3$) δ 160.30, 150.35, 134.66, 134.31, 133.93, 132.05, 131.77, 130.25, 129.60, 129.40, 128.19, 128.01, 125.74, 124.82, 123.77, 117.62, 112.00, 77.16.

HRMS m/z (EI-TOF): calculated for $C_{17}H_9ClO_2$ $[M]^+$ 280.0291, found 280.0290.

8-Chloro-6*H*-dibenzo[*c*,*h*]chromen-6-one (3kb)

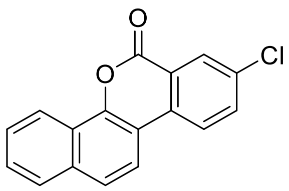

The preparation was according to the ***general procedure 2*** on 0.3 mmol scale, and obtained an isolated yield of 45% (37 mg) as yellow solid after column chromatography (petroleum ether: EtOAc = 8:1) on silica gel.
M.P.:142-145℃.
^{1}H NMR (400 MHz, $CDCl_3$) δ 8.58 – 8.53 (m, 1H), 8.42 (d, *J* = 2.3 Hz, 1H), 8.12 (d, *J* = 8.6 Hz, 1H), 8.00 (d, *J* = 8.8 Hz, 1H), 7.88 (dd, *J* = 6.7, 2.4 Hz, 1H), 7.82 – 7.76 (m, 2H), 7.66 – 7.60 (m, 2H).
^{13}C NMR (101 MHz, $CDCl_3$) δ 160.27, 147.31, 135.41, 134.82, 134.48, 133.98, 130.24, 130.20, 128.28, 127.85, 127.48, 124.96, 123.88, 122.52, 122.42, 119.04, 112.44, 77.16.
HRMS m/z (EI-TOF): calculated for $C_{17}H_9ClO_2$ $[M]^+$ 280.0291, found 280.0295.

3-Bromo-5*H*-dibenzo[*c*,*f*]chromen-5-one (3la)

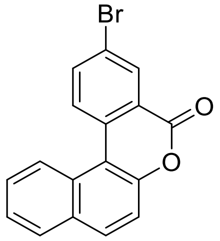

The preparation was according to the ***general procedure 2*** on 0.3 mmol scale, and obtained an isolated yield of 35% (34 mg) as yellow solid after column chromatography (petroleum ether: EtOAc = 8:1) on silica gel.
M.P.:140-143℃.
^{1}H NMR (400 MHz, $CDCl_3$) δ 8.64 (d, *J* = 8.6 Hz, 1H), 8.59 (d, *J* = 2.2 Hz, 1H), 8.49 (d, *J* = 8.8 Hz, 1H), 7.94 (dt, *J* = 15.6, 5.6 Hz, 3H), 7.67 (ddd, *J* = 8.5, 6.9, 1.3 Hz, 1H), 7.59 – 7.53 (m, 1H), 7.45 (d, *J* = 8.9 Hz, 1H).
^{13}C NMR (101 MHz, $CDCl_3$) δ 160.15, 150.40, 137.48, 134.30, 133.31, 132.13, 131.78, 129.61, 129.37, 128.21, 128.08, 125.75, 124.81, 123.90, 122.08, 117.63, 112.04, 77.16.
HRMS m/z (EI-TOF): calculated for $C_{17}H_9BrO_2$ $[M]^+$ 323.9786, found 323.9791.

4-Fluoro-5*H*-dibenzo[*c*,*f*]chromen-5-one (3ma)

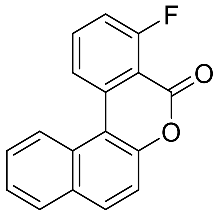

The preparation was according to the ***general procedure 2*** on 0.3 mmol scale, and obtained an isolated yield of 50% (40 mg) as yellow solid after column chromatography (petroleum ether: EtOAc = 8:1) on silica gel.
M.P.:147-150℃.
^{1}H NMR (400 MHz, $CDCl_3$) δ 8.68 (d, *J* = 8.6 Hz, 1H), 8.40 (d, *J* = 8.3 Hz, 1H), 7.94 (dd, *J* = 8.5, 4.9 Hz, 2H), 7.84 (td, *J* = 8.3, 5.4 Hz, 1H), 7.70 – 7.63 (m, 1H), 7.56 (t, *J* = 7.5 Hz, 1H), 7.44 (d, *J* = 8.9 Hz, 1H), 7.31 (dd, *J* = 10.0, 8.6 Hz, 1H).

^{13}C NMR (101 MHz, CDCl$_3$) δ 164.79, 162.13, 156.96, 156.91, 150.66, 137.98, 135.79(d, $J_{C\text{-}F}$ = 10.1Hz), 132.46, 130.63(d, $J_{C\text{-}F}$ = 226.1Hz), 129.55, 128.14, 125.69, 124.95, 122.50(d, $J_{C\text{-}F}$ = 4.1Hz), 117.41, 115.79(d, $J_{C\text{-}F}$ = 21.2Hz), 111.91(d, $J_{C\text{-}F}$ = 22.1Hz), 111.40(d, $J_{C\text{-}F}$ = 10.0Hz), 77.16.
^{19}F NMR (377 MHz, CDCl$_3$) δ -106.47.
HRMS m/z (EI-TOF): calculated for $C_{17}H_9FO_2$ $[M]^+$ 264.0587, found 264.0590.

Part 5. Mechanism study

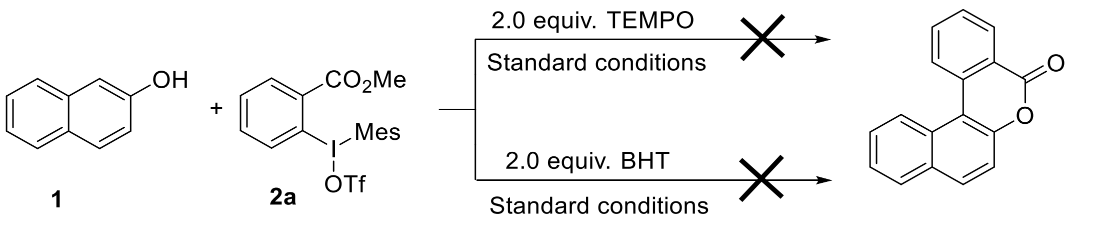

To an oven-dried Schlenk tube was added 2-Naphthol **1** (0.3 mmol, 1.0 equiv) *ortho*-ester-substituted diaryliodonium salt **2** (0.33 mmol, 1.1 equiv) $Cu(OAc)_2$ (0.03 mmol, 10% equiv) and DCE (2 mL). BHT or TEMPO (2.0 equiv) was added. The mixture was stirred at 80 °C in an oil bath until TLC indicated that the 2-naphthol was completely consumed. TEMPO = 2,2,6,6-tetramethylpiperidine-1-oxyl. BHT = butylated hydroxytoluene.

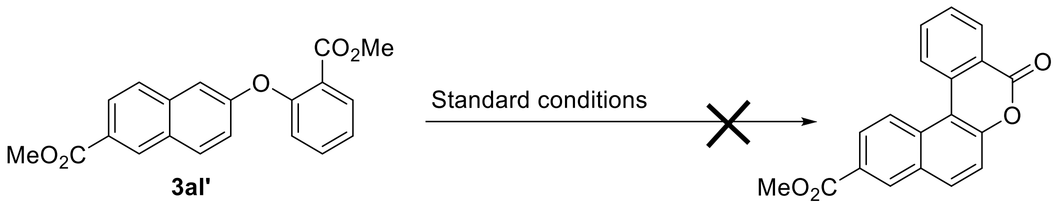

To an oven-dried Schlenk tube was added **3al’** (0.3 mmol, 1.0 equiv) $Cu(OAc)_2$ (0.03 mmol, 10% equiv) and DCE (2 mL). The mixture was stirred at 80 °C in an oil bath.

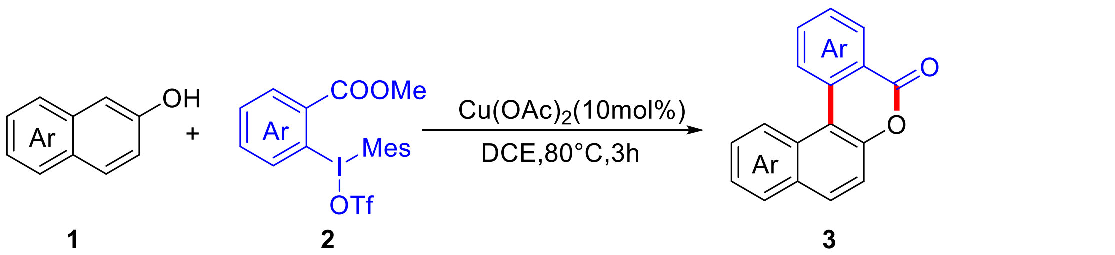

To an oven-dried Schlenk tube was added 2-naphthol **1** (0.3 mmol, 1.0 equiv.) *ortho*-ester-substituted diaryliodonium salt **2** (0.33 mmol, 1.1 equiv) $Cu(OAc)_2$ (0.03mmol, 10% equiv) and DCE (2 mL). The mixture was stirred at 80 °C in an oil bath. After 1 hours, a drop of the reaction mixture was delivered to LC-MS analysis.

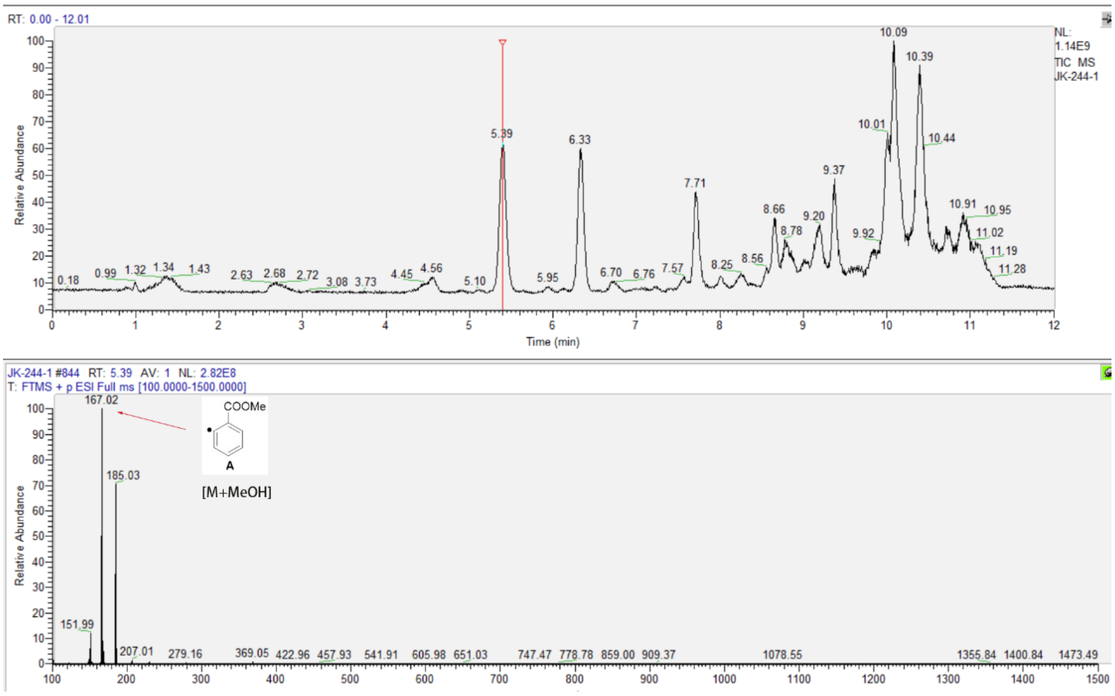

Figure S1. LC-MS spectra of reaction mixture

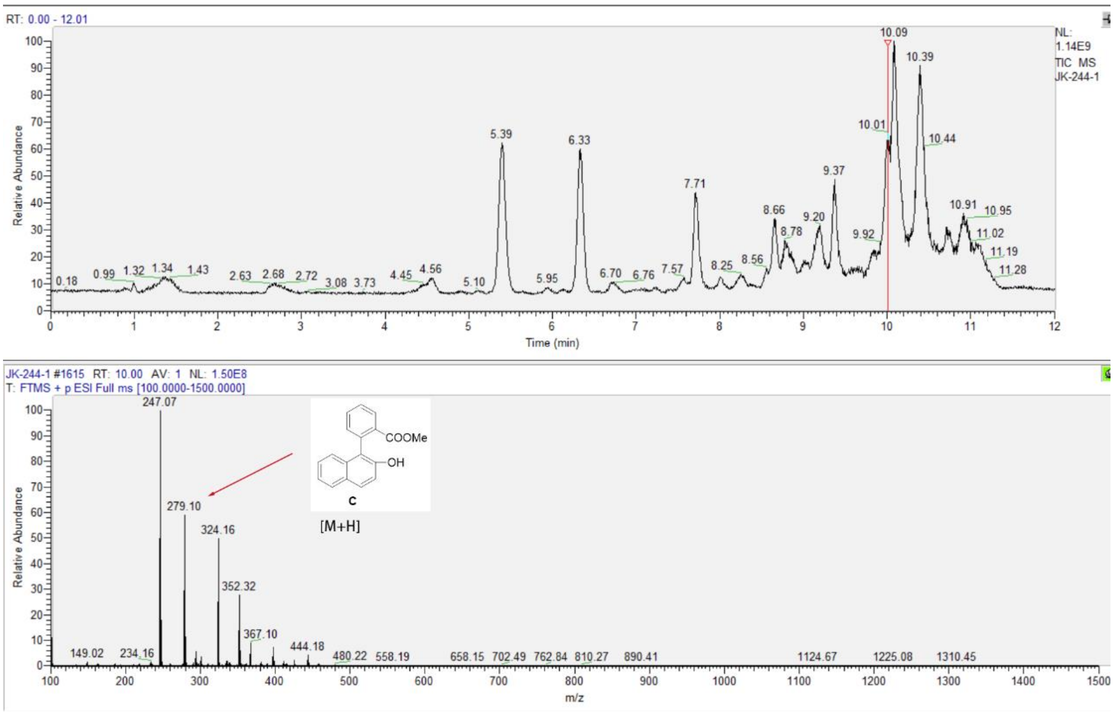

Figure S2. LC-MS spectra of reaction mixture

Part 6. References

[1] Yang, J.; Xu, X.; Qing, F. *J. Fluorine Chem*. **2015**, 180,175-180.

[2] Saravanan, P.; Anbarasan, P.; *Adv. Synth. Catal*. **2015**, *357*, 3521-3528.

[3] Beugelmans, R.; Bois-Choussy, M.; Chastanet, J.; *Heterocycl*. **1993**, *36*, 2723-2732.

Part 7. ^1H, ^{13}C, and ^{19}F NMR spectra of products

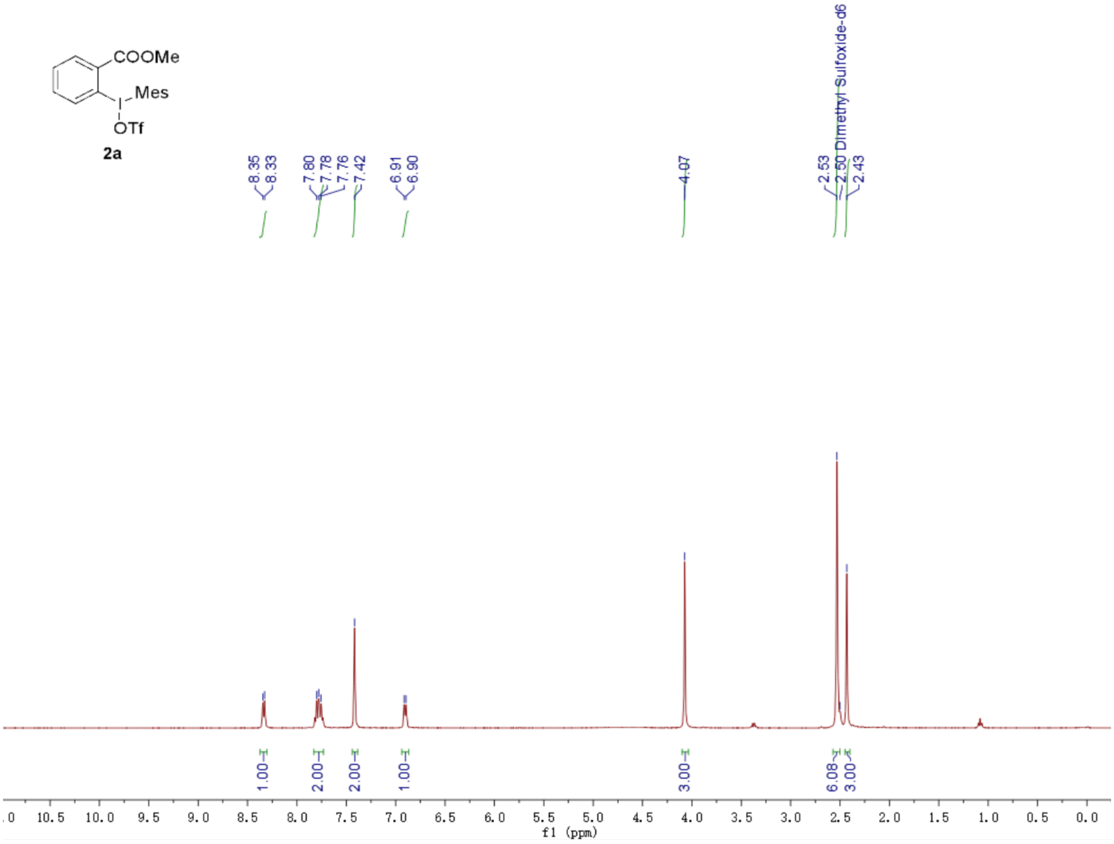

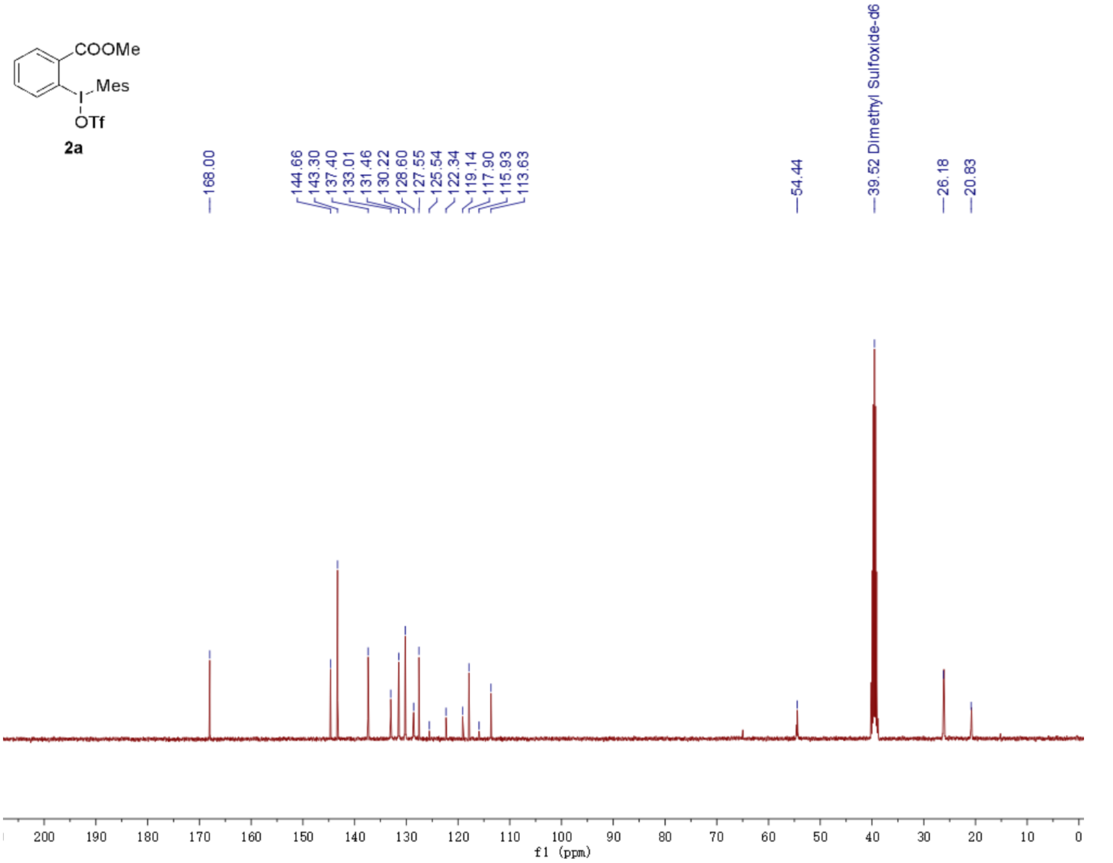

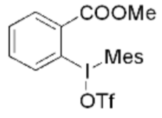
COOMe
Mes
OTf
2a

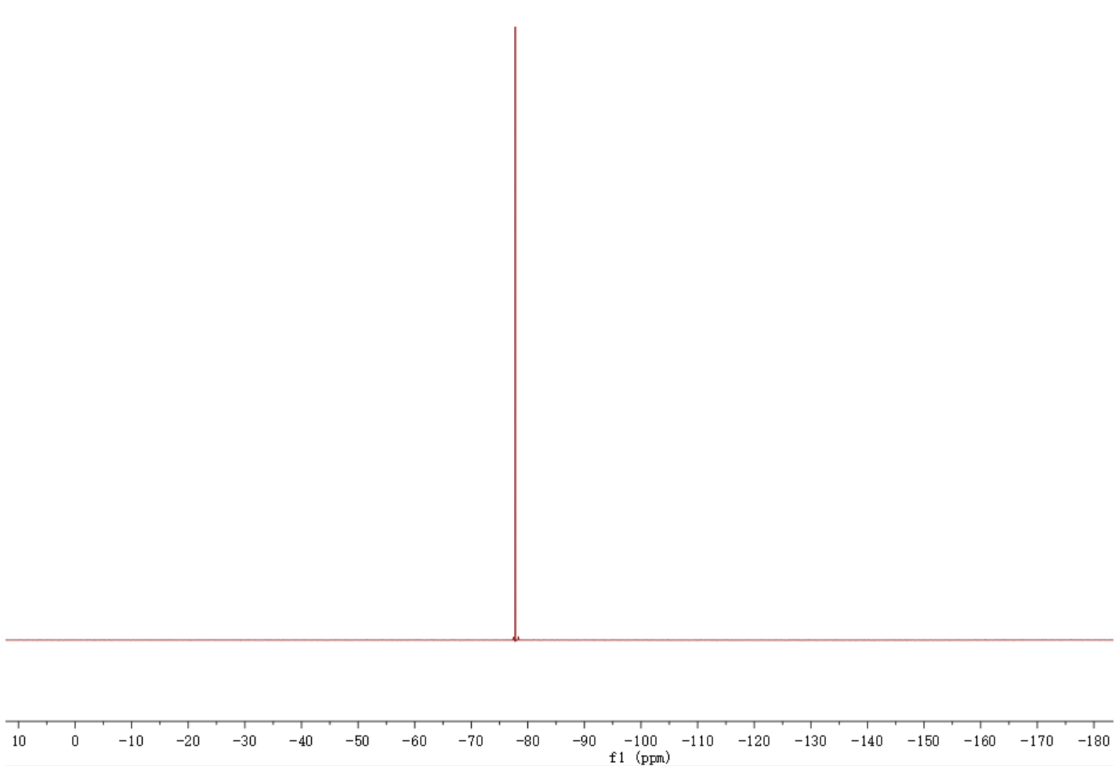
-77.77
10 0 −10 −20 −30 −40 −50 −60 −70 −80 −90 −100 −110 −120 −130 −140 −150 −160 −170 −180
f1 (ppm)

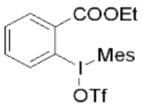
COOEt
Mes
OTf
2b

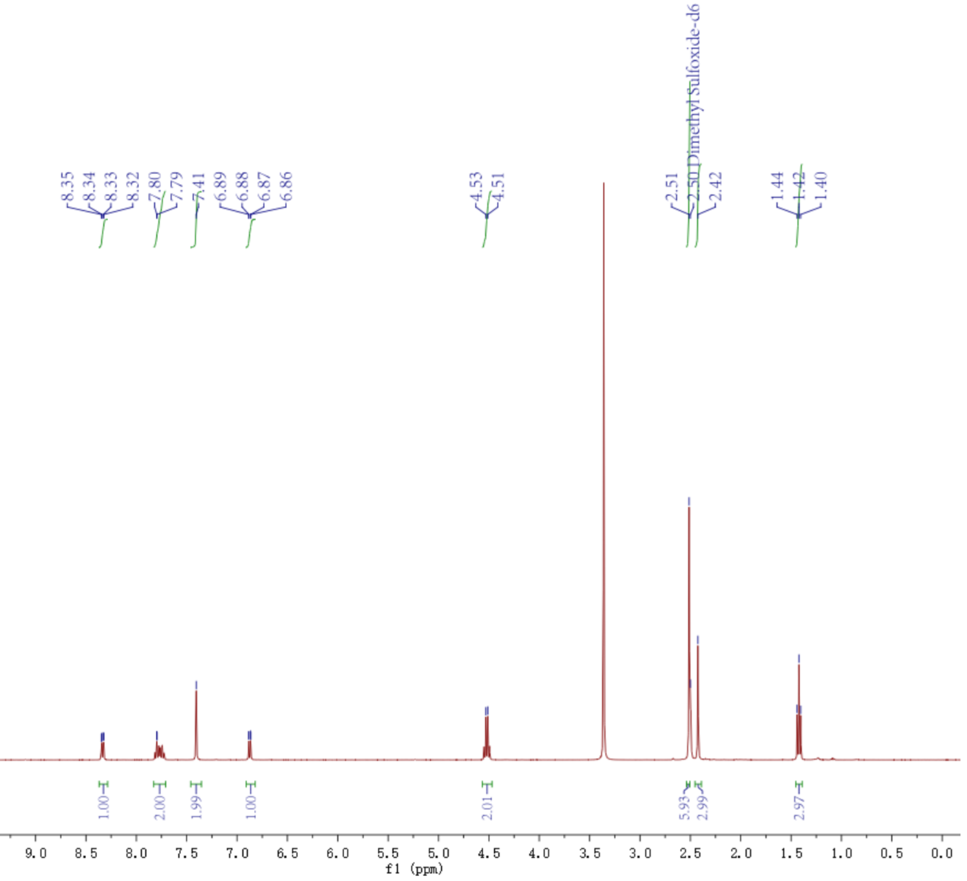
8.35
8.34
8.33
8.32
7.80
7.79
7.41
6.89
6.88
6.87
6.86
4.53
4.51
2.51
2.50 Dimethyl Sulfoxide-d6
2.42
1.44
1.42
1.40
1.00
2.00
1.99
1.00
2.01
5.93
2.99
2.97
10.5 10.0 9.5 9.0 8.5 8.0 7.5 7.0 6.5 6.0 5.5 5.0 4.5 4.0 3.5 3.0 2.5 2.0 1.5 1.0 0.5 0.0
f1 (ppm)

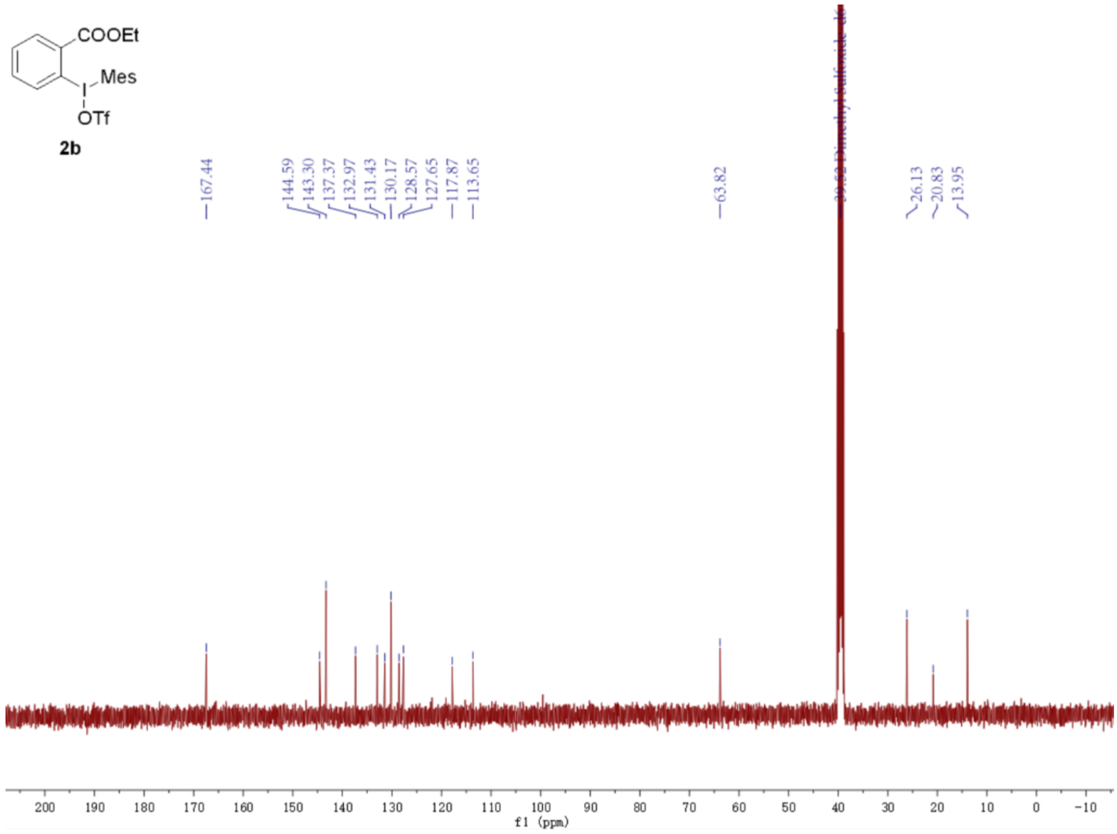
COOEt
Mes
I
OTf
2b
167.44
144.59
143.30
137.37
132.97
131.43
130.17
128.57
127.65
117.87
113.65
63.82
26.13
20.83
13.95
f1 (ppm)

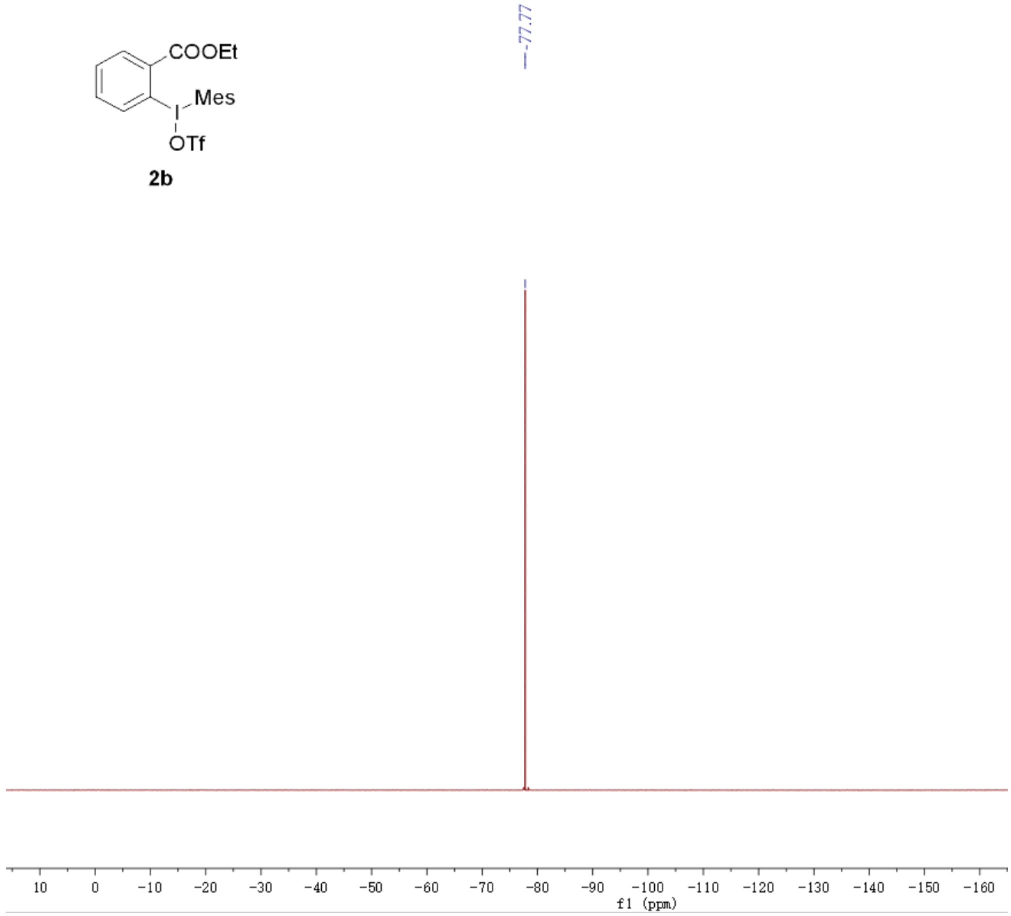
COOEt
Mes
I
OTf
2b
-77.77
f1 (ppm)

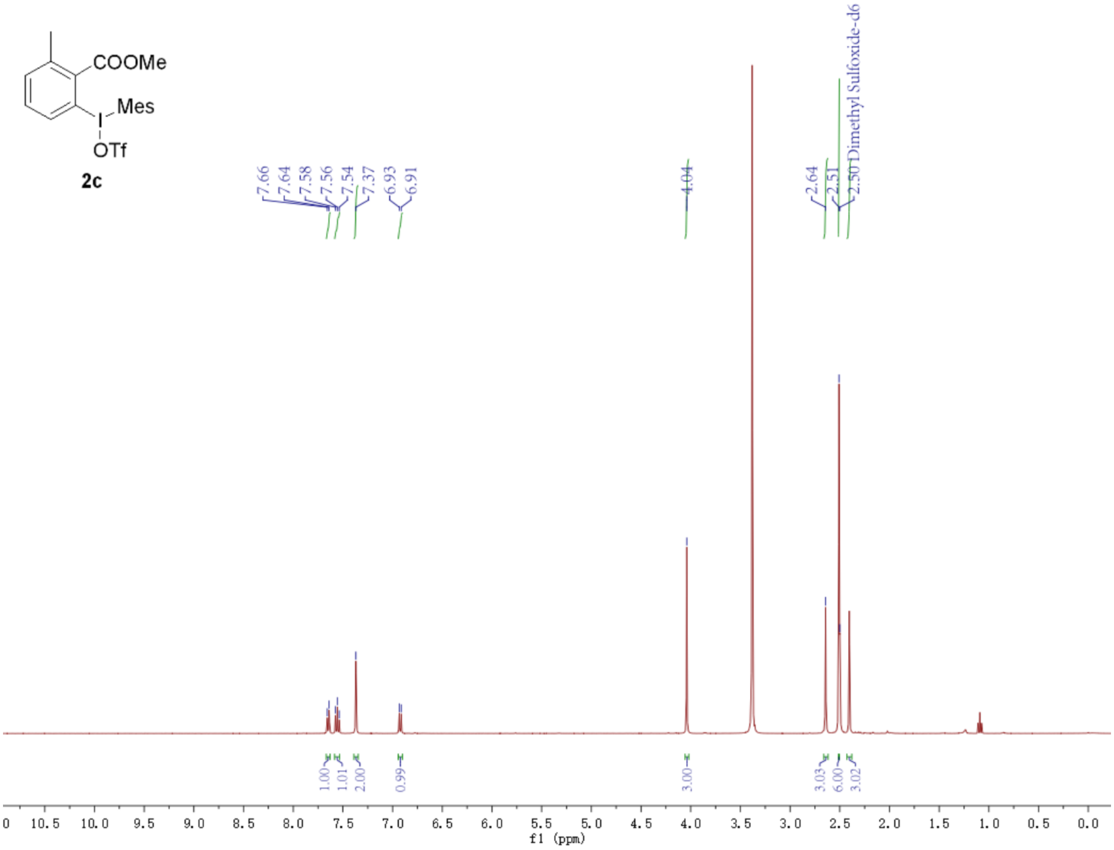
COOMe
Mes
I
OTf
2c
7.66
7.64
7.58
7.56
7.54
7.37
6.93
6.91
4.04
2.64
2.51
2.50 Dimethyl Sulfoxide-d6
1.00
1.01
2.00
0.99
3.00
3.03
6.00
3.02
f1 (ppm)

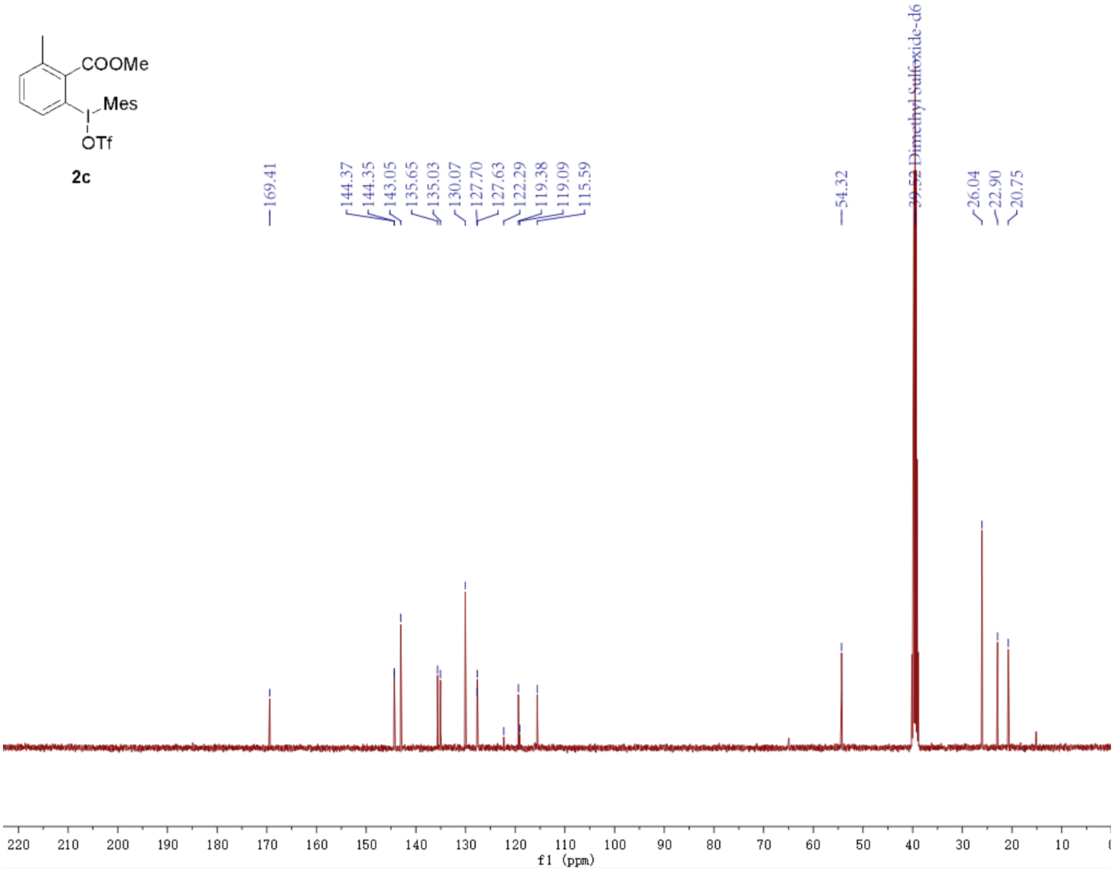
COOMe
Mes
I
OTf
2c
169.41
144.37
144.35
143.05
135.65
135.03
130.07
127.70
127.63
122.29
119.38
119.09
115.59
54.32
39.52 Dimethyl Sulfoxide-d6
26.04
22.90
20.75
f1 (ppm)

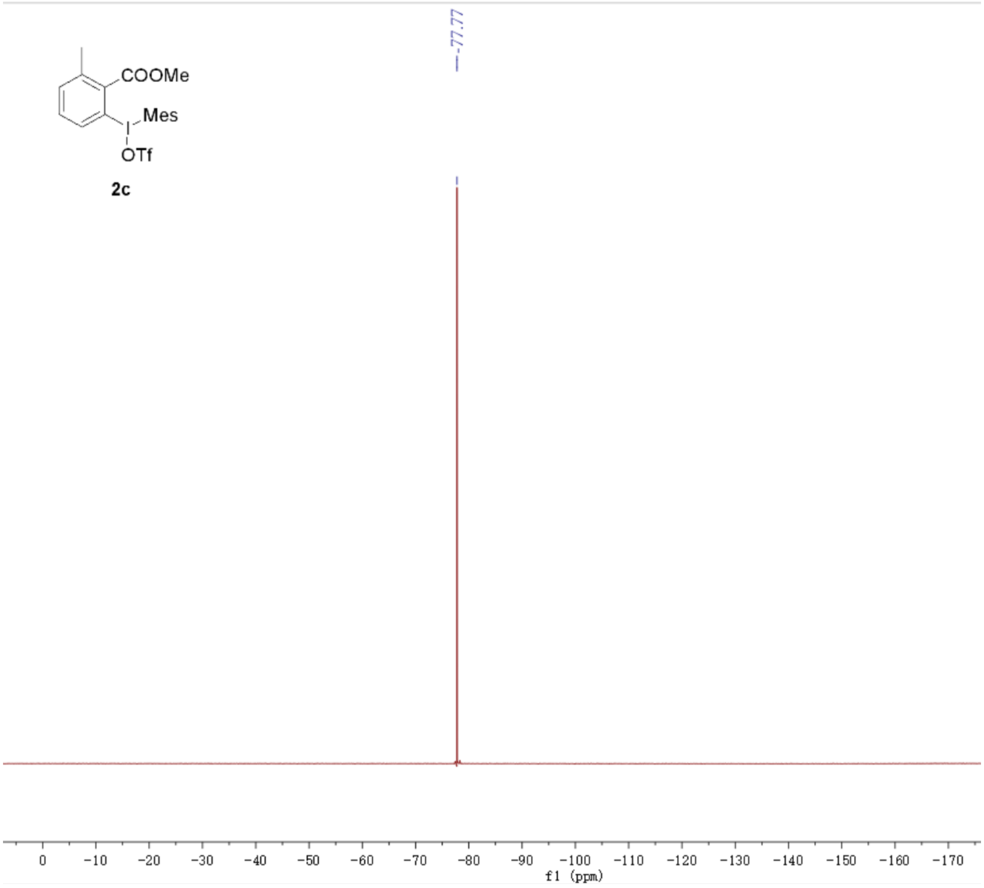
COOMe
Mes
I
OTf
-77.77
f1 (ppm)

2c

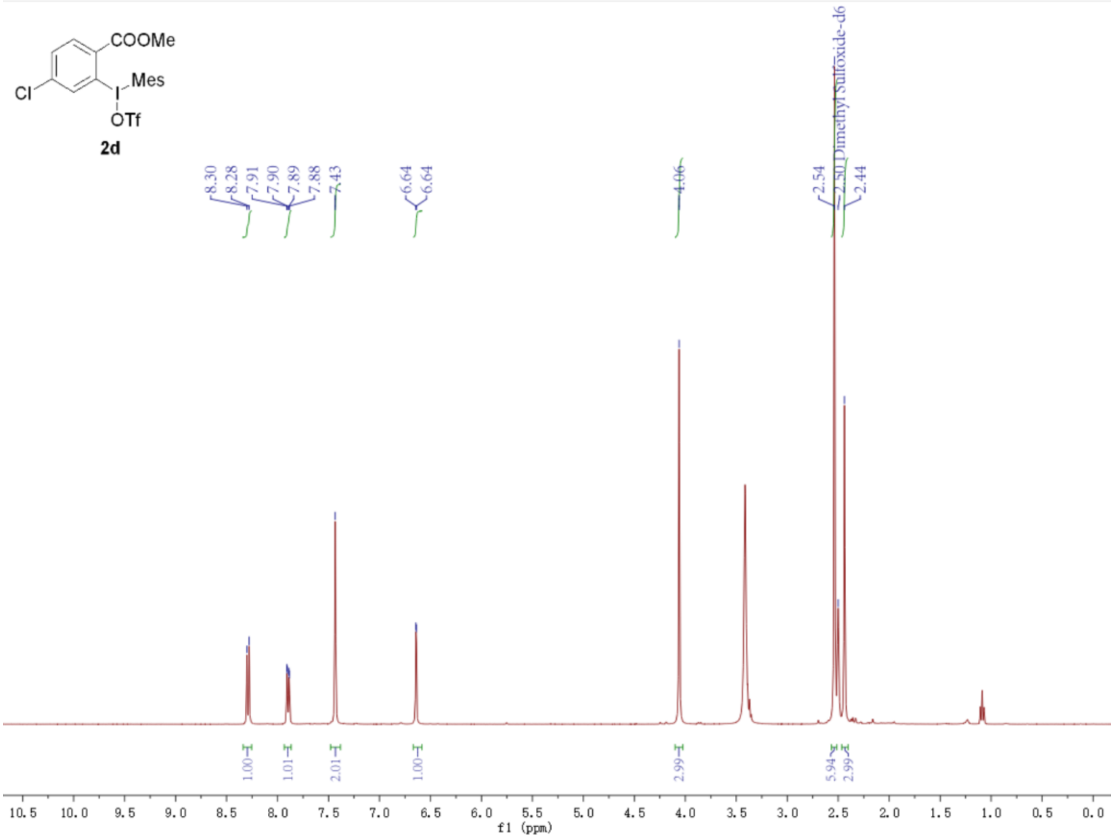
COOMe
Cl
Mes
I
OTf
8.30
8.28
7.91
7.90
7.89
7.88
7.43
6.64
6.64
4.06
2.54
2.50 Dimethyl Sulfoxide-d6
2.44
1.00
1.01
2.01
1.00
2.99
5.94
2.99
f1 (ppm)

2d

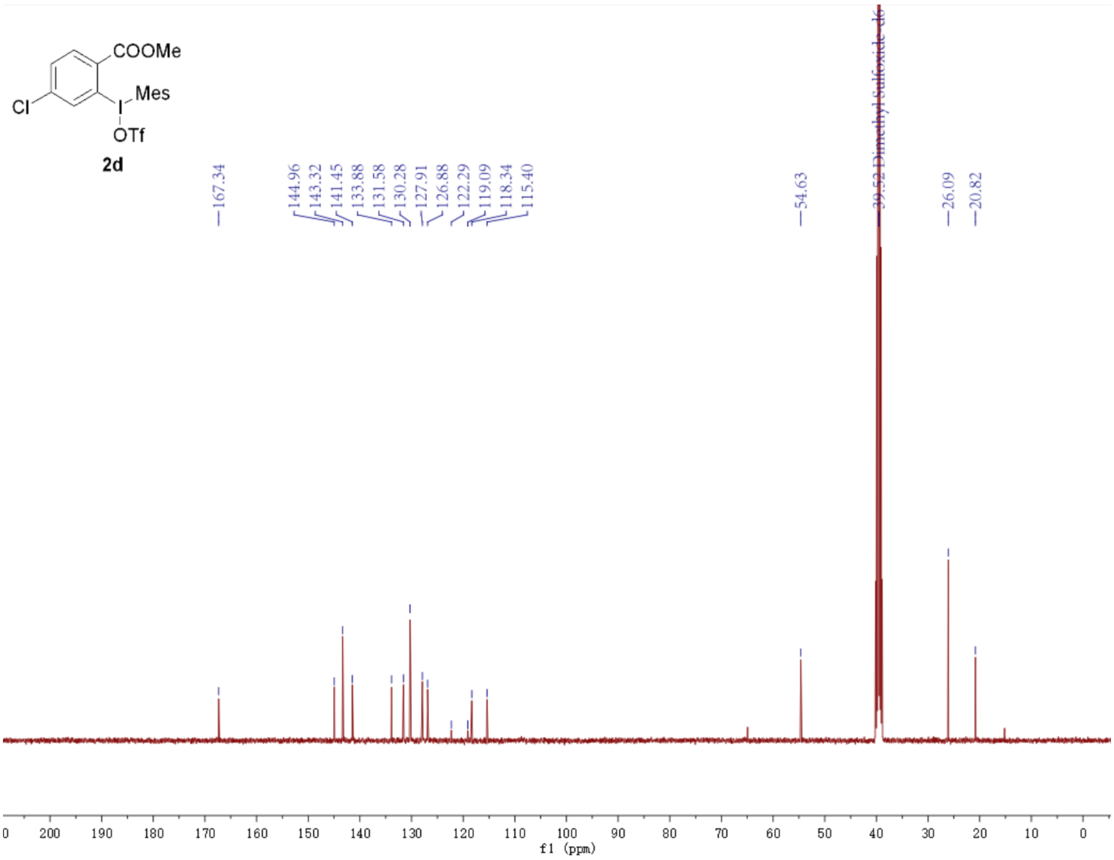
COOMe
Cl
I
Mes
OTf
2d
167.34
144.96
143.32
141.45
133.88
131.58
130.28
127.91
126.88
122.29
119.09
118.34
115.40
54.63
39.52 Dimethyl Sulfoxide-d6
26.09
20.82
f1 (ppm)

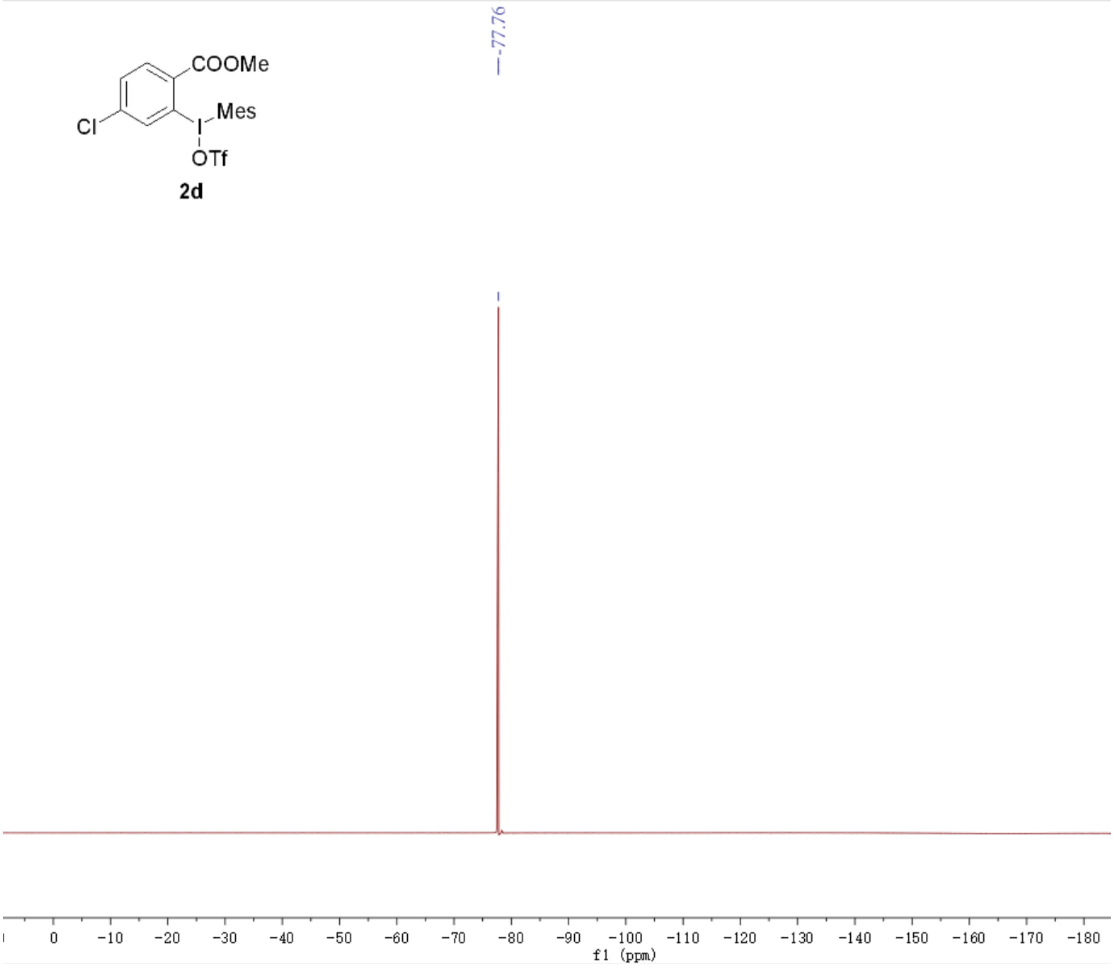
COOMe
Cl
I
Mes
OTf
2d
-77.76
f1 (ppm)

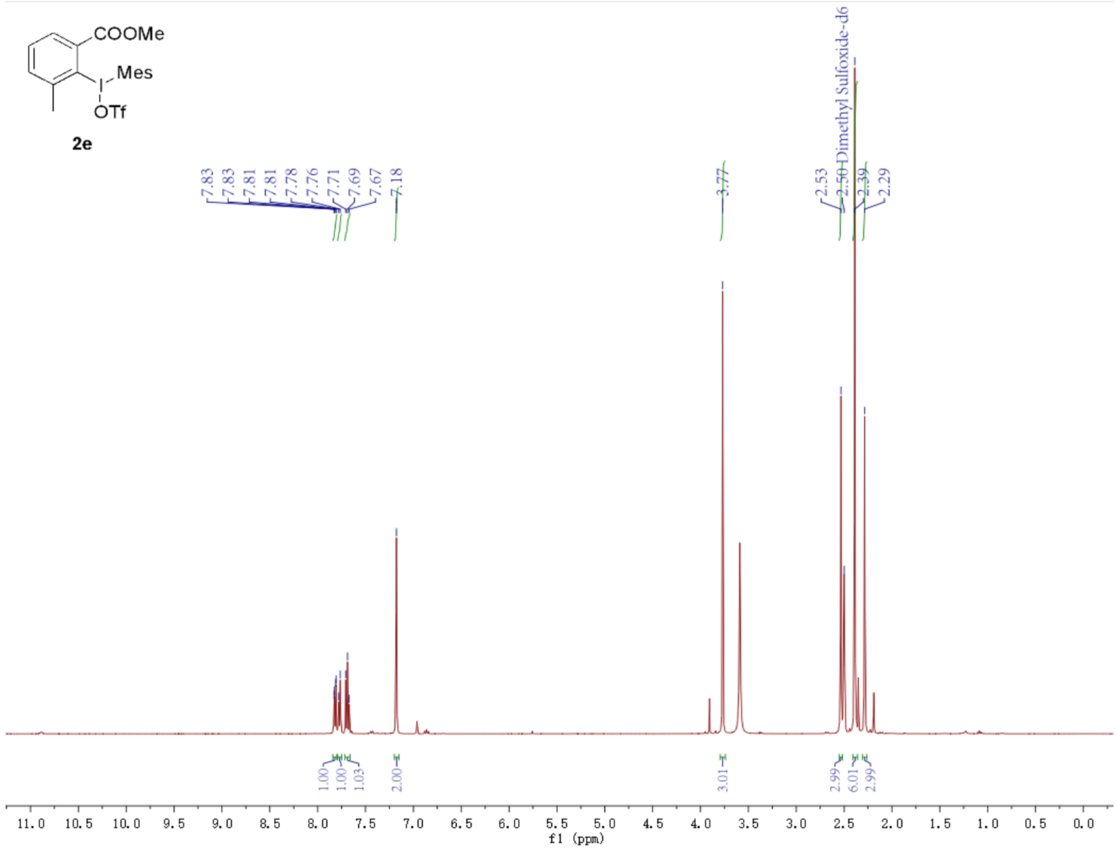
COOMe
Mes
OTf
2e
7.83
7.83
7.81
7.81
7.78
7.76
7.71
7.69
7.67
7.18
3.77
2.53
2.50 Dimethyl Sulfoxide-d6
2.39
2.29
1.00
1.00
1.03
2.00
3.01
2.99
6.01
2.99
f1 (ppm)

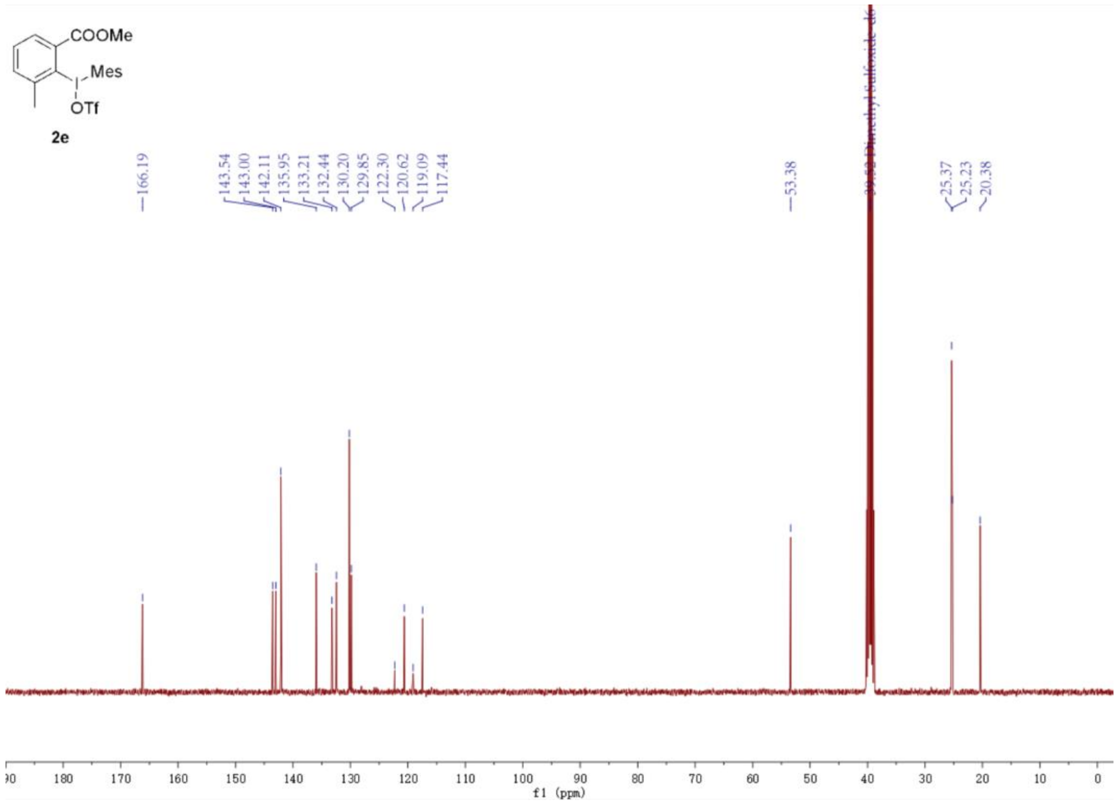
COOMe
Mes
OTf
2e
166.19
143.54
143.00
142.11
135.95
133.21
132.44
130.20
129.85
122.30
120.62
119.09
117.44
53.38
25.37
25.23
20.38
f1 (ppm)

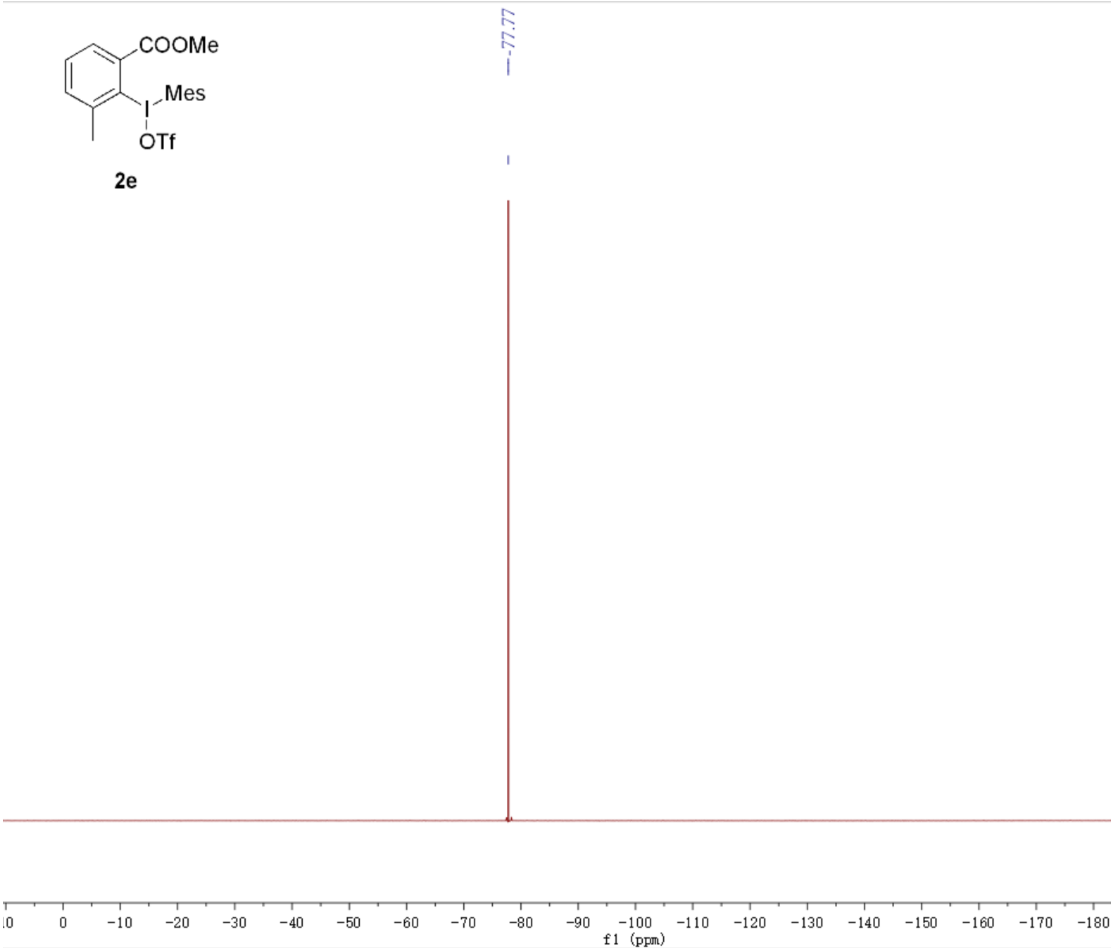
COOMe
Mes
I
OTf
2e
-77.77
f1 (ppm)

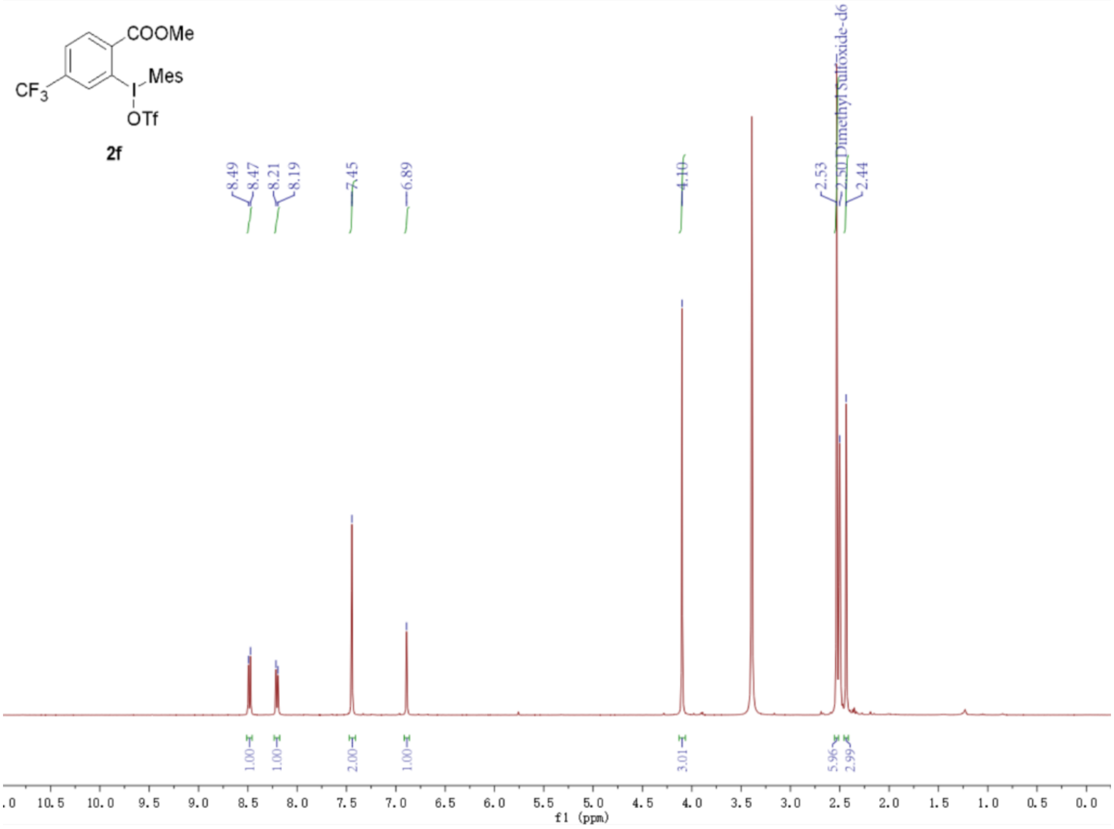
COOMe
CF_3
Mes
I
OTf
2f
8.49
8.47
8.21
8.19
7.45
6.89
4.10
2.53
2.50 Dimethyl Sulfoxide-d6
2.44
1.00
1.00
2.00
1.00
3.01
5.96
2.99
f1 (ppm)

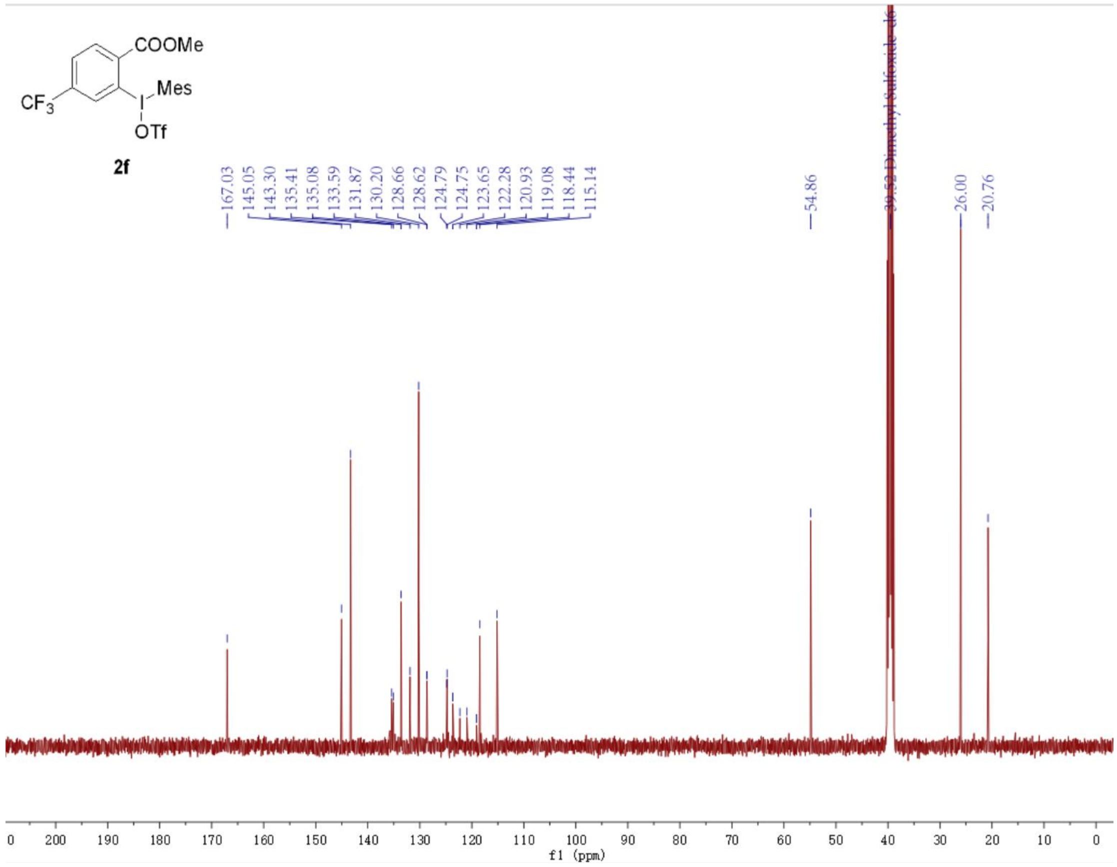
COOMe
Mes
CF3
OTf
2f
167.03
145.05
143.30
135.41
135.08
133.59
131.87
130.20
128.66
128.62
124.79
124.75
123.65
122.28
120.93
119.08
118.44
115.14
54.86
39.52 Dimethyl sulfoxide-d6
26.00
20.76
f1 (ppm)

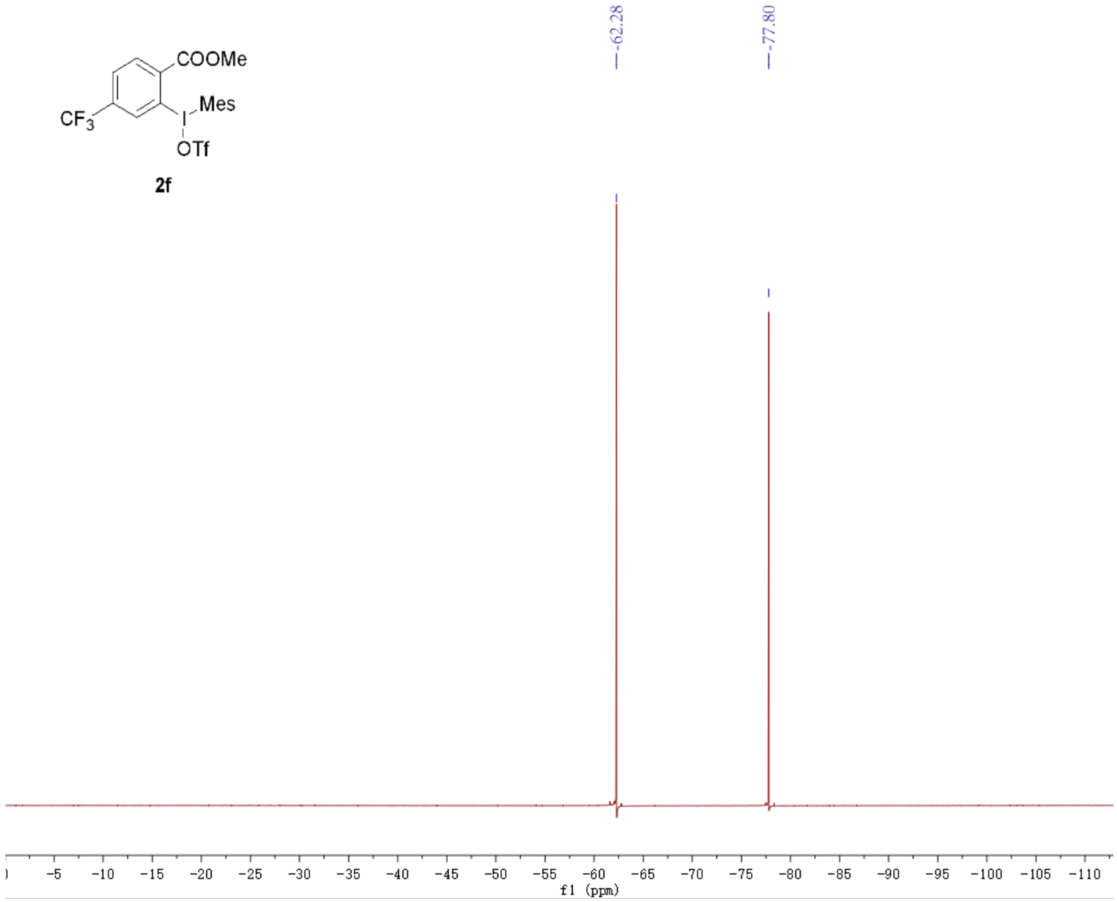
COOMe
Mes
CF3
OTf
2f
-62.28
-77.80
f1 (ppm)

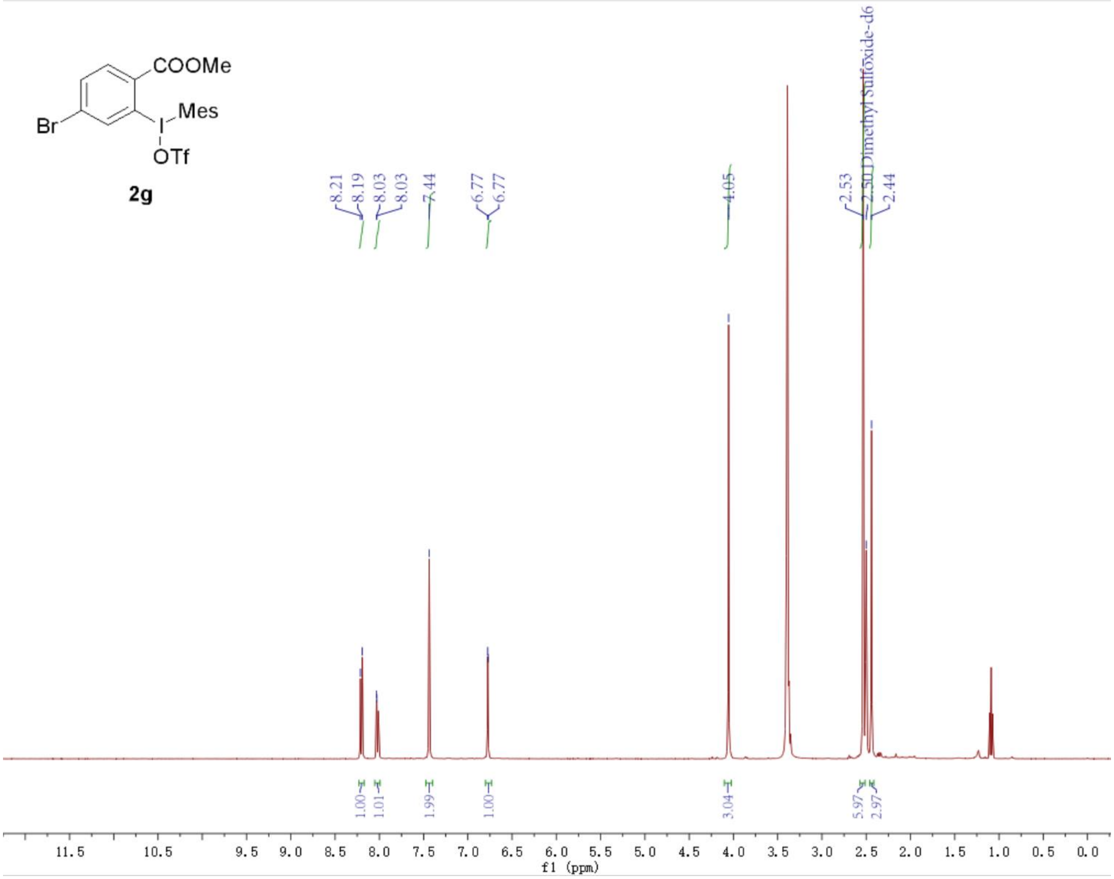
COOMe
Br
I
Mes
OTf
2g
8.21
8.19
8.03
8.03
7.44
6.77
6.77
4.05
2.53
2.50 Dimethyl Sulfoxide-d6
2.44
1.00
1.01
1.99
1.00
3.04
5.97
2.97
f1 (ppm)

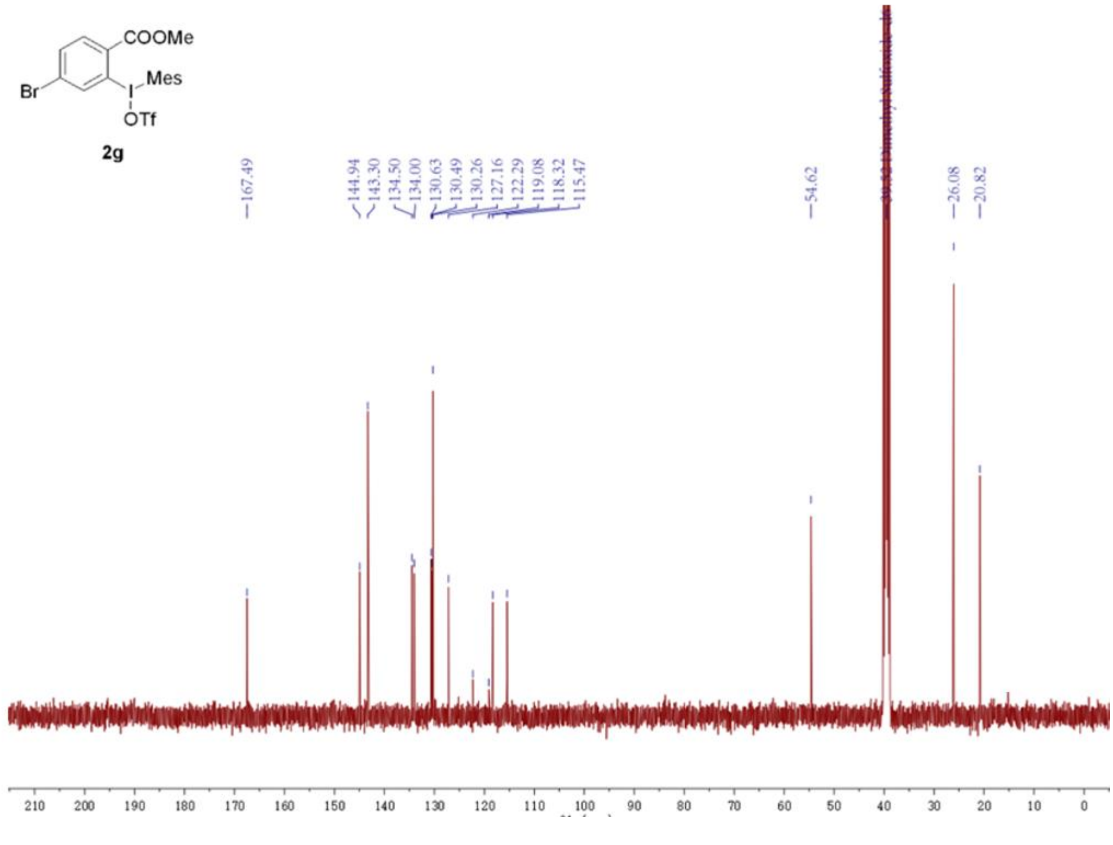
COOMe
Br
I
Mes
OTf
2g
167.49
144.94
143.30
134.50
134.00
130.63
130.49
130.26
127.16
122.29
119.08
118.32
115.47
54.62
26.08
20.82

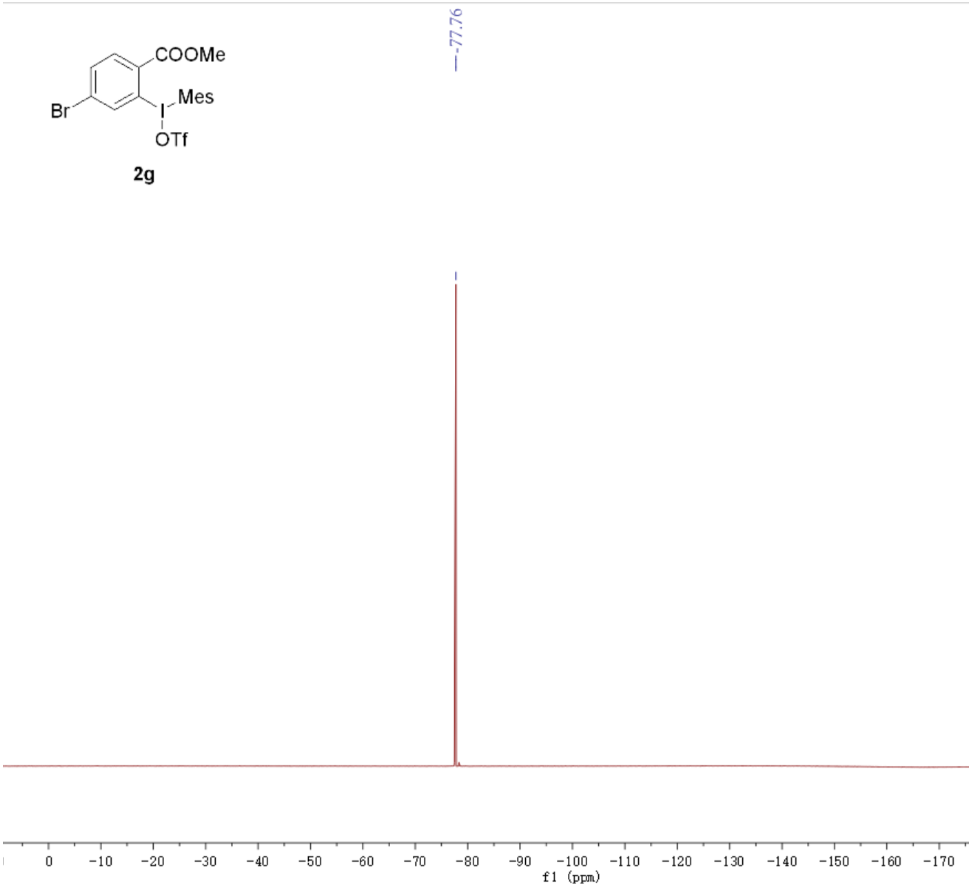
COOMe
Br
I
Mes
OTf
2g
-77.76
0 −10 −20 −30 −40 −50 −60 −70 −80 −90 −100 −110 −120 −130 −140 −150 −160 −170
f1 (ppm)

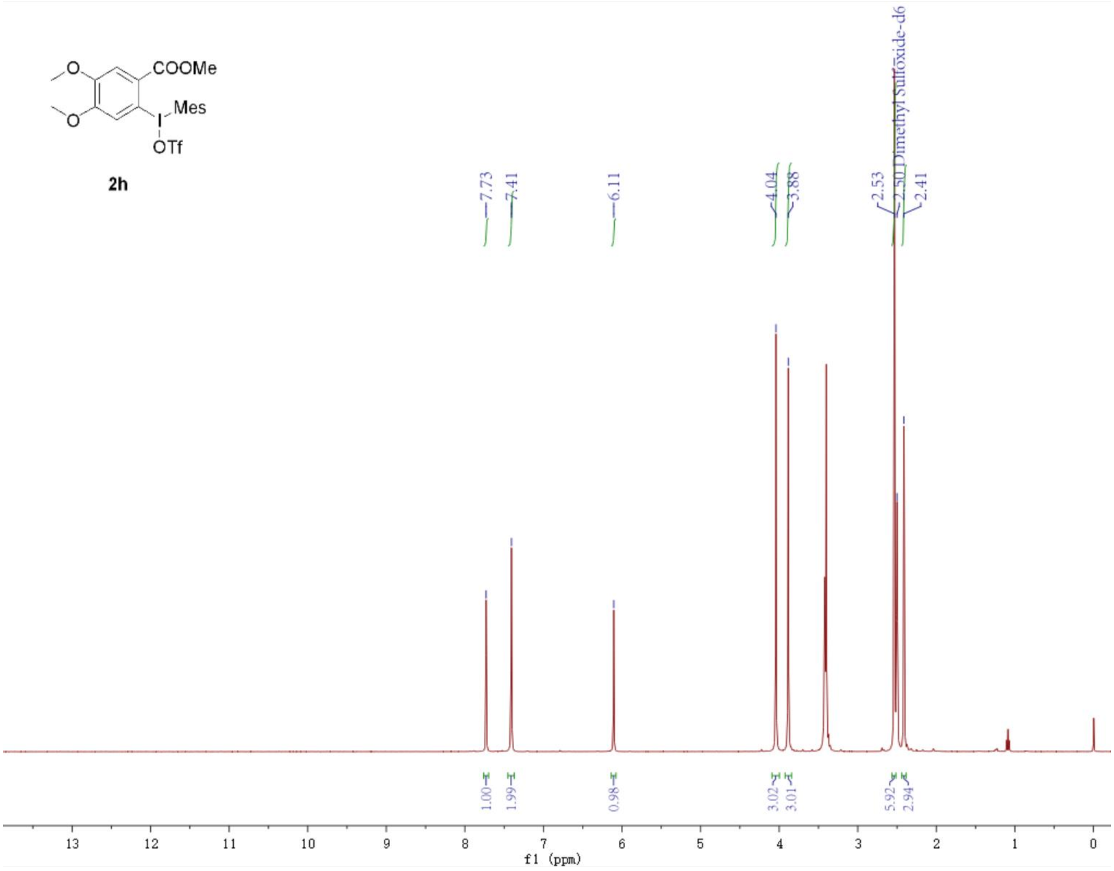
COOMe
I
Mes
OTf
2h
7.73
7.41
6.11
4.04
3.88
2.53
2.50 Dimethyl Sulfoxide-d6
2.41
1.00
1.99
0.98
3.02
3.01
5.92
2.94
13 12 11 10 9 8 7 6 5 4 3 2 1 0
f1 (ppm)

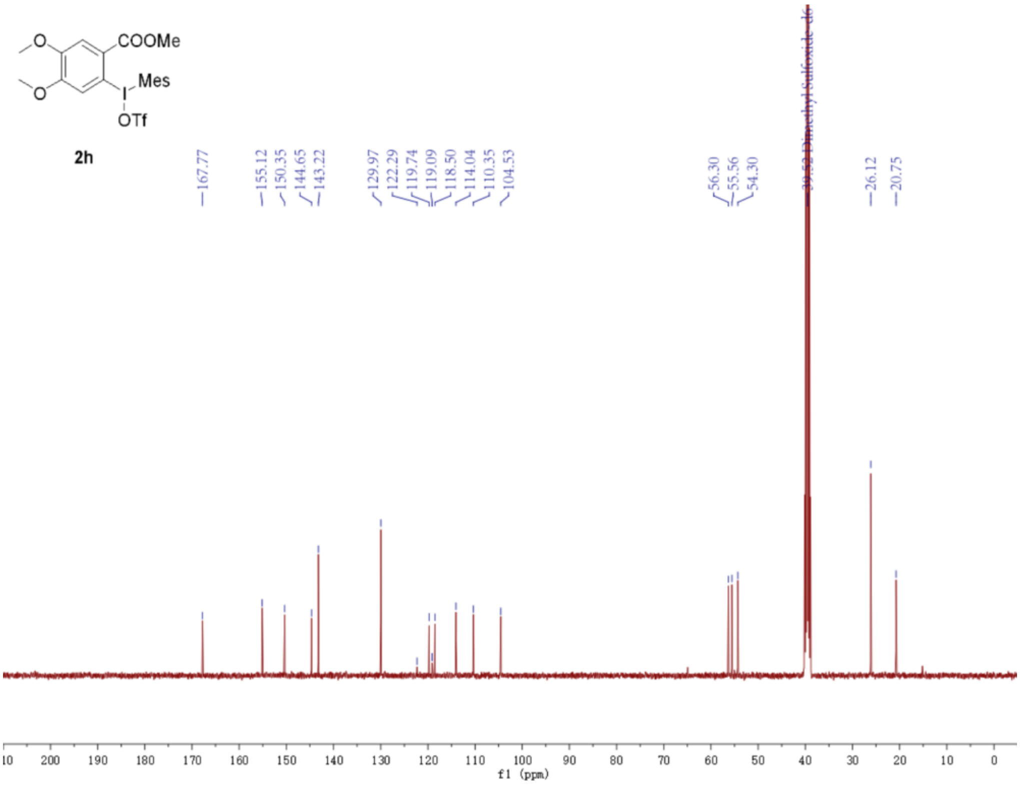
COOMe
Mes
OTf
2h
167.77
155.12
150.35
144.65
143.22
129.97
122.29
119.74
119.09
118.50
114.04
110.35
104.53
56.30
55.56
54.30
39.52 Dimethyl Sulfoxide-d6
26.12
20.75
f1 (ppm)

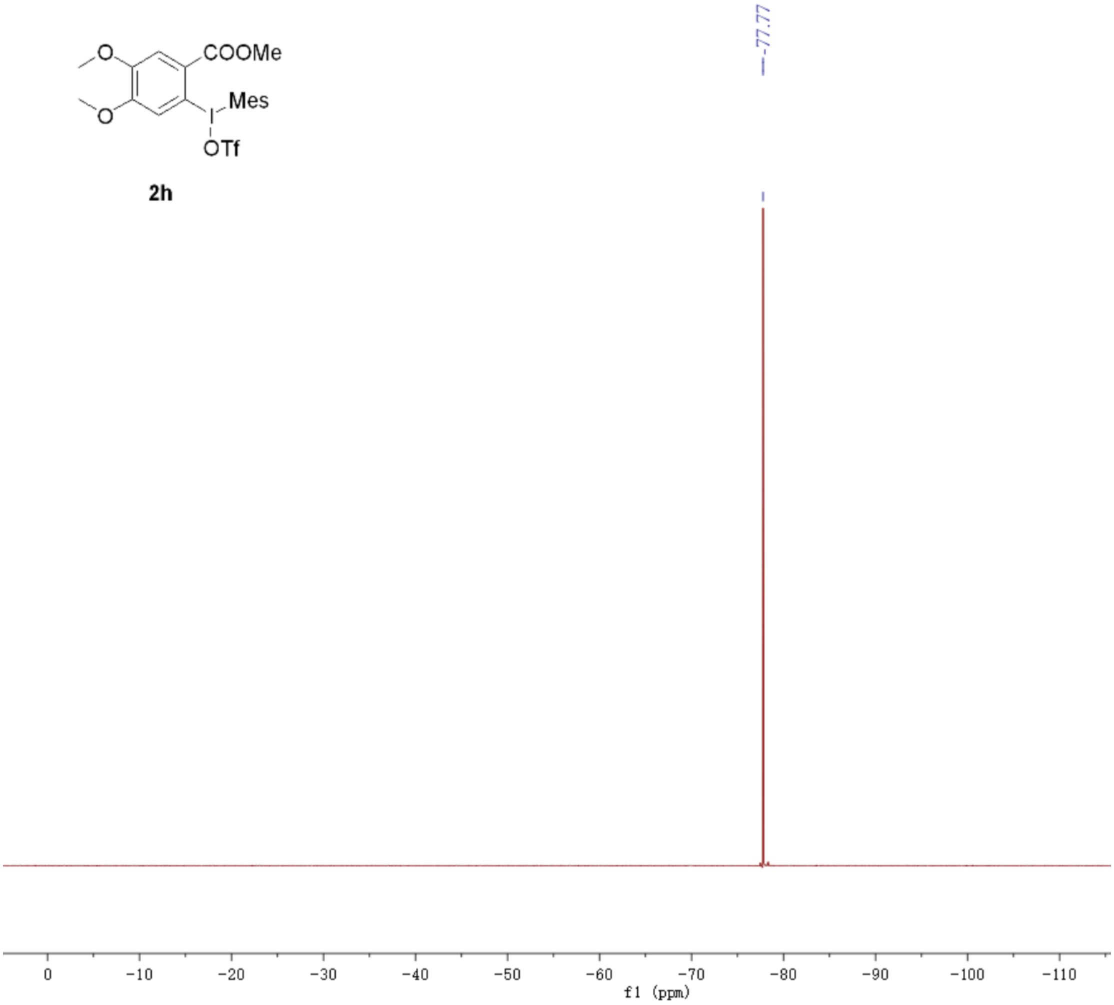
COOMe
Mes
OTf
2h
-77.77
f1 (ppm)

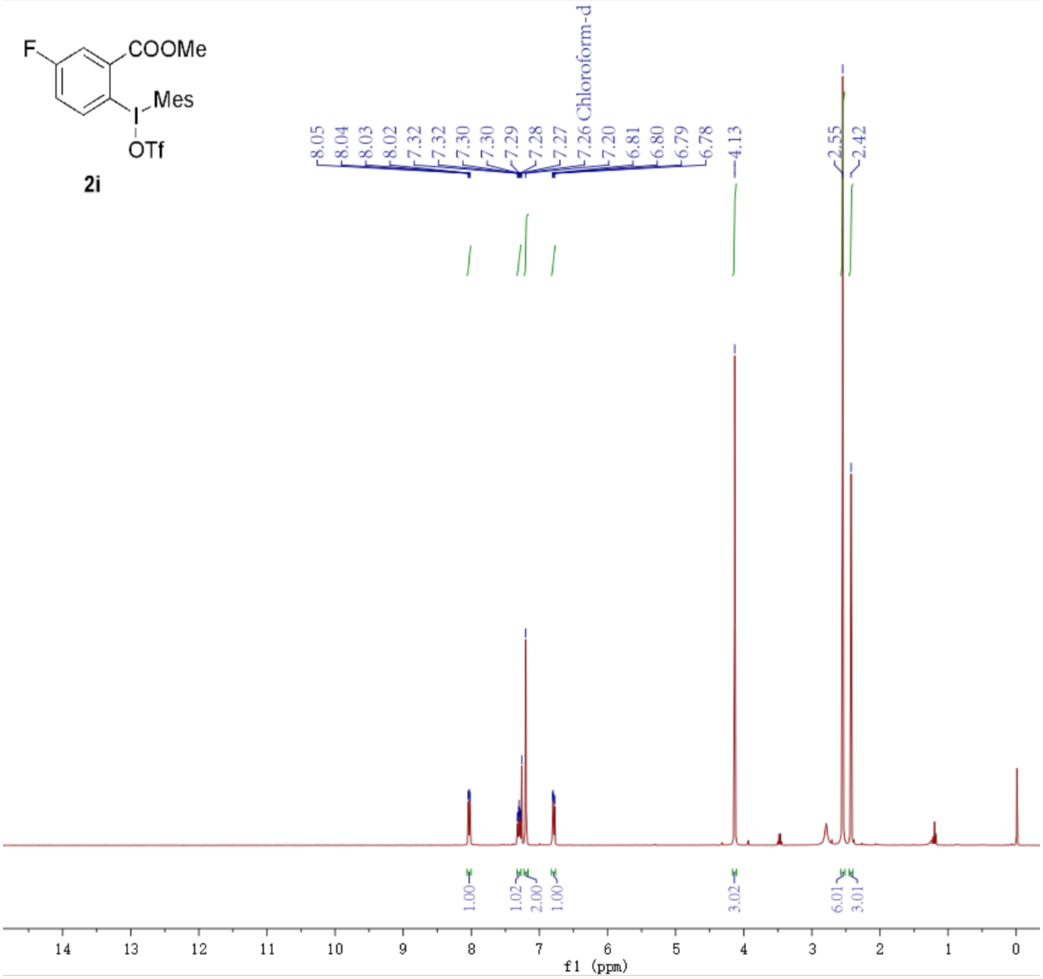
COOMe
F
Mes
OTf
2i
8.05
8.04
8.03
8.02
7.32
7.32
7.30
7.30
7.29
7.28
7.27
7.26 Chloroform-d
7.20
6.81
6.80
6.79
6.78
4.13
2.55
2.42
1.00
1.02
2.00
1.00
3.02
6.01
3.01
f1 (ppm)

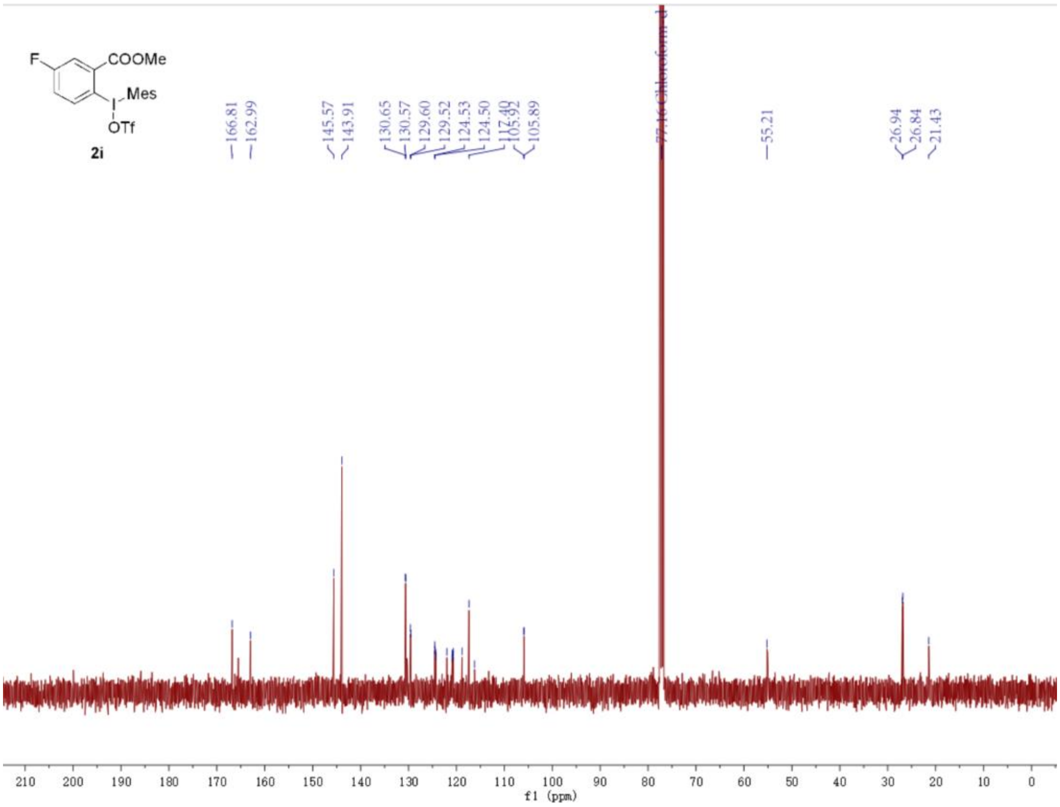
COOMe
F
Mes
OTf
2i
166.81
162.99
145.57
143.91
130.65
130.57
129.60
129.52
124.53
124.50
105.92
105.89
55.21
26.94
26.84
21.43
f1 (ppm)

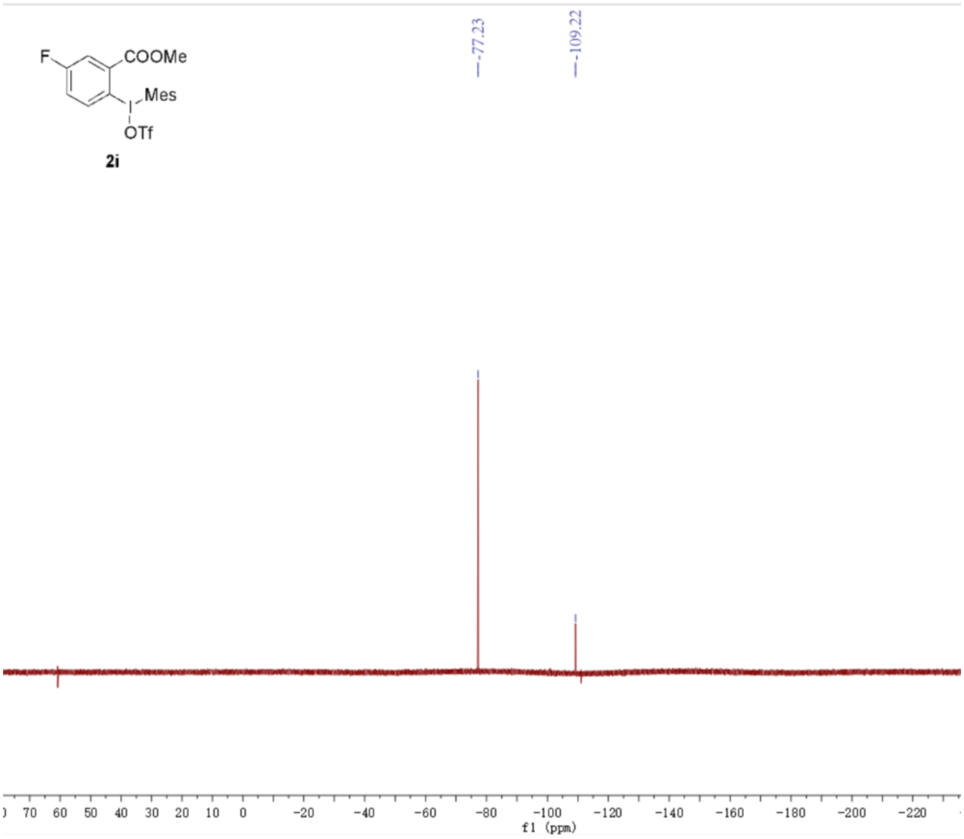
F
COOMe
Mes
I
OTf
2i
-77.23
-109.22
70 60 50 40 30 20 10 0 -20 -40 -60 -80 -100 -120 -140 -160 -180 -200 -220
f1 (ppm)

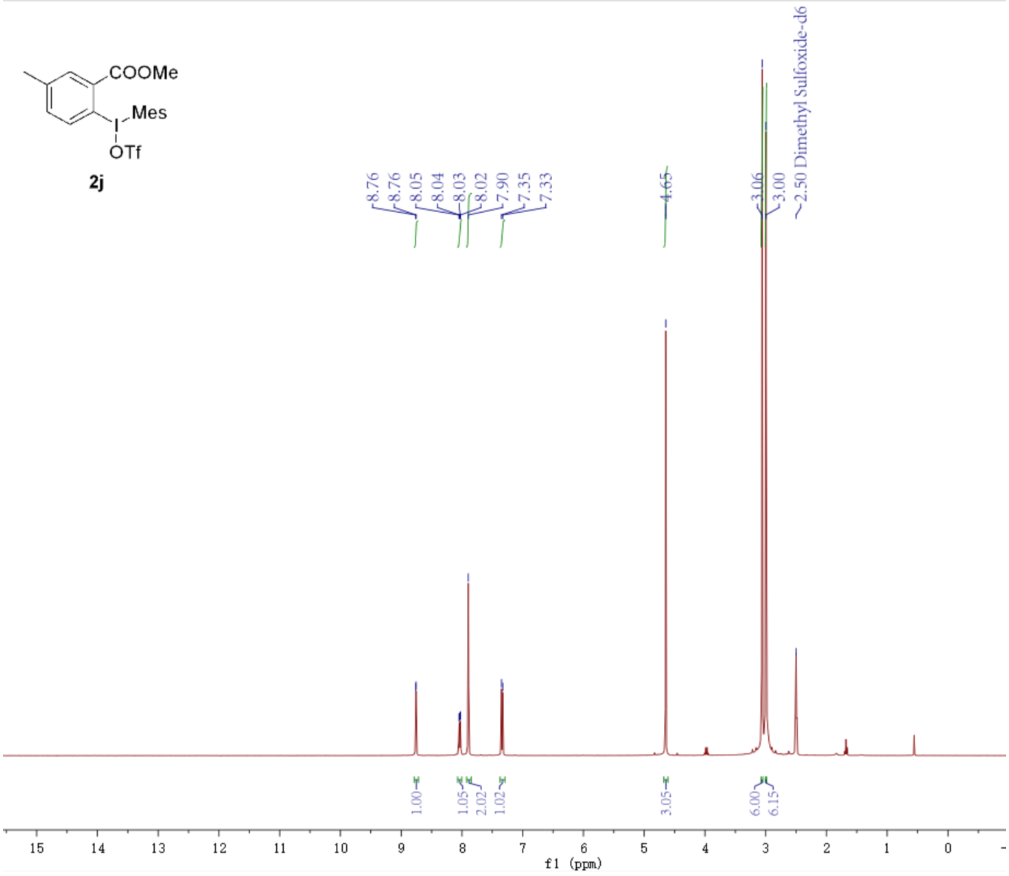
COOMe
Mes
I
OTf
2j
8.76
8.76
8.05
8.04
8.03
8.02
7.90
7.35
7.33
4.65
3.06
3.00
2.50 Dimethyl Sulfoxide-d6
1.00
1.05
2.02
1.02
3.05
6.00
6.15
15 14 13 12 11 10 9 8 7 6 5 4 3 2 1 0
f1 (ppm)

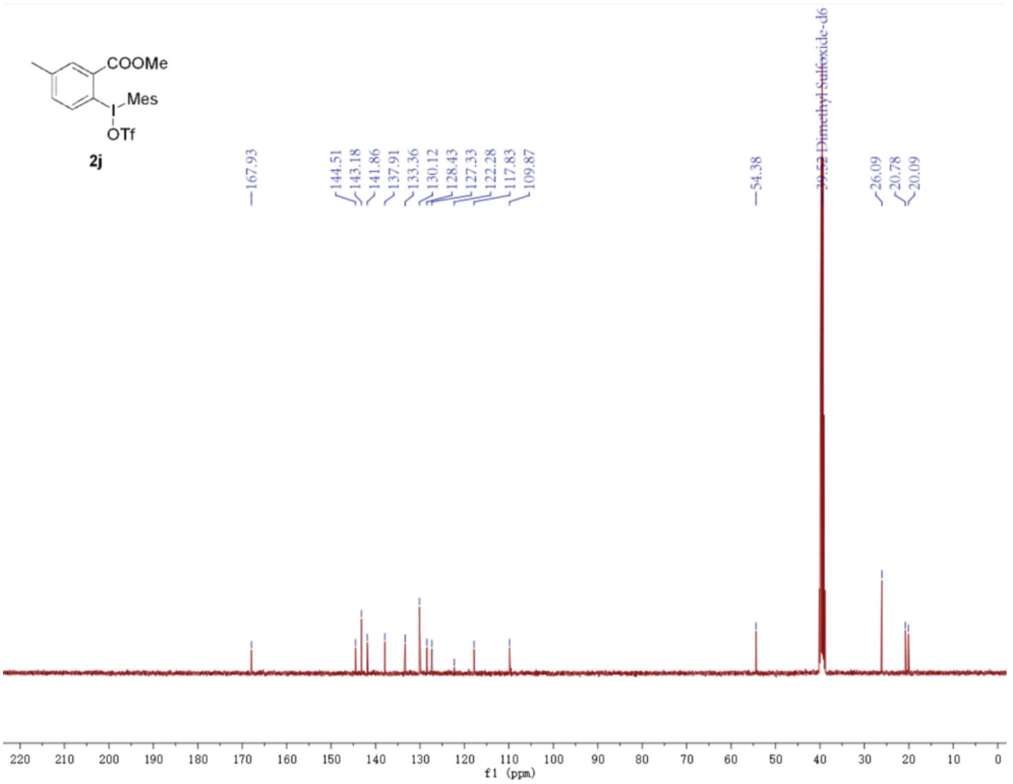
COOMe
Mes
OTf
2j
167.93
144.51
143.18
141.86
137.91
133.36
130.12
128.43
127.33
122.28
117.83
109.87
54.38
39.52 Dimethyl Sulfoxide-d6
26.09
20.78
20.09
f1 (ppm)

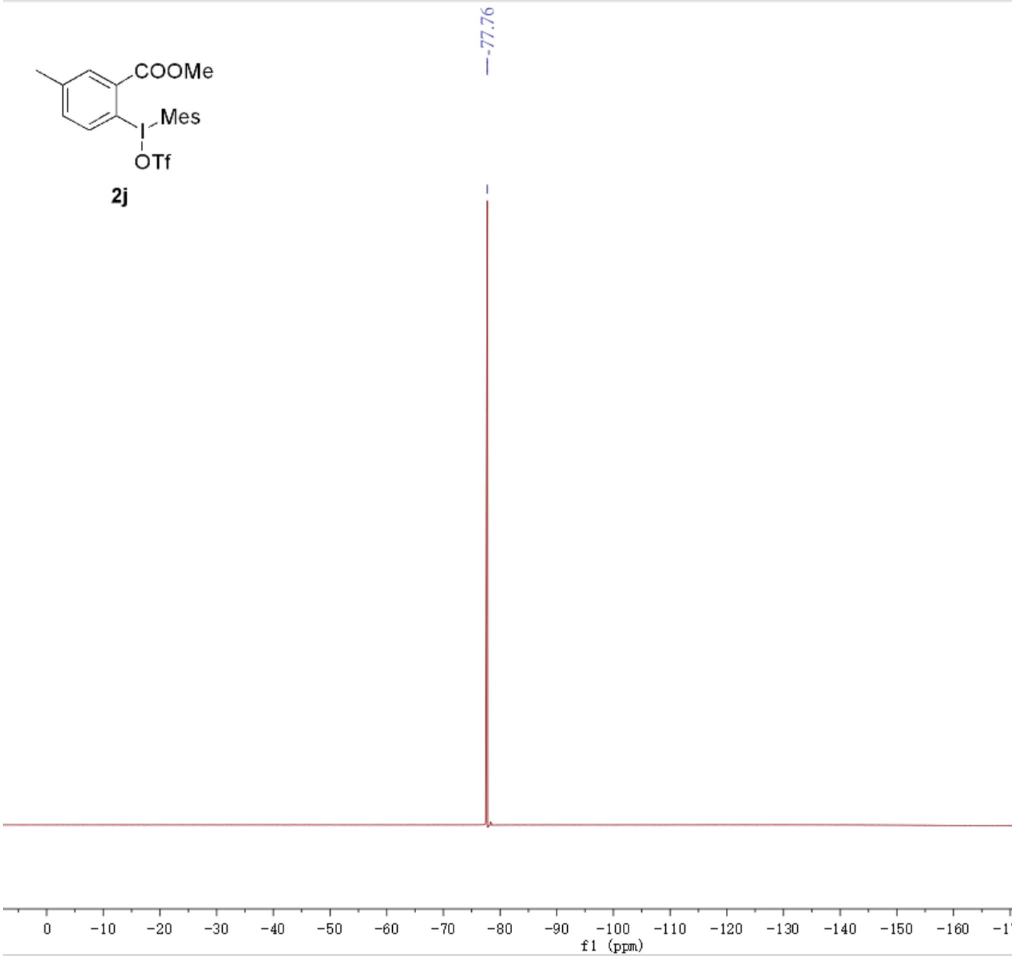
COOMe
Mes
OTf
2j
-77.76
f1 (ppm)

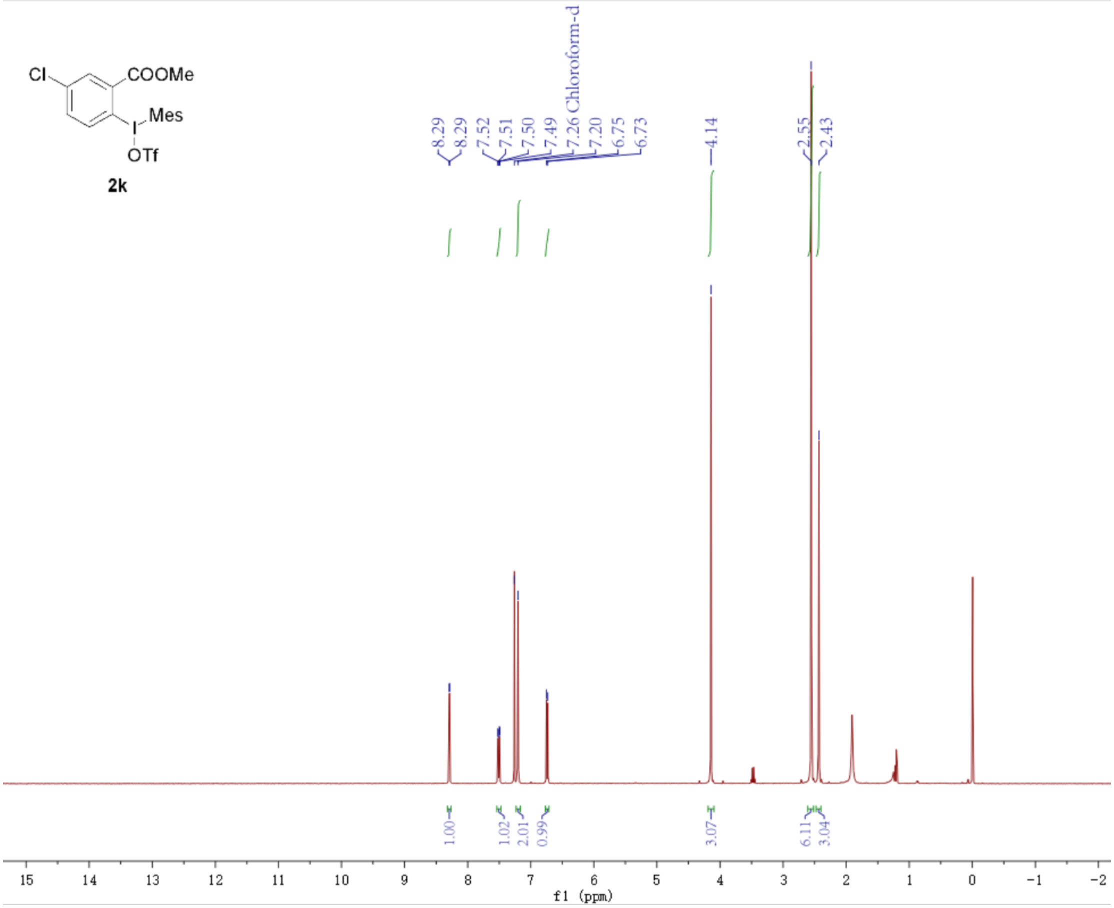

Cl
COOMe
Mes
I
OTf
8.29
8.29
7.52
7.51
7.50
7.49
7.26 Chloroform-d
7.20
6.75
6.73
4.14
2.55
2.43
1.00
1.02
2.01
0.99
3.07
6.11
3.04
15 14 13 12 11 10 9 8 7 6 5 4 3 2 1 0 -1 -2
f1 (ppm)

2k

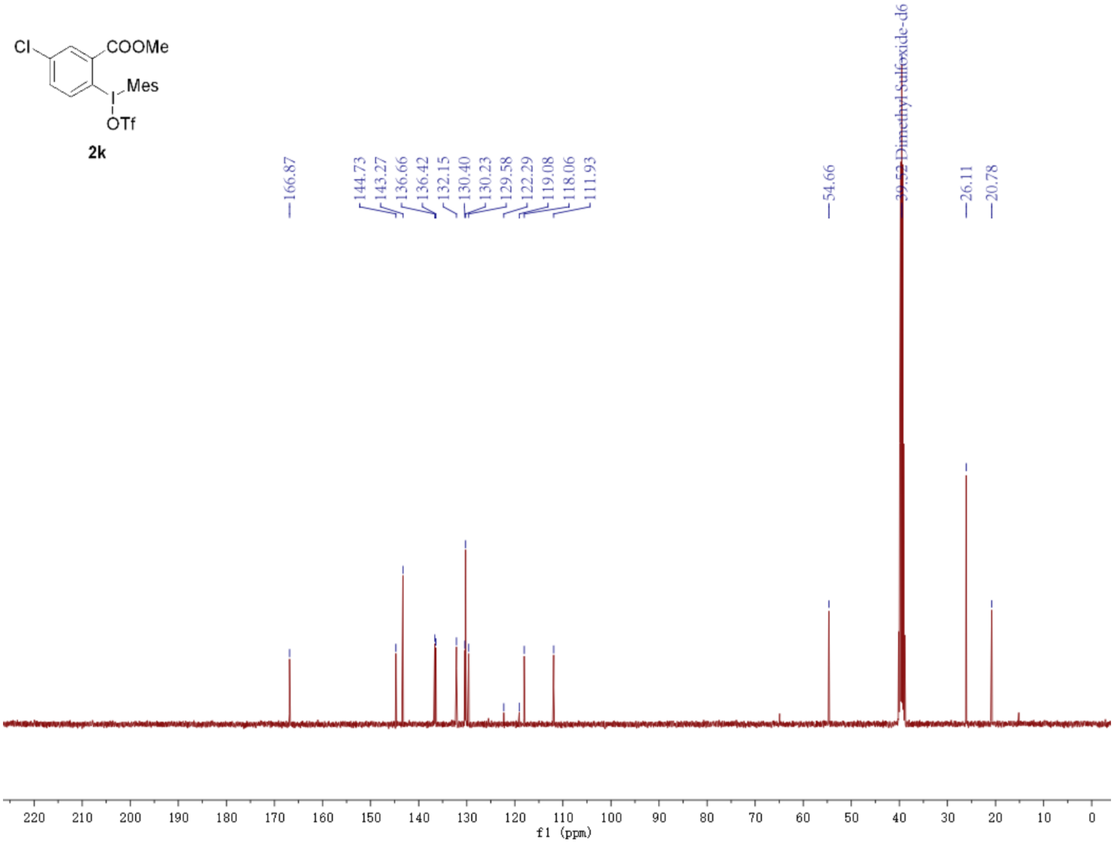

Cl
COOMe
Mes
I
OTf
166.87
144.73
143.27
136.66
136.42
132.15
130.40
130.23
129.58
122.29
119.08
118.06
111.93
54.66
39.52 Dimethyl Sulfoxide-d6
26.11
20.78
220 210 200 190 180 170 160 150 140 130 120 110 100 90 80 70 60 50 40 30 20 10 0
f1 (ppm)

2k

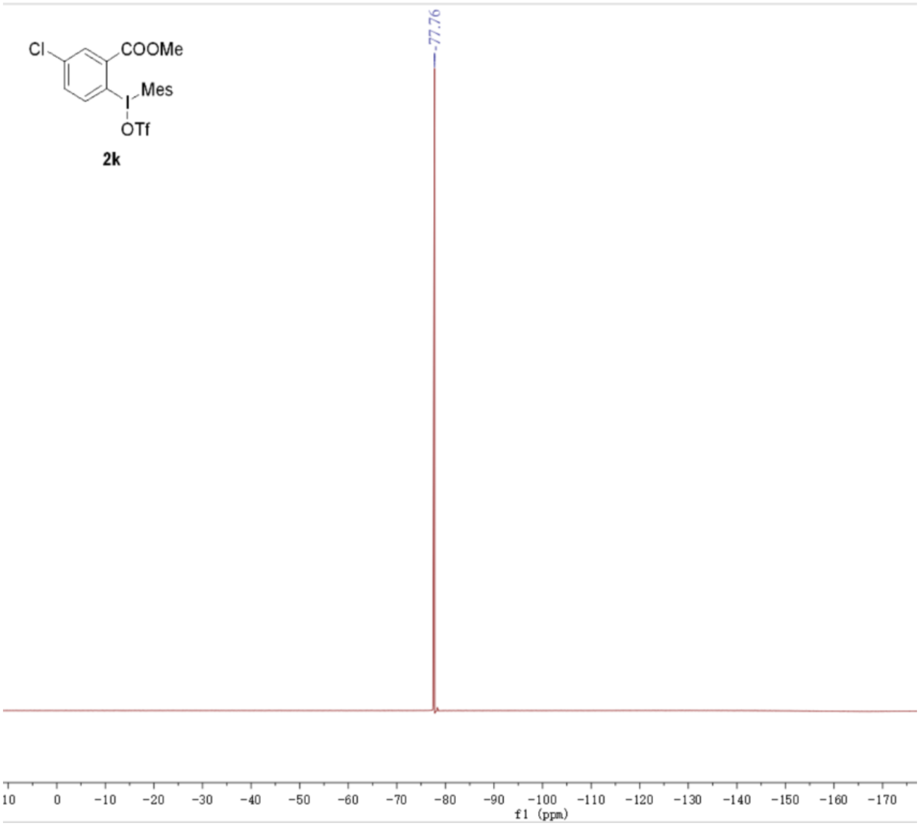

Cl
COOMe
Mes
I
OTf
2k
-77.76
10 0 −10 −20 −30 −40 −50 −60 −70 −80 −90 −100 −110 −120 −130 −140 −150 −160 −170
f1 (ppm)

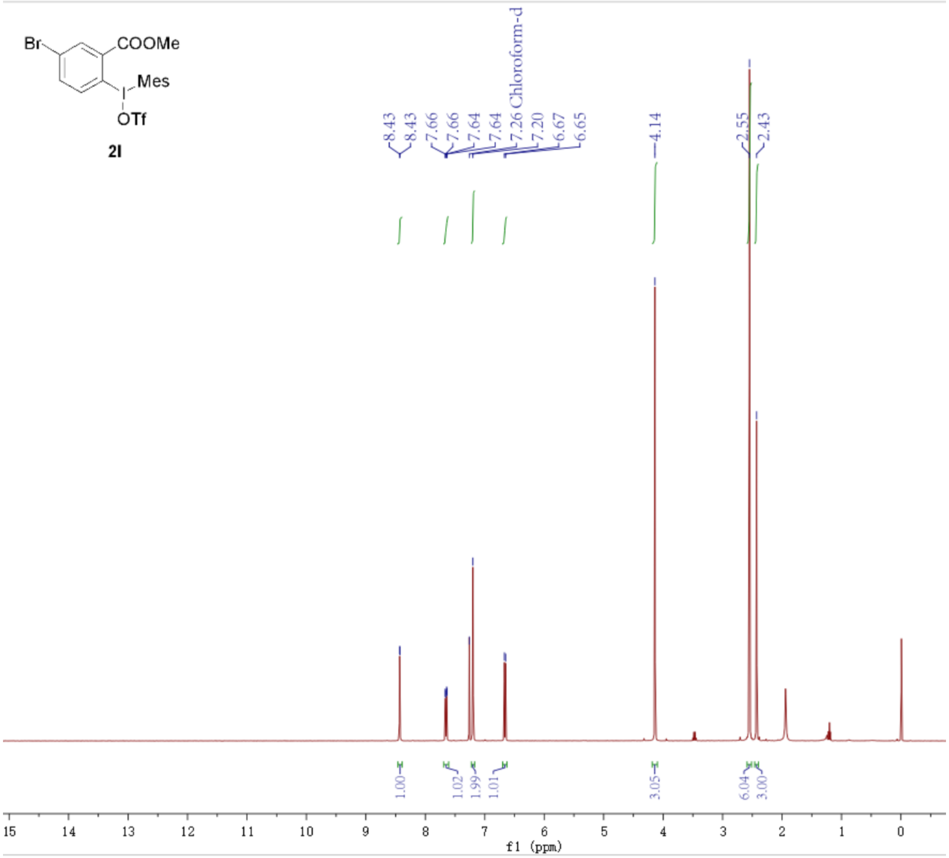

Br
COOMe
Mes
I
OTf
2l
8.43
8.43
7.66
7.66
7.64
7.64
7.26 Chloroform-d
7.20
6.67
6.65
4.14
2.55
2.43
1.00
1.02
1.99
1.01
3.05
6.04
3.00
15 14 13 12 11 10 9 8 7 6 5 4 3 2 1 0
f1 (ppm)

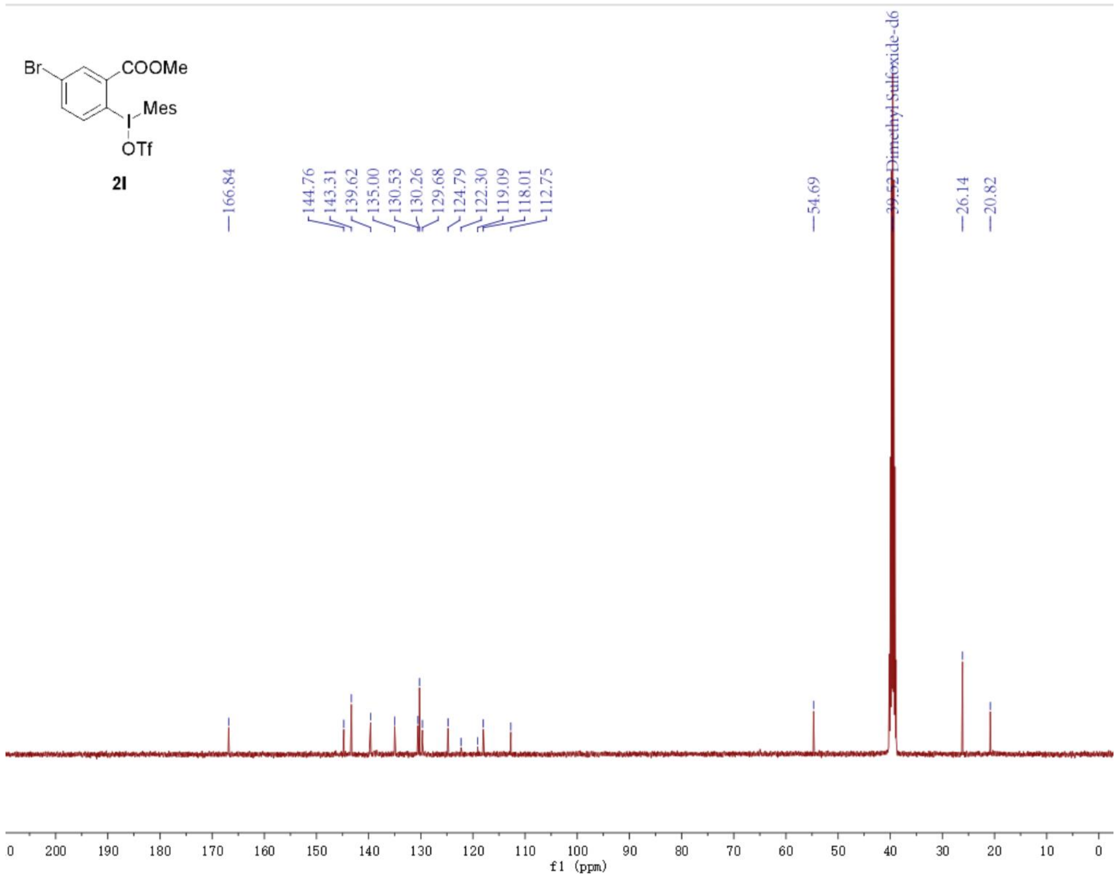
Br
COOMe
Mes
I
OTf
2l
166.84
144.76
143.31
139.62
135.00
130.53
130.26
129.68
124.79
122.30
119.09
118.01
112.75
54.69
39.52 Dimethyl Sulfoxide-d6
26.14
20.82
f1 (ppm)

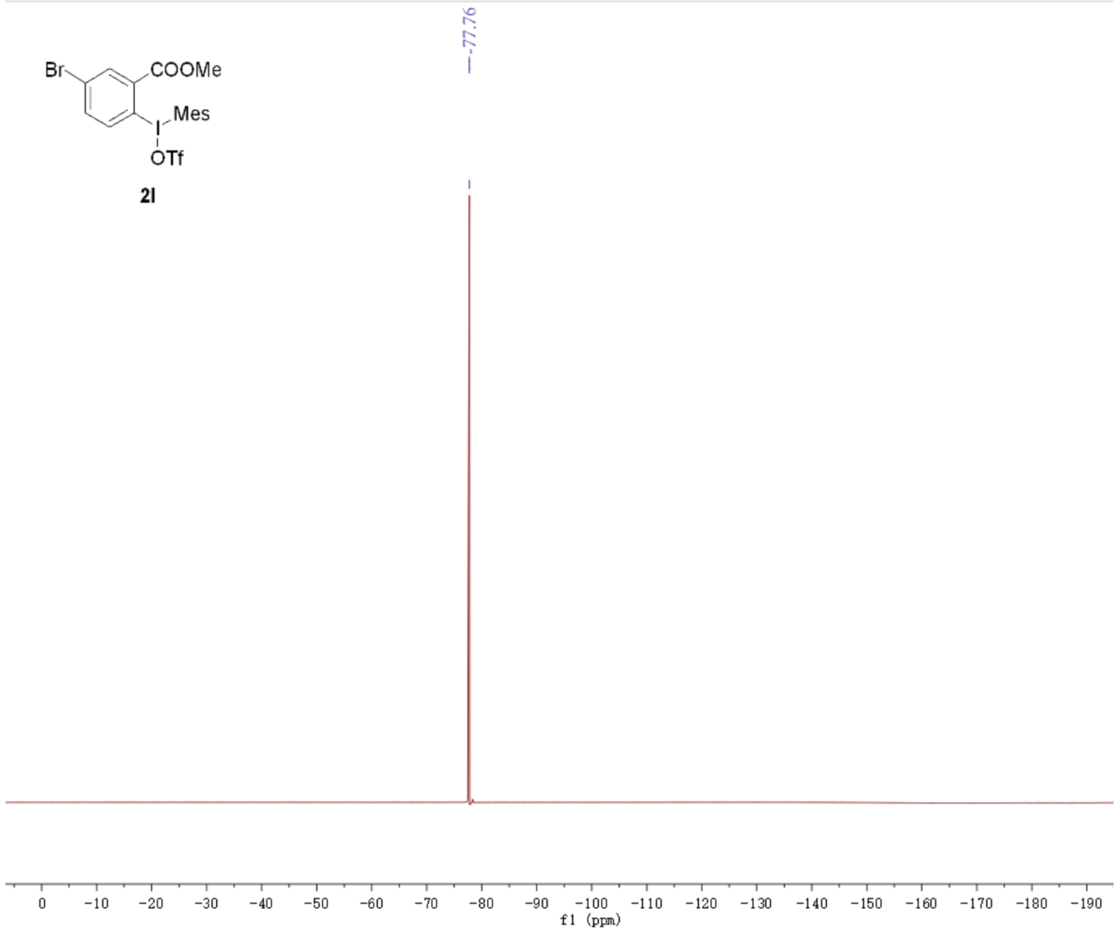
Br
COOMe
Mes
I
OTf
2l
-77.76
f1 (ppm)

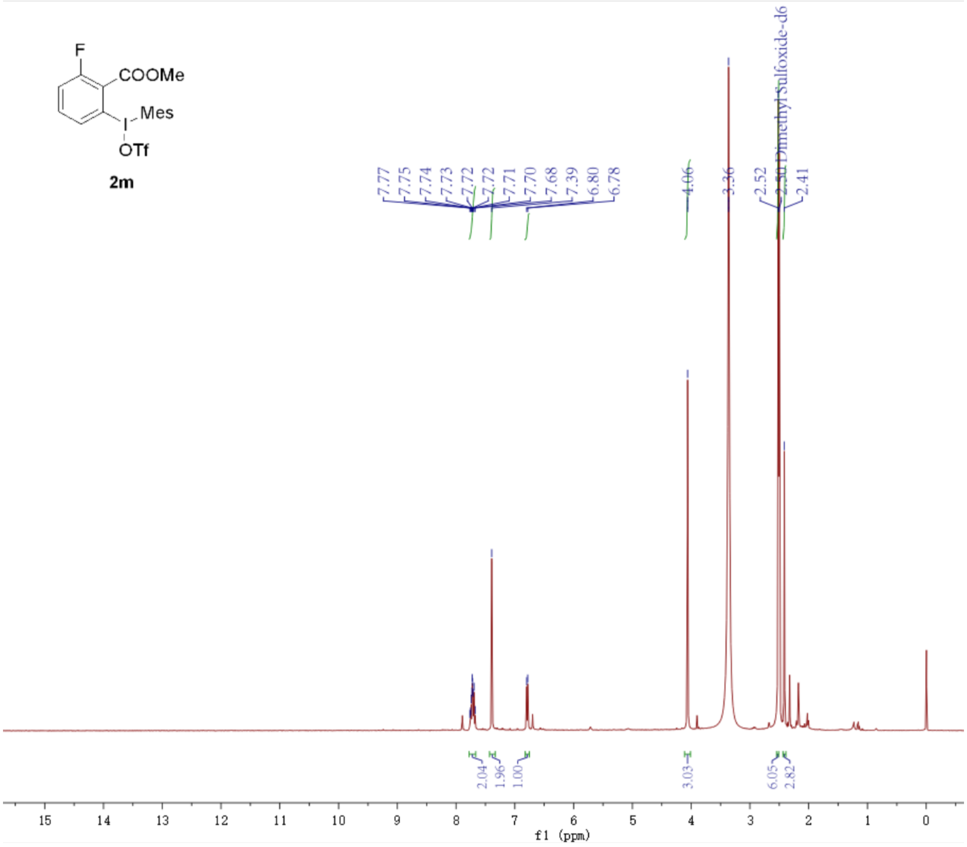
F
COOMe
Mes
OTf
2m
7.77
7.75
7.74
7.73
7.72
7.72
7.71
7.70
7.68
7.39
6.80
6.78
4.06
3.36
2.52
2.50 Dimethyl Sulfoxide-d6
2.41
2.04
1.96
1.00
3.03
6.05
2.82
15 14 13 12 11 10 9 8 7 6 5 4 3 2 1 0
f1 (ppm)

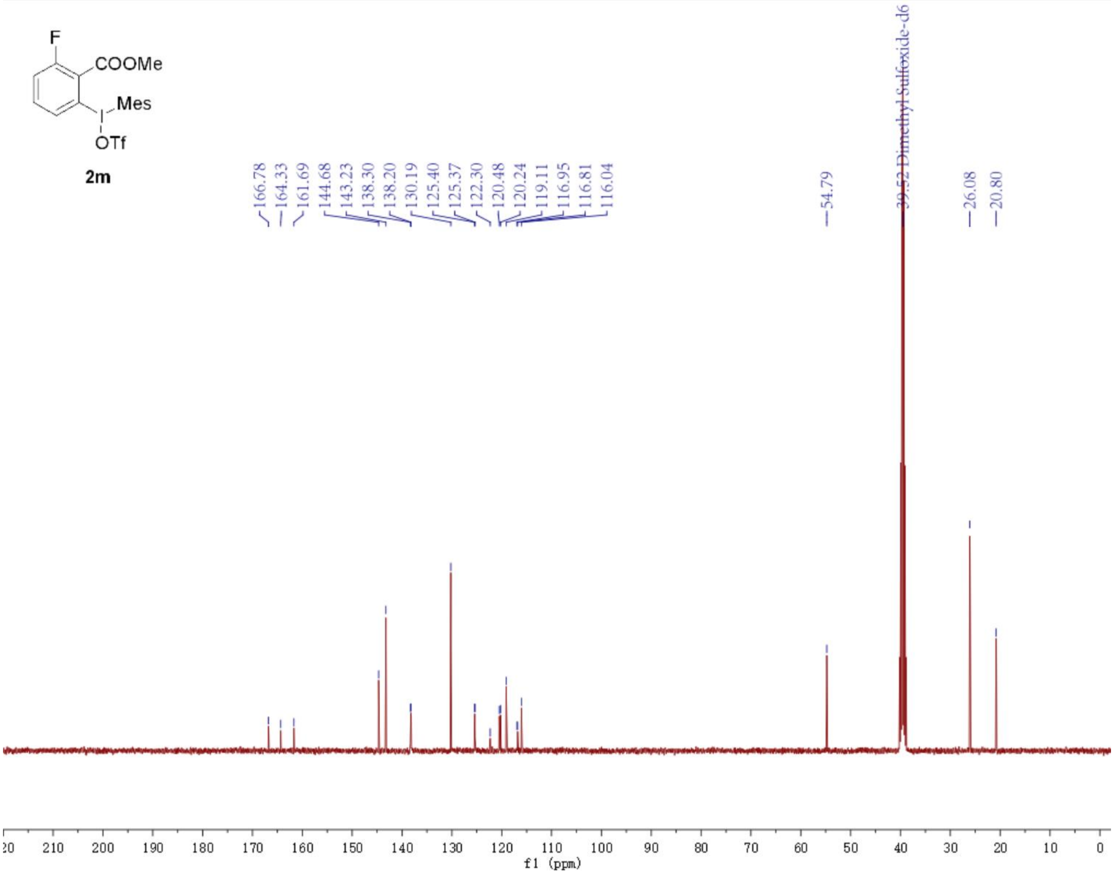
F
COOMe
Mes
OTf
2m
166.78
164.33
161.69
144.68
143.23
138.30
138.20
130.19
125.40
125.37
122.30
120.48
120.24
119.11
116.95
116.81
116.04
54.79
39.52 Dimethyl Sulfoxide-d6
26.08
20.80
20 210 200 190 180 170 160 150 140 130 120 110 100 90 80 70 60 50 40 30 20 10 0
f1 (ppm)

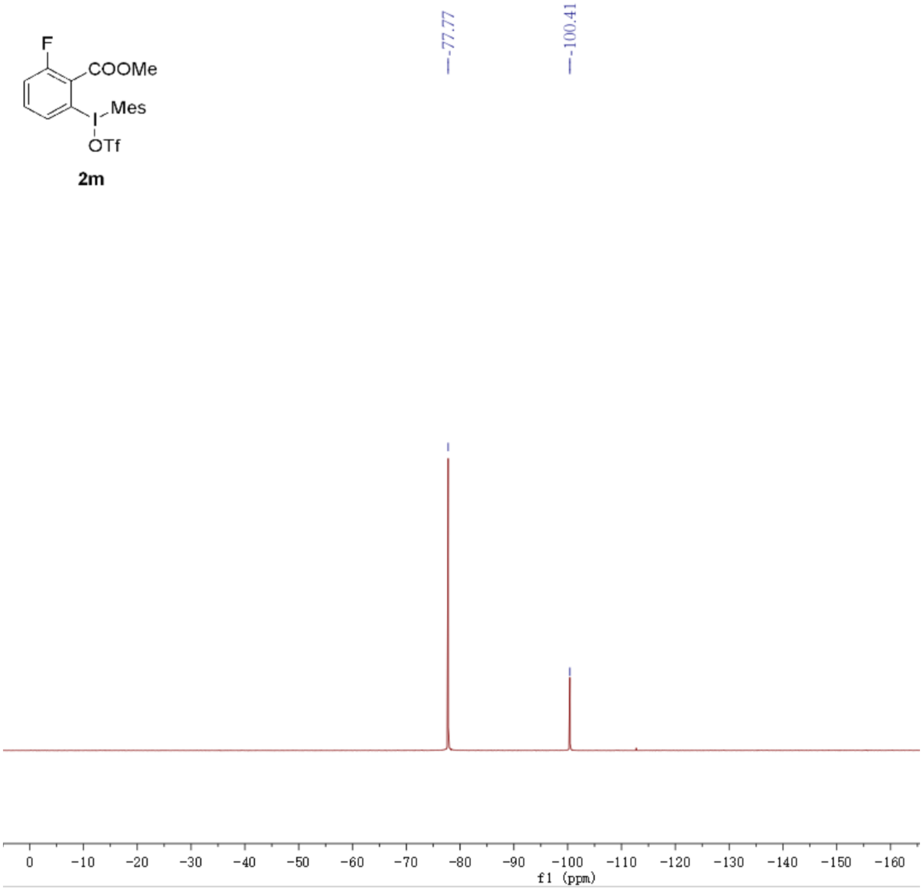
-77.77
-100.41
f1 (ppm)

2m

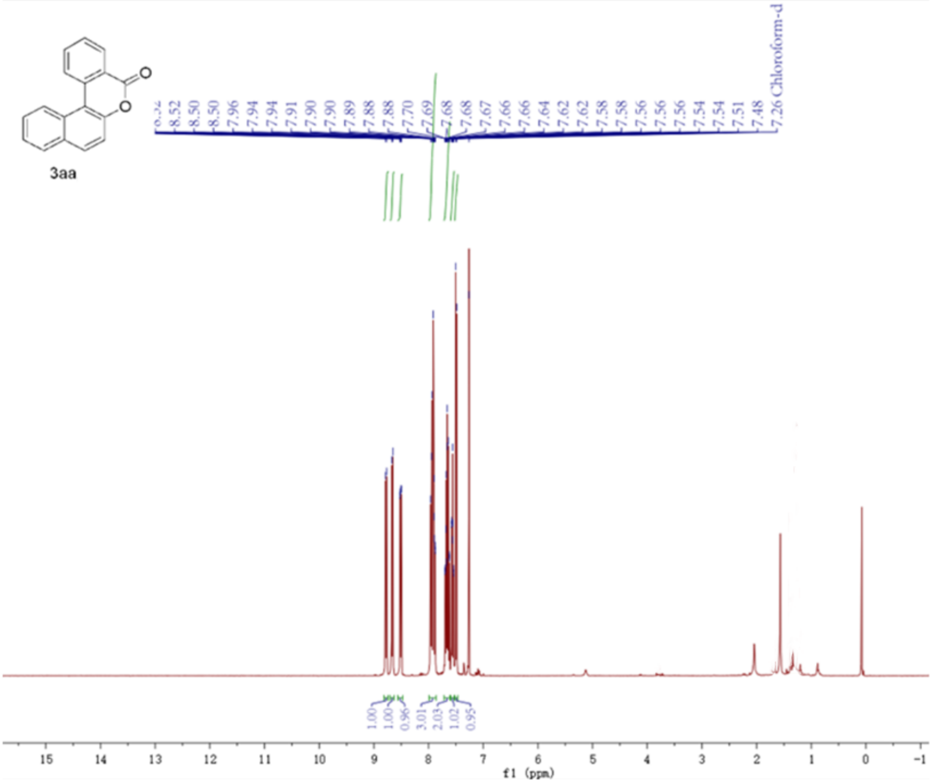
8.52
8.50
8.50
7.96
7.94
7.94
7.91
7.90
7.90
7.89
7.88
7.88
7.70
7.69
7.68
7.68
7.67
7.66
7.66
7.64
7.62
7.62
7.58
7.58
7.56
7.56
7.56
7.54
7.54
7.51
7.48
7.26 Chloroform-d
1.00
1.00
0.96
3.01
2.03
1.02
0.95
f1 (ppm)

3aa

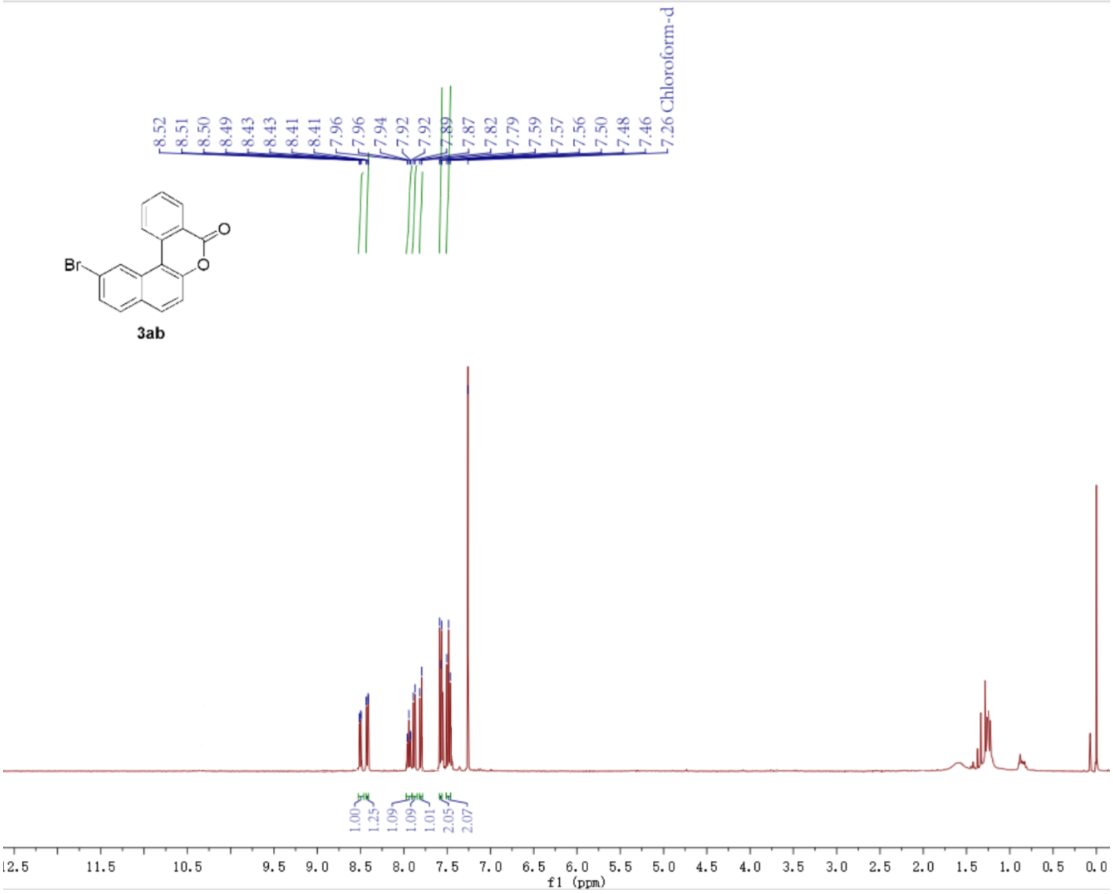
8.52
8.51
8.50
8.49
8.43
8.43
8.41
8.41
7.96
7.96
7.94
7.92
7.92
7.89
7.87
7.82
7.79
7.59
7.57
7.56
7.50
7.48
7.46
7.26 Chloroform-d
Br
O
O
3ab
1.00
1.25
1.09
1.09
1.01
2.05
2.07
f1 (ppm)

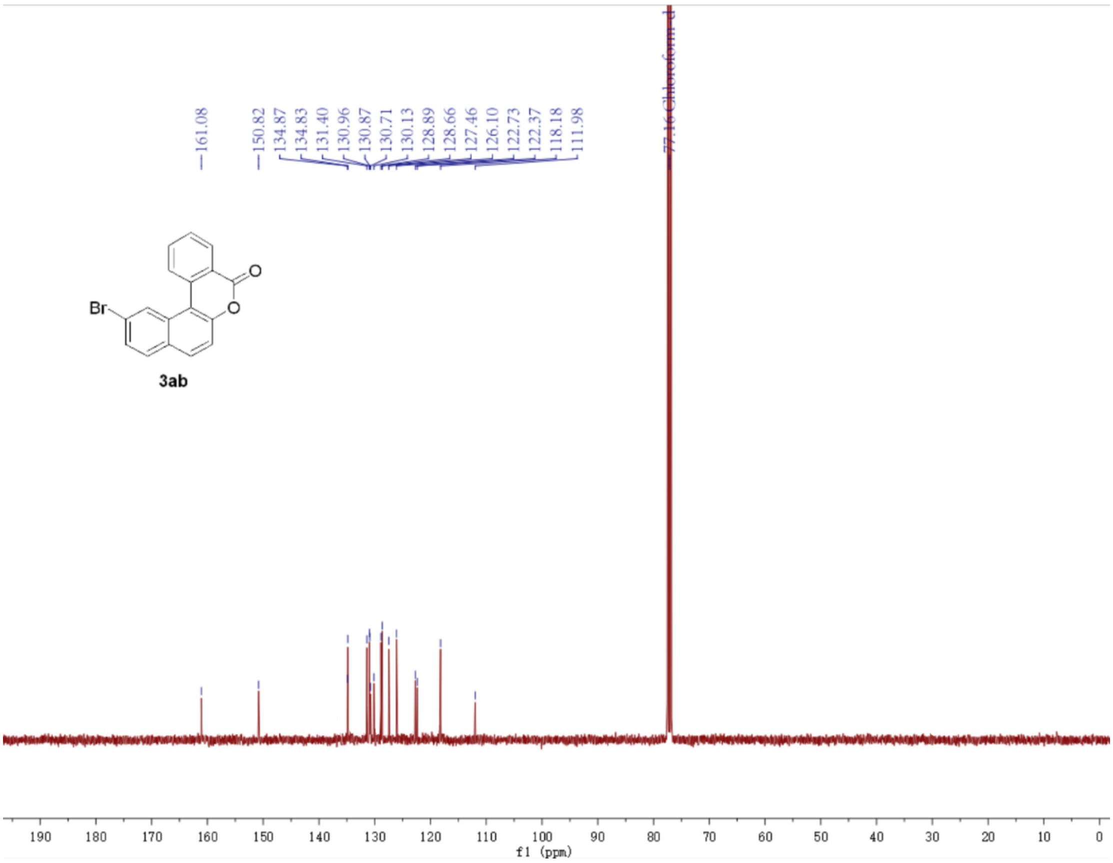
161.08
150.82
134.87
134.83
131.40
130.96
130.87
130.71
130.13
128.89
128.66
127.46
126.10
122.73
122.37
118.18
111.98
77.16 Chloroform-d
Br
O
O
3ab
f1 (ppm)

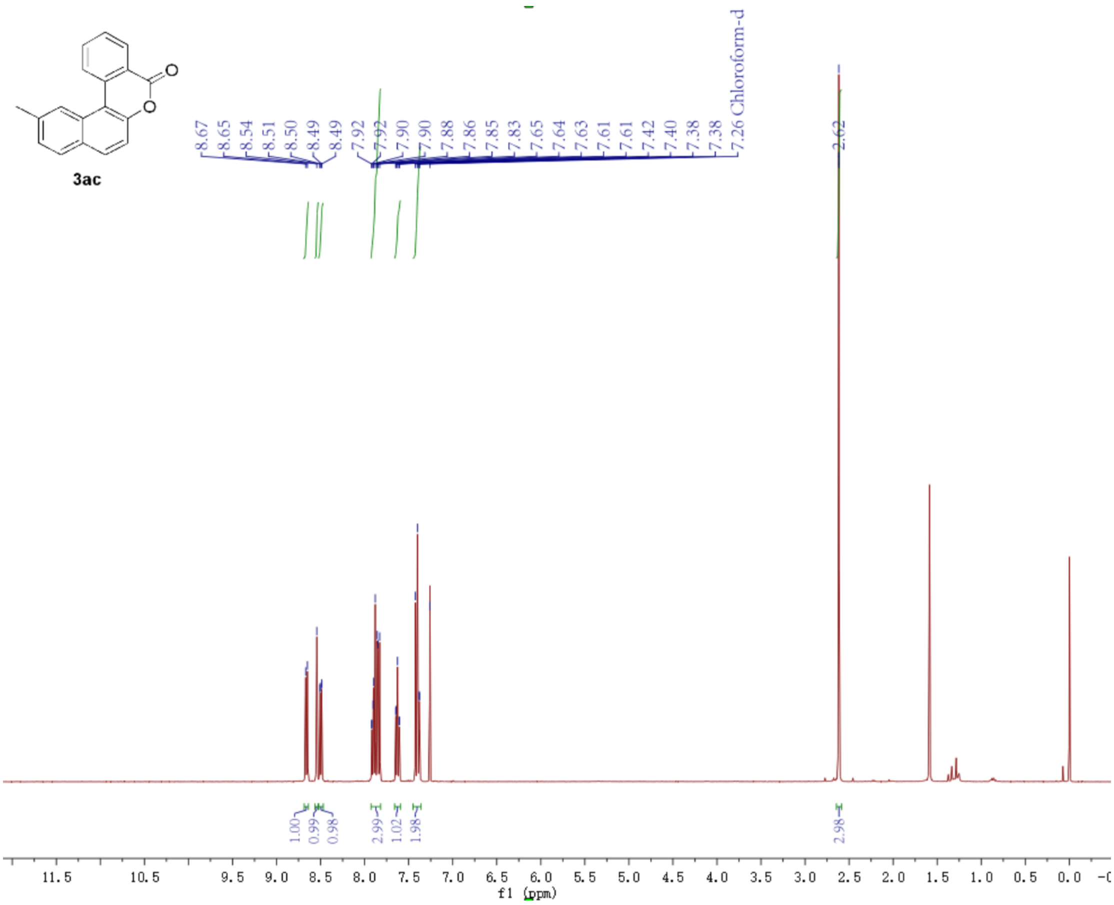
3ac
8.67
8.65
8.54
8.51
8.50
8.49
8.49
7.92
7.92
7.90
7.90
7.88
7.86
7.85
7.83
7.65
7.64
7.63
7.61
7.61
7.42
7.40
7.38
7.38
7.26 Chloroform-d
2.62
1.00
0.99
0.98
2.99
1.02
1.98
2.98
f1 (ppm)

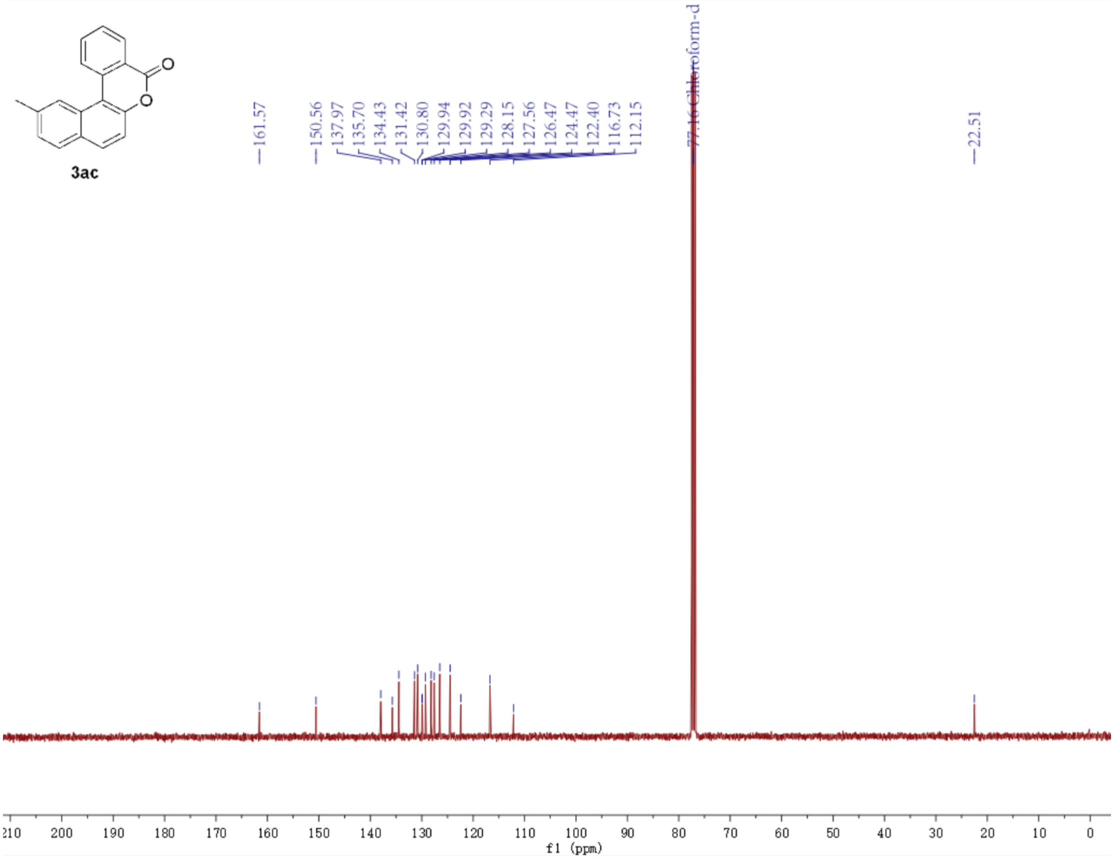
3ac
161.57
150.56
137.97
135.70
134.43
131.42
130.80
129.94
129.92
129.29
128.15
127.56
126.47
124.47
122.40
116.73
112.15
77.16 Chloroform-d
22.51
f1 (ppm)

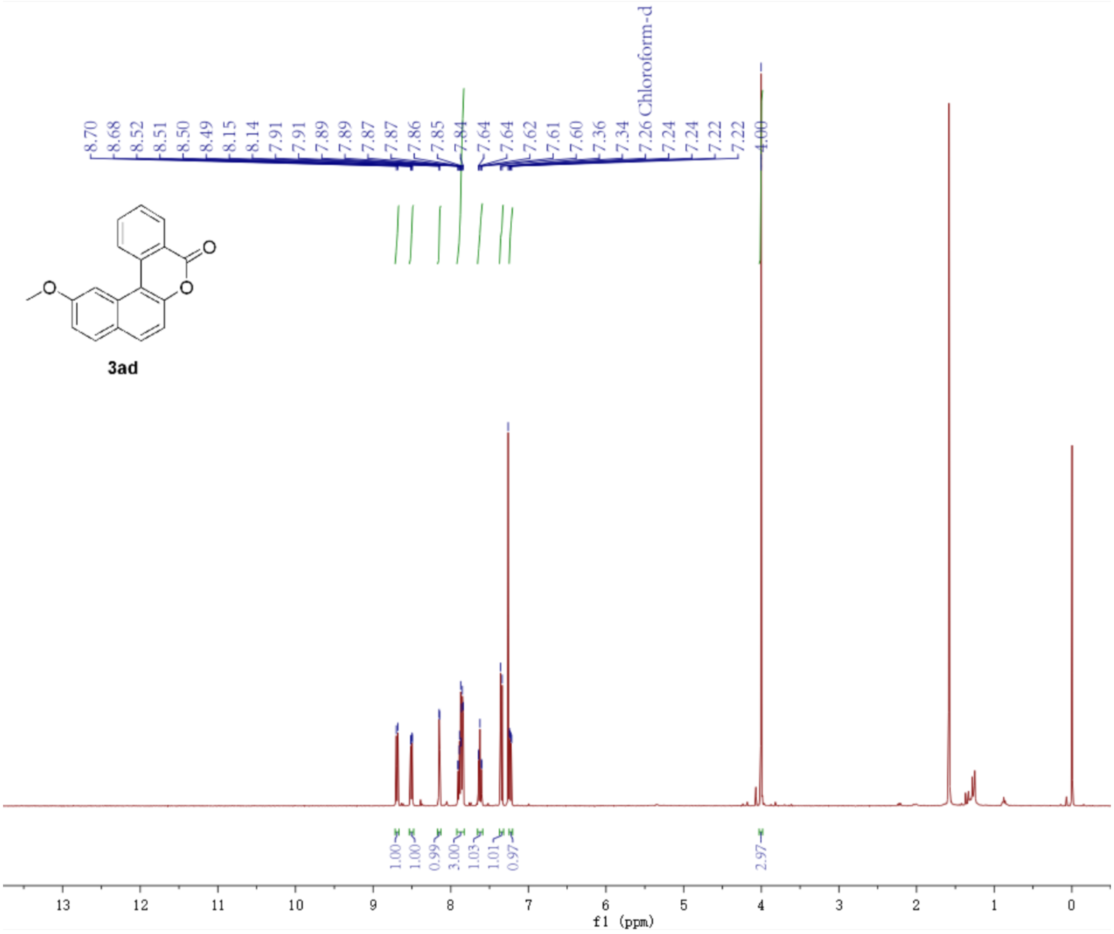
8.70
8.68
8.52
8.51
8.50
8.49
8.15
8.14
7.91
7.91
7.89
7.89
7.87
7.87
7.86
7.85
7.84
7.64
7.64
7.62
7.61
7.60
7.36
7.34
7.26 Chloroform-d
7.24
7.24
7.22
7.22
4.00
3ad
1.00
1.00
0.99
3.00
1.03
1.01
0.97
2.97
13
12
11
10
9
8
7
6
5
4
3
2
1
0
f1 (ppm)

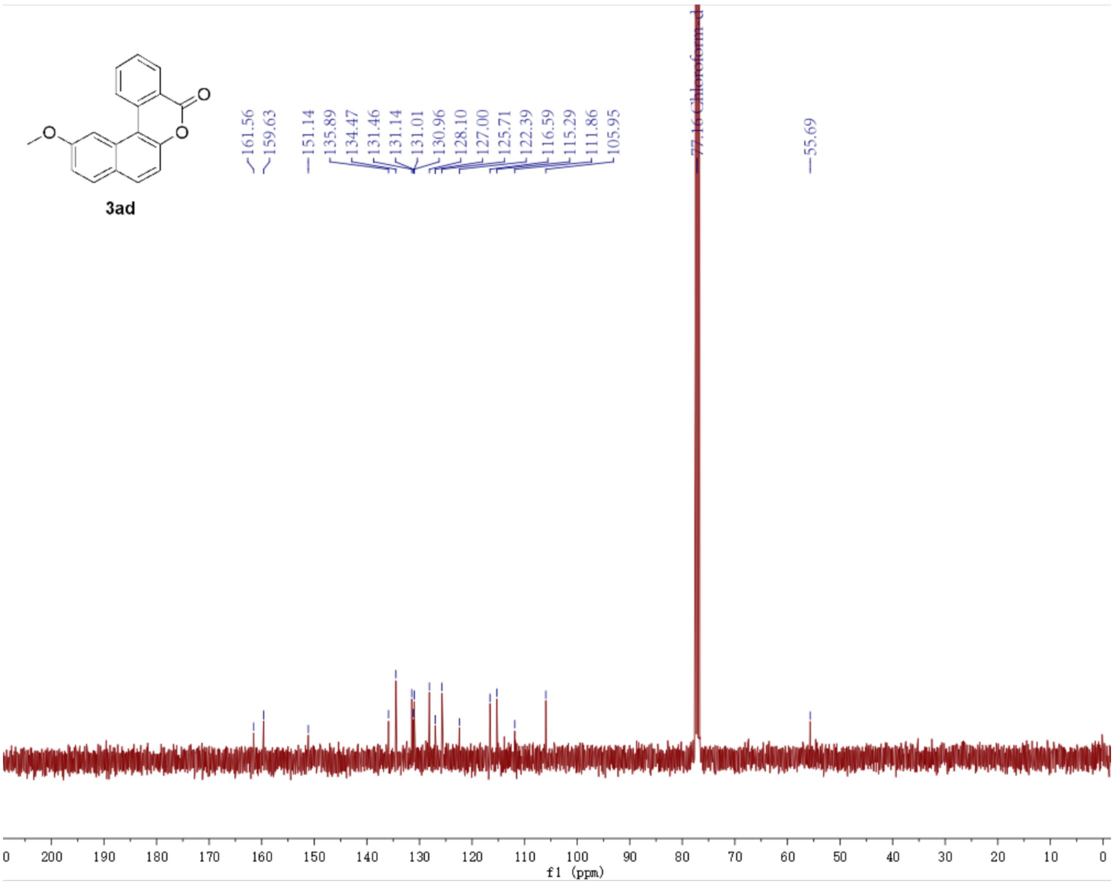
3ad
161.56
159.63
151.14
135.89
134.47
131.46
131.14
131.01
130.96
128.10
127.00
125.71
122.39
116.59
115.29
111.86
105.95
77.16 Chloroform-d
55.69
200
190
180
170
160
150
140
130
120
110
100
90
80
70
60
50
40
30
20
10
0
f1 (ppm)

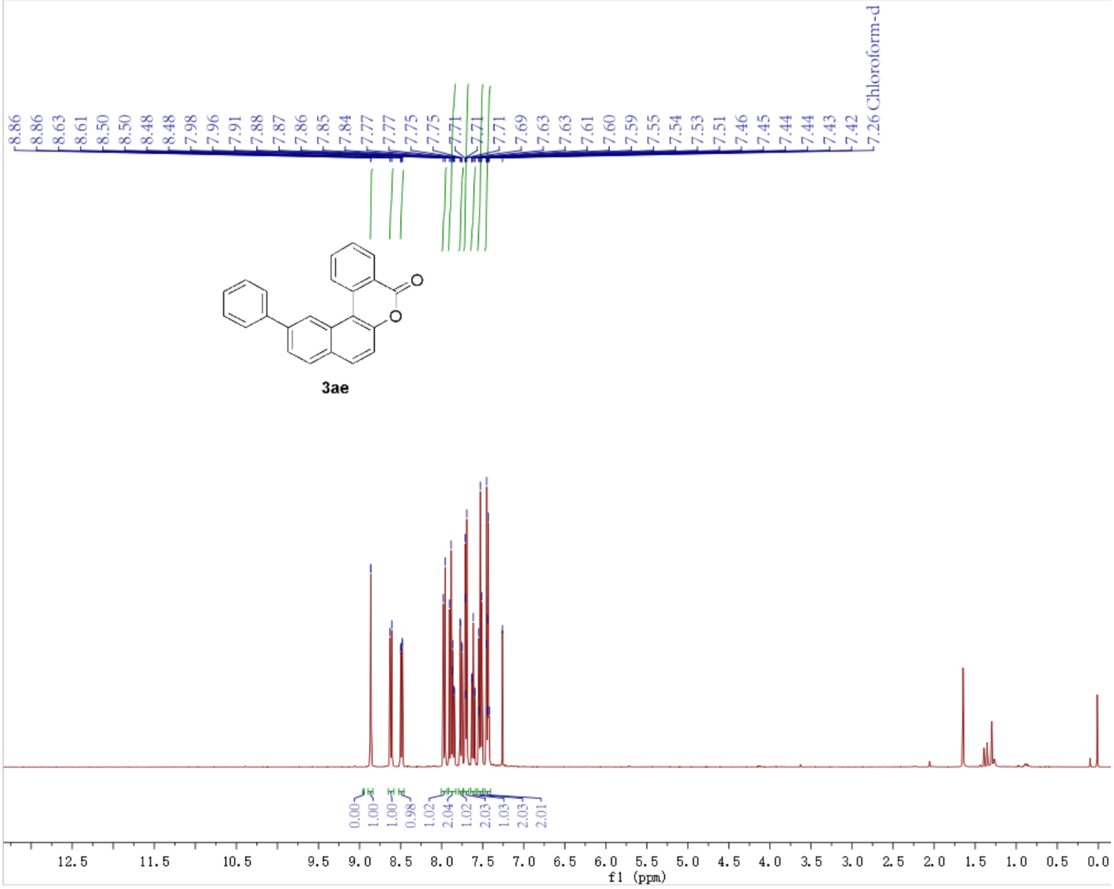

8.86
8.86
8.63
8.61
8.50
8.50
8.48
8.48
7.98
7.96
7.91
7.88
7.87
7.86
7.85
7.84
7.77
7.77
7.75
7.75
7.71
7.71
7.71
7.71
7.69
7.63
7.63
7.61
7.60
7.59
7.55
7.54
7.53
7.51
7.46
7.45
7.44
7.44
7.43
7.42
7.26 Chloroform-d
3ae
0.00
1.00
1.00
0.98
1.02
2.04
1.02
2.03
1.03
2.03
2.01
12.5
11.5
10.5
9.5
9.0
8.5
8.0
7.5
7.0
6.5
6.0
5.5
5.0
4.5
4.0
3.5
3.0
2.5
2.0
1.5
1.0
0.5
0.0
f1 (ppm)

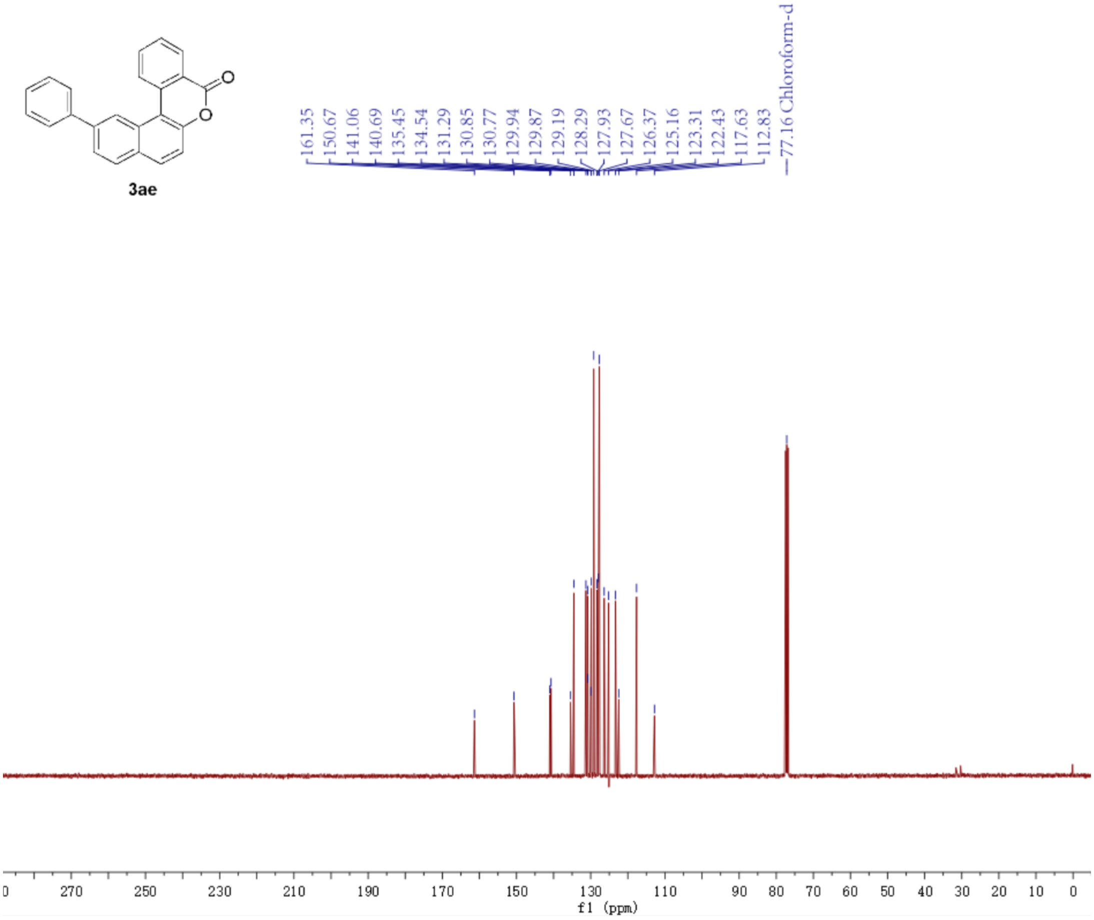

3ae
161.35
150.67
141.06
140.69
135.45
134.54
131.29
130.85
130.77
129.94
129.87
129.19
128.29
127.93
127.67
126.37
125.16
123.31
122.43
117.63
112.83
77.16 Chloroform-d
270
250
230
210
190
170
150
130
110
90
80
70
60
50
40
30
20
10
0
f1 (ppm)

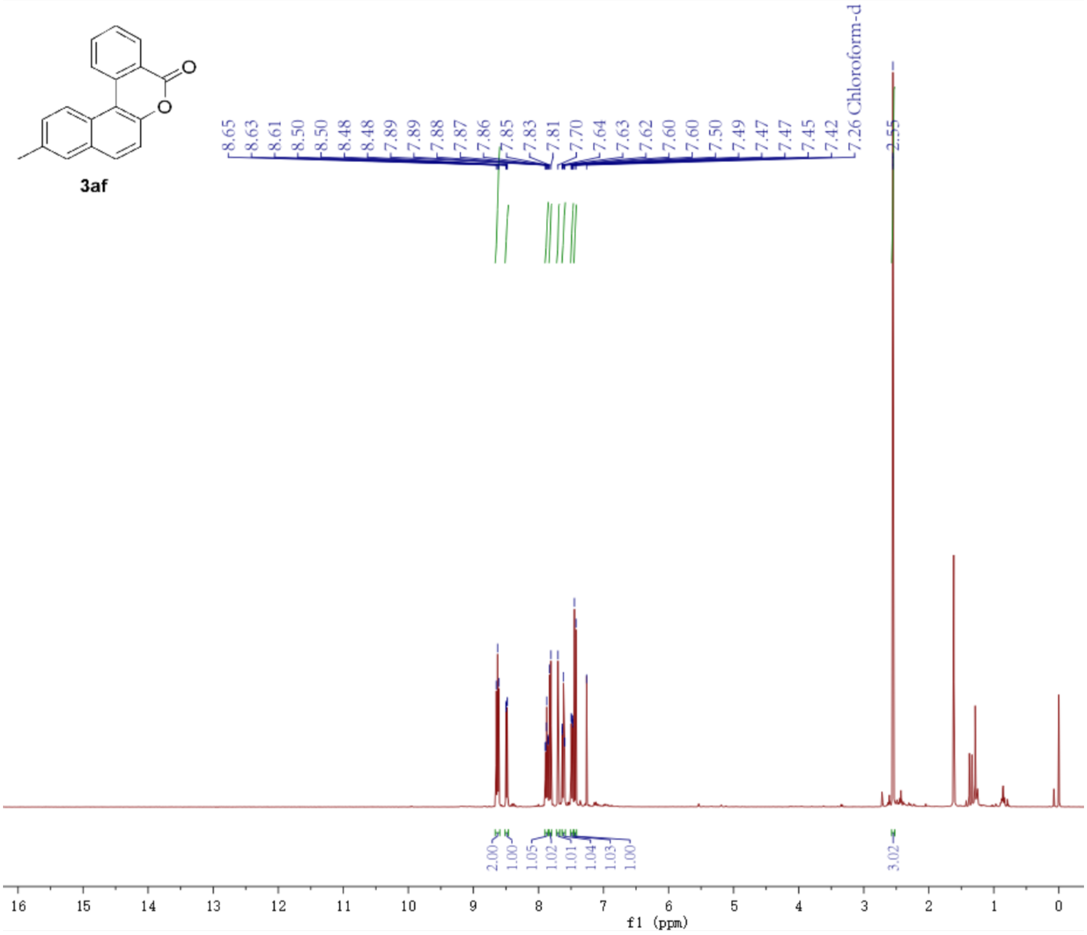
3af
8.65
8.63
8.61
8.50
8.50
8.48
8.48
7.89
7.88
7.87
7.86
7.85
7.83
7.81
7.70
7.64
7.63
7.62
7.60
7.60
7.50
7.49
7.47
7.47
7.45
7.42
7.26 Chloroform-d
2.55
2.00
1.00
1.05
1.02
1.01
1.04
1.03
1.00
3.02
f1 (ppm)

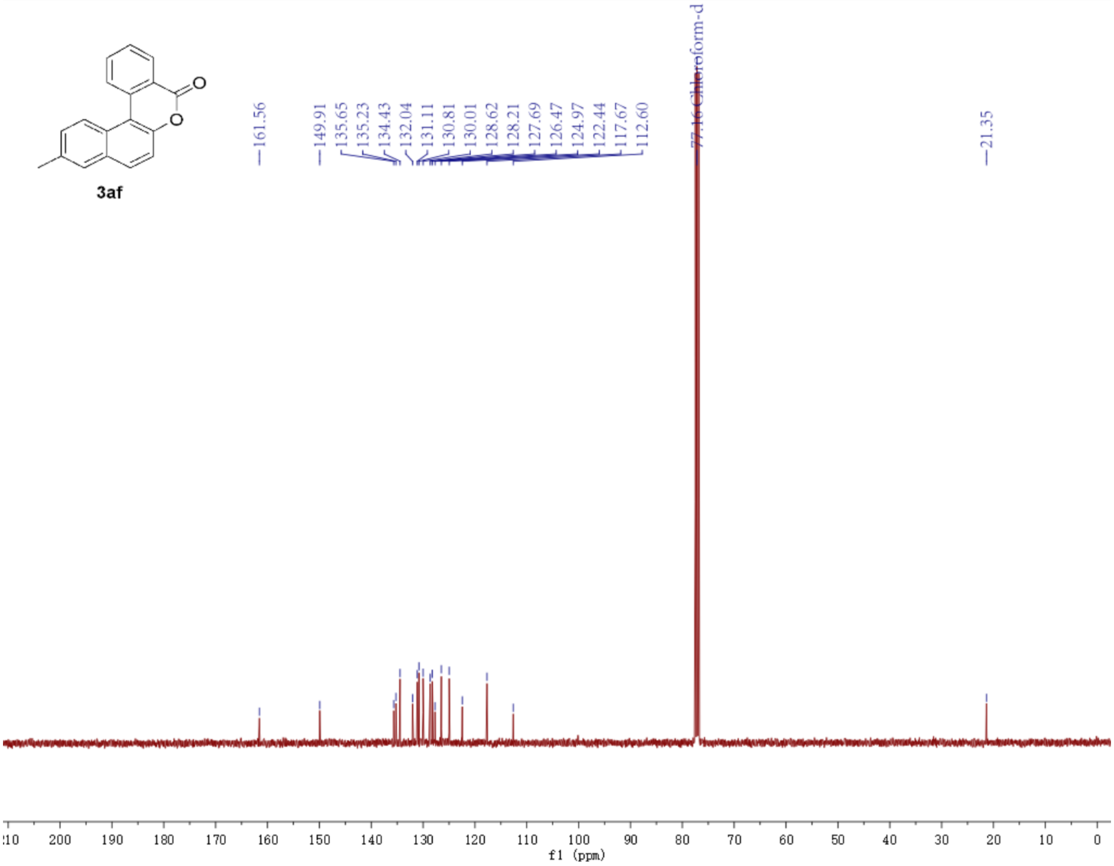
3af
161.56
149.91
135.65
135.23
134.43
132.04
131.11
130.81
130.01
128.62
128.21
127.69
126.47
124.97
122.44
117.67
112.60
77.16 Chloroform-d
21.35
f1 (ppm)

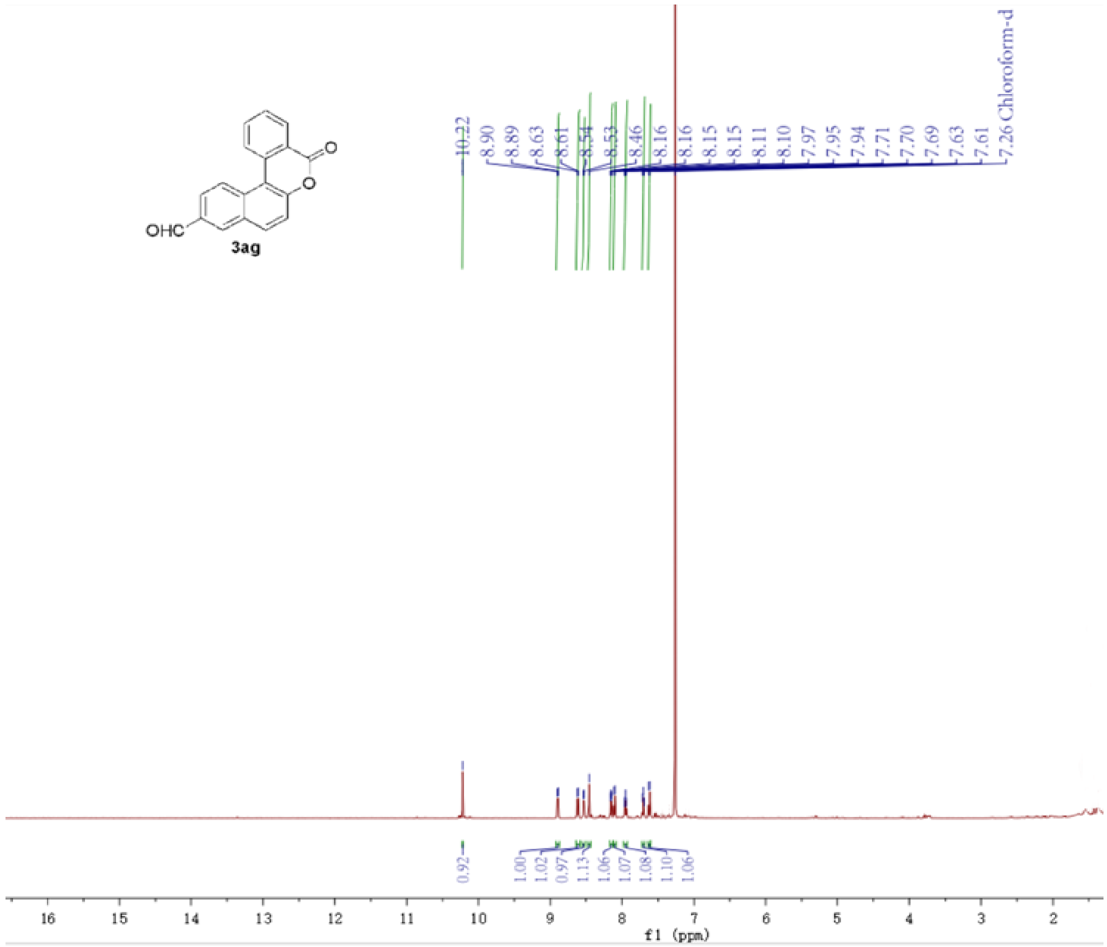

OHC
3ag
10.22
8.90
8.89
8.63
8.61
8.54
8.53
8.46
8.16
8.16
8.15
8.15
8.11
8.10
7.97
7.95
7.94
7.71
7.70
7.69
7.63
7.61
7.26 Chloroform-d
0.92
1.00
1.02
0.97
1.13
1.06
1.07
1.08
1.10
1.06
f1 (ppm)

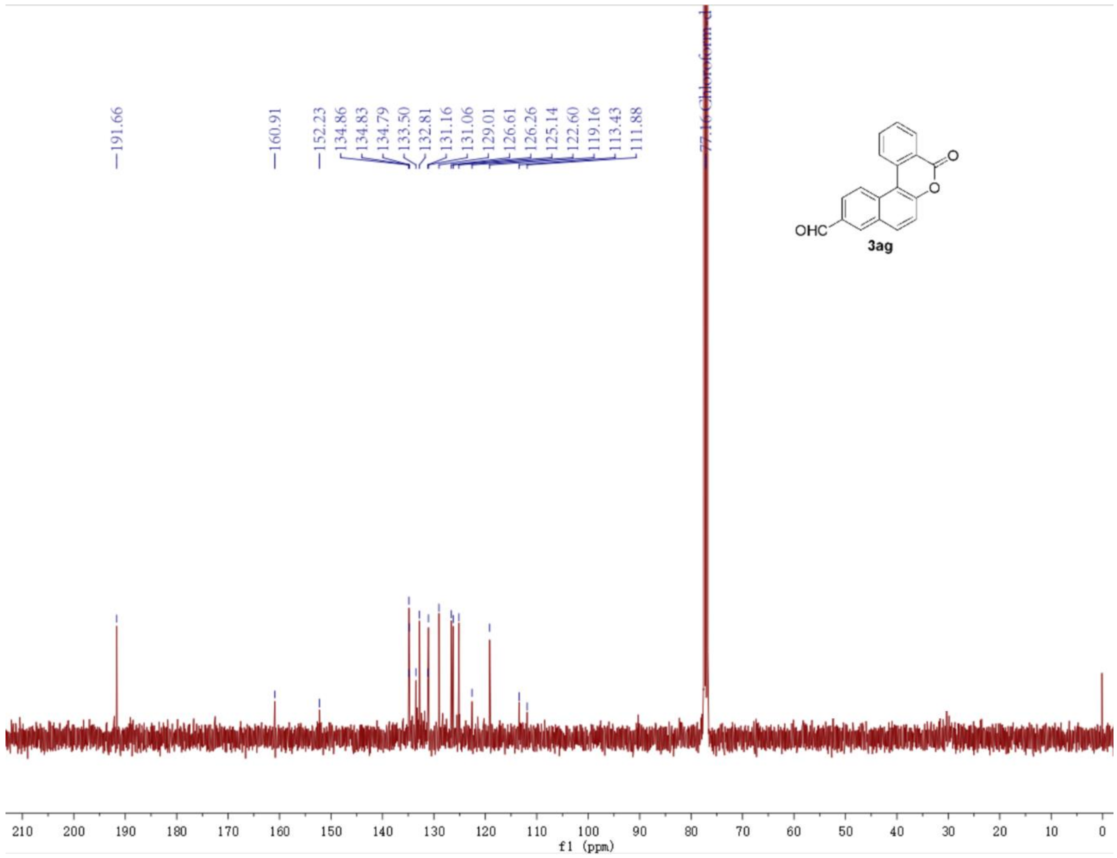

191.66
160.91
152.23
134.86
134.83
134.79
133.50
132.81
131.16
131.06
129.01
126.61
126.26
125.14
122.60
119.16
113.43
111.88
77.16 Chloroform-d
OHC
3ag
f1 (ppm)

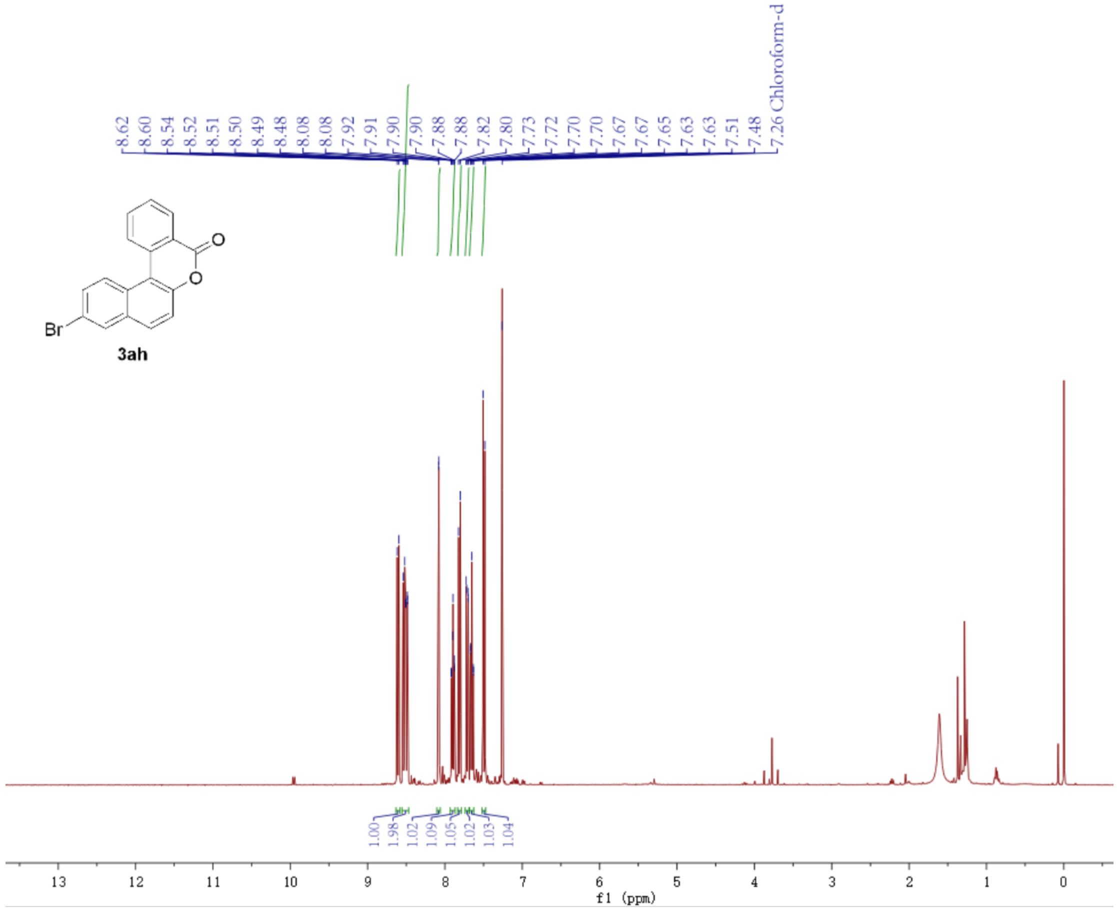
8.62
8.60
8.54
8.52
8.51
8.50
8.49
8.48
8.08
8.08
7.92
7.91
7.90
7.90
7.88
7.88
7.82
7.80
7.73
7.72
7.70
7.70
7.67
7.67
7.65
7.63
7.63
7.51
7.48
7.26 Chloroform-d
Br
O
O
3ah
1.00
1.98
1.02
1.09
1.05
1.02
1.03
1.04
f1 (ppm)

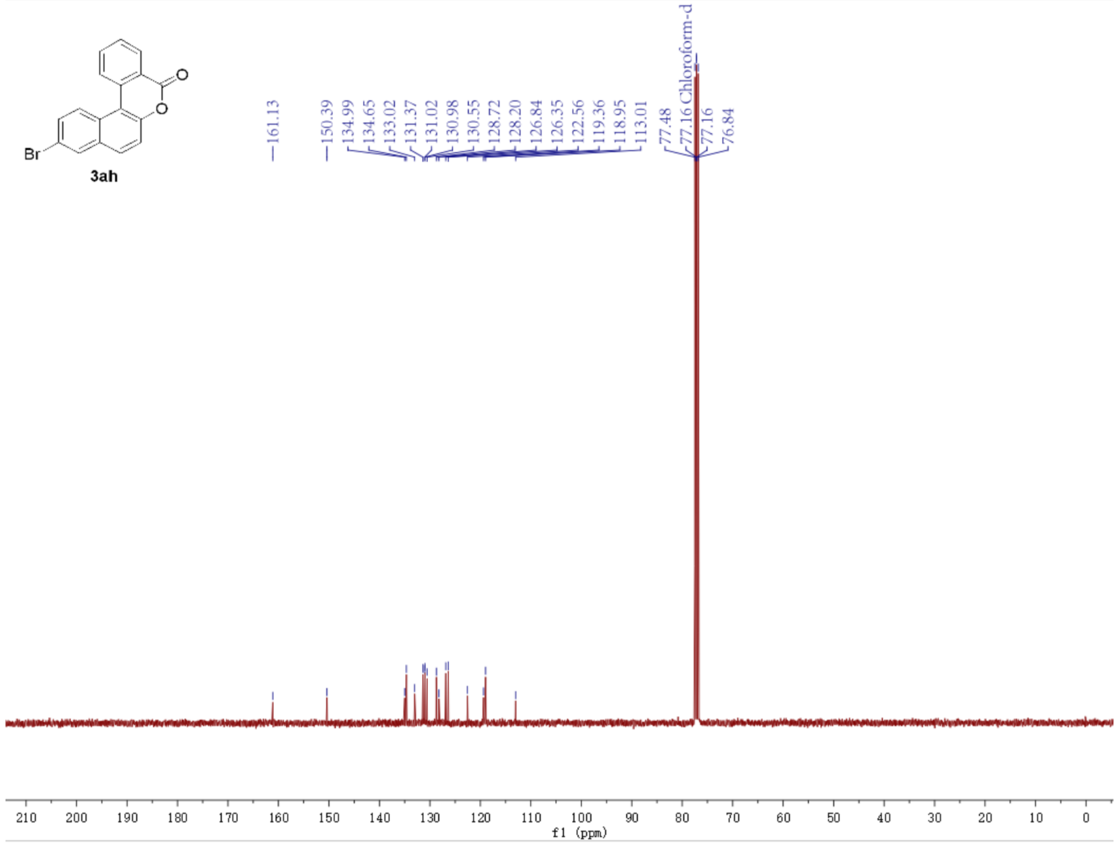
Br
O
O
3ah
161.13
150.39
134.99
134.65
133.02
131.37
131.02
130.98
130.55
128.72
128.20
126.84
126.35
122.56
119.36
118.95
113.01
77.48
77.16 Chloroform-d
77.16
76.84
f1 (ppm)

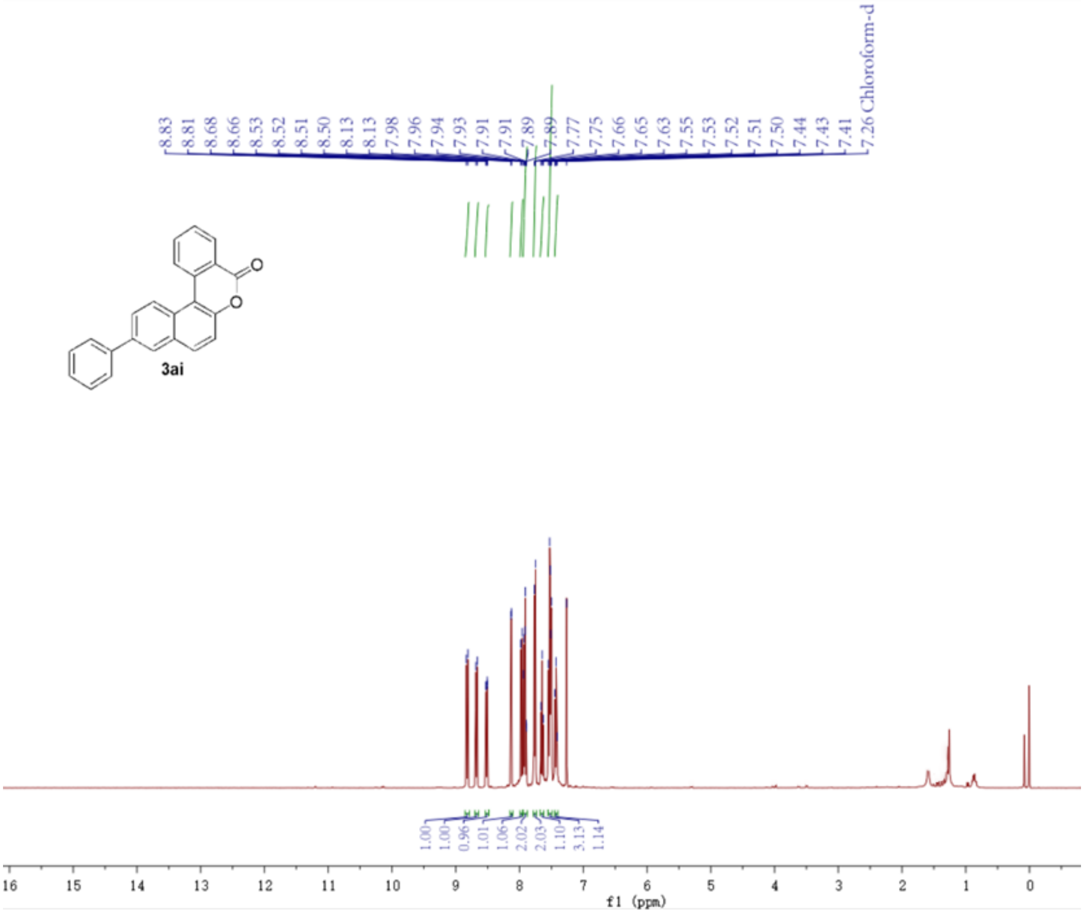
8.83
8.81
8.68
8.66
8.53
8.52
8.51
8.50
8.13
8.13
7.98
7.96
7.94
7.93
7.91
7.91
7.89
7.89
7.77
7.75
7.66
7.65
7.63
7.55
7.53
7.52
7.51
7.50
7.44
7.43
7.41
7.26 Chloroform-d
3ai
1.00
1.00
0.96
1.01
1.06
2.02
2.03
1.10
3.13
1.14
f1 (ppm)

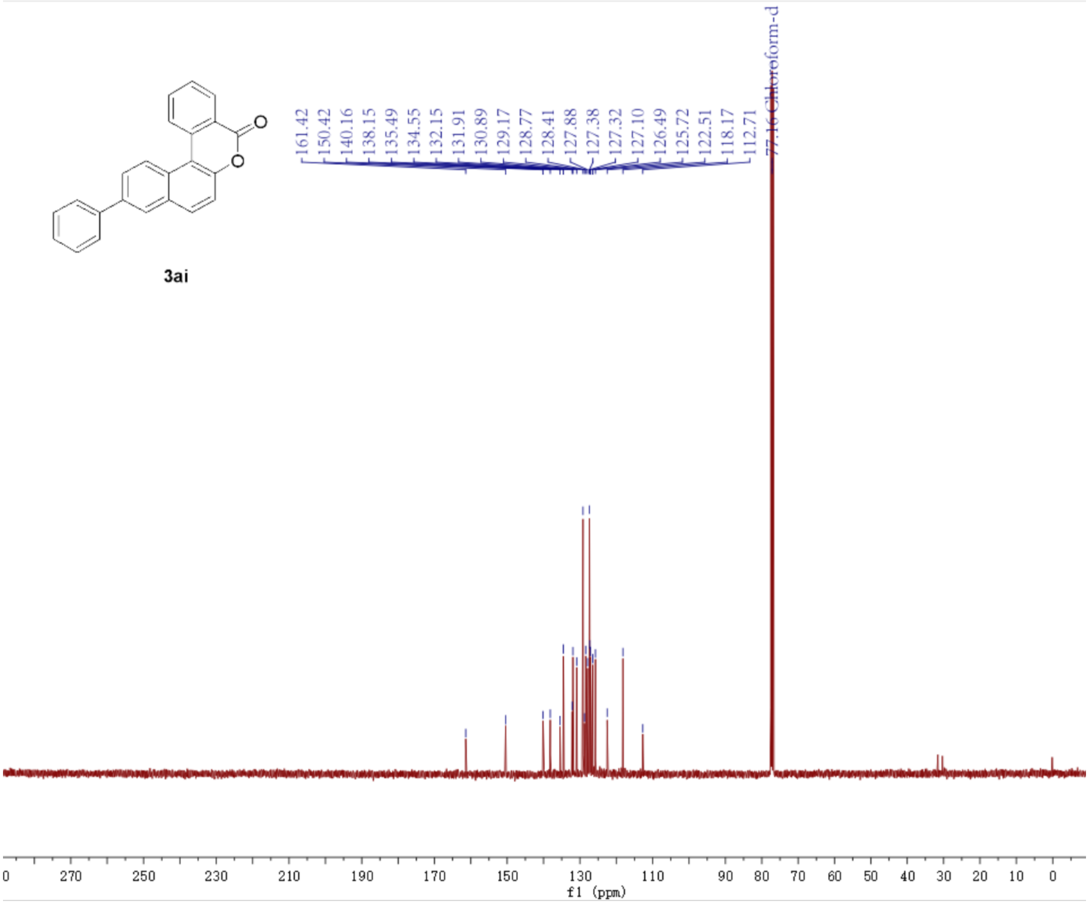
161.42
150.42
140.16
138.15
135.49
134.55
132.15
131.91
130.89
129.17
128.77
128.41
127.88
127.38
127.32
127.10
126.49
125.72
122.51
118.17
112.71
77.16 Chloroform-d
3ai
f1 (ppm)

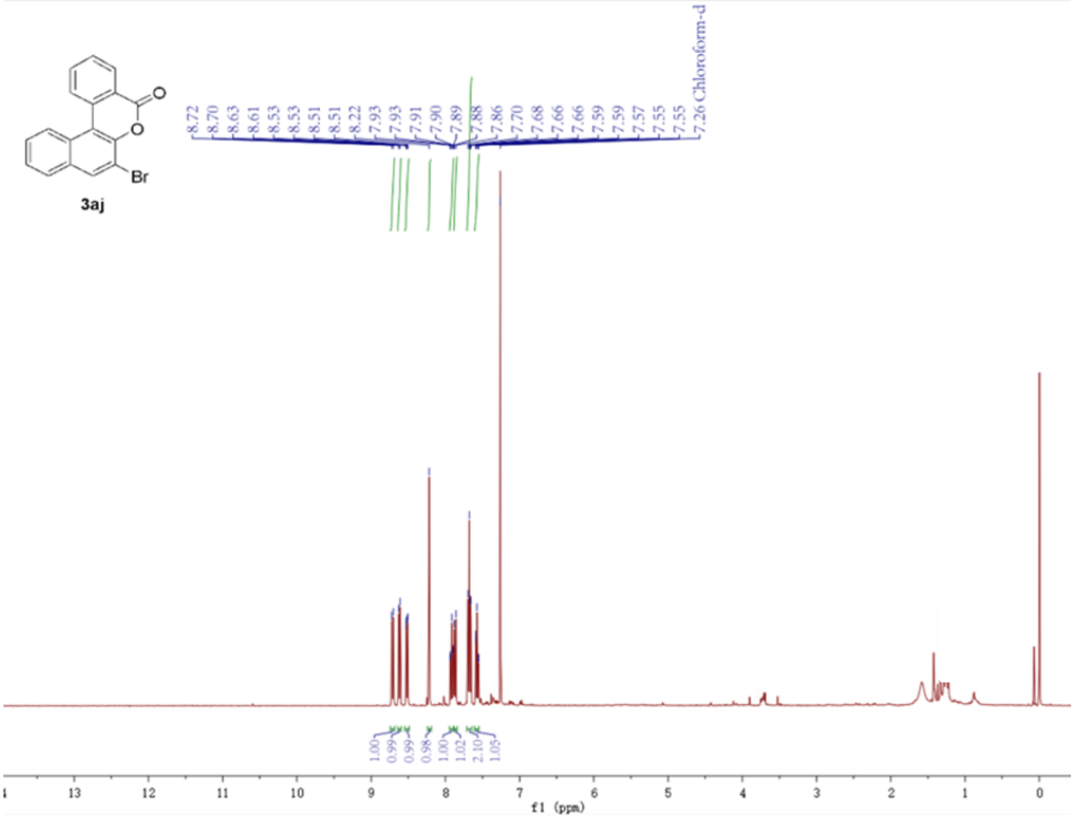

Br
3aj
8.72
8.70
8.63
8.61
8.53
8.53
8.51
8.51
8.22
7.93
7.93
7.91
7.90
7.89
7.88
7.86
7.70
7.68
7.66
7.66
7.59
7.59
7.57
7.55
7.55
7.26 Chloroform-d
1.00
0.99
0.99
0.98
1.00
1.02
2.10
1.05
f1 (ppm)

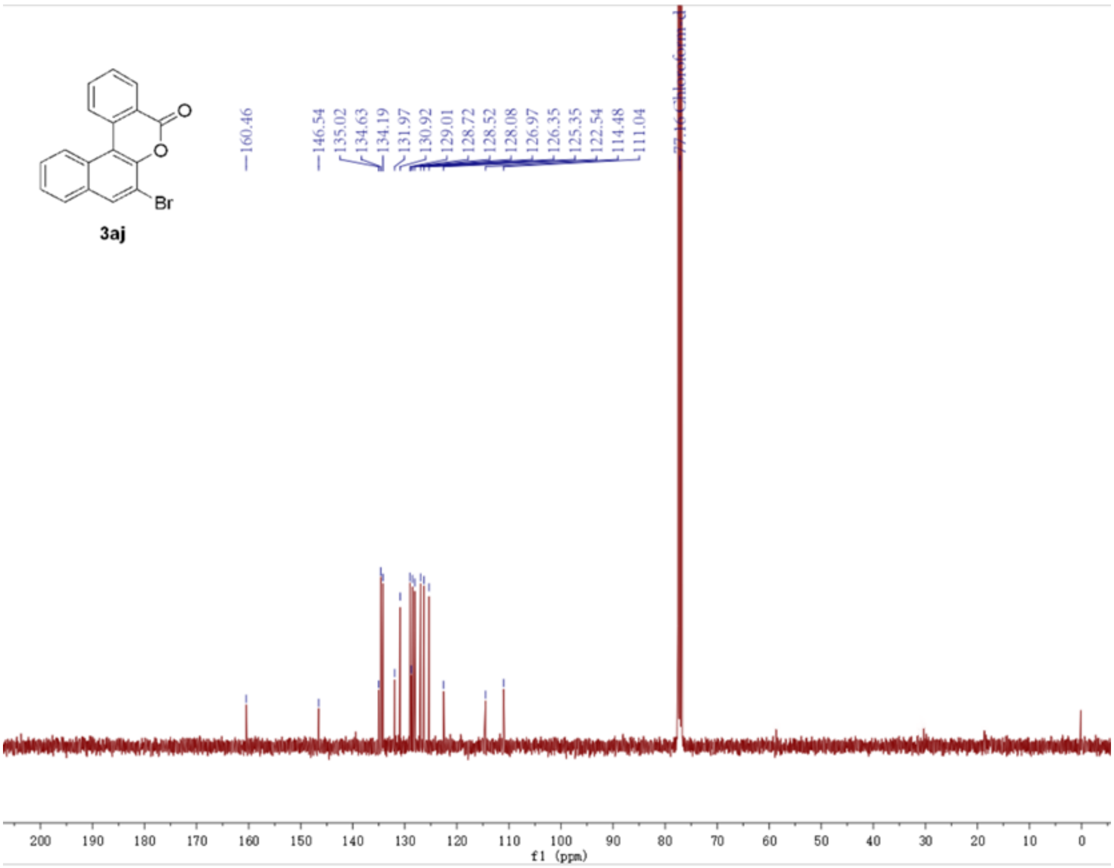

Br
3aj
160.46
146.54
135.02
134.63
134.19
131.97
130.92
129.01
128.72
128.52
128.08
126.97
126.35
125.35
122.54
114.48
111.04
77.16 Chloroform-d
f1 (ppm)

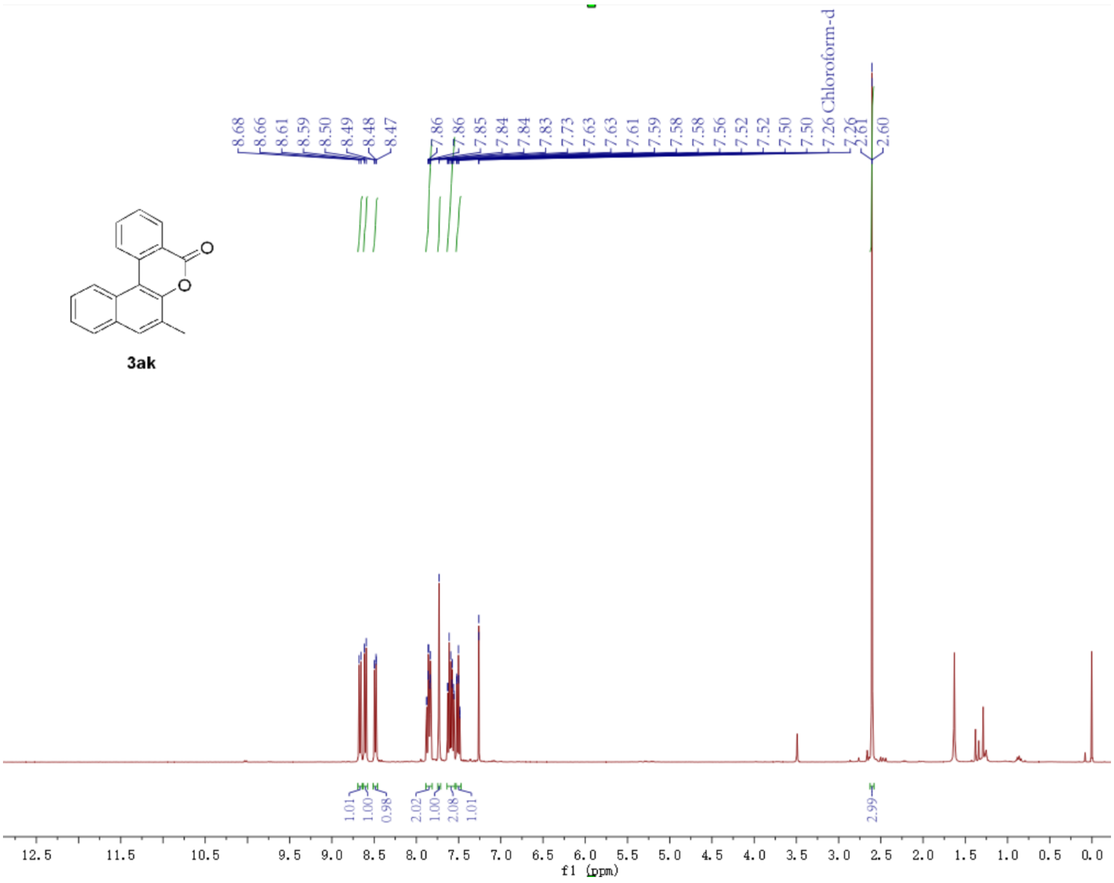
8.68
8.66
8.61
8.59
8.50
8.49
8.48
8.47
7.86
7.86
7.85
7.84
7.84
7.83
7.73
7.63
7.63
7.61
7.59
7.58
7.58
7.56
7.52
7.52
7.50
7.50
7.26 Chloroform-d
7.26
2.61
2.60
3ak
1.01
1.00
0.98
2.02
1.00
2.08
1.01
2.99
12.5 11.5 10.5 9.5 9.0 8.5 8.0 7.5 7.0 6.5 6.0 5.5 5.0 4.5 4.0 3.5 3.0 2.5 2.0 1.5 1.0 0.5 0.0
f1 (ppm)

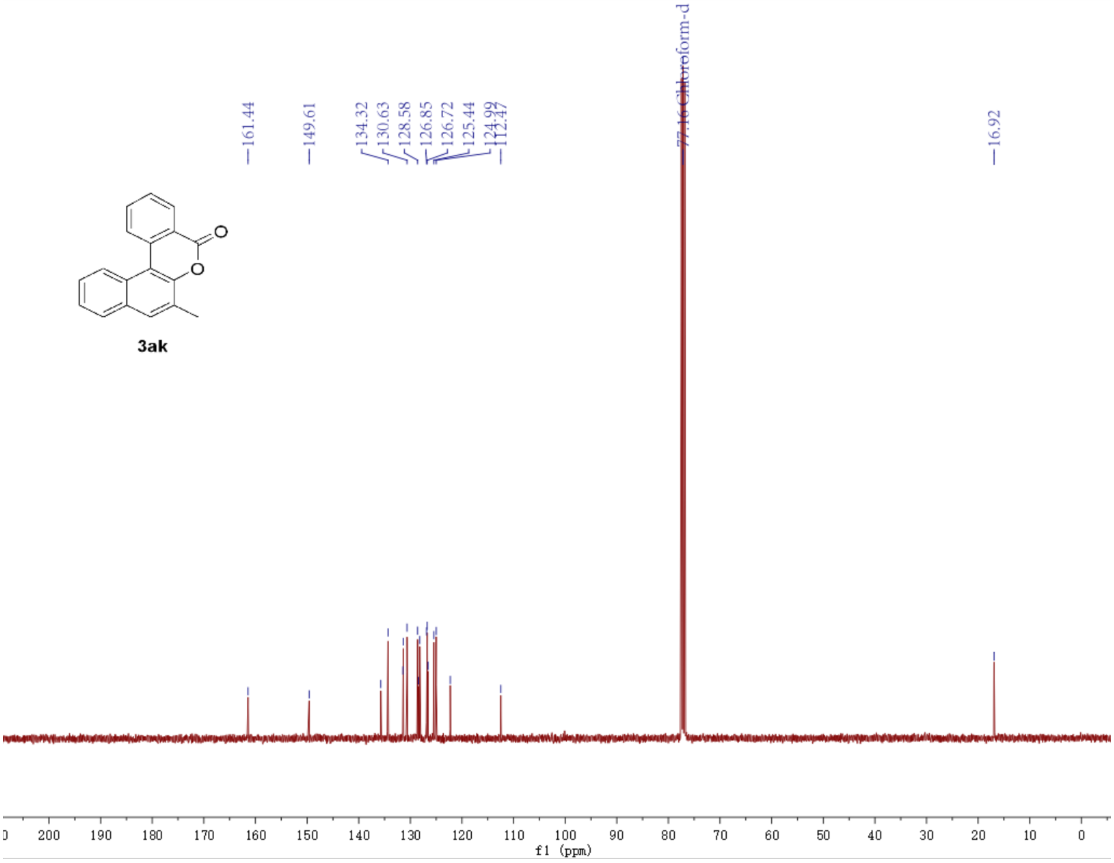
161.44
149.61
134.32
130.63
128.58
126.85
126.72
125.44
124.99
112.47
77.16 Chloroform-d
16.92
3ak
200 190 180 170 160 150 140 130 120 110 100 90 80 70 60 50 40 30 20 10 0
f1 (ppm)

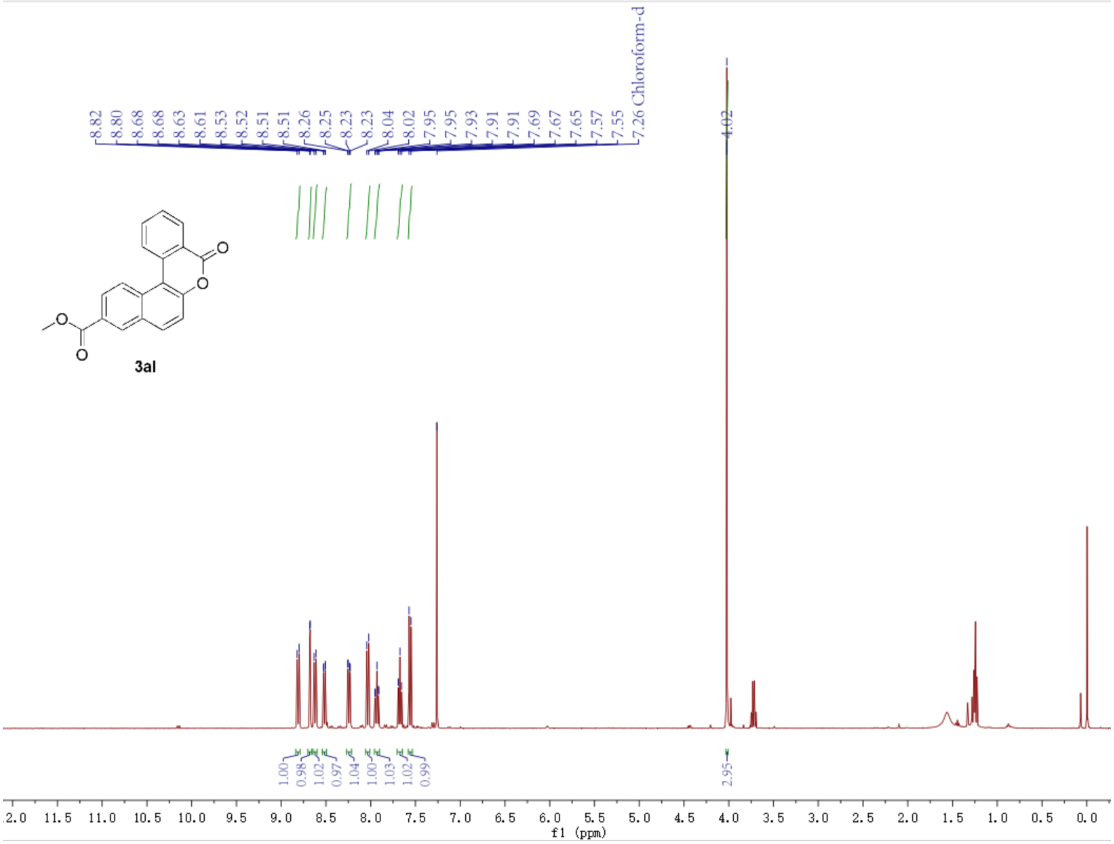

8.82 8.80 8.68 8.68 8.63 8.61 8.53 8.52 8.51 8.51 8.26 8.25 8.23 8.23 8.04 8.02 7.95 7.95 7.93 7.91 7.91 7.69 7.67 7.65 7.57 7.55 7.26 Chloroform-d
4.02
3al
1.00 0.98 1.02 0.97 1.04 1.00 1.03 1.02 0.99
2.95
f1 (ppm)

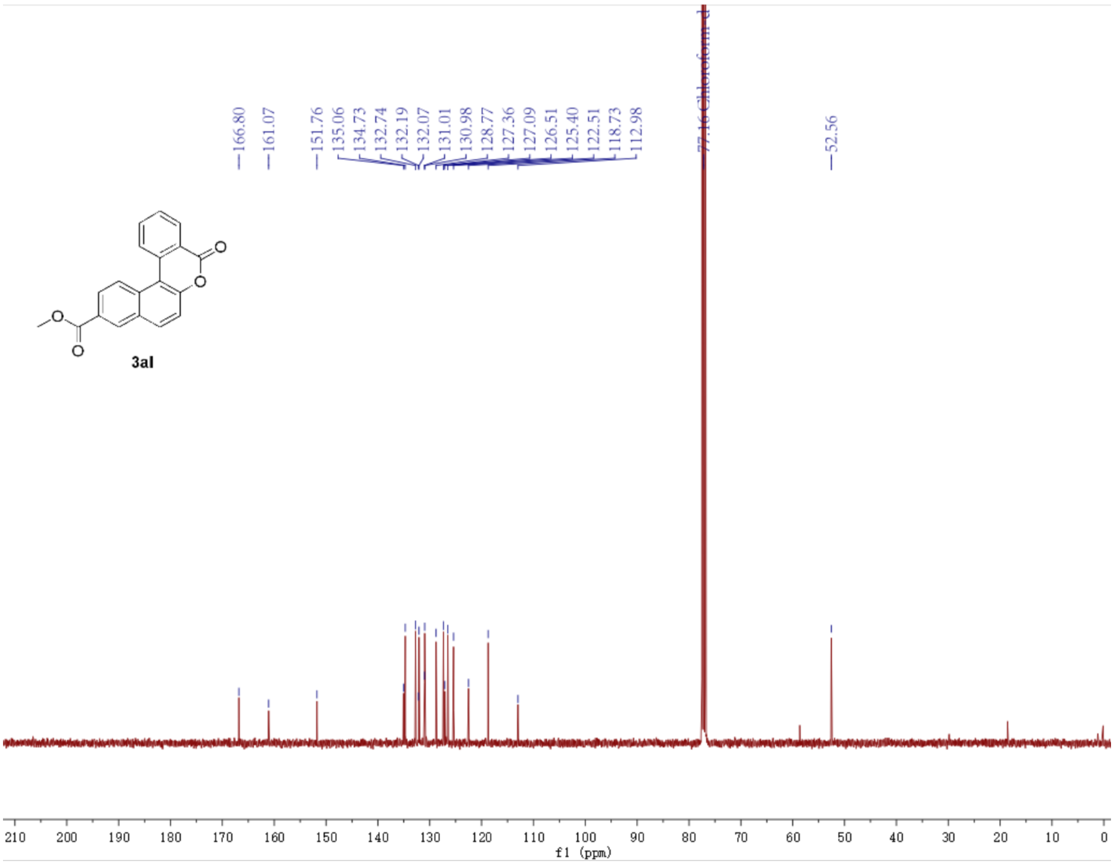

166.80
161.07
151.76 135.06 134.73 132.74 132.19 132.07 131.01 130.98 128.77 127.36 127.09 126.51 125.40 122.51 118.73 112.98
77.16 Chloroform-d
52.56
3al
f1 (ppm)

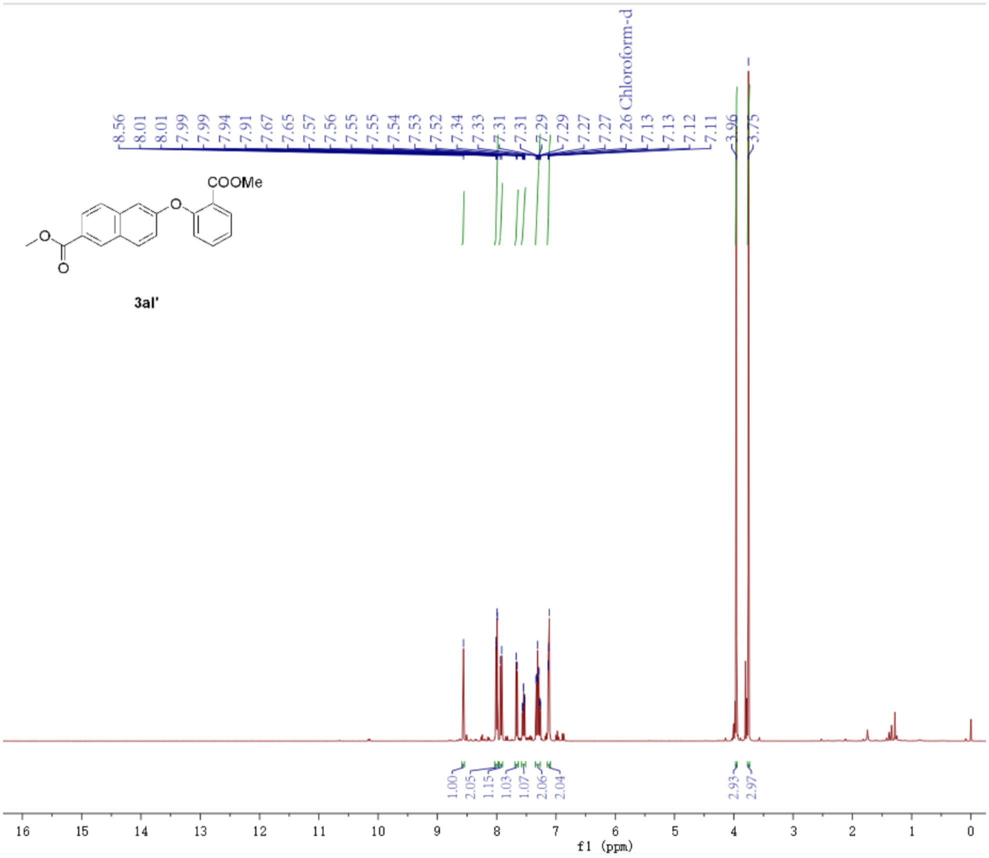
8.56
8.01
8.01
7.99
7.99
7.94
7.91
7.67
7.65
7.57
7.56
7.55
7.55
7.54
7.53
7.52
7.34
7.33
7.31
7.31
7.29
7.29
7.27
7.27
7.26 Chloroform-d
7.13
7.13
7.12
7.11
3.96
3.75
COOMe
3al'
1.00
2.05
1.15
1.03
1.07
2.06
2.04
2.93
2.97
f1 (ppm)

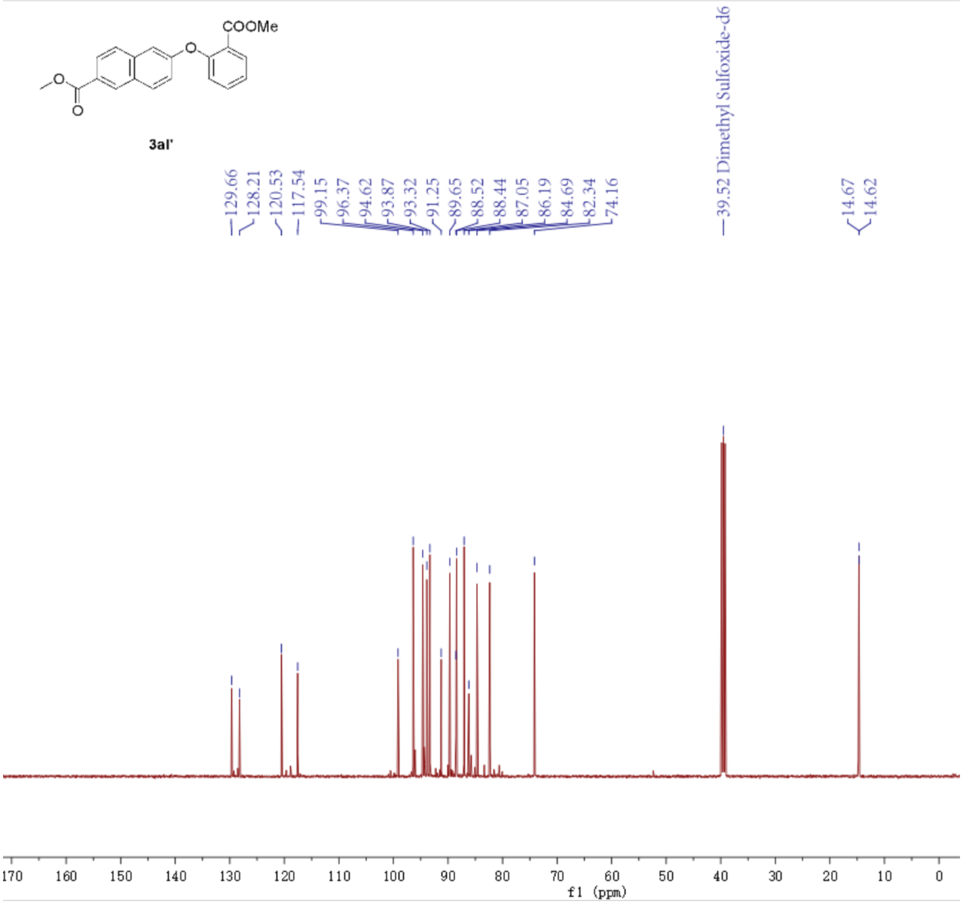
COOMe
3al'
129.66
128.21
120.53
117.54
99.15
96.37
94.62
93.87
93.32
91.25
89.65
88.52
88.44
87.05
86.19
84.69
82.34
74.16
39.52 Dimethyl Sulfoxide-d6
14.67
14.62
f1 (ppm)

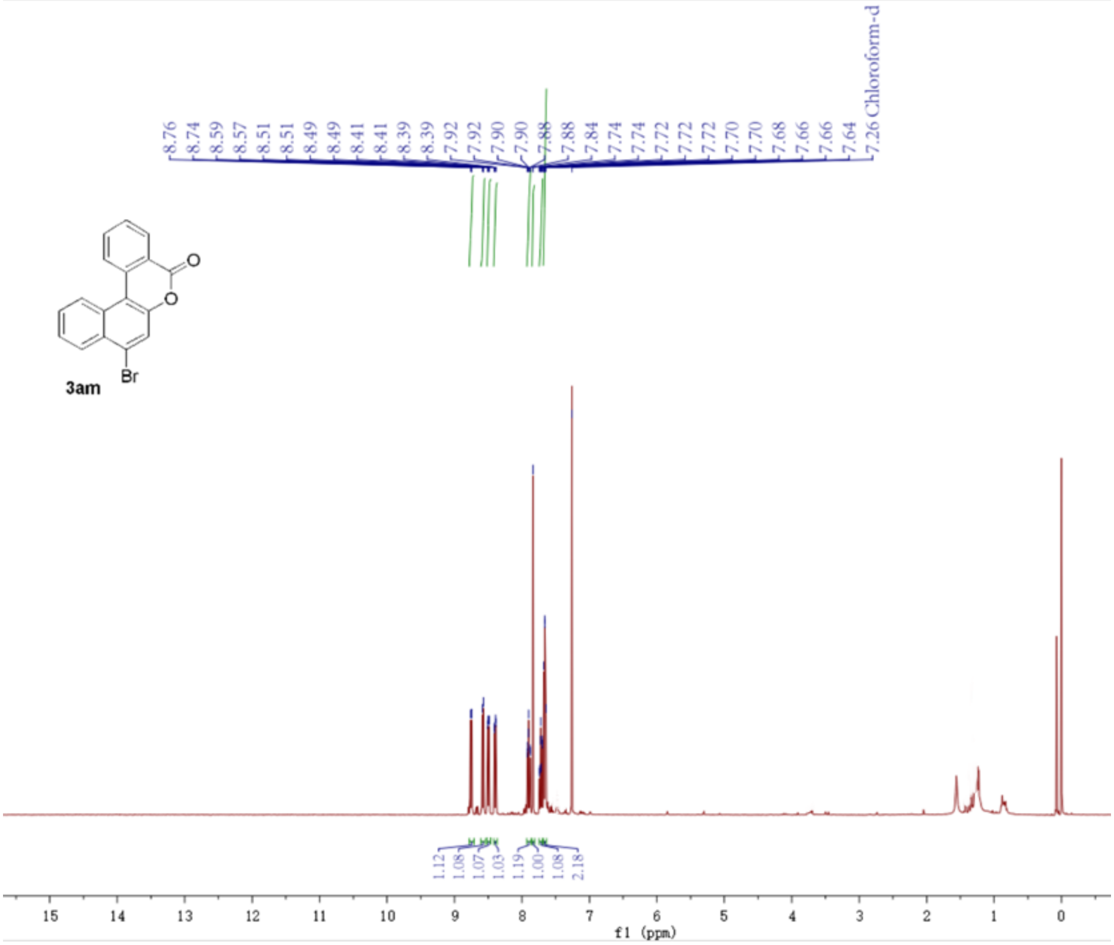
8.76
8.74
8.59
8.57
8.51
8.51
8.49
8.49
8.41
8.41
8.39
8.39
7.92
7.92
7.90
7.90
7.88
7.88
7.84
7.74
7.74
7.72
7.72
7.72
7.72
7.70
7.70
7.68
7.66
7.66
7.64
7.26 Chloroform-d
3am
Br
O
1.12
1.08
1.07
1.03
1.19
1.00
1.08
2.18
15 14 13 12 11 10 9 8 7 6 5 4 3 2 1 0
f1 (ppm)

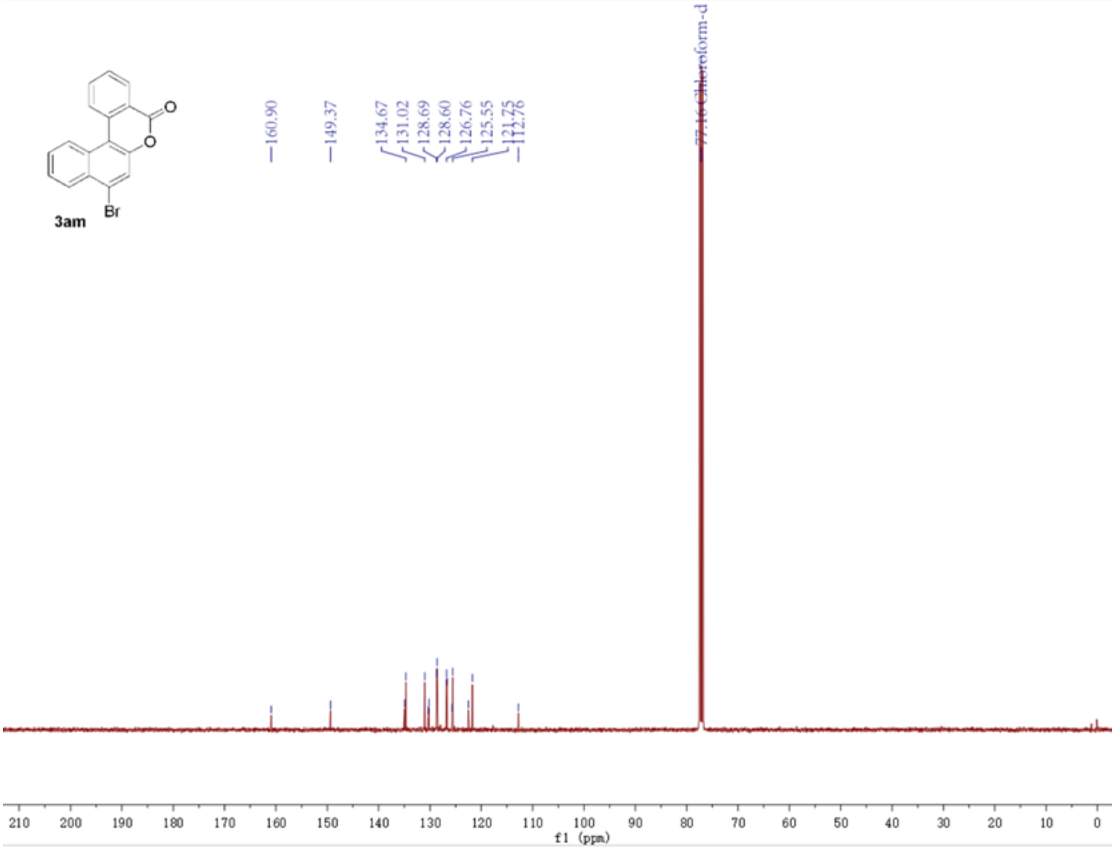
160.90
149.37
134.67
131.02
128.69
128.60
126.76
125.55
121.75
112.76
77.16 Chloroform-d
3am
Br
O
210 200 190 180 170 160 150 140 130 120 110 100 90 80 70 60 50 40 30 20 10 0
f1 (ppm)

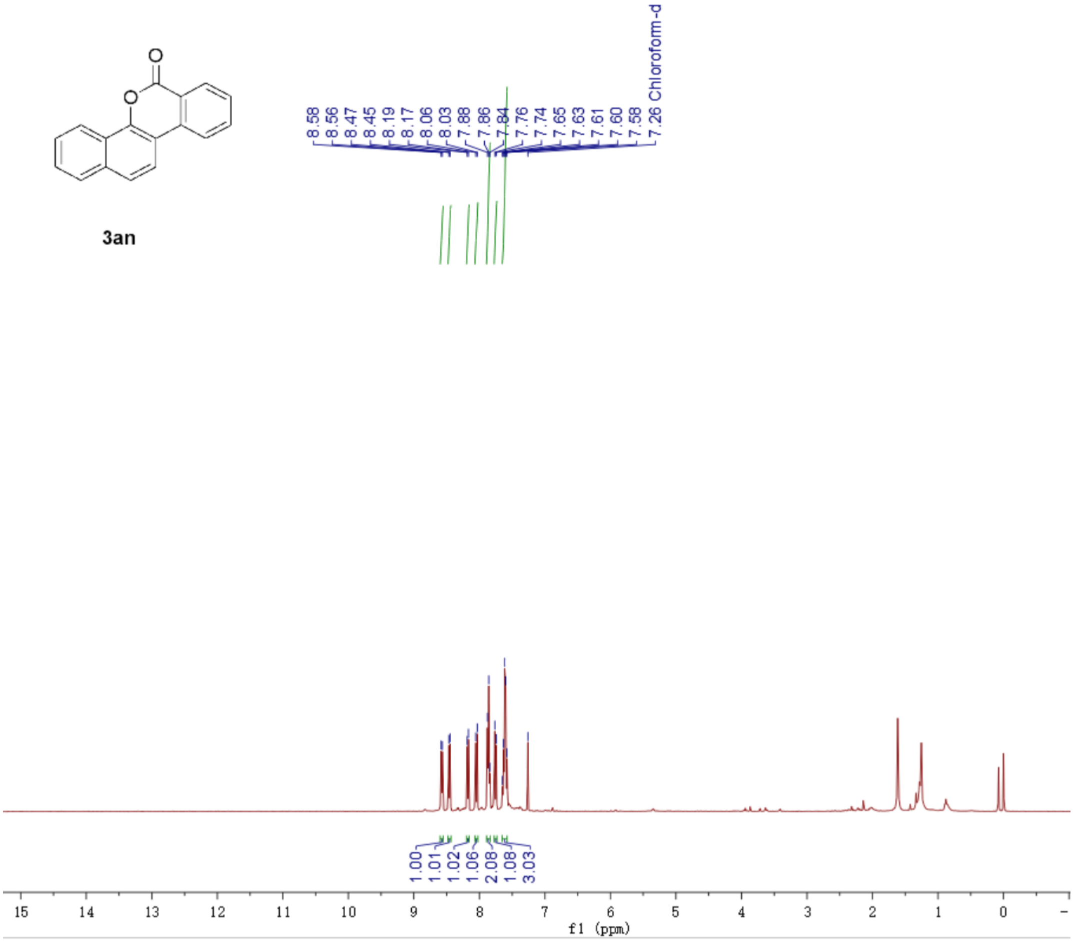
8.58
8.56
8.47
8.45
8.19
8.17
8.06
8.03
7.88
7.86
7.84
7.76
7.74
7.65
7.63
7.61
7.60
7.58
7.26 Chloroform-d
1.00
1.01
1.02
1.06
2.08
1.08
3.03
f1 (ppm)

3an

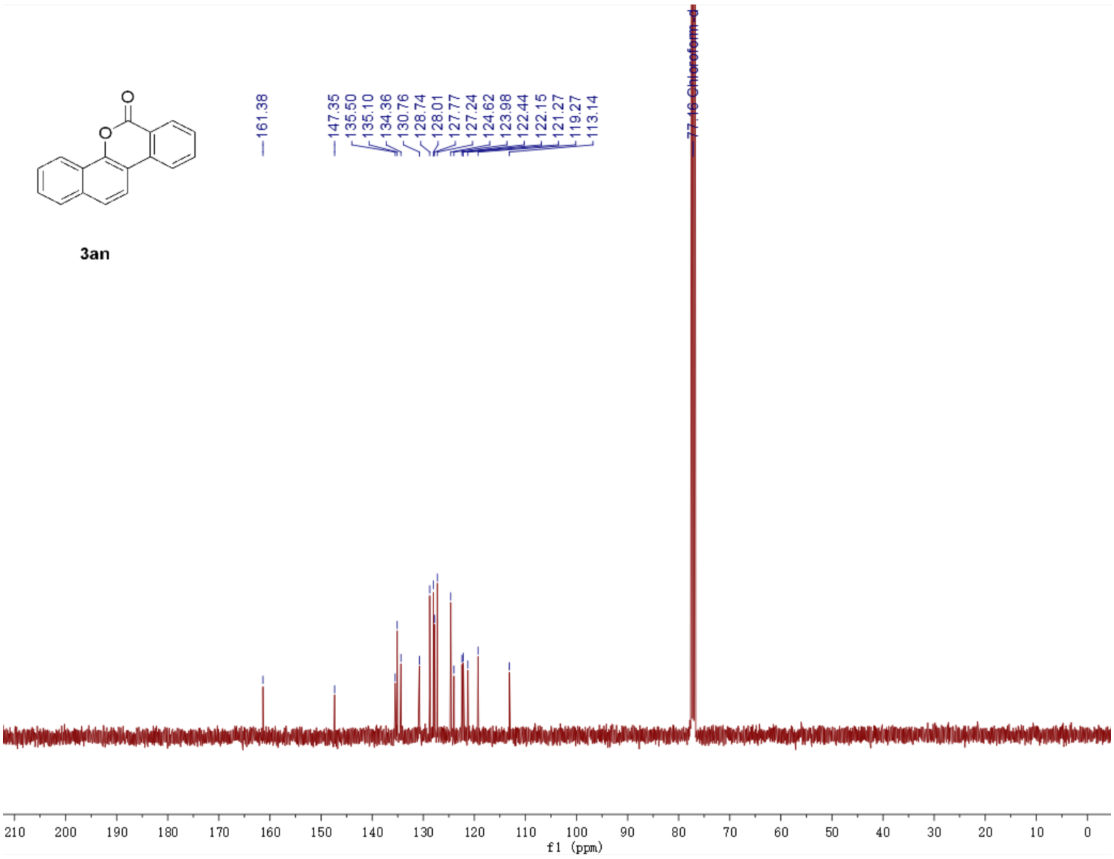
161.38
147.35
135.50
135.10
134.36
130.76
128.74
128.01
127.77
127.24
124.62
123.98
122.44
122.15
121.27
119.27
113.14
77.16 Chloroform-d
f1 (ppm)

3an

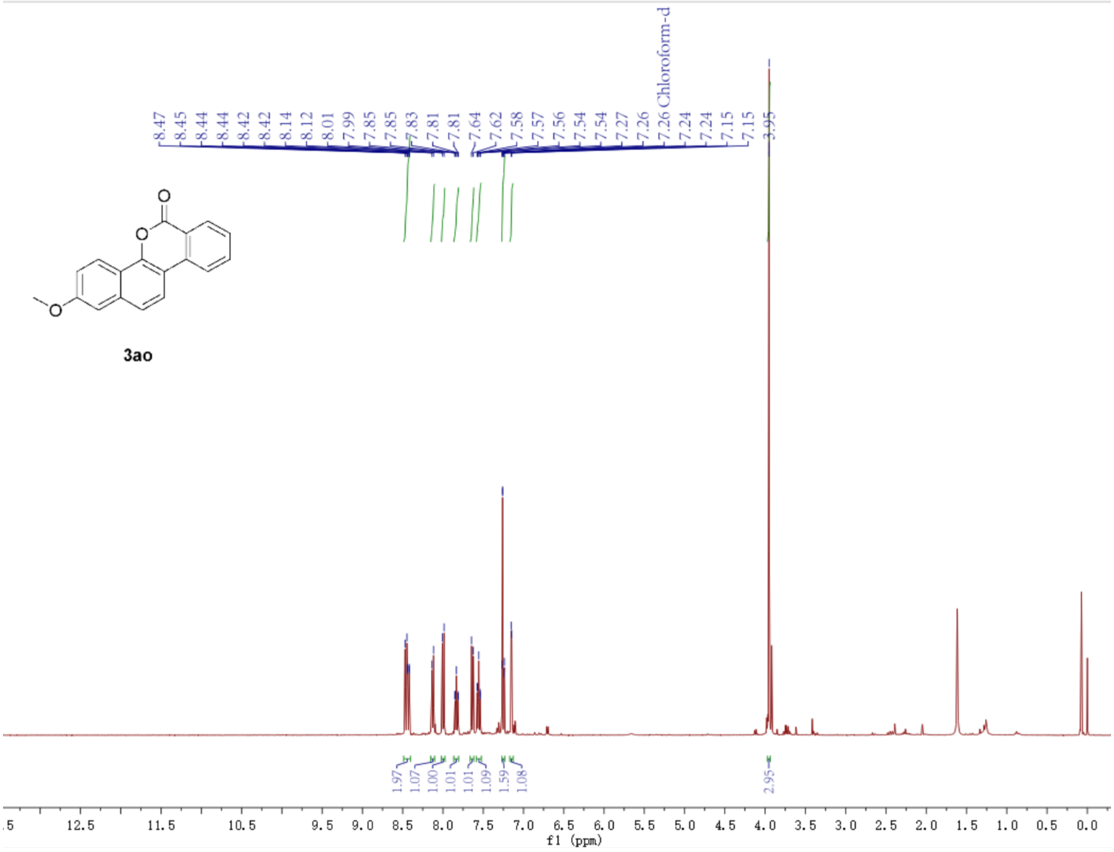
8.47 8.45 8.44 8.44 8.42 8.42 8.14 8.12 8.01 7.99 7.85 7.85 7.83 7.81 7.81 7.64 7.62 7.58 7.57 7.56 7.54 7.54 7.27 7.26 Chloroform-d 7.24 7.24 7.15 7.15
3.95
3ao
1.97 1.07 1.00 1.01 1.01 1.09 1.59 1.08
2.95
f1 (ppm)

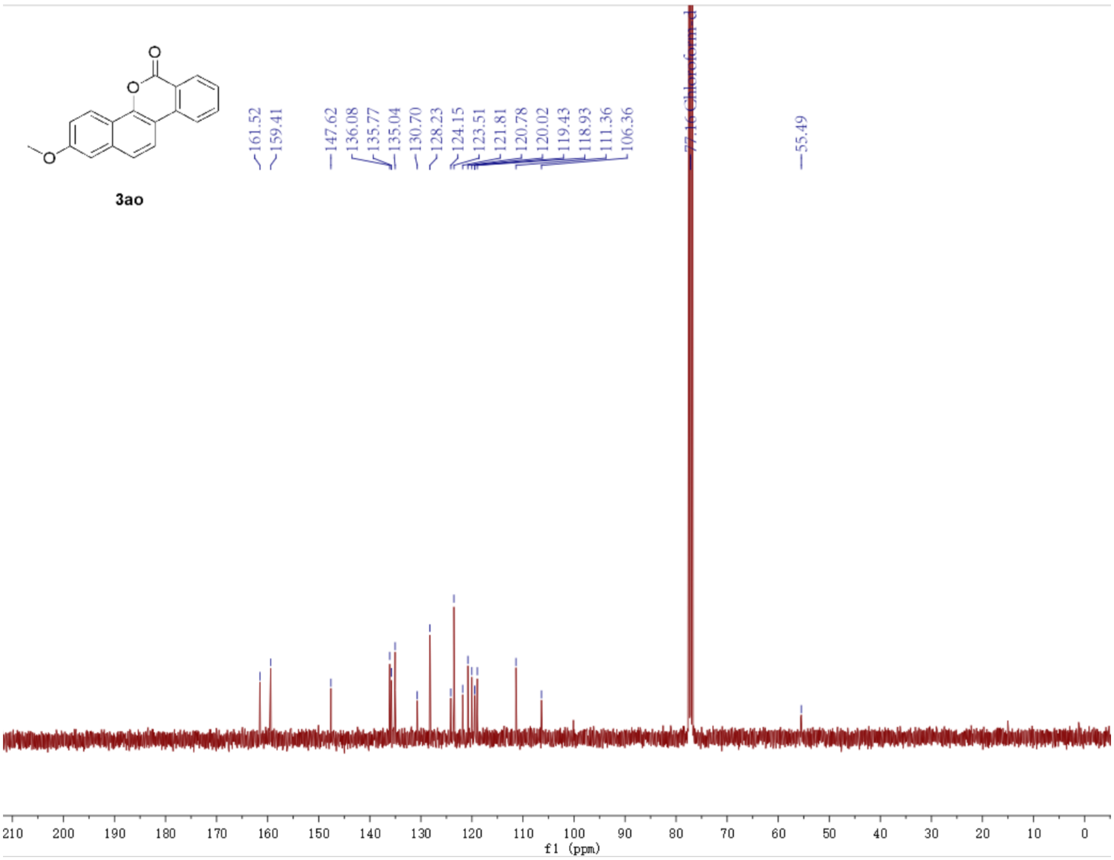
3ao
161.52 159.41
147.62 136.08 135.77 135.04 130.70 128.23 124.15 123.51 121.81 120.78 120.02 119.43 118.93 111.36 106.36
77.16 Chloroform-d
55.49
f1 (ppm)

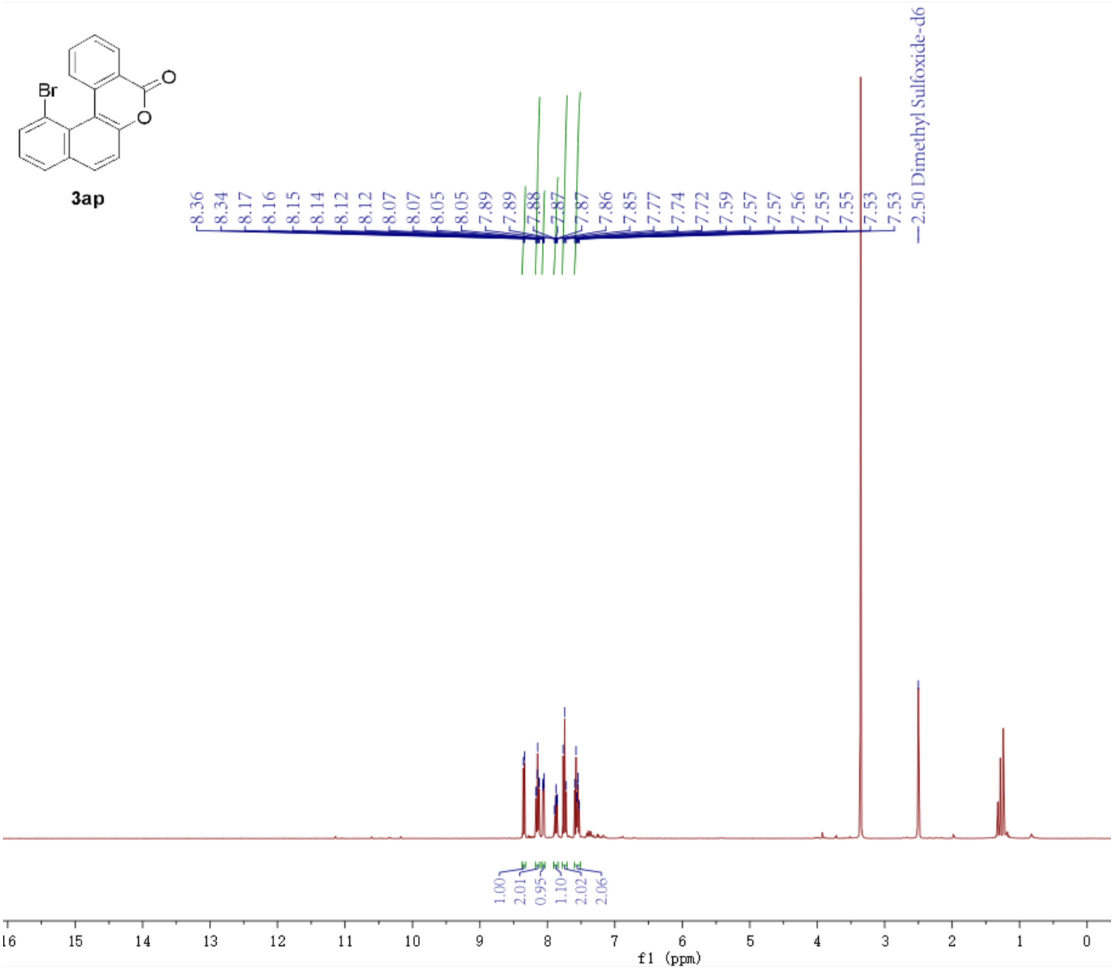
Br
O
O
3ap
8.36
8.34
8.17
8.16
8.15
8.14
8.12
8.12
8.07
8.07
8.05
8.05
7.89
7.89
7.88
7.87
7.87
7.86
7.85
7.77
7.74
7.72
7.59
7.57
7.57
7.56
7.55
7.55
7.53
7.53
2.50 Dimethyl Sulfoxide-d6
1.00
2.01
0.95
1.10
2.02
2.06
16 15 14 13 12 11 10 9 8 7 6 5 4 3 2 1 0
f1 (ppm)

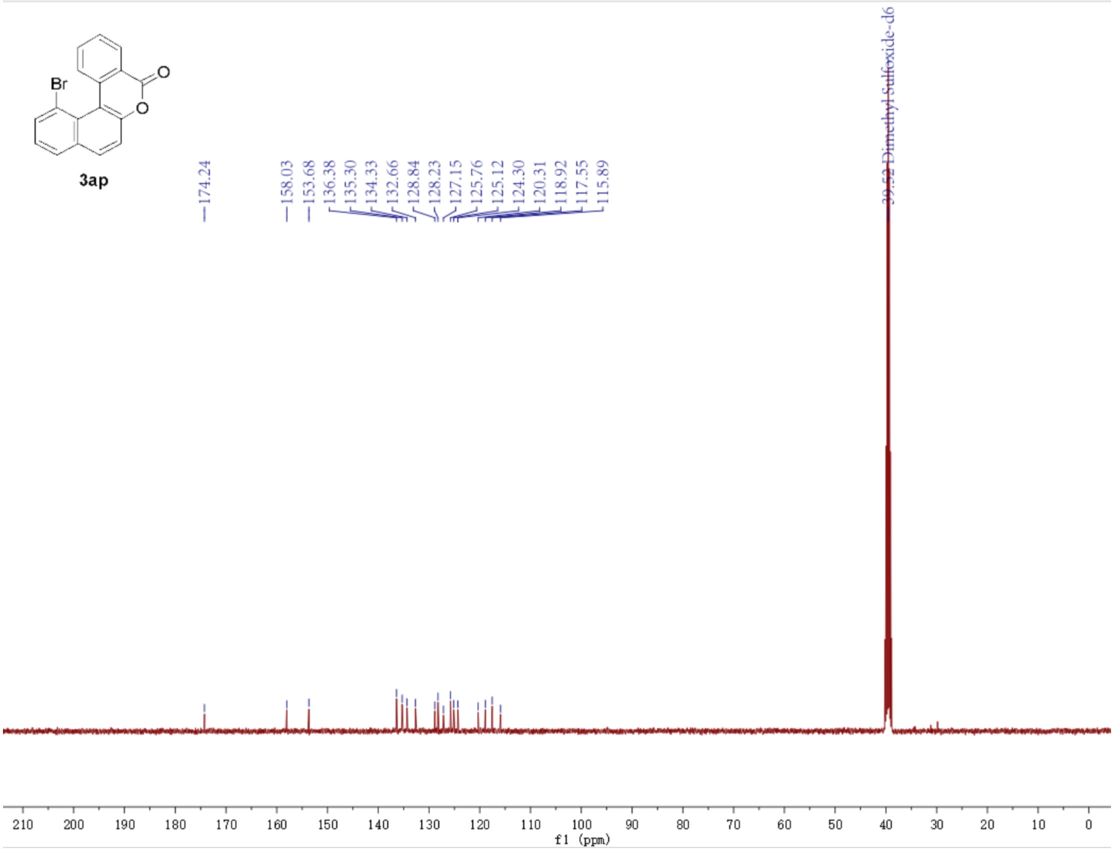
Br
O
O
3ap
174.24
158.03
153.68
136.38
135.30
134.33
132.66
128.84
128.23
127.15
125.76
125.12
124.30
120.31
118.92
117.55
115.89
39.52 Dimethyl Sulfoxide-d6
210 200 190 180 170 160 150 140 130 120 110 100 90 80 70 60 50 40 30 20 10 0
f1 (ppm)

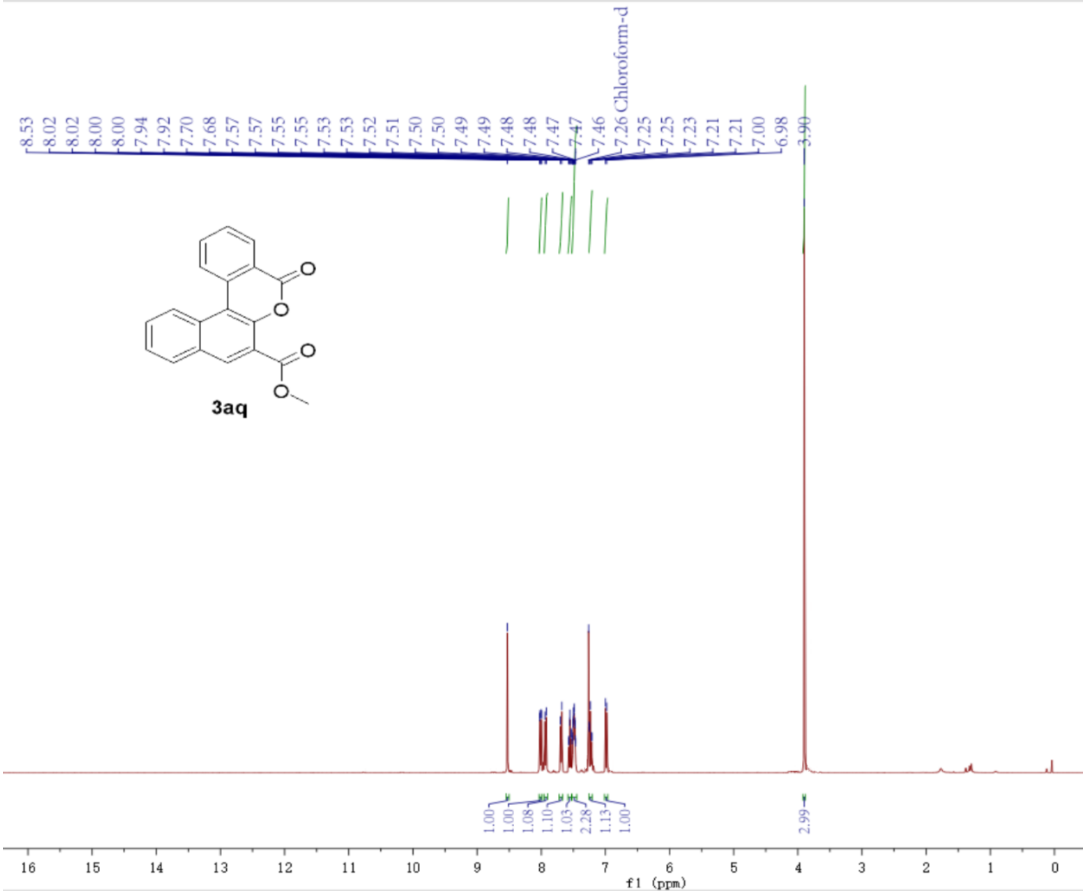
8.53
8.02
8.02
8.00
8.00
7.94
7.92
7.70
7.68
7.57
7.57
7.55
7.55
7.53
7.53
7.52
7.51
7.50
7.50
7.49
7.49
7.48
7.48
7.47
7.47
7.46
7.26 Chloroform-d
7.25
7.25
7.23
7.21
7.21
7.00
6.98
3.90
3aq
1.00
1.00
1.08
1.10
1.03
2.28
1.13
1.00
2.99
f1 (ppm)

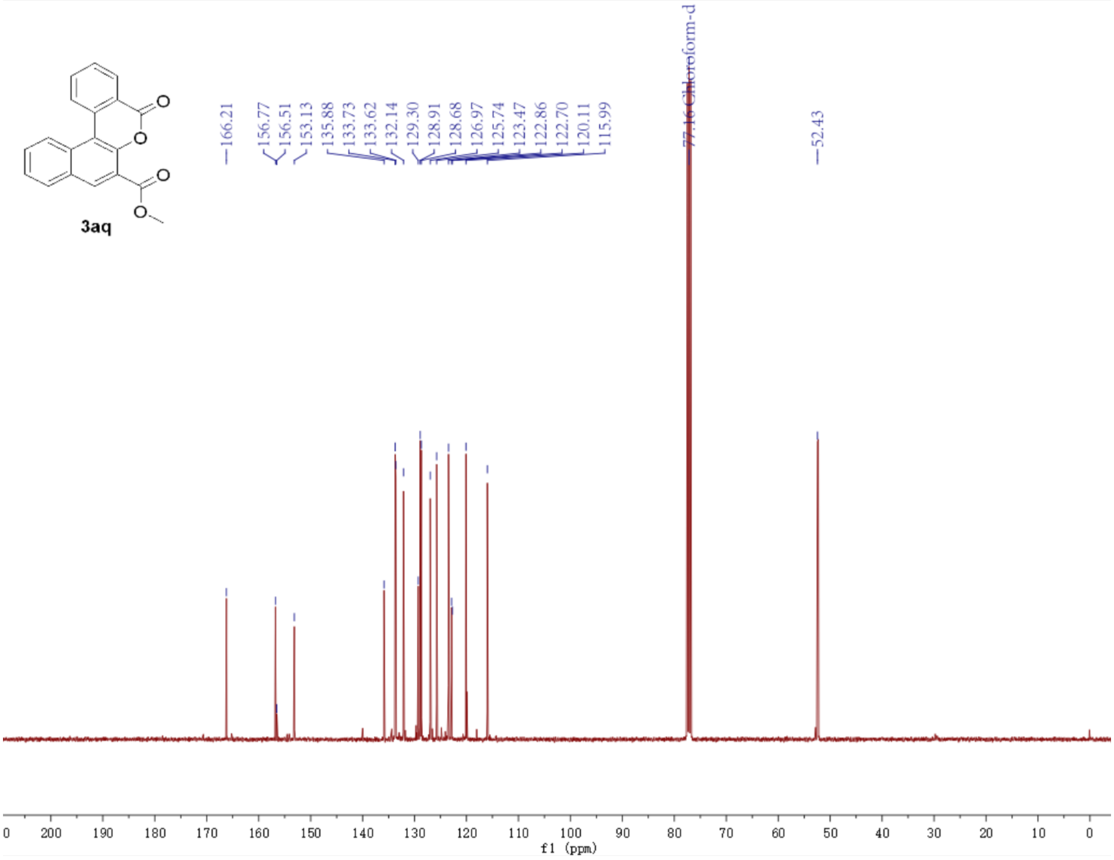
3aq
166.21
156.77
156.51
153.13
135.88
133.73
133.62
132.14
129.30
128.91
128.68
126.97
125.74
123.47
122.86
122.70
120.11
115.99
77.16 Chloroform-d
52.43
f1 (ppm)

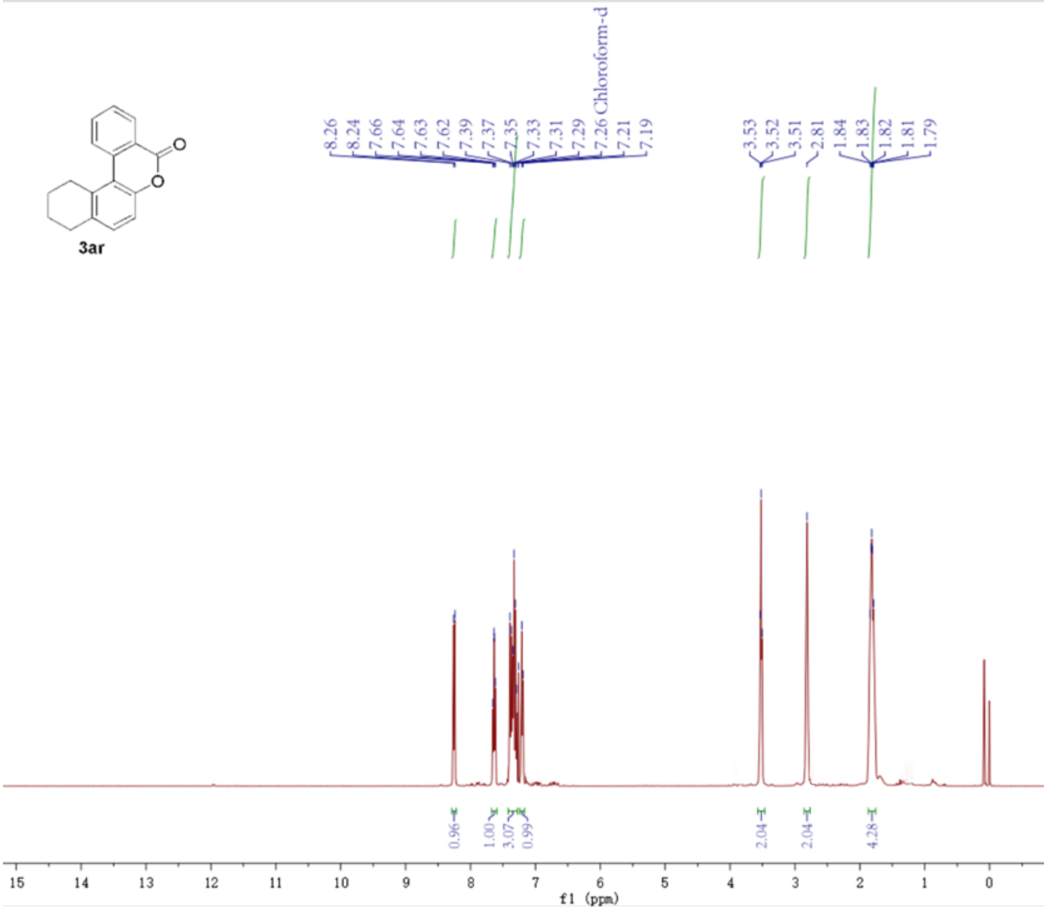

3ar
8.26
8.24
7.66
7.64
7.63
7.62
7.39
7.37
7.35
7.33
7.31
7.29
7.26 Chloroform-d
7.21
7.19
3.53
3.52
3.51
2.81
1.84
1.83
1.82
1.81
1.79
0.96
1.00
3.07
0.99
2.04
2.04
4.28
f1 (ppm)

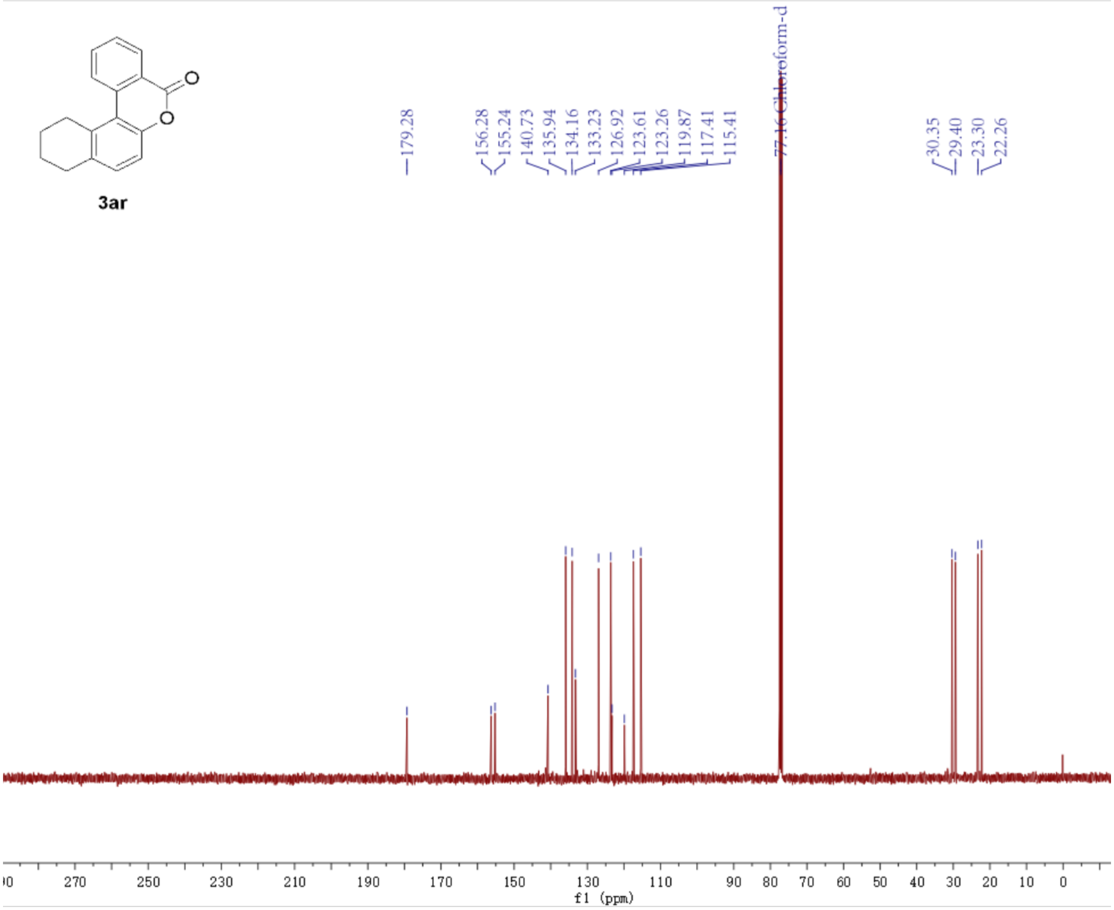

3ar
179.28
156.28
155.24
140.73
135.94
134.16
133.23
126.92
123.61
123.26
119.87
117.41
115.41
77.16 Chloroform-d
30.35
29.40
23.30
22.26
f1 (ppm)

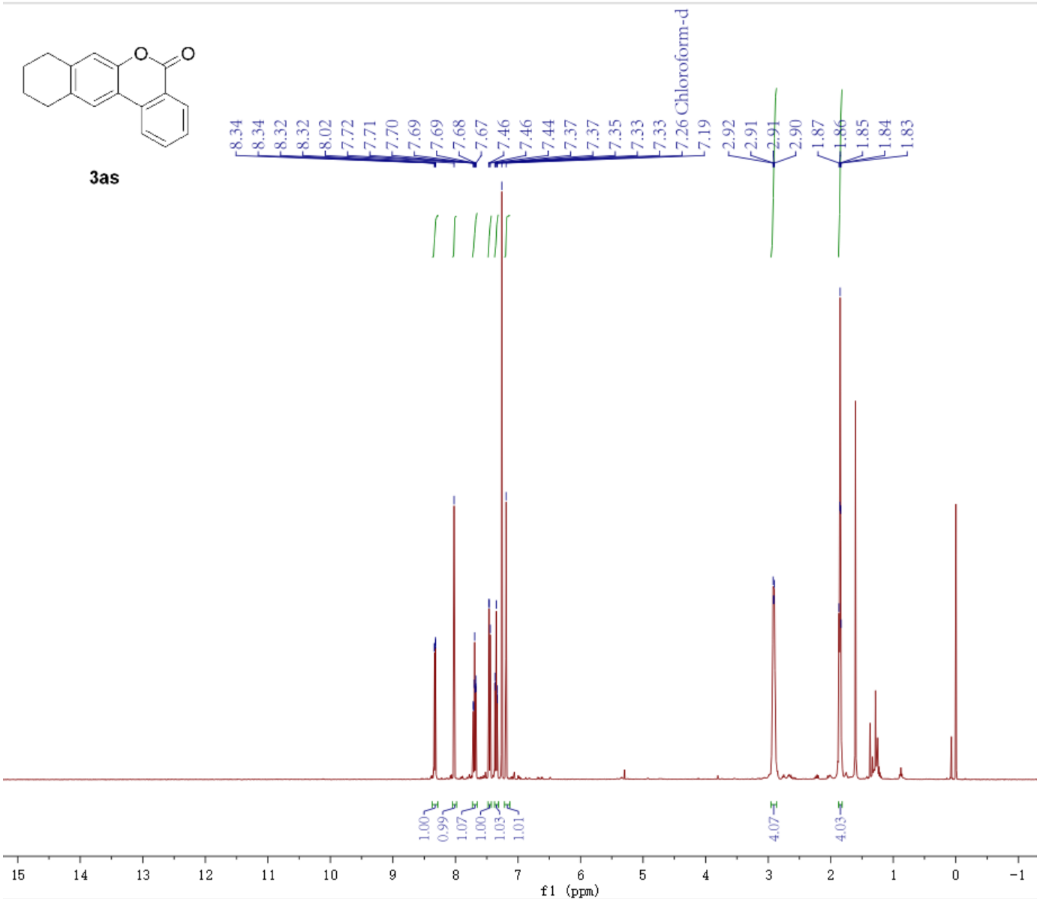
8.34
8.34
8.32
8.32
8.02
7.72
7.71
7.70
7.69
7.68
7.67
7.46
7.46
7.44
7.37
7.37
7.35
7.33
7.33
7.26 Chloroform-d
7.19
2.92
2.91
2.91
2.90
1.87
1.86
1.85
1.84
1.83
1.00
0.99
1.07
1.00
1.03
1.01
4.07
4.03
f1 (ppm)

3as

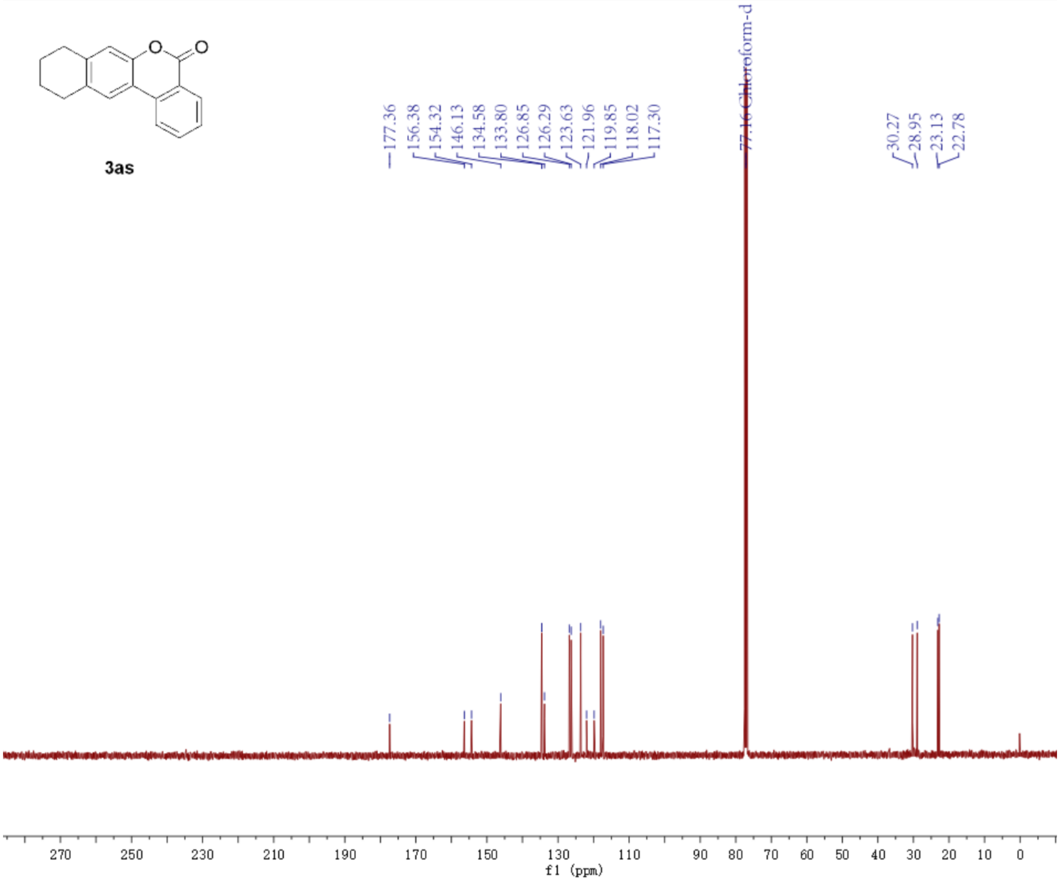
177.36
156.38
154.32
146.13
134.58
133.80
126.85
126.29
123.63
121.96
119.85
118.02
117.30
77.16 Chloroform-d
30.27
28.95
23.13
22.78
f1 (ppm)

3as

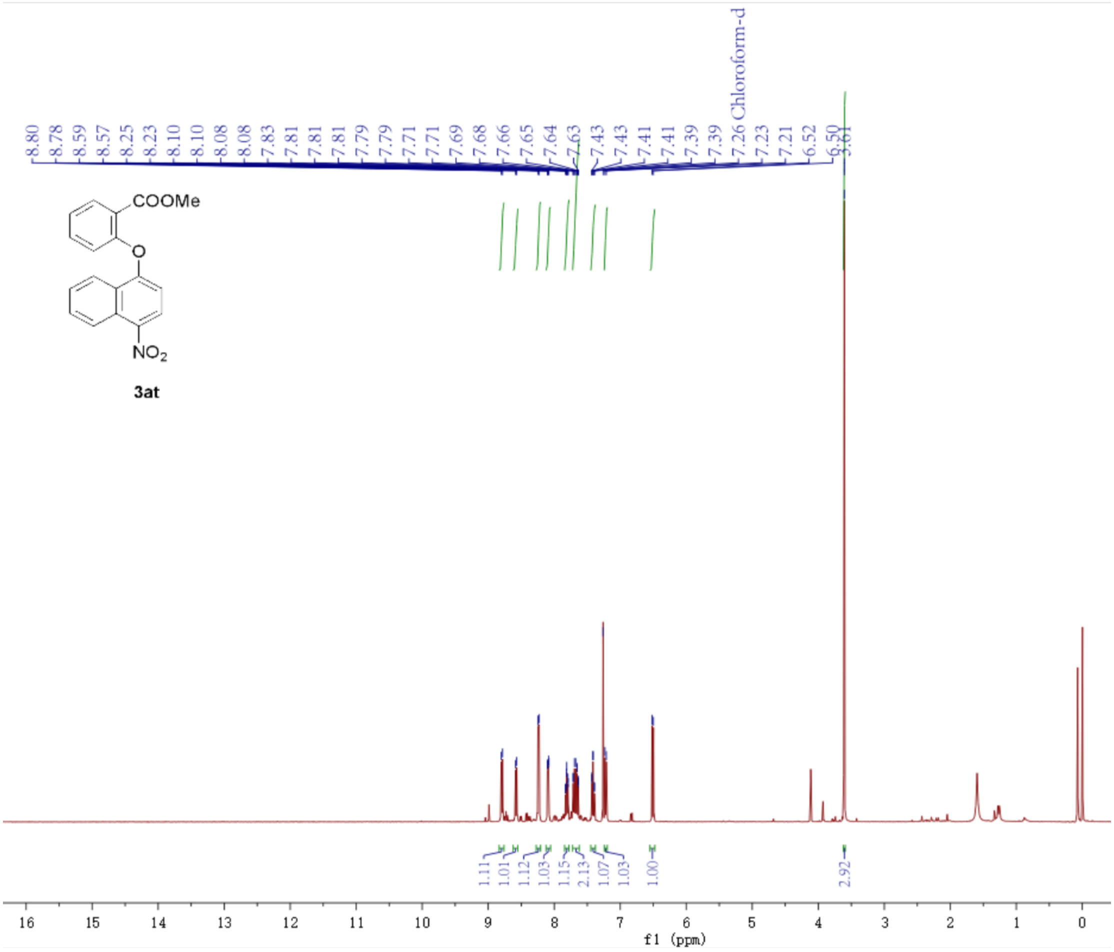
8.80
8.78
8.59
8.57
8.25
8.23
8.10
8.10
8.08
8.08
7.83
7.81
7.81
7.81
7.79
7.79
7.71
7.71
7.69
7.68
7.66
7.65
7.64
7.63
7.43
7.43
7.41
7.41
7.39
7.39
7.26 Chloroform-d
7.23
7.21
6.52
6.50
3.61
COOMe
O
NO_2
3at
1.11
1.01
1.12
1.03
1.15
2.13
1.07
1.03
1.00
2.92
f1 (ppm)

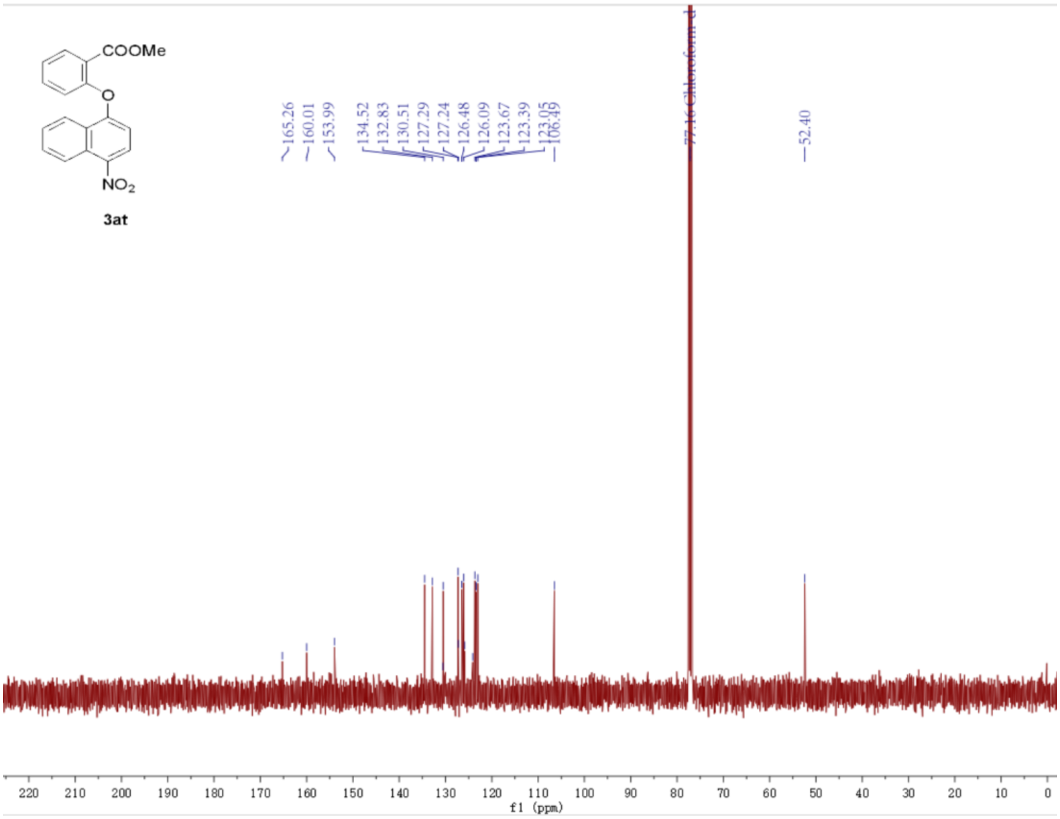
165.26
160.01
153.99
134.52
132.83
130.51
127.29
127.24
126.48
126.09
123.67
123.39
123.05
106.49
77.16 Chloroform-d
52.40
COOMe
O
NO_2
3at
f1 (ppm)

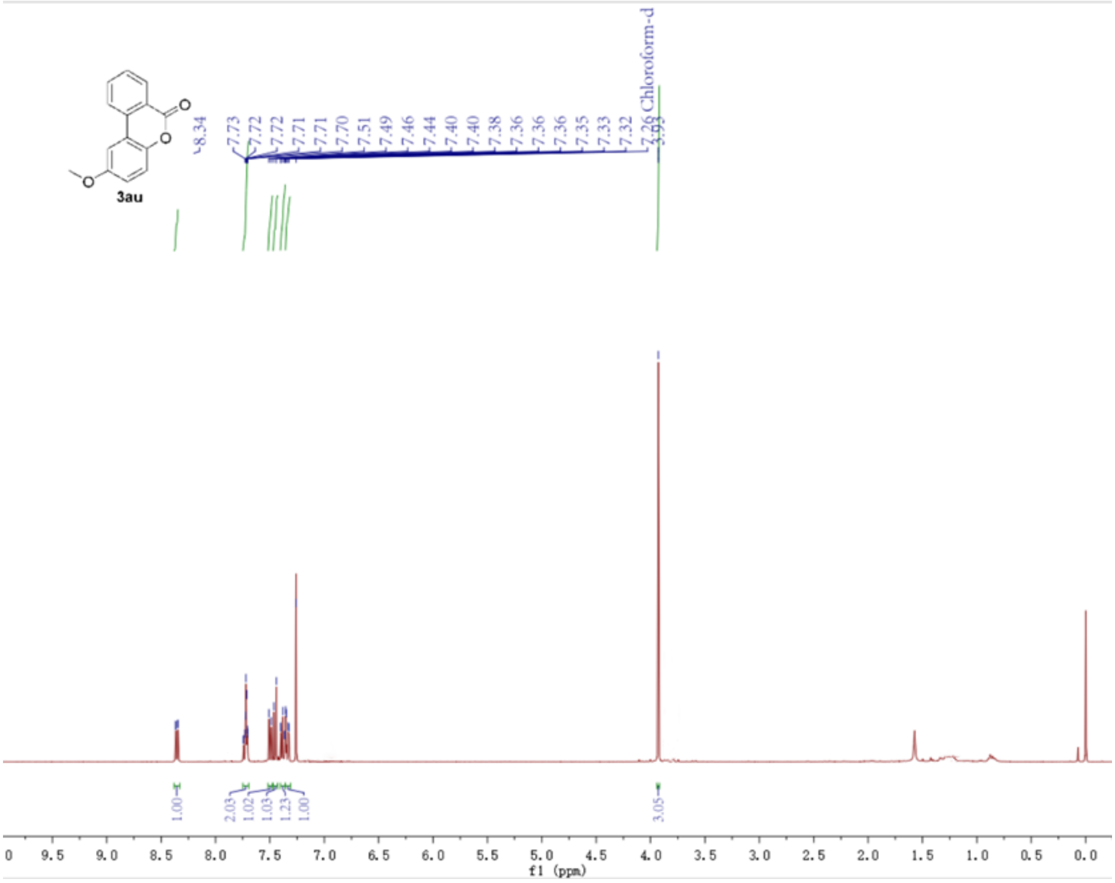
3au
8.34
7.73 7.72 7.72 7.71 7.71 7.70 7.51 7.49 7.46 7.44 7.40 7.40 7.38 7.36 7.36 7.36 7.35 7.33 7.32 7.26 Chloroform-d
3.93
1.00
2.03 1.02 1.03 1.23 1.00
3.05
f1 (ppm)

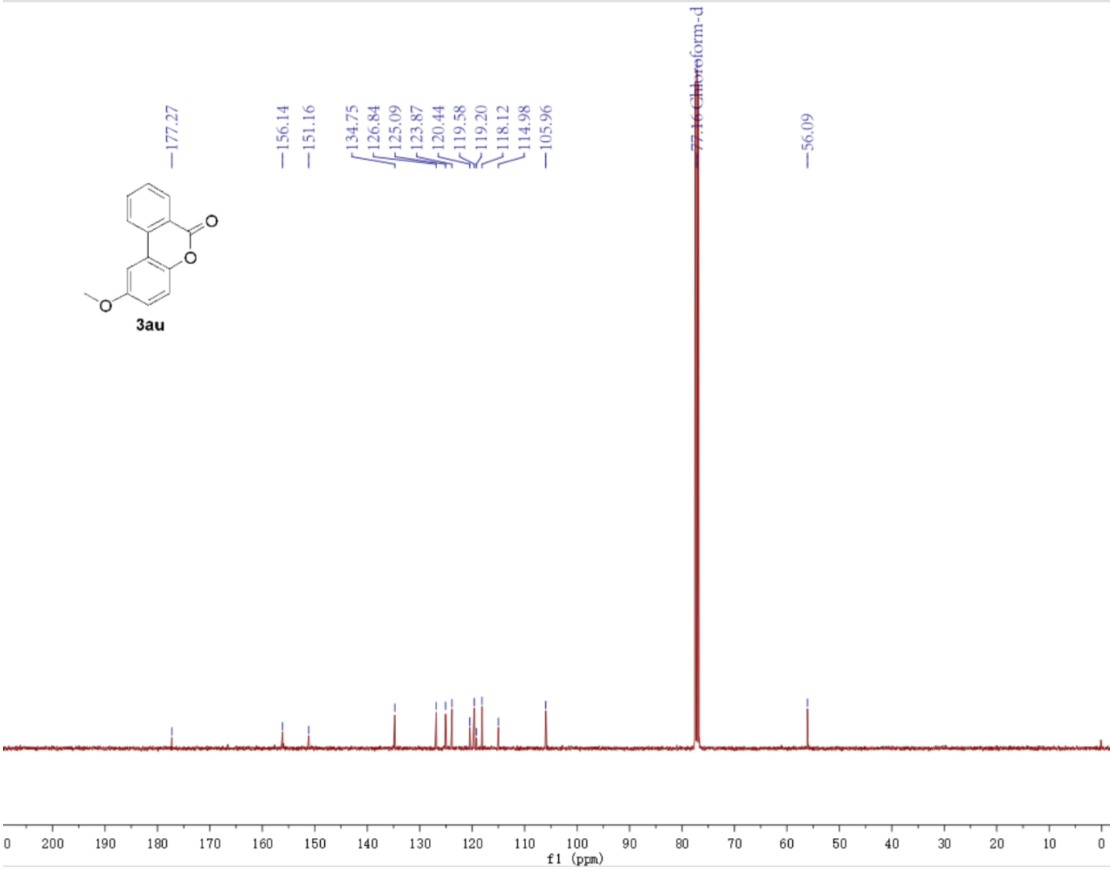
3au
177.27
156.14
151.16
134.75 126.84 125.09 123.87 120.44 119.58 119.20 118.12 114.98
105.96
77.16 Chloroform-d
56.09
f1 (ppm)

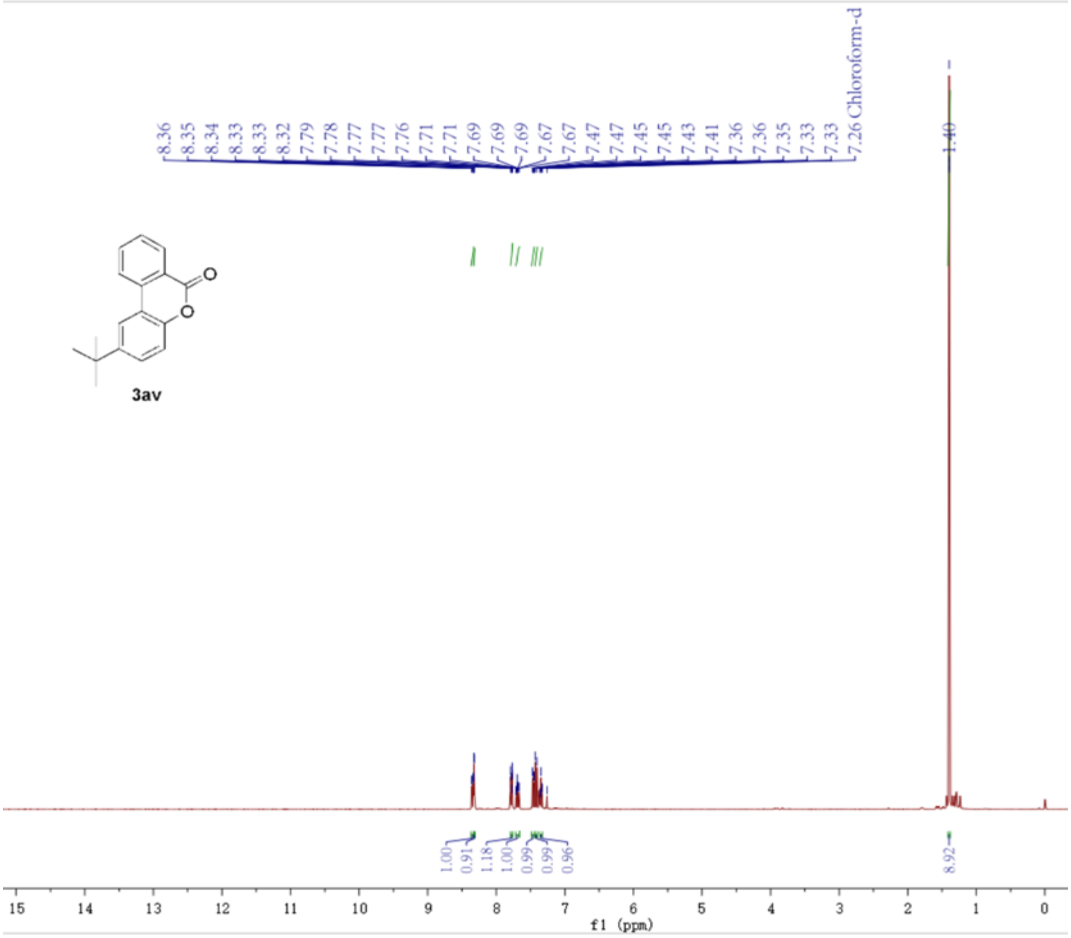
8.36
8.35
8.34
8.33
8.33
8.32
7.79
7.78
7.77
7.77
7.76
7.71
7.71
7.69
7.69
7.69
7.67
7.67
7.47
7.47
7.45
7.45
7.43
7.41
7.36
7.36
7.35
7.33
7.33
7.26 Chloroform-d
1.40
3av
1.00
0.91
1.18
1.00
0.99
0.99
0.96
8.92
15 14 13 12 11 10 9 8 7 6 5 4 3 2 1 0
f1 (ppm)

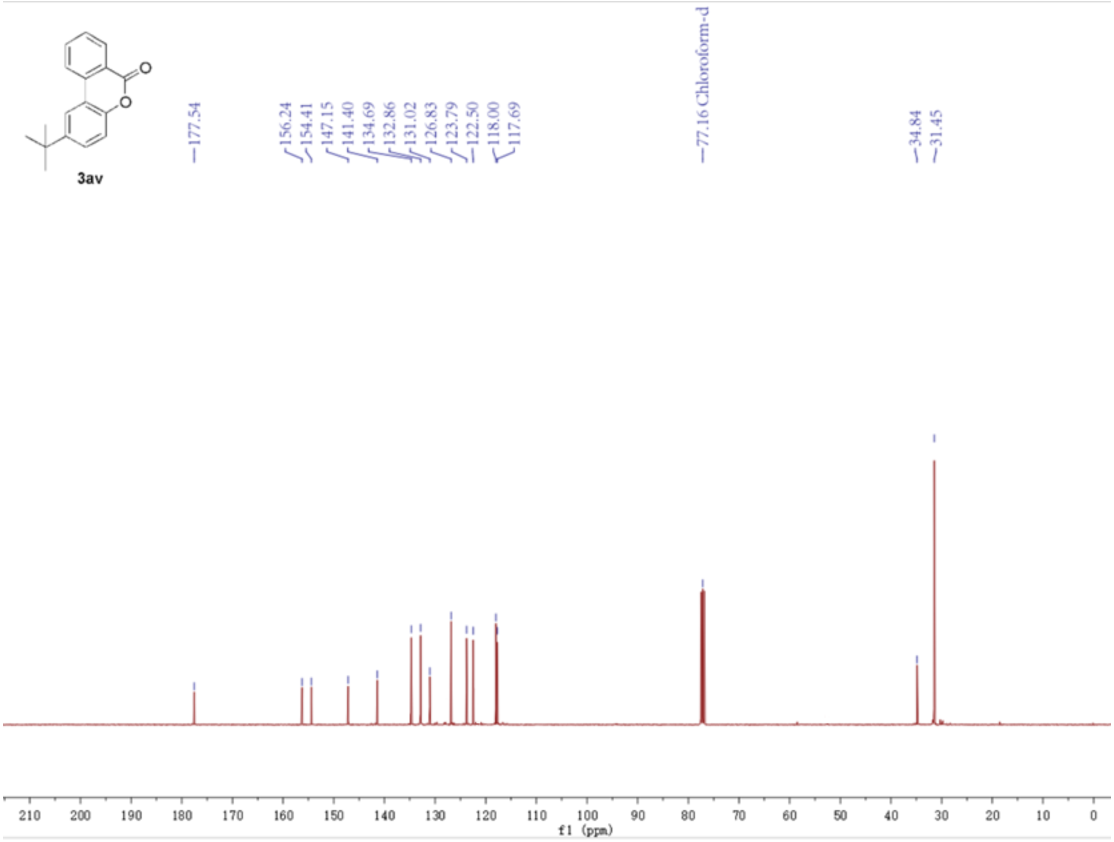
3av
177.54
156.24
154.41
147.15
141.40
134.69
132.86
131.02
126.83
123.79
122.50
118.00
117.69
77.16 Chloroform-d
34.84
31.45
210 200 190 180 170 160 150 140 130 120 110 100 90 80 70 60 50 40 30 20 10 0
f1 (ppm)

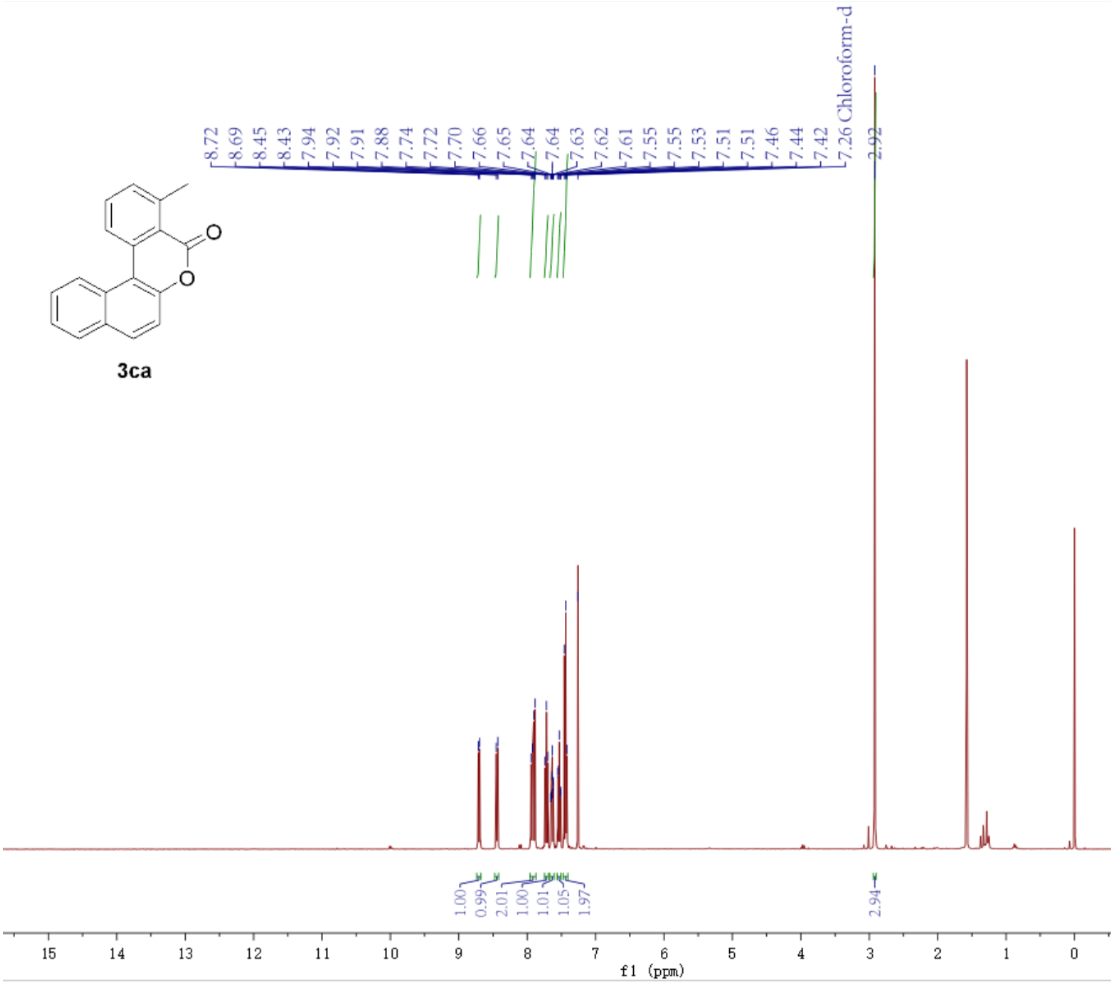

3ca

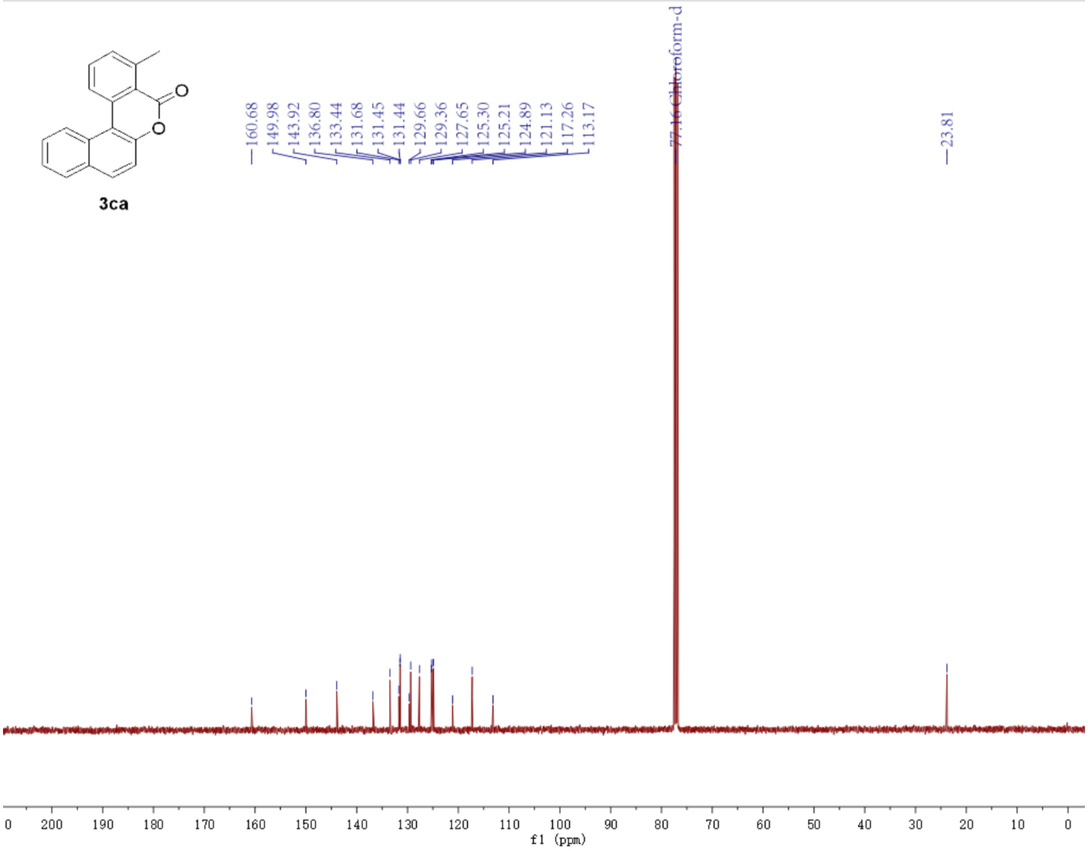

3ca

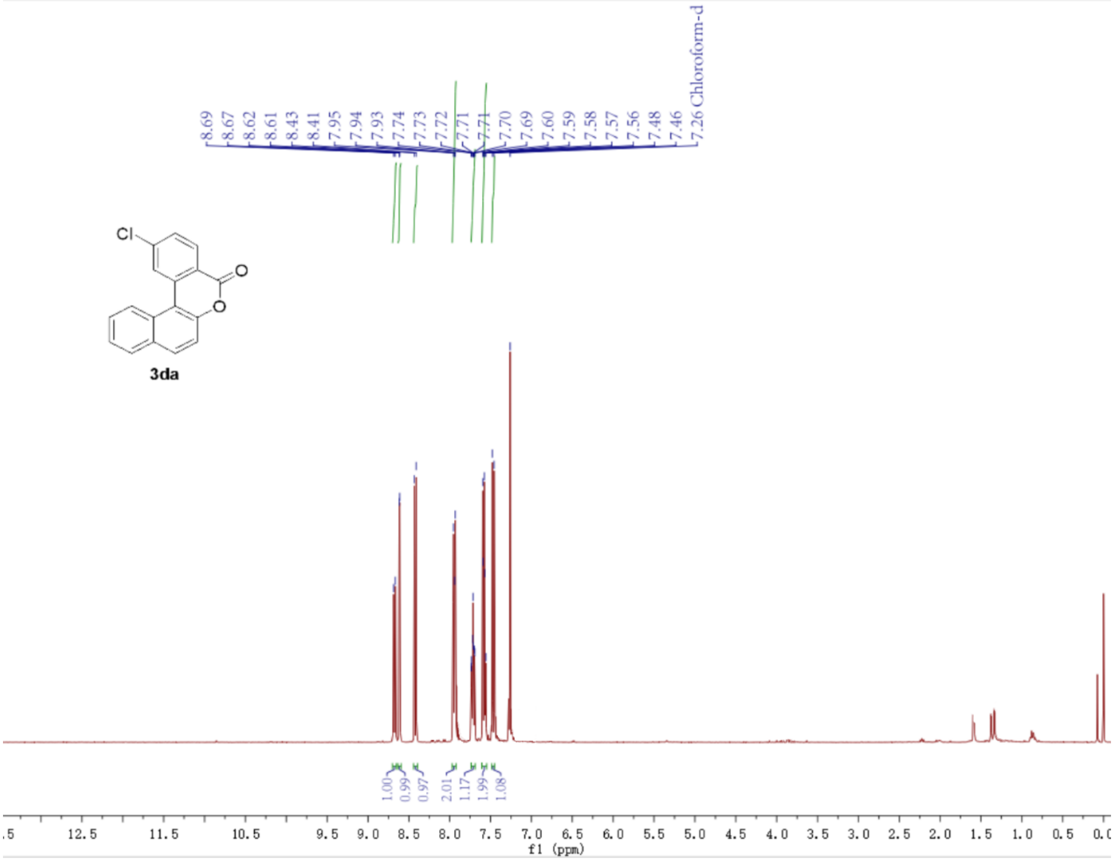
8.69
8.67
8.62
8.61
8.43
8.41
7.95
7.94
7.93
7.74
7.73
7.72
7.71
7.71
7.70
7.69
7.60
7.59
7.58
7.57
7.56
7.48
7.46
7.26 Chloroform-d
Cl
O
O
1.00
0.99
0.97
2.01
1.17
1.99
1.08
f1 (ppm)

3da

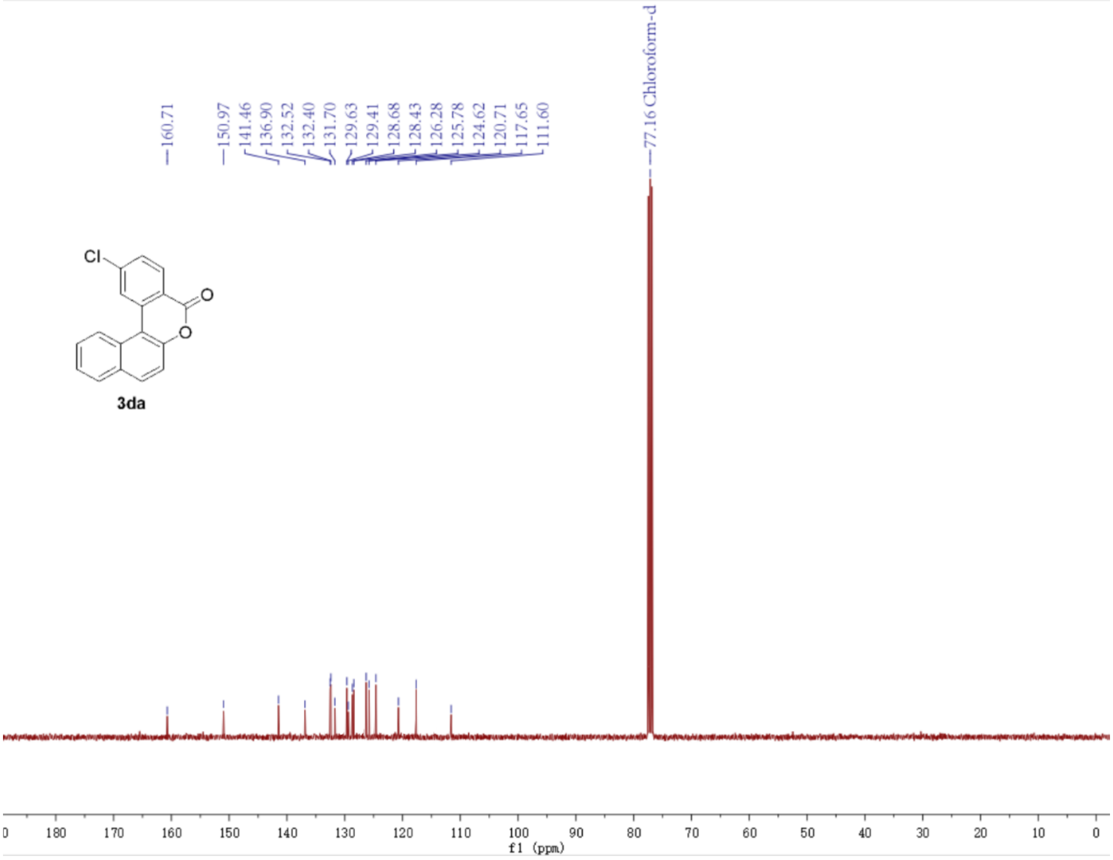
160.71
150.97
141.46
136.90
132.52
132.40
131.70
129.63
129.41
128.68
128.43
126.28
125.78
124.62
120.71
117.65
111.60
77.16 Chloroform-d
Cl
O
O
f1 (ppm)

3da

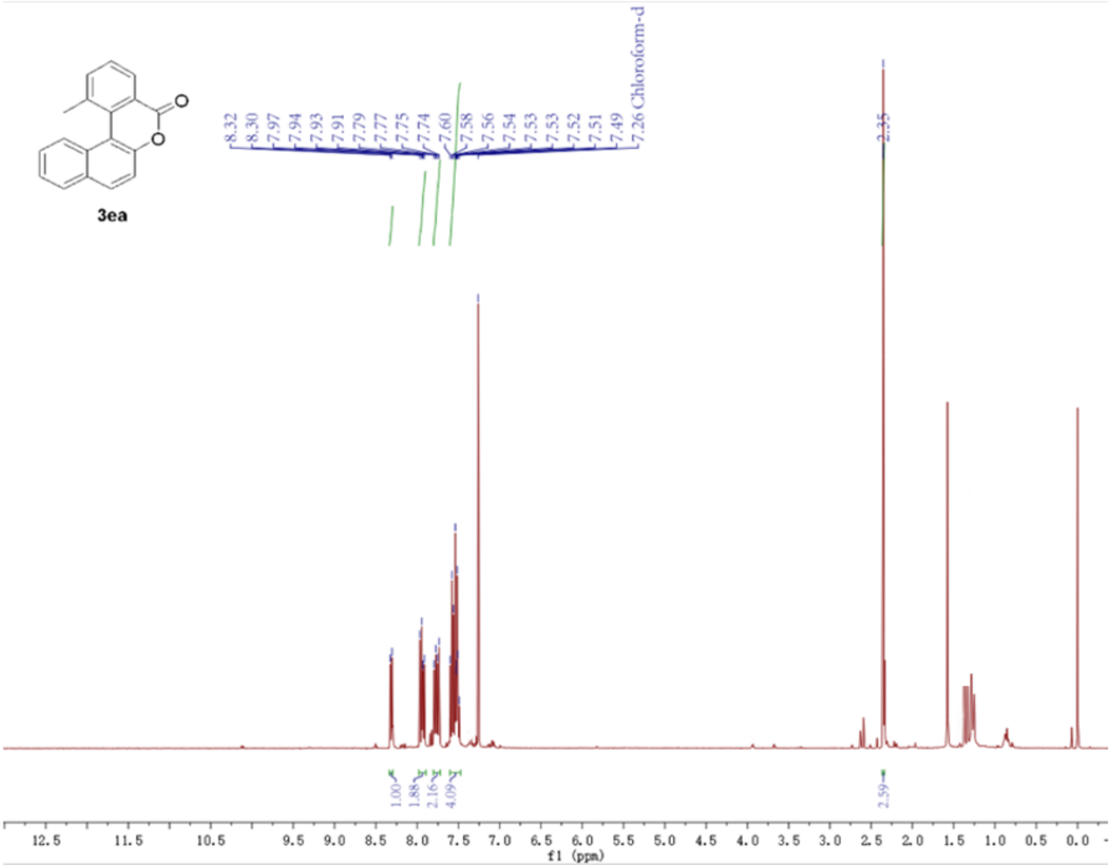
3ea
8.32
8.30
7.97
7.94
7.93
7.91
7.79
7.77
7.75
7.74
7.60
7.58
7.56
7.54
7.53
7.53
7.52
7.51
7.49
7.26 Chloroform-d
2.35
1.00
1.88
2.16
4.09
2.59
12.5 11.5 10.5 9.5 9.0 8.5 8.0 7.5 7.0 6.5 6.0 5.5 5.0 4.5 4.0 3.5 3.0 2.5 2.0 1.5 1.0 0.5 0.0
f1 (ppm)

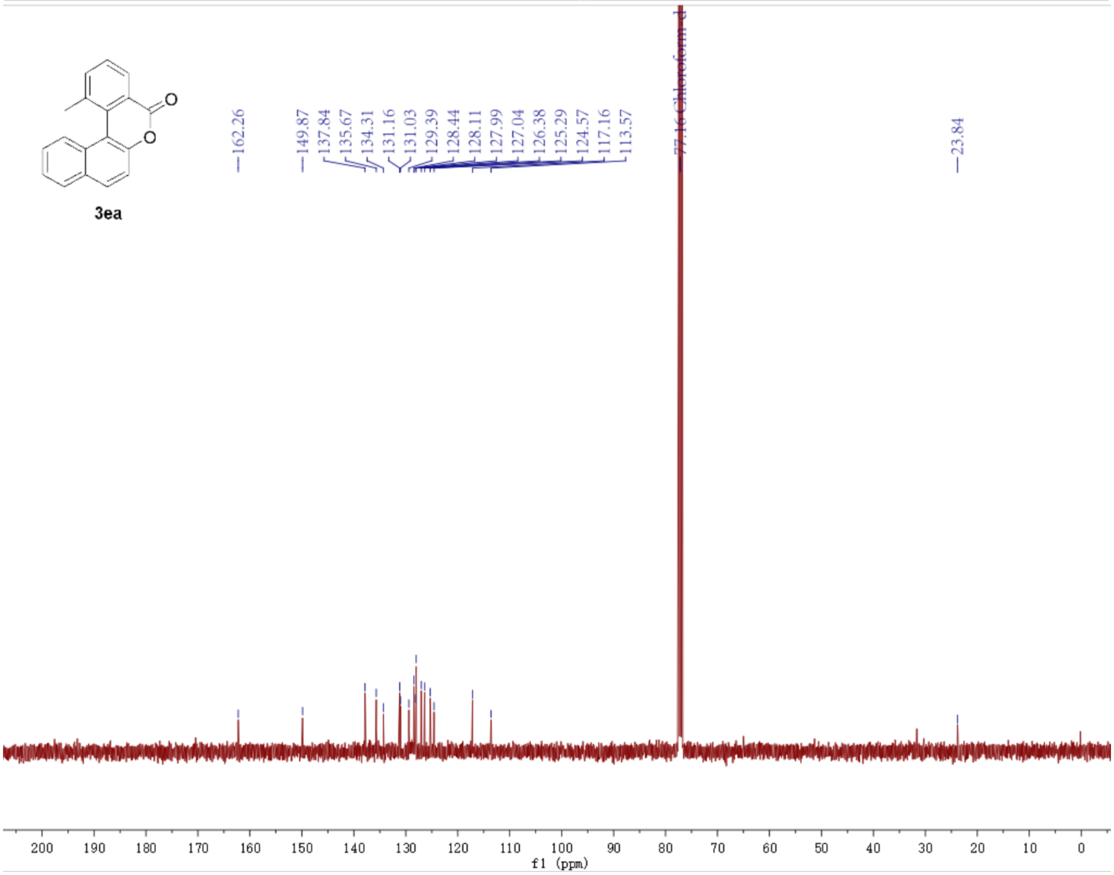
3ea
162.26
149.87
137.84
135.67
134.31
131.16
131.03
129.39
128.44
128.11
127.99
127.04
126.38
125.29
124.57
117.16
113.57
77.16 Chloroform-d
23.84
200 190 180 170 160 150 140 130 120 110 100 90 80 70 60 50 40 30 20 10 0
f1 (ppm)

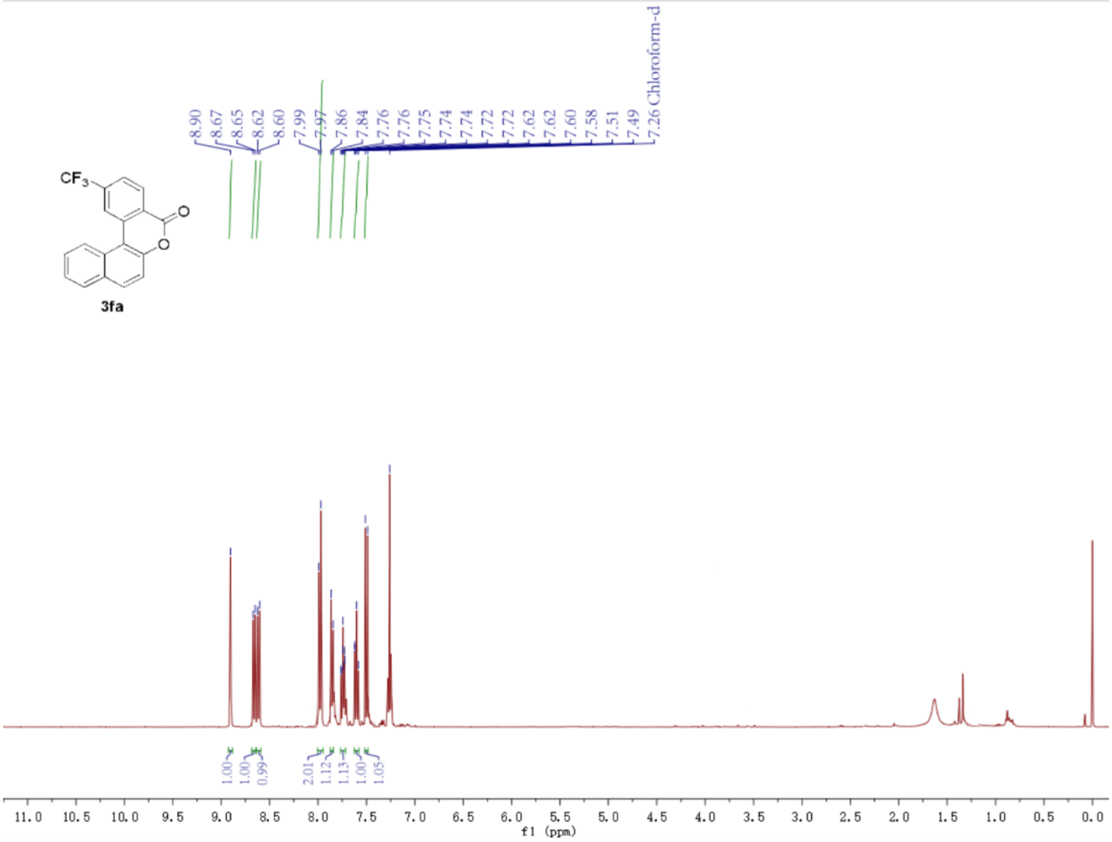
8.90
8.67
8.65
8.62
8.60
7.99
7.97
7.86
7.84
7.76
7.76
7.75
7.74
7.74
7.72
7.72
7.62
7.62
7.60
7.58
7.51
7.49
7.26 Chloroform-d
CF3
O
O
3fa
1.00
1.00
0.99
2.01
1.12
1.13
1.00
1.05
f1 (ppm)

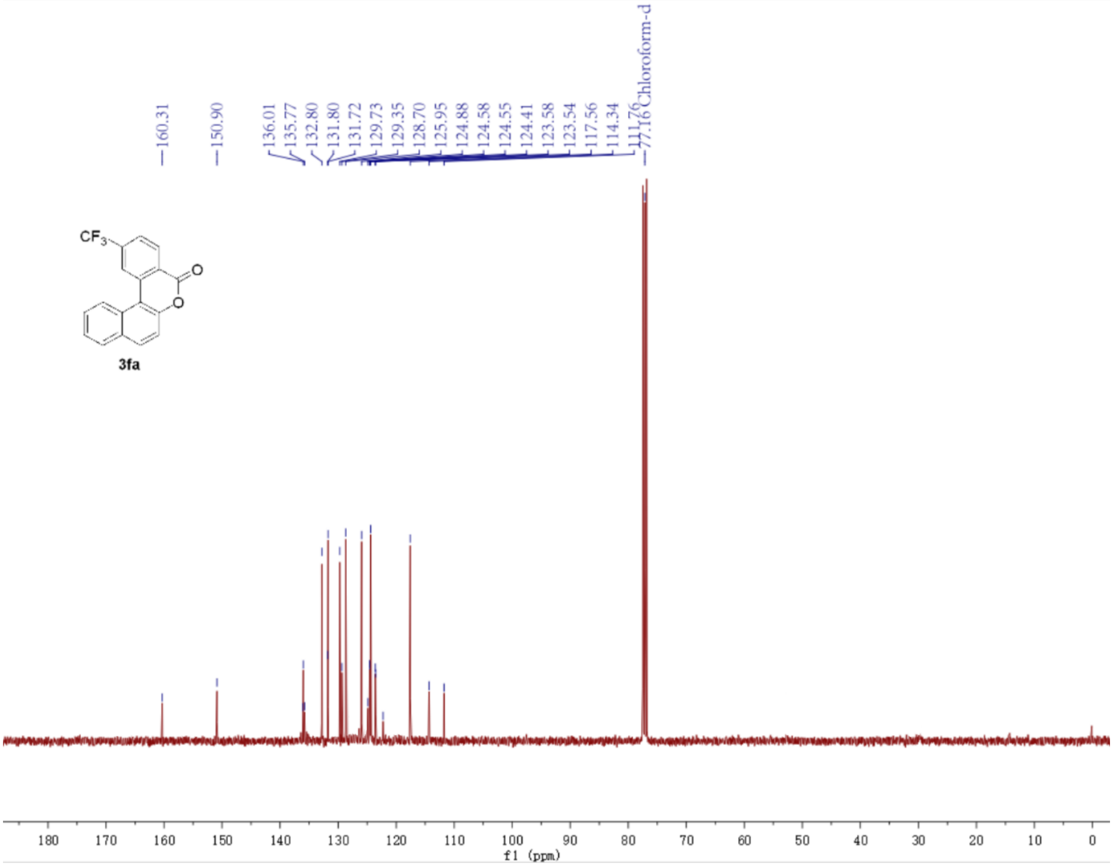
160.31
150.90
136.01
135.77
132.80
131.80
131.72
129.73
129.35
128.70
125.95
124.88
124.58
124.55
124.41
123.58
123.54
117.56
114.34
111.76
77.16 Chloroform-d
CF3
O
O
3fa
f1 (ppm)

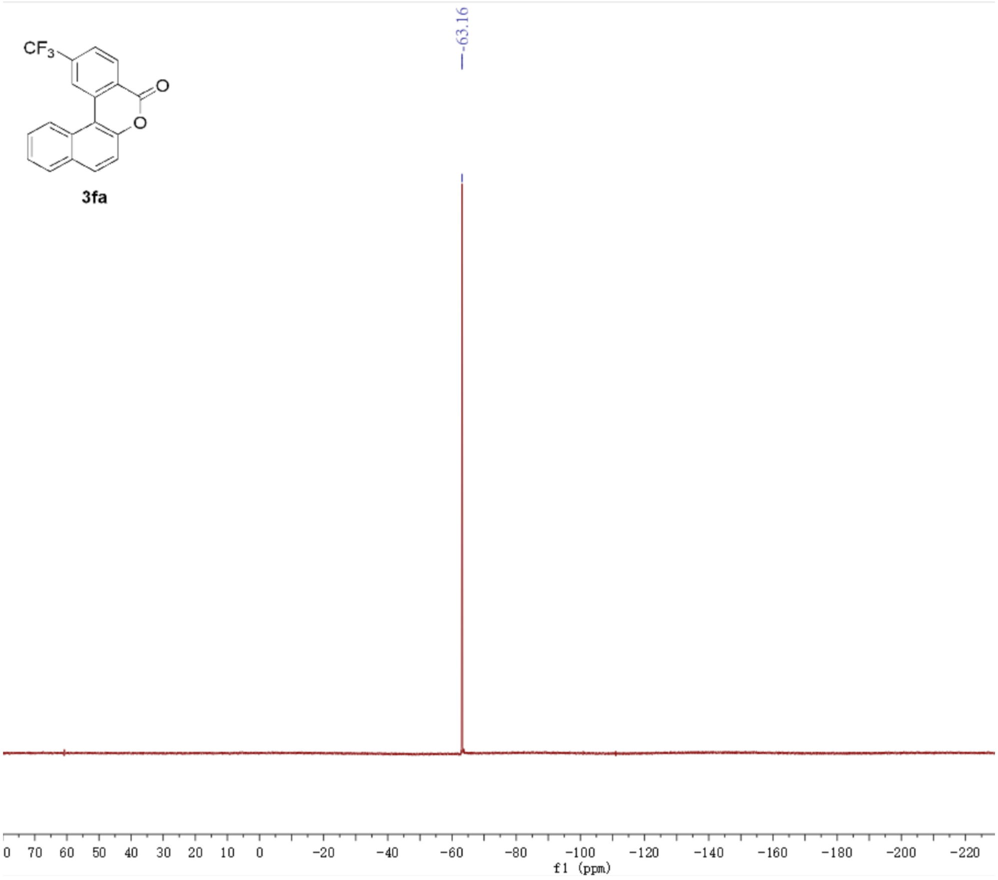

CF3
O
O
3fa
-63.16
0
70
60
50
40
30
20
10
0
-20
-40
-60
-80
-100
-120
-140
-160
-180
-200
-220
f1 (ppm)

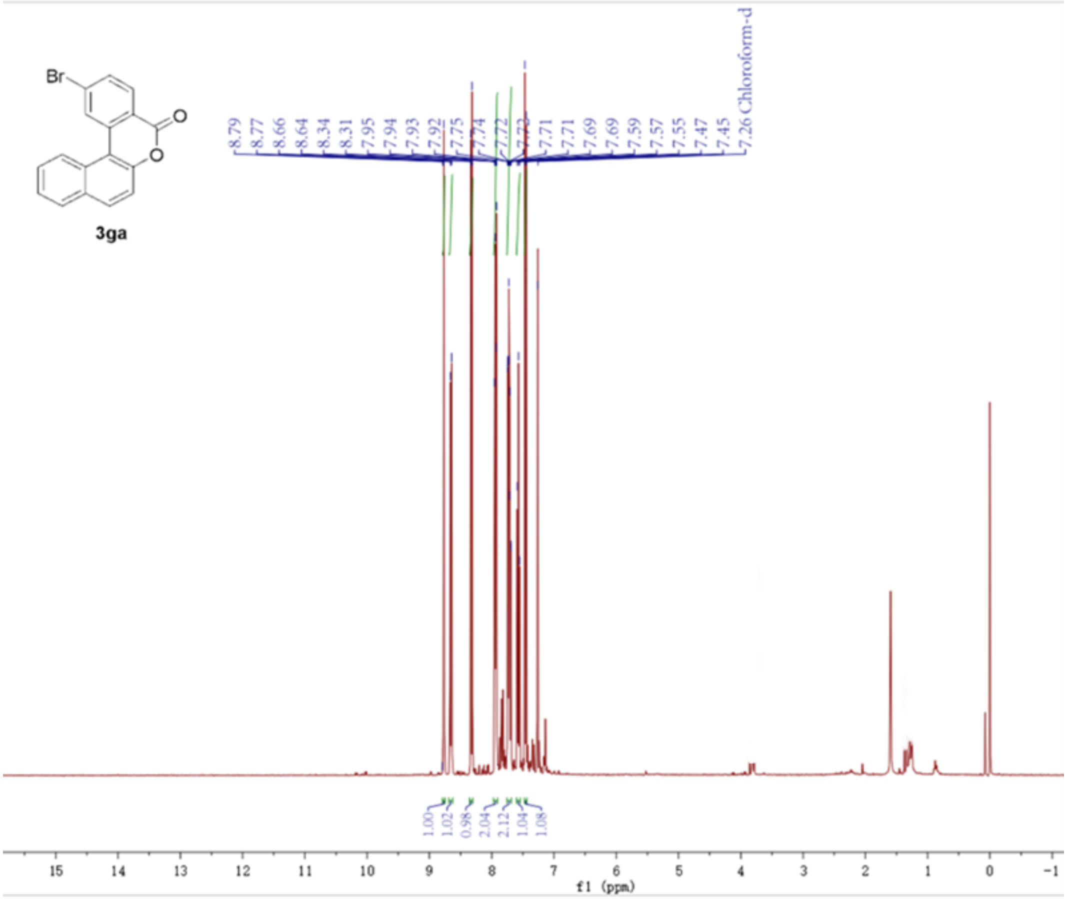

Br
O
O
3ga
8.79
8.77
8.66
8.64
8.34
8.31
7.95
7.94
7.93
7.92
7.75
7.74
7.72
7.72
7.71
7.71
7.69
7.69
7.59
7.57
7.55
7.47
7.45
7.26 Chloroform-d
1.00
1.02
0.98
2.04
2.12
1.04
1.08
15
14
13
12
11
10
9
8
7
6
5
4
3
2
1
0
-1
f1 (ppm)

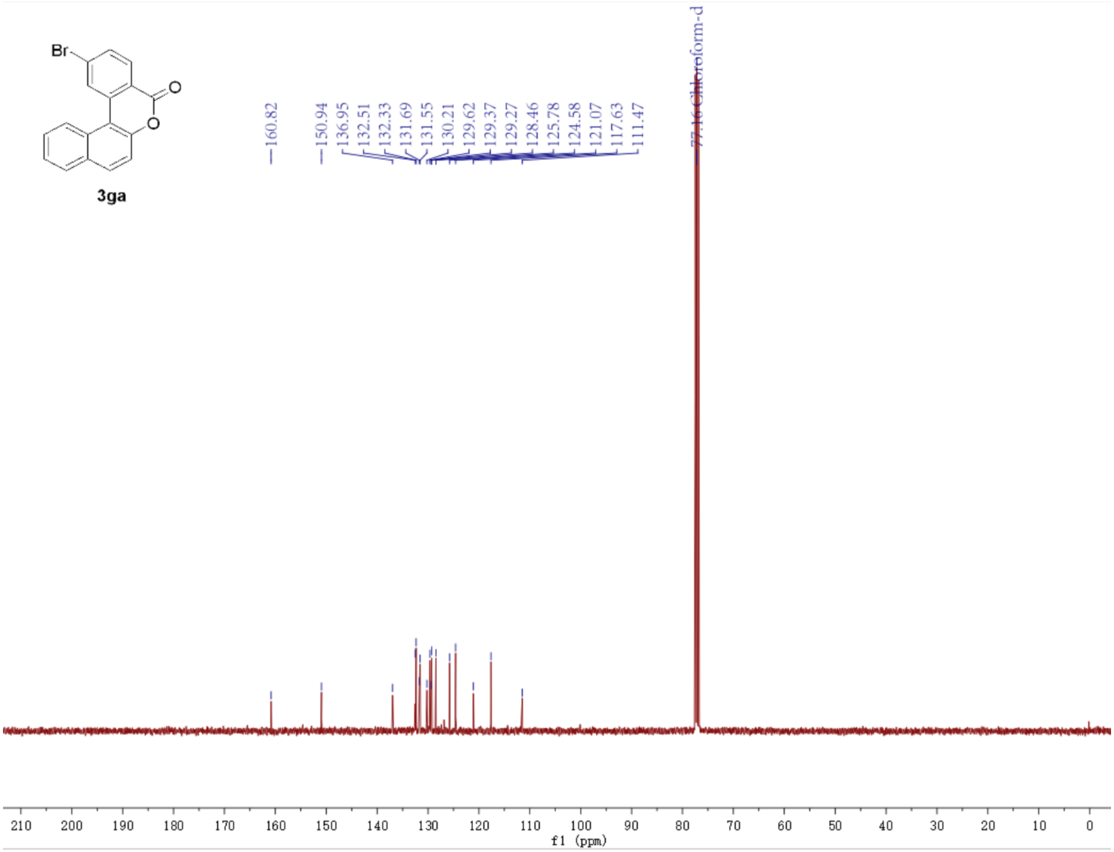

Br
O
O
3ga
160.82
150.94
136.95
132.51
132.33
131.69
131.55
130.21
129.62
129.37
129.27
128.46
125.78
124.58
121.07
117.63
111.47
77.16 Chloroform-d
210
200
190
180
170
160
150
140
130
120
110
100
90
80
70
60
50
40
30
20
10
0
f1 (ppm)

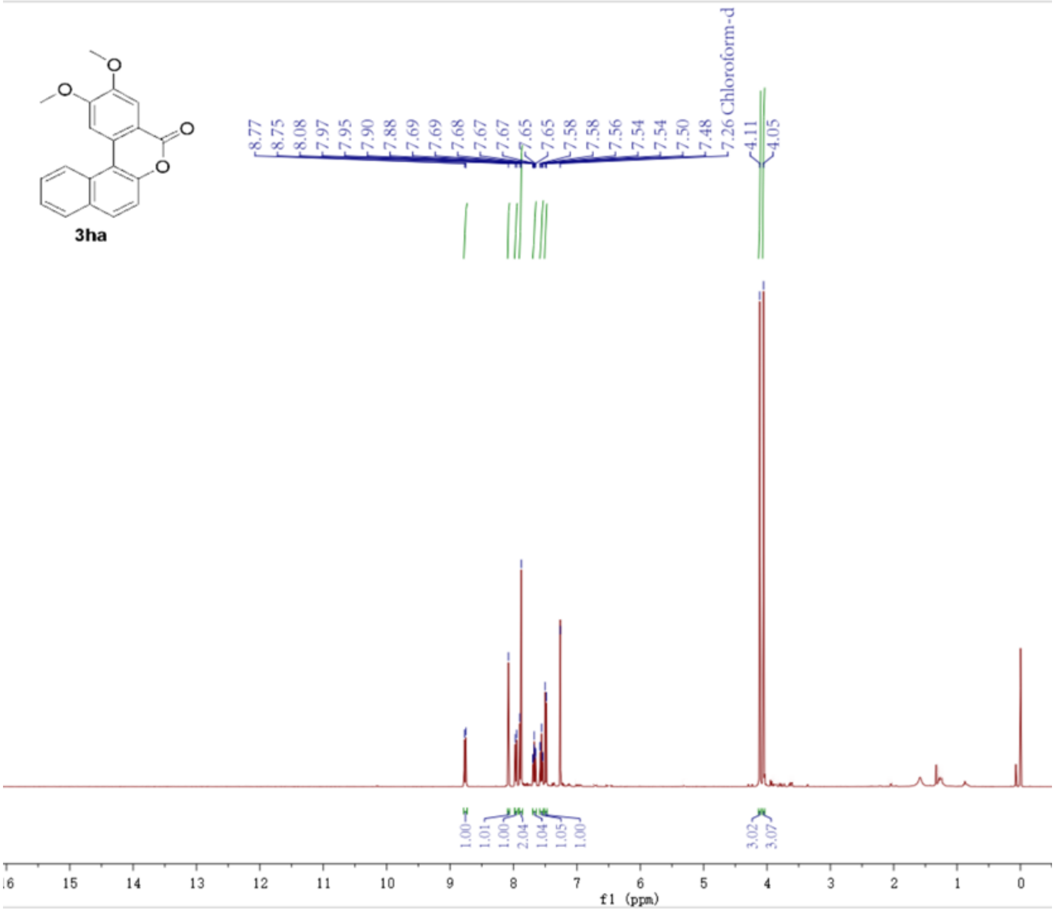

O
O
O
O
3ha
8.77
8.75
8.08
7.97
7.95
7.90
7.88
7.69
7.69
7.68
7.67
7.67
7.65
7.65
7.58
7.56
7.54
7.50
7.48
7.26 Chloroform-d
4.11
4.05
1.00
1.01
1.00
2.04
1.04
1.05
1.00
3.02
3.07
16
15
14
13
12
11
10
9
8
7
6
5
4
3
2
1
0
f1 (ppm)

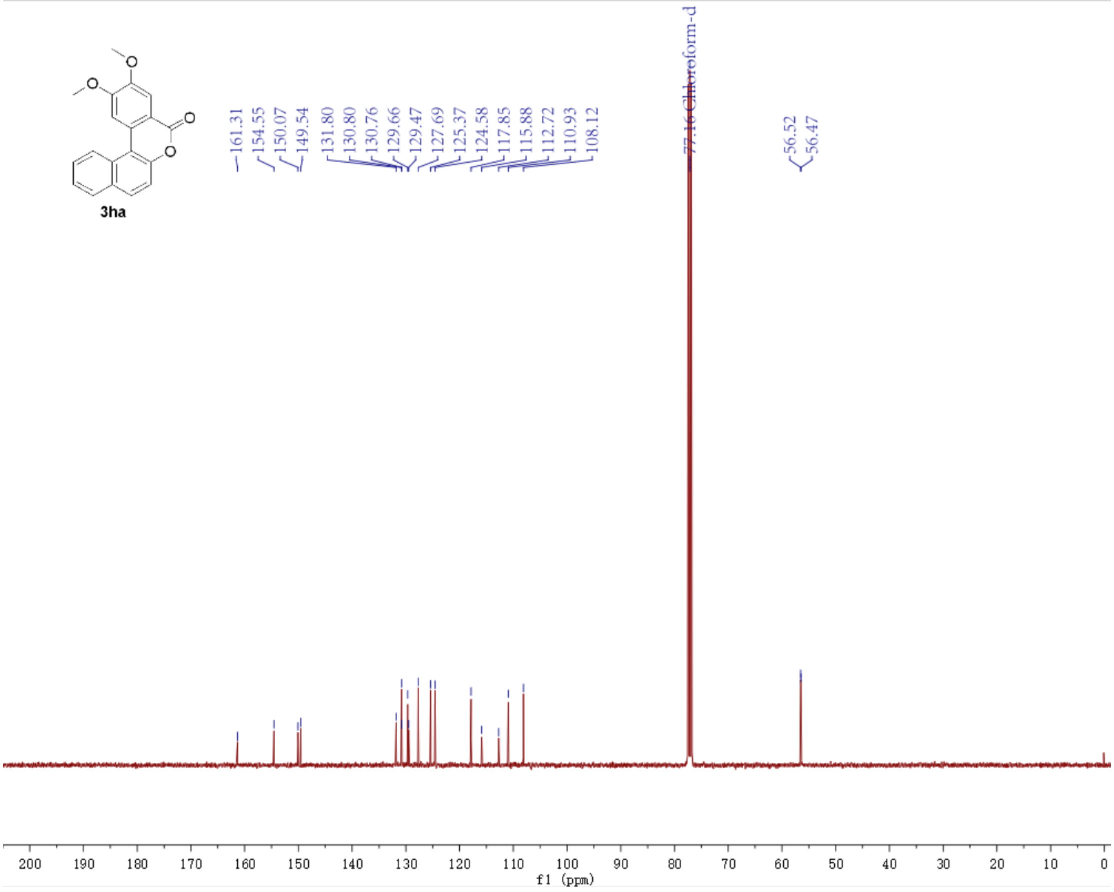
3ha
161.31
154.55
150.07
149.54
131.80
130.80
130.76
129.66
129.47
127.69
125.37
124.58
117.85
115.88
112.72
110.93
108.12
77.16 Chloroform-d
56.52
56.47
f1 (ppm)

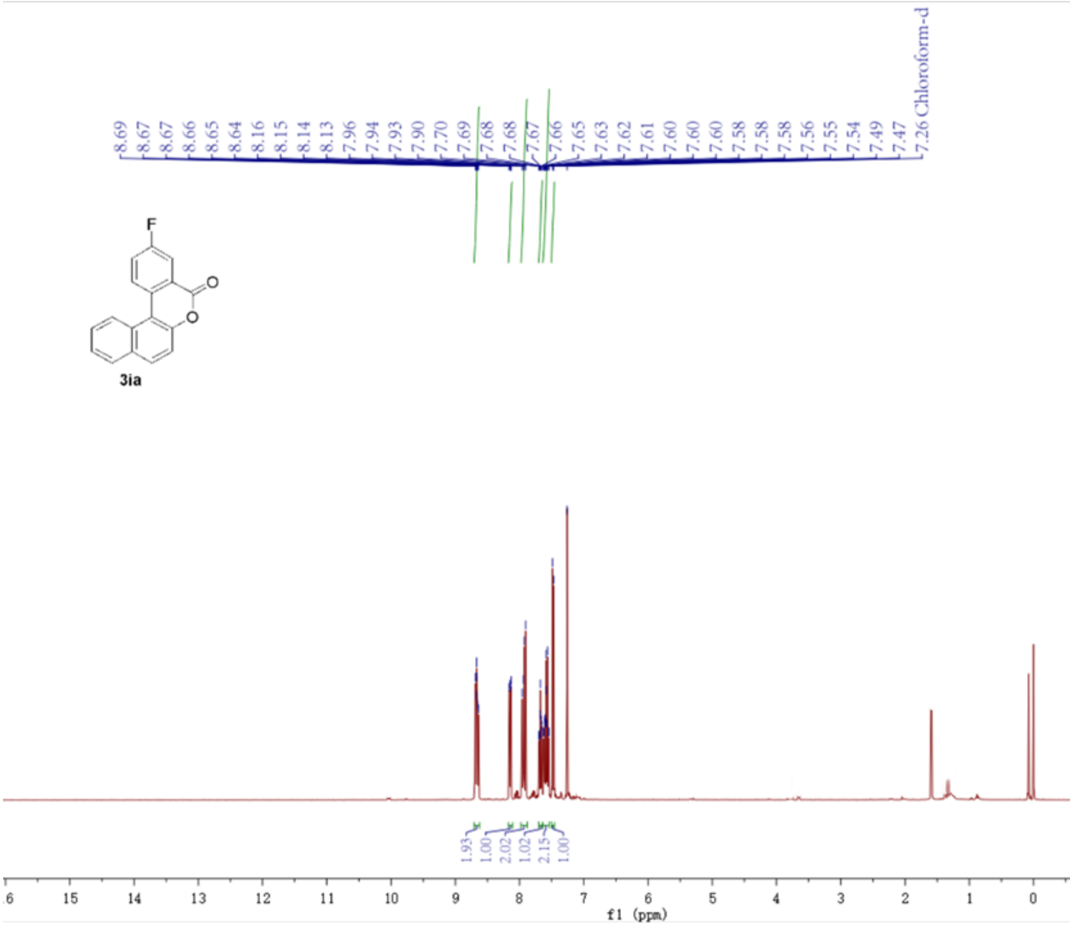
3ia
8.69
8.67
8.67
8.66
8.65
8.64
8.16
8.15
8.14
8.13
7.96
7.94
7.93
7.90
7.70
7.69
7.68
7.68
7.67
7.66
7.65
7.63
7.62
7.61
7.60
7.60
7.60
7.58
7.58
7.58
7.56
7.55
7.54
7.49
7.47
7.26 Chloroform-d
1.93
1.00
2.02
1.02
2.15
1.00
f1 (ppm)

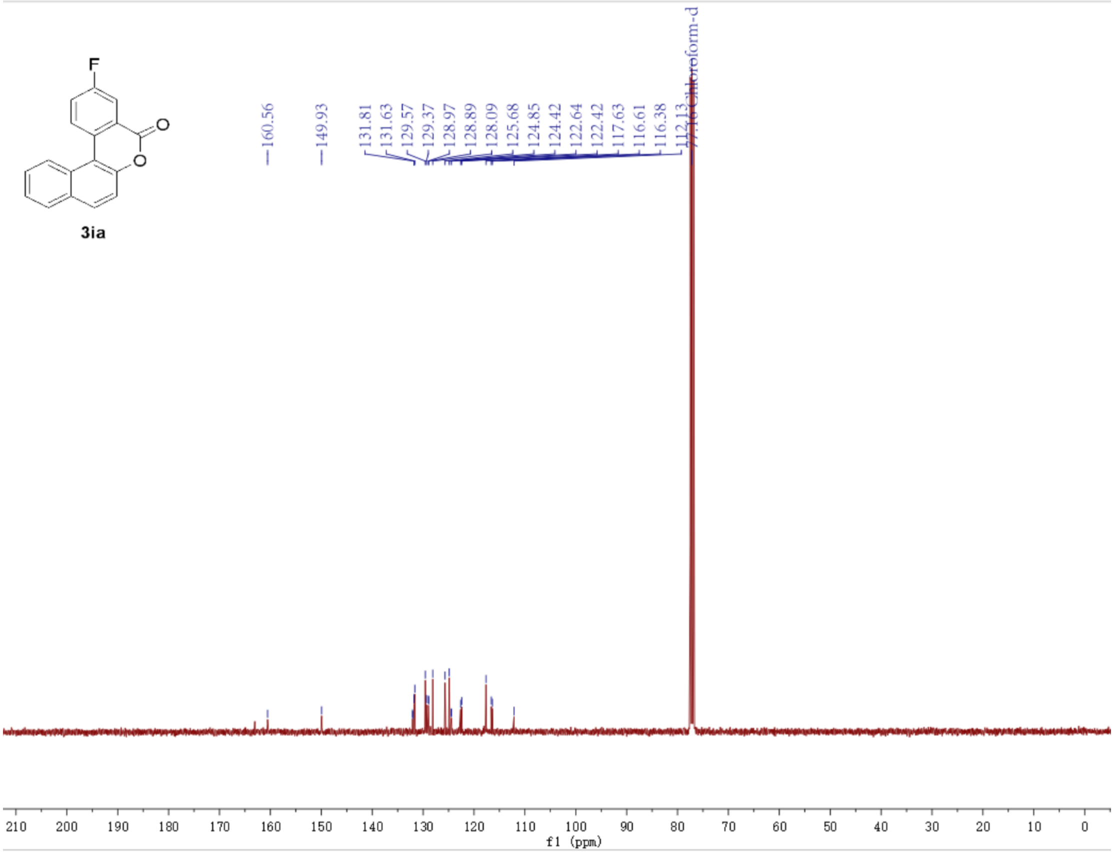
F
O
O
3ia
160.56
149.93
131.81
131.63
129.57
129.37
128.97
128.89
128.09
125.68
124.85
124.42
122.64
122.42
117.63
116.61
116.38
112.13
77.16 Chloroform-d
210 200 190 180 170 160 150 140 130 120 110 100 90 80 70 60 50 40 30 20 10 0
f1 (ppm)

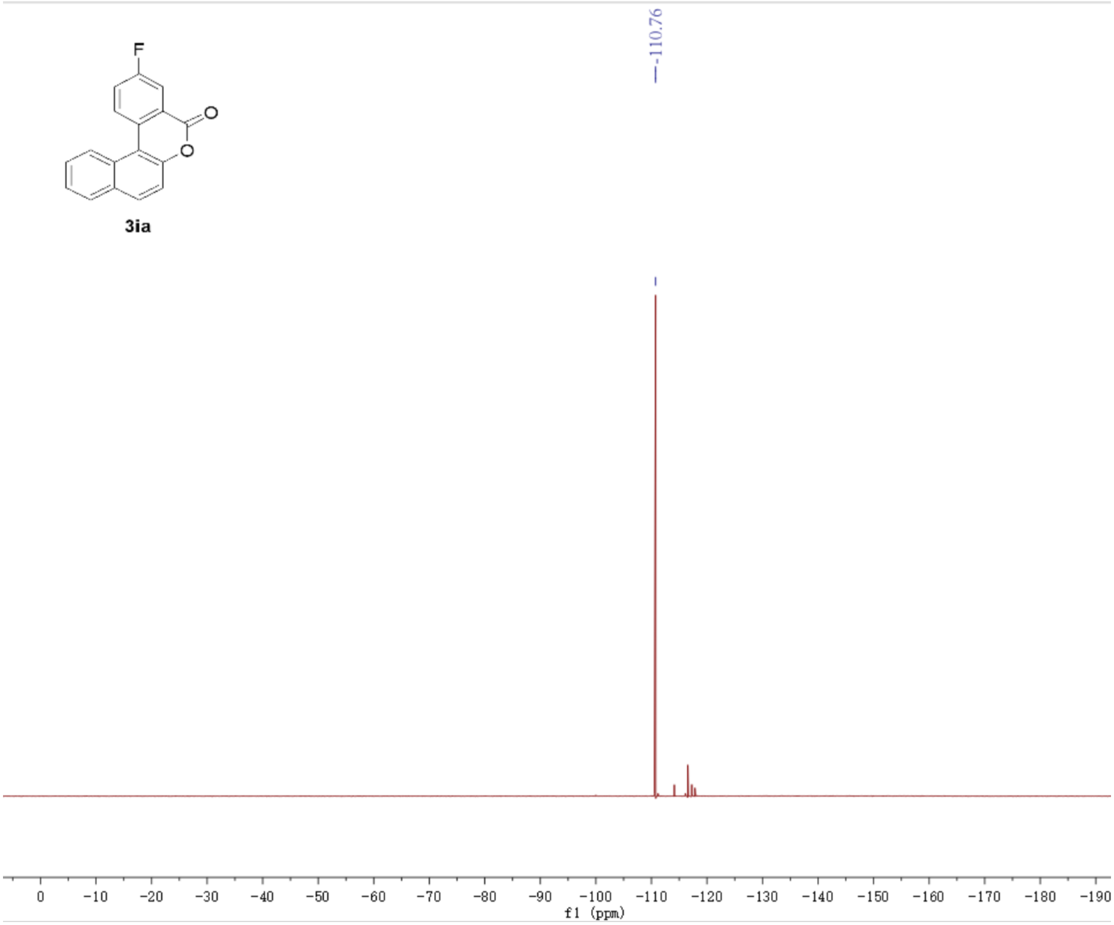
F
O
O
3ia
-110.76
0 −10 −20 −30 −40 −50 −60 −70 −80 −90 −100 −110 −120 −130 −140 −150 −160 −170 −180 −190
f1 (ppm)

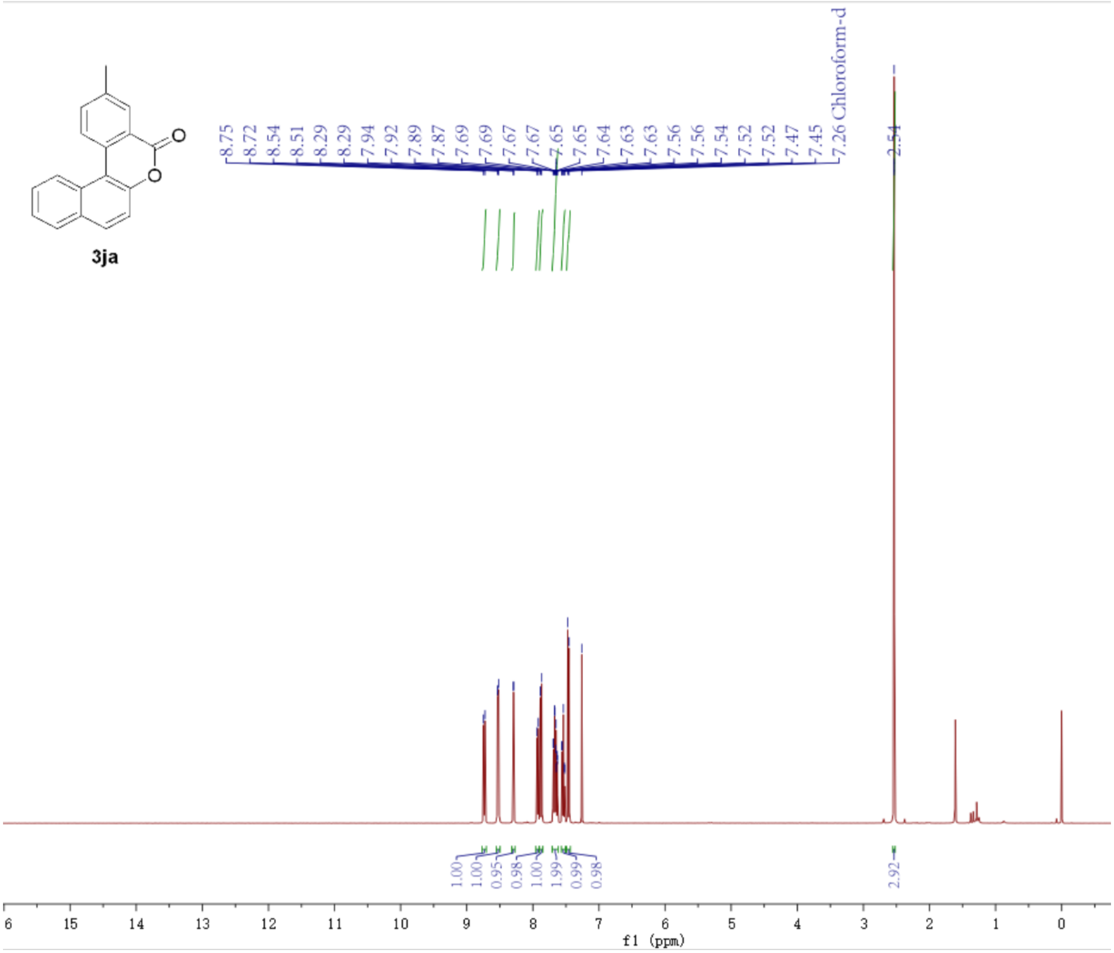

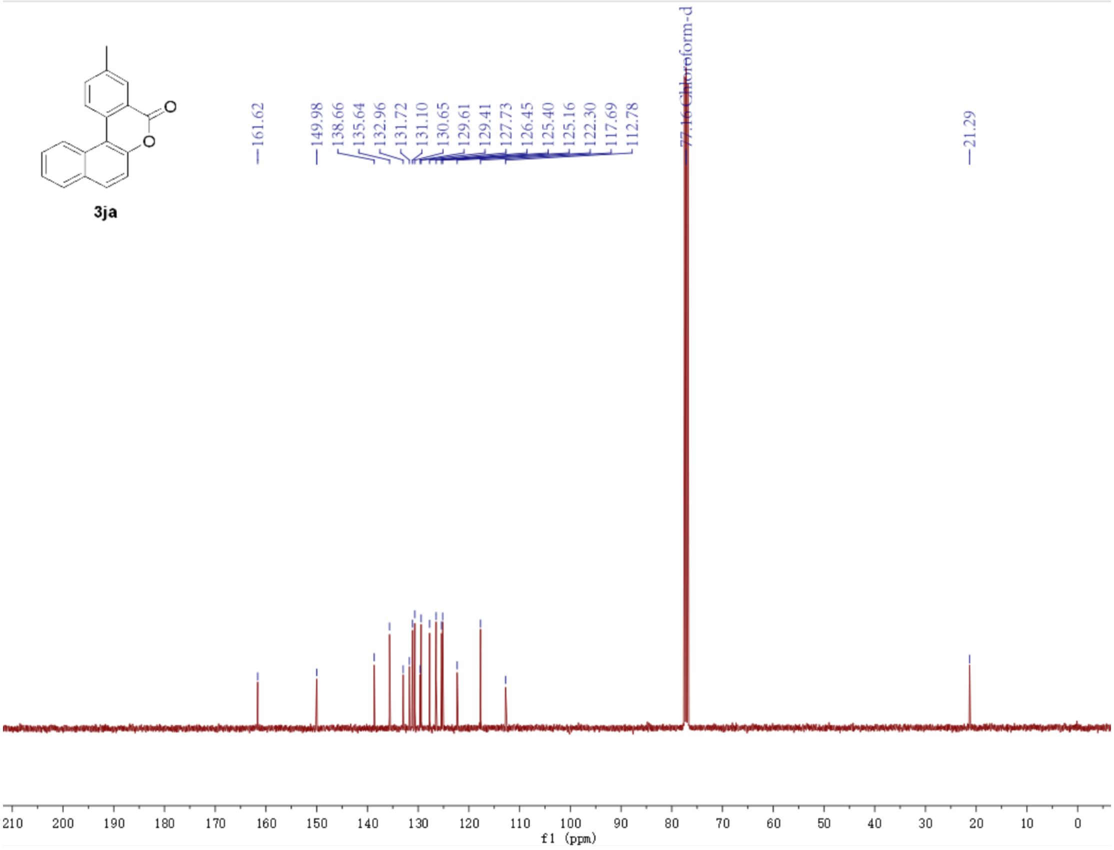

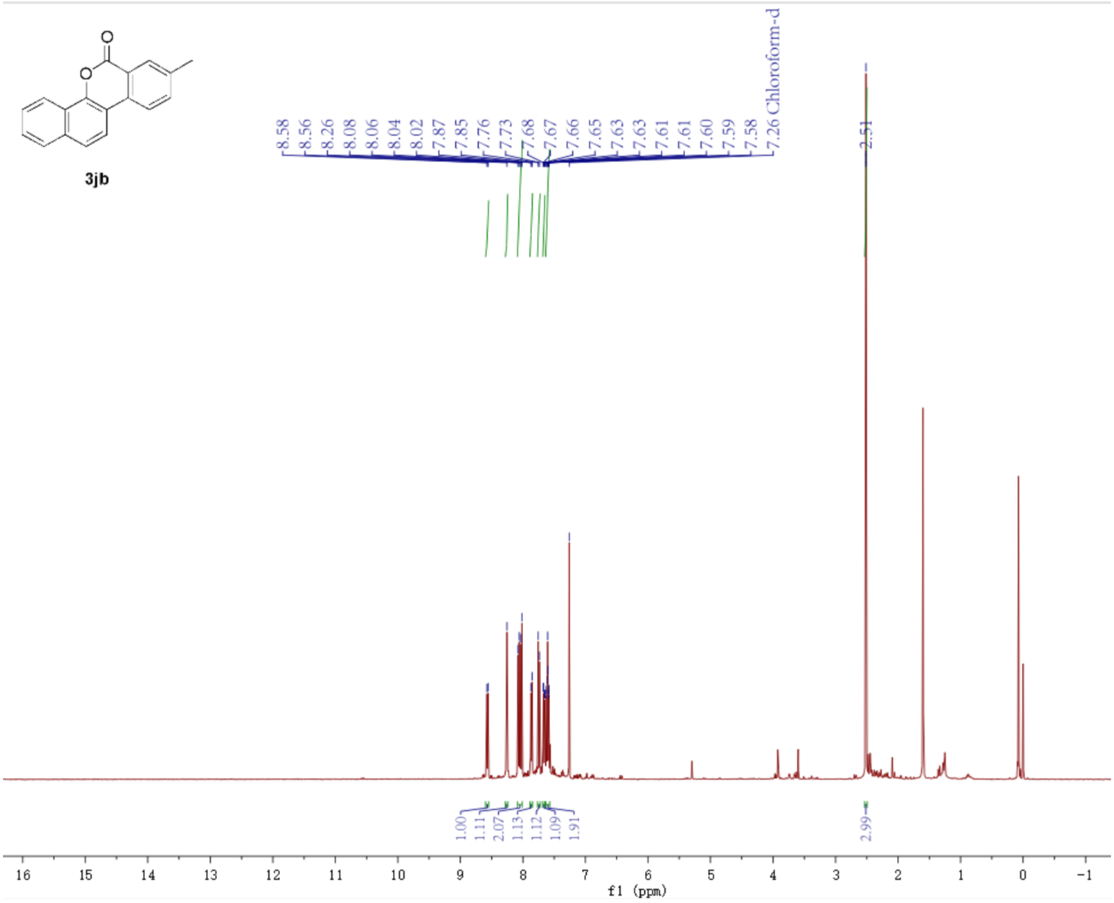
3jb
8.58
8.56
8.26
8.08
8.06
8.04
8.02
7.87
7.85
7.76
7.73
7.68
7.67
7.66
7.65
7.63
7.63
7.61
7.61
7.60
7.59
7.58
7.26 Chloroform-d
2.51
1.00
1.11
2.07
1.13
1.12
1.09
1.91
2.99
f1 (ppm)

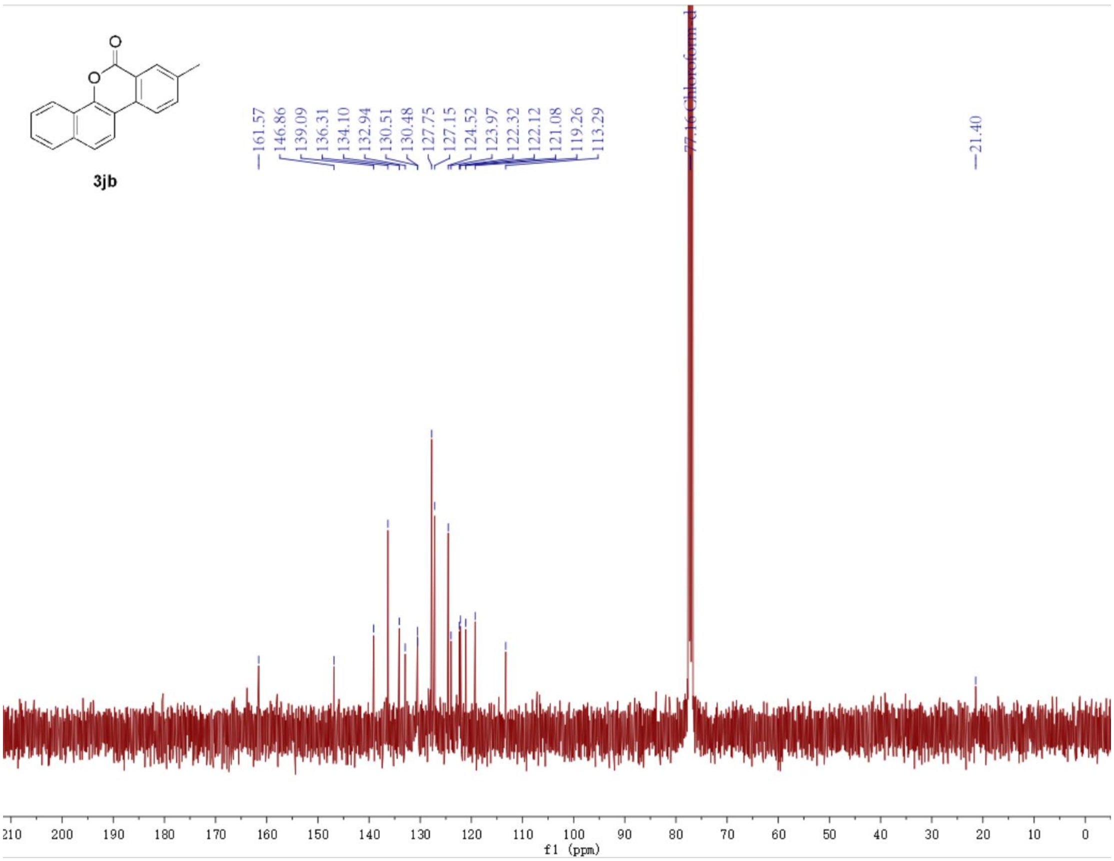
3jb
161.57
146.86
139.09
136.31
134.10
132.94
130.51
130.48
127.75
127.15
124.52
123.97
122.32
122.12
121.08
119.26
113.29
77.16 Chloroform-d
21.40
f1 (ppm)

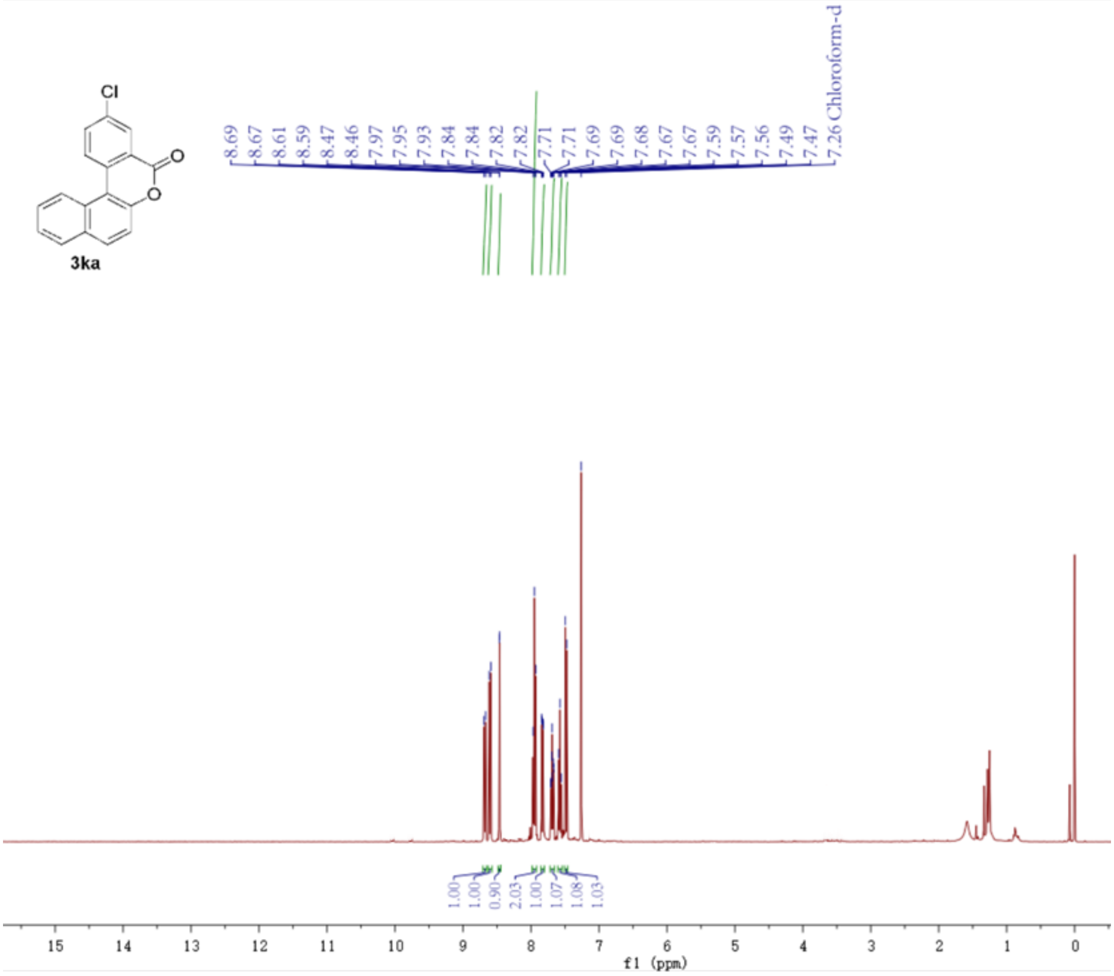
Cl
O
O
3ka
8.69
8.67
8.61
8.59
8.47
8.46
7.97
7.95
7.93
7.84
7.82
7.82
7.71
7.71
7.69
7.69
7.68
7.67
7.67
7.59
7.57
7.56
7.49
7.47
7.26 Chloroform-d
1.00
1.00
0.90
2.03
1.00
1.07
1.08
1.03
15 14 13 12 11 10 9 8 7 6 5 4 3 2 1 0
f1 (ppm)

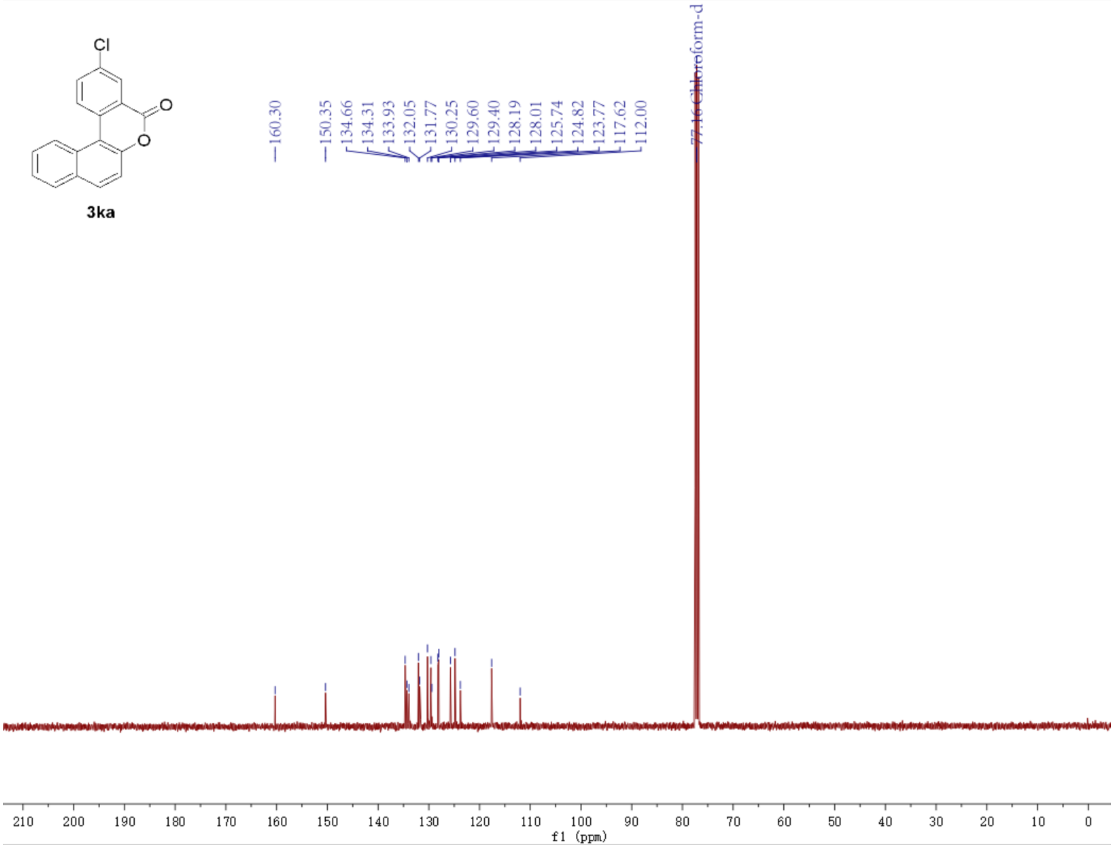
Cl
O
O
3ka
160.30
150.35
134.66
134.31
133.93
132.05
131.77
130.25
129.60
129.40
128.19
128.01
125.74
124.82
123.77
117.62
112.00
77.16 Chloroform-d
210 200 190 180 170 160 150 140 130 120 110 100 90 80 70 60 50 40 30 20 10 0
f1 (ppm)

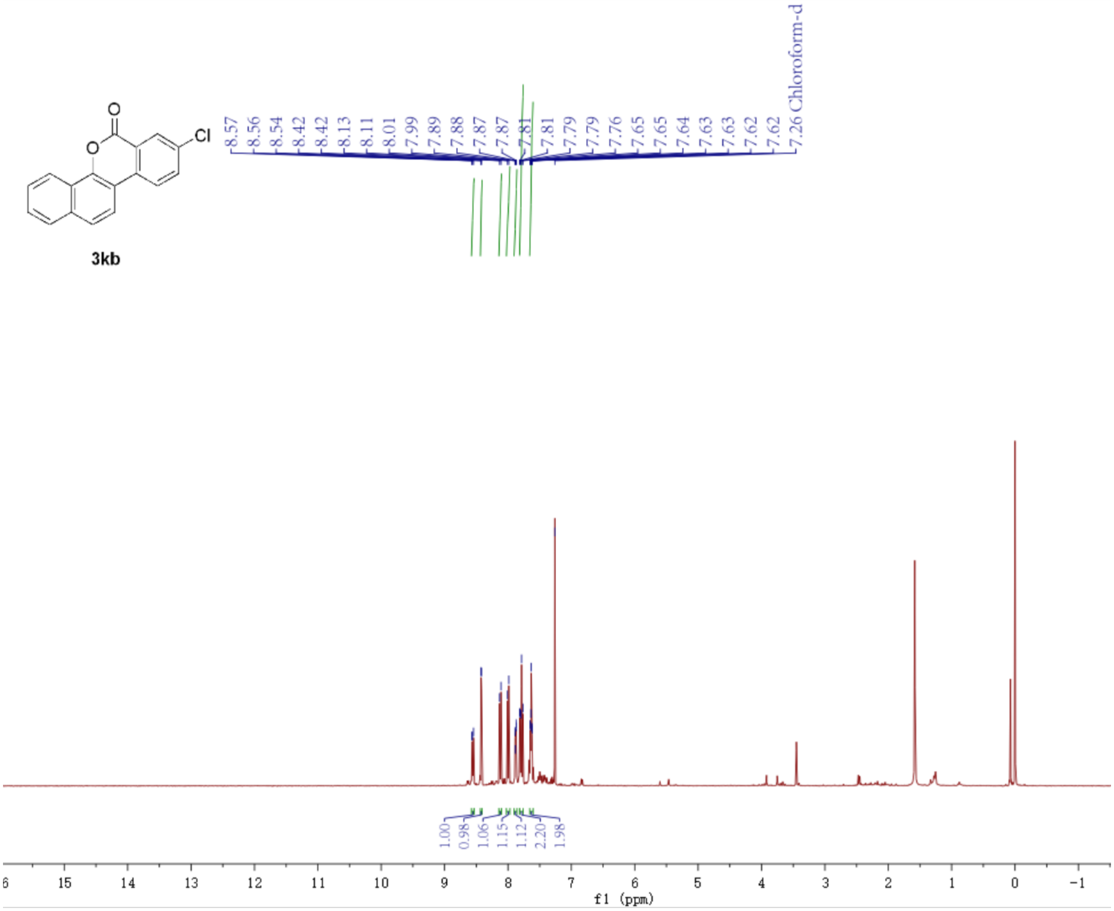

Cl
O
O
3kb
8.57
8.56
8.54
8.42
8.42
8.13
8.11
8.01
7.99
7.89
7.88
7.87
7.87
7.81
7.81
7.79
7.79
7.76
7.65
7.65
7.64
7.63
7.63
7.62
7.62
7.26 Chloroform-d
1.00
0.98
1.06
1.15
1.12
2.20
1.98
f1 (ppm)

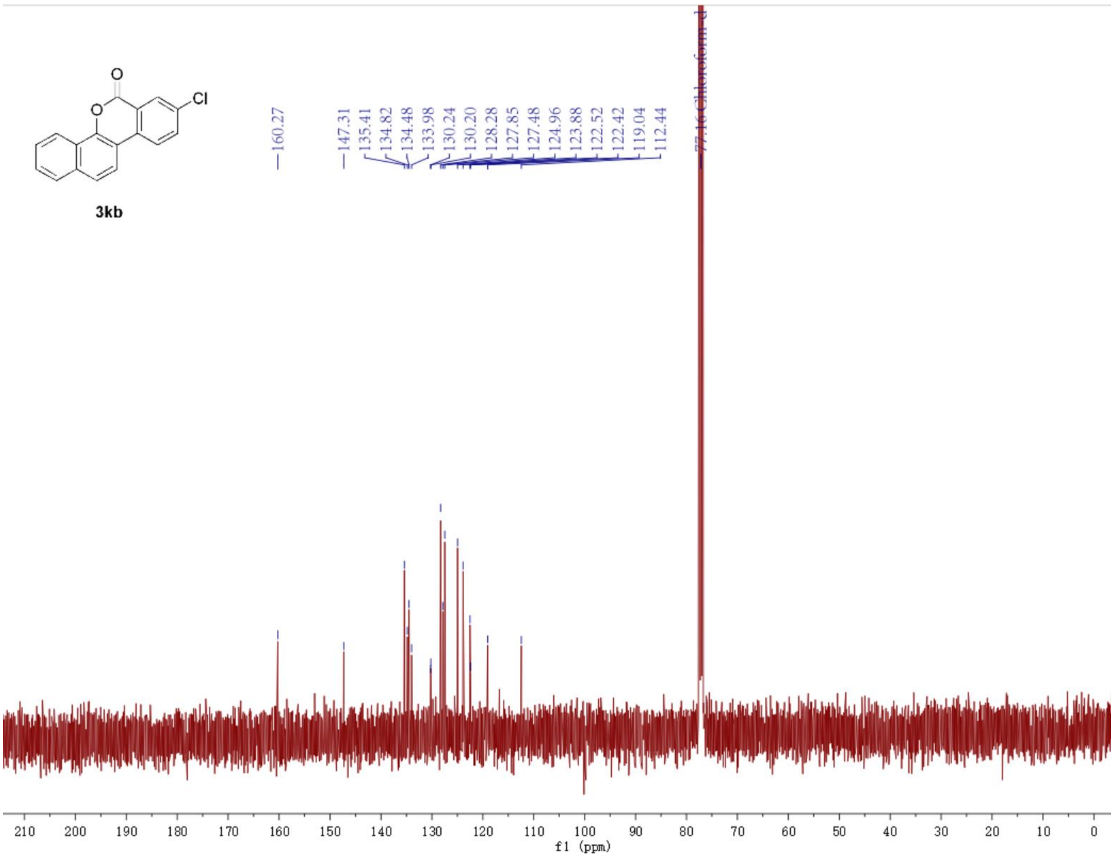

Cl
O
O
3kb
160.27
147.31
135.41
134.82
134.48
133.98
130.24
130.20
128.28
127.85
127.48
124.96
123.88
122.52
122.42
119.04
112.44
77.16 Chloroform-d
f1 (ppm)

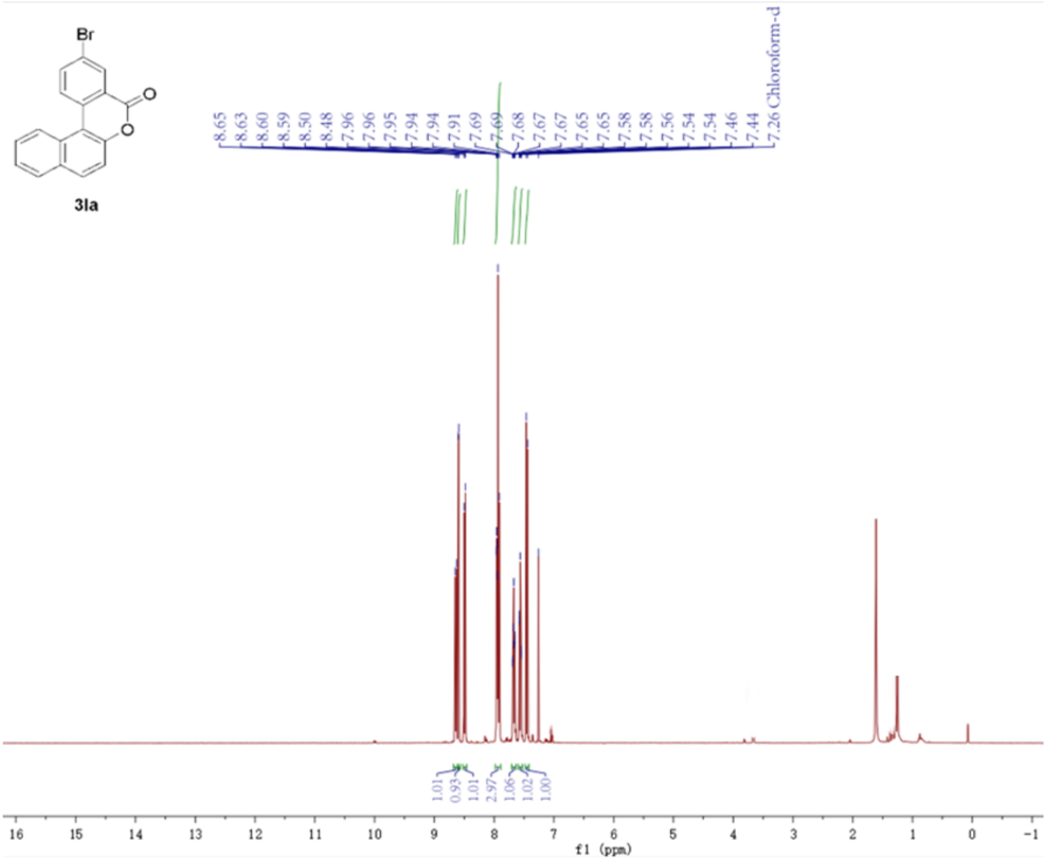
Br
O
O
3la
8.65 8.63 8.60 8.59 8.50 8.48 7.96 7.96 7.95 7.94 7.94 7.91 7.69 7.69 7.68 7.67 7.67 7.65 7.65 7.58 7.58 7.56 7.54 7.54 7.46 7.44 7.26 Chloroform-d
1.01 0.93 1.01 2.97 1.06 1.02 1.00
16 15 14 13 12 11 10 9 8 7 6 5 4 3 2 1 0 -1
f1 (ppm)

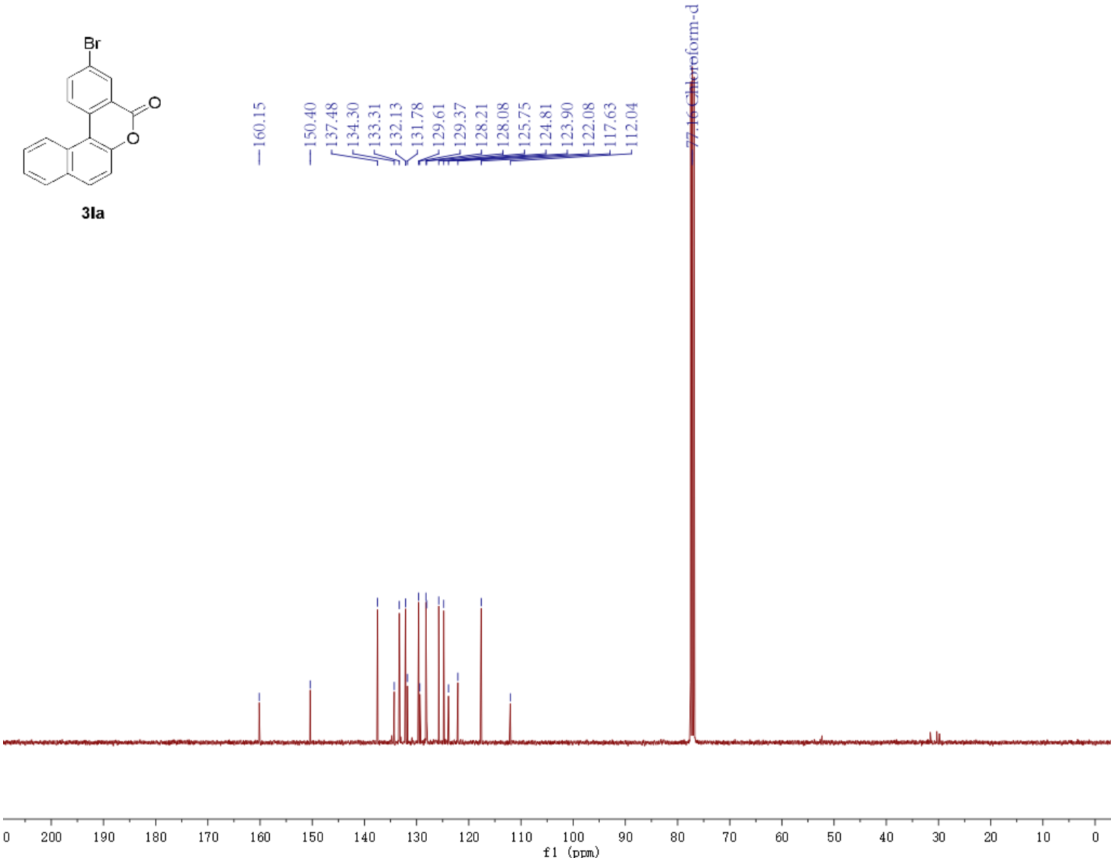
Br
O
O
3la
160.15
150.40
137.48 134.30 133.31 132.13 131.78 129.61 129.37 128.21 128.08 125.75 124.81 123.90 122.08 117.63 112.04
77.16 Chloroform-d
0 200 190 180 170 160 150 140 130 120 110 100 90 80 70 60 50 40 30 20 10 0
f1 (ppm)

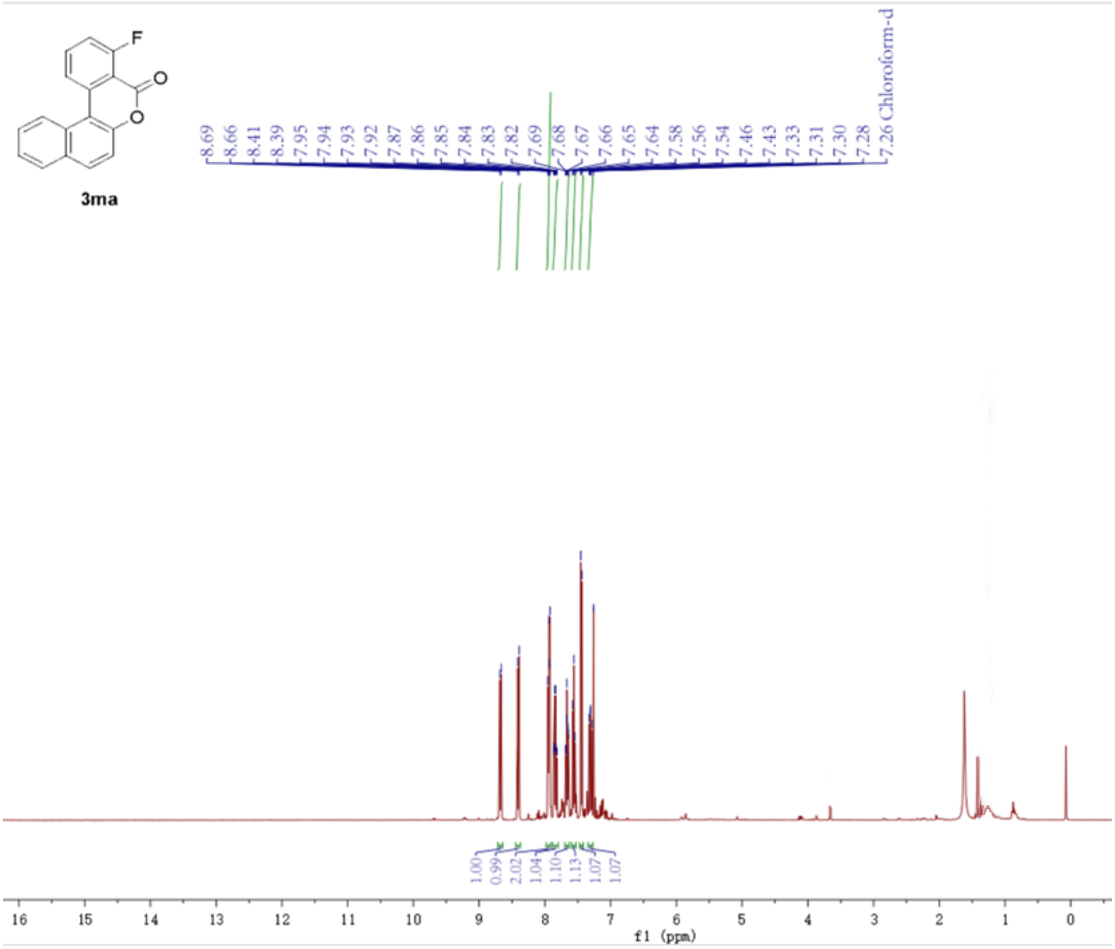
F
O
O
3ma
8.69
8.66
8.41
8.39
7.95
7.94
7.93
7.92
7.87
7.86
7.85
7.84
7.83
7.82
7.69
7.68
7.67
7.66
7.65
7.64
7.58
7.56
7.54
7.46
7.43
7.33
7.31
7.30
7.28
7.26 Chloroform-d
1.00
0.99
2.02
1.04
1.10
1.13
1.07
1.07
f1 (ppm)

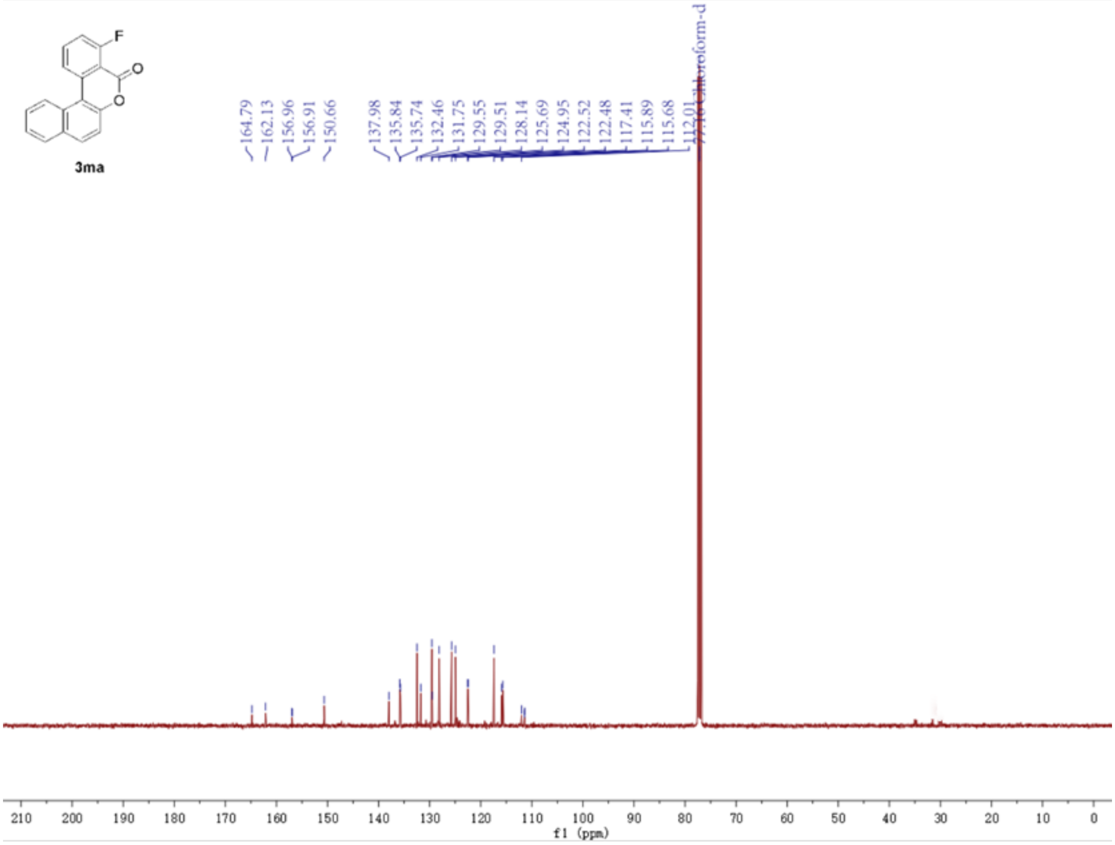
F
O
O
3ma
164.79
162.13
156.96
156.91
150.66
137.98
135.84
135.74
132.46
131.75
129.55
129.51
128.14
125.69
124.95
122.52
122.48
117.41
115.89
115.68
112.01
77.16 Chloroform-d
f1 (ppm)

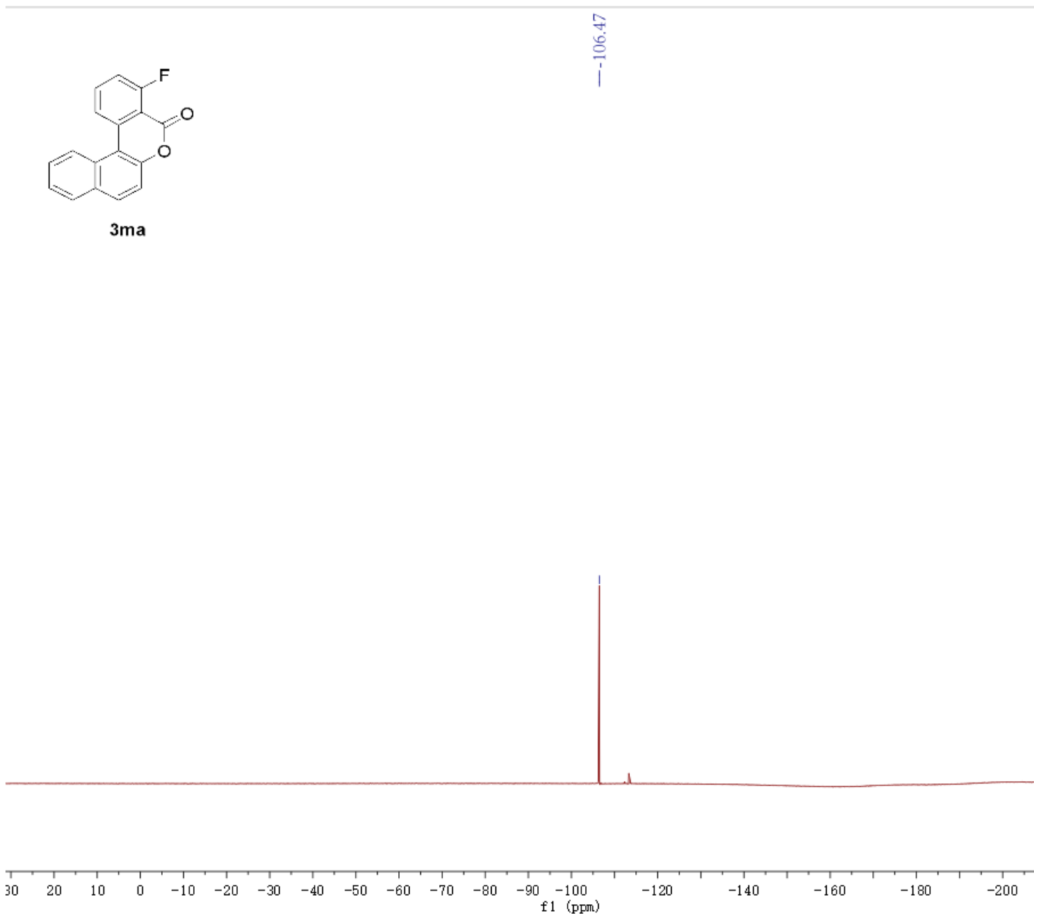
F
O
O
3ma
-106.47
30
20
10
0
−10
−20
−30
−40
−50
−60
−70
−80
−90
−100
−120
−140
−160
−180
−200
f1 (ppm)
